# Lung disease network reveals impact of comorbidity on SARS-CoV-2 infection and opportunities of drug repurposing

**DOI:** 10.1186/s12920-021-01079-7

**Published:** 2021-09-17

**Authors:** Asim Bikas Das

**Affiliations:** Department of Biotechnology, National Institute of Technology Warangal, Warangal, 506004 Telangana India

**Keywords:** COVID-19, SARS-CoV-2, Lung disease, Comorbidity, Disease network

## Abstract

**Background:**

Higher mortality of COVID-19 patients with lung disease is a formidable challenge for the health care system. Genetic association between COVID-19 and various lung disorders must be understood to comprehend the molecular basis of comorbidity and accelerate drug development.

**Methods:**

Lungs tissue-specific neighborhood network of human targets of SARS-CoV-2 was constructed. This network was integrated with lung diseases to build a disease–gene and disease-disease association network. Network-based toolset was used to identify the overlapping disease modules and drug targets. The functional protein modules were identified using community detection algorithms and biological processes, and pathway enrichment analysis.

**Results:**

In total, 141 lung diseases were linked to a neighborhood network of SARS-CoV-2 targets, and 59 lung diseases were found to be topologically overlapped with the COVID-19 module. Topological overlap with various lung disorders allows repurposing of drugs used for these disorders to hit the closely associated COVID-19 module. Further analysis showed that functional protein–protein interaction modules in the lungs, substantially hijacked by SARS-CoV-2, are connected to several lung disorders. FDA-approved targets in the hijacked protein modules were identified and that can be hit by exiting drugs to rescue these modules from virus possession.

**Conclusion:**

Lung diseases are clustered with COVID-19 in the same network vicinity, indicating the potential threat for patients with respiratory diseases after SARS-CoV-2 infection. Pathobiological similarities between lung diseases and COVID-19 and clinical evidence suggest that shared molecular features are the probable reason for comorbidity. Network-based drug repurposing approaches can be applied to improve the clinical conditions of COVID-19 patients.

**Supplementary Information:**

The online version contains supplementary material available at 10.1186/s12920-021-01079-7.

## Background

The novel coronavirus disease 2019 (COVID-19) cases, caused by SARS-CoV-2, crossed 189,000,000 globally as of July 16, 2021. Data show that the most affected groups had two or more pre-existing medical conditions such as hypertension, diabetes, and metabolic, cardiovascular, and digestive disorders [1–3]. Moreover, comorbidity (or existence of multiple disorders) in COVID-19 patients is associated with a higher risk of severe illness, poor prognosis, and high mortality [4]. During viral infection, a virus hijacks the host cell machinery for its replication. Virus–host interactions perturb highly organized host cellular networks and reconstruct different networks favouring virus replication. The topology of molecular interactions is altered in a disease. Hence, the interaction of SARS-CoV-2 with healthy human cells is different from that with disease cells, which thus leads to various impacts on humans after SARS-CoV-2infection. Human diseases are connected via defects in common genes [5, 6]. Moreover, the similarity in disease phenotypes often indicates underlying genetic connections. Therefore, pre-existing medical conditions can facilitate the development of another disease if they share the same or functionally related genes [7, 8]. SARS-CoV-2 has been associated with respiratory tract infections, and in some cases, it severely damages lungs in adult patients. Here, we investigated the underlying molecular link between COVID-19 and lung diseases to understand the basis of comorbidity. In the present study, we have considered a disease in the lung or symptoms in the lung or diseases in other tissues or organs affecting the lungs as a "lung disease." Gordon et al. [9] recently identified 26 of the 29 SARS-CoV-2 proteins that bind to 332 human proteins and hijack the host translational machinery. Here, we constructed a tissue (lungs)-specific neighborhood network of the 332 human targets of SARS-CoV-2. This network was integrated with lung diseases to build a disease–gene network of the lung. Subsequently, we constructed a lung disease network, which also includes COVID-19. In total, 141lung diseases were found to be associated with COVID-19. Among them, 49 were directly linked to COVID-19, apparently justifying the characteristics of a complex disorder. Further, we observed that 59 lung diseases topologically overlapped with COVID-19, indicating a higher risk of comorbidity. This observation also presents the opportunity to repurpose drugs used to treat lung diseases because these drugs can simultaneously hit a lung disease and closely associated COVID-19 module. Moreover, we observed that genes in overlapping lung diseases and COVID-19 are coexpressed and involved in a similar molecular function and biological processes, representing pathobiological similarities between various lung disorders and COVID-19.

Next, we identified functional protein modules that are maximally perturbed by SARS-CoV-2 and involved in RNA processing, export, and protein synthesis machinery of the cell. Moreover, these modules are associated with various lung disorders, indicating the hotspots for comorbidity. Hence, we employed a network-based proximity approach [10] and explored the DrugBank database [11] to identified approved targets in these protein modules that can be hit by existing drugs and rescued from virus possession. Studies have reported that a network-based toolset can be effectively used to identify drugs for COVID-19 treatment [12, 13].We identified 56 druggable human proteins in proximity to the COVID-19 disease module and found that these proteins can be targeted by FDA-approved or investigational drugs. SARS-CoV-2 has a very high mutation rate, which allows it to develop drug resistance [14]. Therefore, identifying and targeting host factors, rather than targeting viral proteins, will be an enduring approach. In summary, this work presents the risk of different lung disorders at COVID-19 onset and drug repurposing opportunities to treat patients with lung disorders.

## Materials and methods

### Construction of a lung-specific PPI network of SARS-CoV-2 targets

Human lung tissue-specific interactome data were retrieved from the TissueNet v.2 database. TissueNet v.2 synergizes between large-scale data of human PPIs and tissue-specific expression profiles to generate tissue-specific PPIs. This database also consolidates PPI data from four major databases, BioGrid, IntAct, MINT, and DIP, and integrates resulting PPIs with RNA-sequencing profiles of the Genotype-Tissue Expression consortium (GTEx). We downloaded 168,296 lung-specific interactions from TissueNet v.2 to construct a SARS-CoV-2 target interactome. Next, we obtained a list of 332 human proteins targeted by SARS-CoV-2, which were identified through affinity-purification mass spectrometry [9]. Using these 332 proteins, we built a subnetwork from 168,296 lung-specific interactions, SARS-CoV-2 target network (STN). Nine SARS-CoV-2 targets (AATF, CEP43, CISD3, MTARC1, NUP62, SRP19, THTPA, TIMM10B, and TRIM59) showed no interaction in the lung.

### Construction of a lung-specific disease–gene and disease–disease network

The disease-gene association data in the lungs were retrieved from the Gene ORGANizer [15], which is a phenotype-based curated database that links human genes to the body parts they affect. Phenotypes classified by Human Phenotype Ontology (HPO) were considered with certain modifications. After disease-gene association data were pre-processed, disease–gene pairs that were not included but matched with the HPO phenotype were manually added. Aspirin-induced asthma and asthma were both considered as asthma. Pulmonary emphysema, sarcoidosis, and silicosis and their associated genes were also added to the list. Finally, 6040 disease–gene pairs and 184 various lung diseases were listed. If a gene is associated with a known lung disorder, then the gene and lung disorder were connected via links. Subsequently, nodes in the STN were linked to the lung disorder to construct the disease–gene association map of the network. Of the 5050 nodes of the STN, 618 were linked to 145 lung diseases. Of the 618 genes, 36 were the direct targets of SARS-CoV-2 and were connected to COVID-19 as a new disease–gene pair. Finally, a lung disease–gene network (LDGN) consisting of 1815 disease–gene pairs, including that for COVID-19, was constructed. The disease–disease association network (DDAN) was derived from the lung disease–gene association network; two diseases were connected if they shared one common gene. The disgenet2r package [16] was used to study the association between disease classes and functional protein modules.

### Network-based separation measure between diseases

To identify the overlapping disease modules, a "separation" measure, *S*_*ab*_ was calculated between COVID-19 (a) and lung disease (b) using the following formula:

$${{\varvec{S}}}_{{\varvec{a}}{\varvec{b}}}=\boldsymbol{ }<{{\varvec{d}}}_{{\varvec{a}}{\varvec{b}}\boldsymbol{ }}>- \frac{<{{\varvec{d}}}_{{\varvec{a}}{\varvec{a}}\boldsymbol{ }}>- <{{\varvec{d}}}_{{\varvec{b}}{\varvec{b}}\boldsymbol{ }}>}{2}$$

${S}_{ab}$ compares the shortest distances between proteins connected to each disease,$<{{\varvec{d}}}_{{\varvec{a}}{\varvec{a}}}>$ and $<{{\varvec{d}}}_{{\varvec{b}}{\varvec{b}}}>$, to those $<{{\varvec{d}}}_{{\varvec{a}}{\varvec{b}}}>$ between a–b protein pairs. Positive ${{\varvec{S}}}_{{\varvec{a}}{\varvec{b}}}$ shows that the two disease modules are separated on the lung interactome, whereas a negative value indicates overlapping modules. The statistical significance of module overlap between COVID-19 and the lung disease was evaluated using the full randomization model. The same number of proteins associated with two diseases was randomly sampled 1000 times, and the corresponding ${S}_{ab}^{ran}$ between the two gene sets was calculated. Next, z-score was calculated as follows:

$$\mathrm{z}-\mathrm{score}=\frac{{S}_{ab}- m}{\sigma }$$

where m and σ indicates the mean value and standard deviation of 1000 ${S}_{ab}^{ran}$. Here, z-score < 0 indicates that the two diseases are closely overlapped than expected by chance [17].

### Community detection

We applied fast-greedy, walktrap, louvain, leading eigenvector, and spinglass on the STN as an undirected, unweighted network. These community detection algorithms segregate the nodes into higher-density modules and optimize an objective function, that is, modularity. Communities separated by spinglass were selected for subsequent analysis based on the modularity score and community size. Spinglass uses a random number generator to find the communities. Therefore, we ran Spinglass 10 times with different seed values. We compared the rand statistics between each run, and results showed that the structures of these communities are highly similar (> 0.7) [18, 19].

### Network-based proximity measure

Network proximity between drug targets (A) and SARS-CoV-2 targets in the host (B) was measured using the closest method (d_c_).

$${d}_{c}=\frac{1}{\Vert A\Vert + \Vert B\Vert }\left(\sum_{a\in A}{min}_{b\in B}d\left(a,b\right)+\sum_{b\in B}{min}_{b\in A}d(a,b)\right)$$

where *d*(*a*, *b*) represents the shortest distance between genes *a* and *b* in the lung interactome. The statistical significance of proximity was evaluated using z-score (z_c_). z_c_ was calculated by comparing the observed distance to a reference distance distribution. To compute reference distance distribution, the sets of proteins of size and degree similar to those of the drug targets and disease proteins were randomly selected for 1000 times from the lung interactome. The mean and standard deviation of distance distribution was calculated to compute z_c_ [10, 20].

### Process and pathway enrichment analysis and gene ontology semantic similarity

Pathway and process enrichment analysis was performed using Metascape [21]. Gene ontology biological processes, KEGG Pathway, and Reactome were used as ontology sources. GO semantic similarity between genes was measured using Wang et al. [22] method with the GOSemSim package in R. Considering that two genes G1 and G2 are annotated by the GO term sets GO1 = [go11, go12, …, go1m] and GO2 = [go21, go22, …, go2n], respectively, their semantic similarity score, which is determined using Wang's method, is defined as follows:

$$\mathrm{Sim}\left(\mathrm{G}1,\mathrm{G}2\right)=\frac{\sum_{1\le \mathrm{i}\le \mathrm{m}}\mathrm{Sim}\left({go}_{1i,}{GO}_{2}\right)+\sum_{1\le \mathrm{j}\le \mathrm{n}}\mathrm{Sim}\left({go}_{2j,}{GO}_{1}\right)}{m+n}.$$

### Correlation analysis

GTEx gene expression datasets of healthy human lung tissues were downloaded from the UCSC Xena project [23]. log_2_(RSEM + 1) (RSEM: RNA-Seq by Expectation Maximization) transformed gene expression data (n = 288) were retrieved, and the Pearson correlation coefficient was computed to measure coexpression levels using the Hmisc package in R.

### Computation of topological parameters

The largest connected component (LCC), dyadicity, and Jaccard similarity coefficient were measured using the igraph package in R. Dyadicity (D) is the number of same label edges divided by the expected number of same label edges, and D > 1 indicates higher connectedness between the nodes with the same label. The Jaccard similarity coefficient of two nodes was calculated as the number of common neighbors divided by the number of nodes that are neighbors of at least one of the two nodes.

### Tools for data analysis, plotting, and statistical analysis

R packages tidyverse and stringr were used for data analysis, and graphs were plotted using ggplot2. Networks were visualized using Gephi. Statistical significance between the groups was analyzed using the non-parametric Mann–Whitney test in R, and that of the overlap between gene lists was analyzed using Fisher's exact test.

## Results

### Construction of SARS-CoV-2–host interactome in the lung

**Fig. 1:**
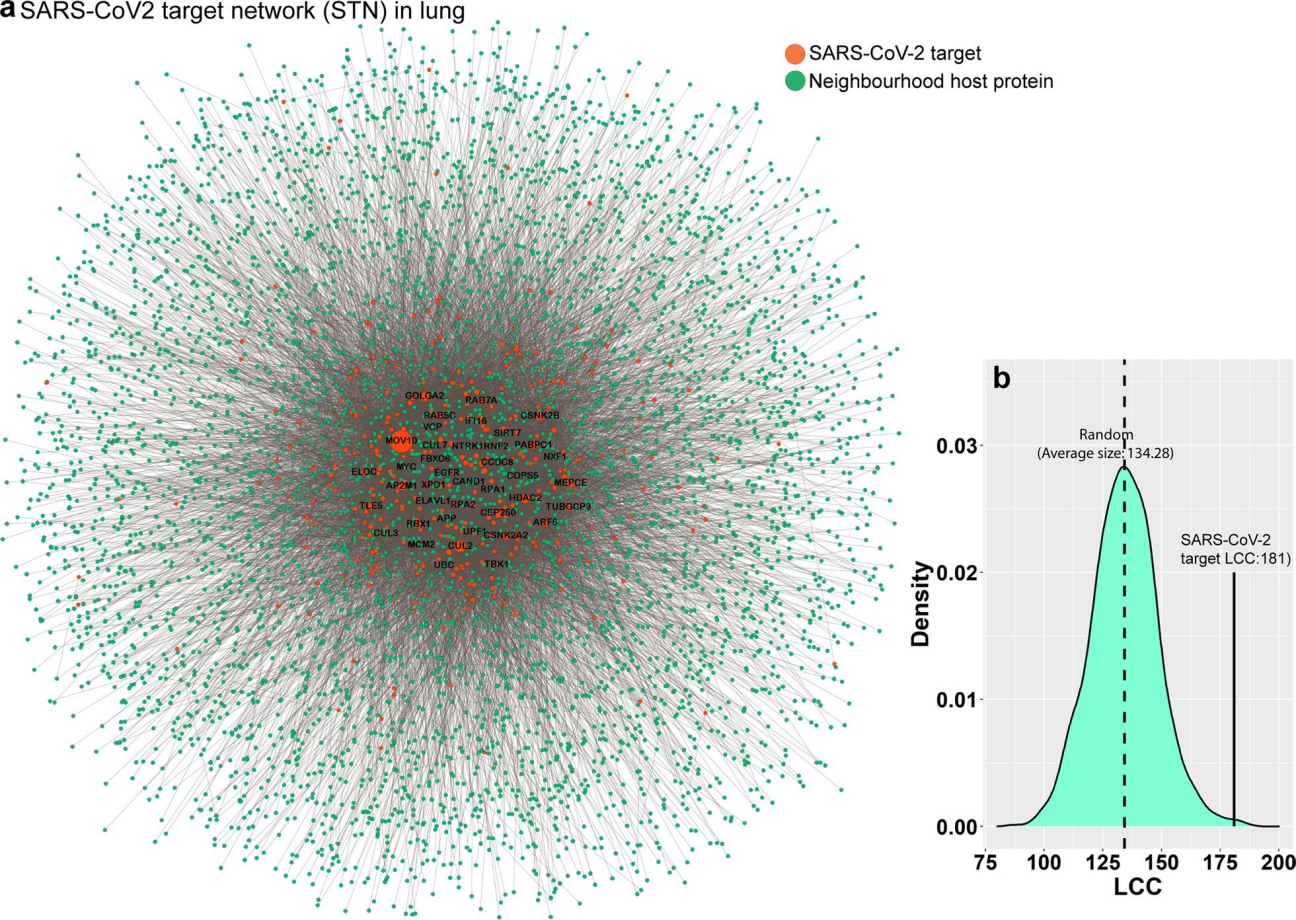
**a** Neighbourhood interaction network of SARS-CoV-2 targets (STN) in the lung. The size of the node is proportional to its degree. **b** SARS-CoV-2 targets form a LCC of size 181 in the lung interactome. The size of the LCC is significantly larger than the random expectation

To depict the SARS-CoV-2–host interaction network, the protein–protein interaction (PPI) network of the lungs (lung interactome) was obtained from the TissueNet v.2 database [24]. We referred to Gordon et al. [9] for the list of 332 human targets of SARS-CoV-2 and constructed a subnetwork of these proteins from the PPI network. Of the 332 viral targets, 323 proteins were present in the subnetwork. The resulting subnetwork, named as the SARS-CoV-2 target network (STN), has 5050 nodes and 11,256 pairwise interactions (Fig. 1a, Additional file 2: Table S1). Next, 181 of the 323 viral targets form the LCC within the lung interactome. To determine the statistical significance of the LCC, we randomly selected proteins with a matching degree and calculated the size of the LCC. We repeated the random selection 1000 times and found that the size of the random LCC was 136.28 ± 16.05 (Fig. 1b), and z-score = 2.78 (p-value = 5.36 × 10^−3^), indicating that the SARS-CoV-2–host interaction network did not appear by chance and the target proteins were located in the same network vicinity [10, 13]. To confirm this result, we computed dyadicity (D) (a measure of the connectedness of the nodes with the same label, see Materials and Methods) among the SARS-CoV-2 targets in the STN to determine if they share more or fewer edges than expected in a random configuration of the network. We found D = 7.664, indicating high connectedness among SARS-CoV-2 targets. D > 1 signifies that the SARS-CoV-2 targets form a community-like structure to hijack the host cellular machinery. If implicated in diseases, proteins in a community confer a higher chance of comorbidity than those not in the community because proteins in a community frequently interact, coexpress, and are functionally interconnected [25]. Therefore, to understand the link between COVID-19 and other lung diseases, we constructed and analyzed the disease–gene and disease–disease association map linked to the STN.

### Disease–gene and disease–disease association map of COVID-19 in lungs

**Fig. 2:**
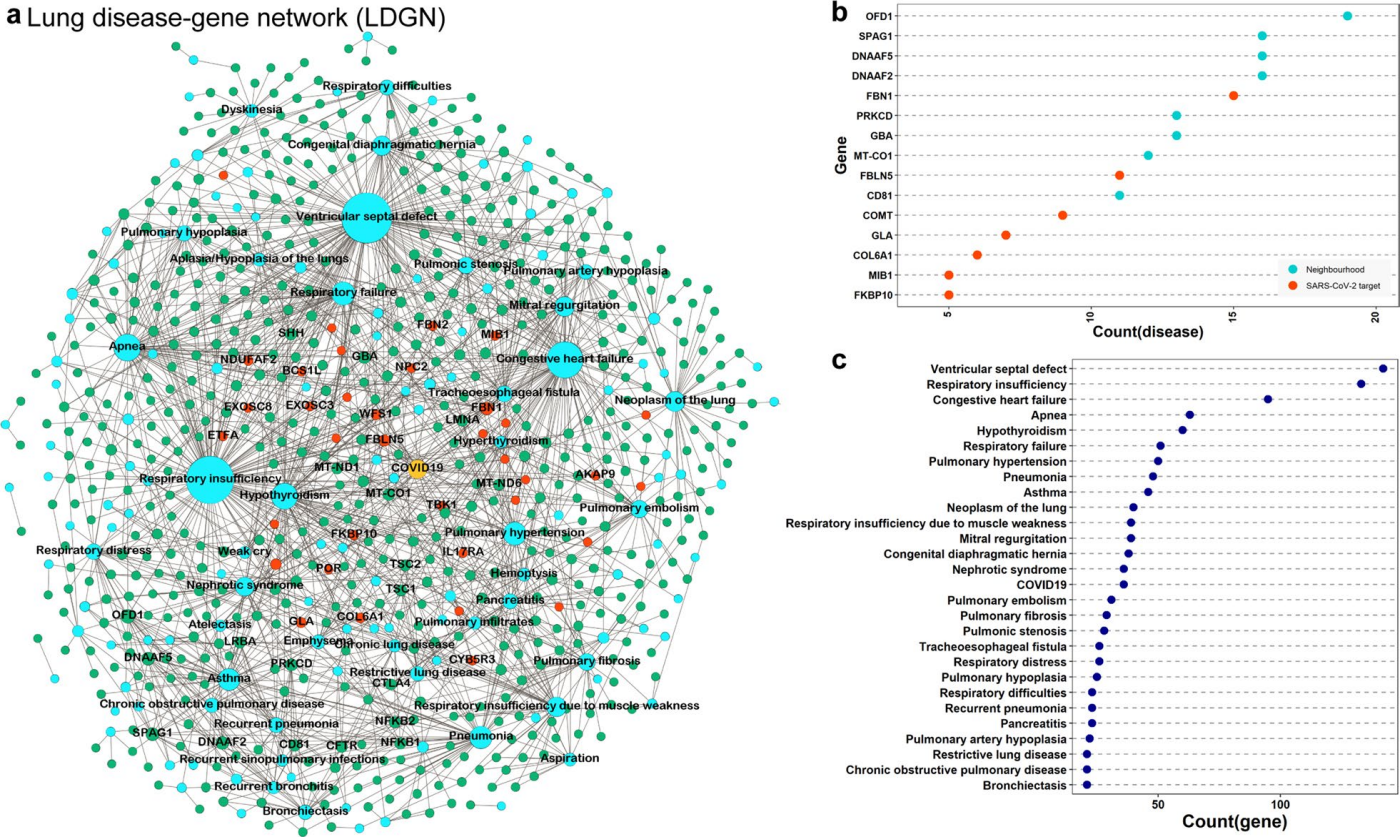
Disease-gene association network. **a** Lung disease-gene network (LDGN), including COVID19 (yellow node). The network shows the SARS-CoV-2 targets (red) and neighborhood genes (green). **b**, **c** Dot plot shows the highly connected diseases (*k* > 20) and genes in LDGN, respectively

To construct a disease association map of the STN, we obtained the disease**-**gene association data from the ORGANizer database [15]. In total, 184 lung diseases, 1957 genes, and 6040 disease–gene pairs were considered for further analysis (see Materials and Methods) (Additional file 3: Table S2). However, 1442 of the 1957 genes are present in the lung interactome. To create the disease–gene association map, we screened the diseases associated with proteins (nodes) in the STN. A disease and gene are then connected if the gene is associated with the lung disorder. We observed that 618 proteins consisting of 36 SARS-CoV-2 targets were linked to 146 disorders, which includes COVID-19 (Additional file 4: Table S3). The overlap between SARS-CoV-2 targets and 1442 lung disease-associated genes was not statistically significant (Fisher's exact test, value = 0.454). Gysi et al. [13] reported a similar observation with a group of genes involved in various disease classes. However, the overlap between 5050 nodes in the STN and lung disease-associated genes was statistically significant (Fisher's exact test, p-value = 2.93 × 10^−5^). Figure 2a shows the resulting disease–gene association map of the STN, named as the lung disease–gene network (LDGN), consisting of1814 disease–gene pairs.

The LCC within the LDGN consists of 141 lung diseases and 610 genes, indicating that many of the disorders share a common genotype. For example, the SARS-CoV-2 targets, FBN1 (degree, *k* = 15), FBLN5 (*k* = 11), and COMT (*k* = 9), and neighborhood nodes, OFD1 (*k* = 19), DNAAF2 (*k* = 16), and DNAAF5 (*k* = 16), are linked to multiple disorders (Fig. 2b). Similarly, a disorder in the LDGN is also connected with multiple genes [e.g., ventricular septal defect (*k* = 142), respiratory insufficiency (*k* = 133), congestive heart failure (*k* = 95), apnea (*k* = 63), and hypothyroidism (*k* = 60) (Fig. 2c, Additional file 1: Fig. S1 and Fig. S2)].

**Fig. 3:**
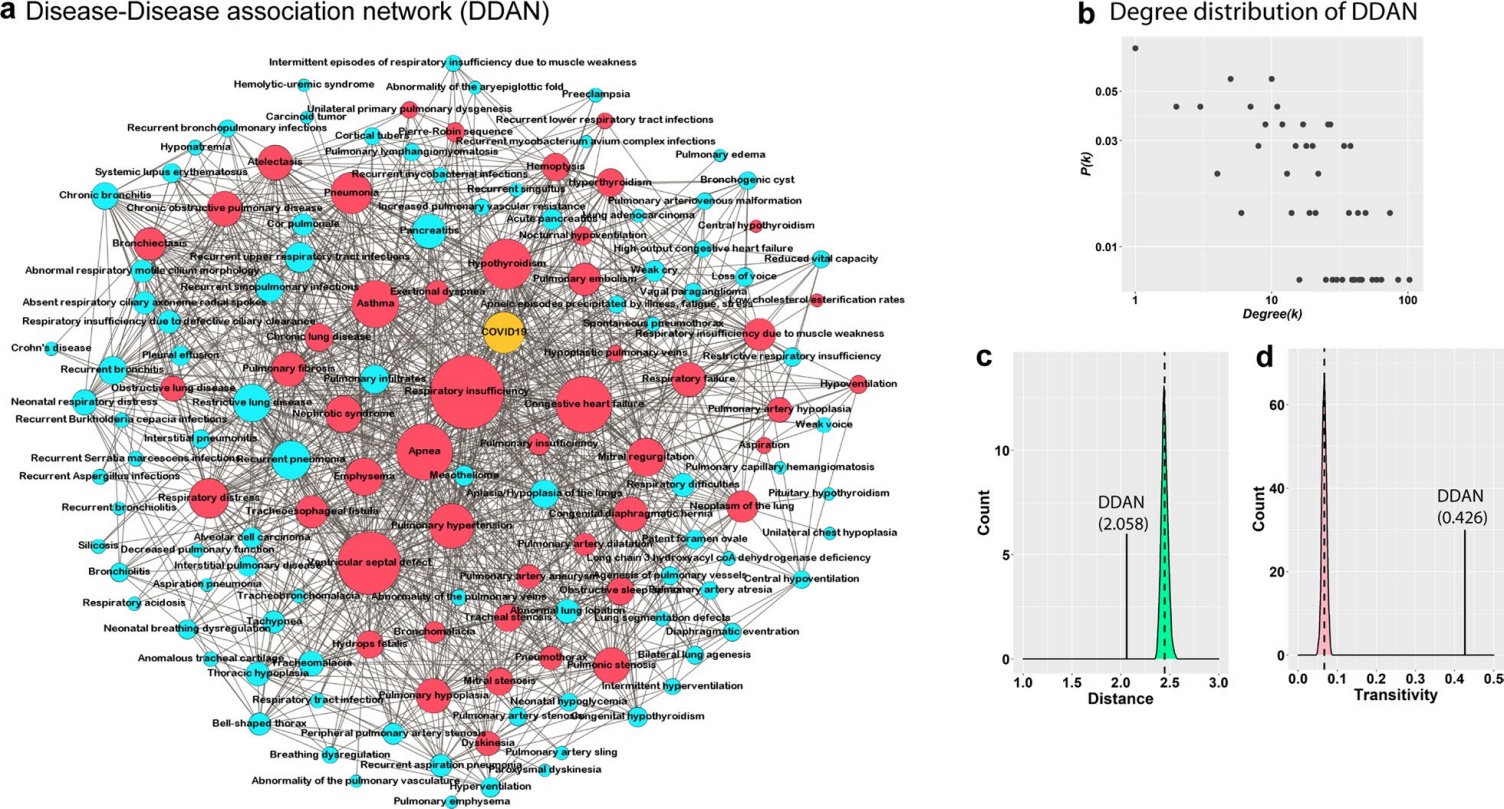
Disease-disease association network (DDAN). **a** DDAN, including COVID19, red nodes represent the diseases that are directly direct linked to COVID19. **b** Scatter plot shows the degree distribution of DDAN, which does not follow the scale-free property. **c** The average path length between the diseases in DDNA and distribution of average path length of 1000 random networks (green). **d** Transitivity of DDNA and distribution of transitivity of 1000 random networks (pink)

The disease-gene association pattern in the LDGN indicates the presence of a molecular connection between COVID-19 and a wide range of lung disorders. To comprehend this connection, a disease-disease association network, DDAN was constructed, where two diseases were linked if they share one associated gene (Fig. 3a). DDAN consists of 141 diseases (nodes) and 1326 links, indicating a higher clustering between diseases. Further, the degree distribution of the DDAN did not follow the scale-free property (Fig. 3b). To determine the exact topological nature, we measured network transitivity (${T}_{DDAN}$=0.4264) and average path length (${L}_{DDAN}$=*2.0585*) of the DDAN and compared them with the equivalent 1000 Erdős − Rényi random graphs. The results showed that the average path length is significantly lower (p-value < 0.0001), whereas transitivity is significantly higher (p-value < 0.0001) than random graphs (${L}_{random}=2.44680$ and ${T}_{random}=0$.0668*)* (Fig. 3c, d). Further, we calculated the small-worldness scalar (*S*) for the DDAN as follows:

$$\gamma =\frac{{T}_{DDAN}}{{T}_{random}}=6.383$$

$$\lambda =\frac{{L}_{DDAN}}{{L}_{random}}=0.841$$

$$S=\frac{\gamma }{\lambda }=7.589$$

A network is considered a small-world network if *S* > 1 [26]. Hence, the topology of the DDAN represents a small-world property, indicating that any two diseases in this network have a high tendency to be interconnected and may cause the overlapping disease pathogenesis.

Forty-nine diseases in the DDAN were directly connected to COVID-19. Using the number of common genes, the Jaccard similarity coefficient was computed to identify the extent of molecular overlap between the 49 lung diseases and COVID-19 (Additional file 1: Fig. S3). Several diseases, such as respiratory insufficiency, congestive heart failure, respiratory failure, ventricular septal defect, mitral regurgitation, and hyperthyroidism, are closely associated with COVID-19. Of note, although molecular connections exist between COVID-19 and various lung diseases, these overlaps are not statistically significant (considering only SARS-CoV-2 targets). Nevertheless, these molecular connections are crucial for analyzing the effect of SARS-CoV-2 infection on lung patients; however, opportunities to comprehend disease comorbidity are limited with these molecular connections.

### Topological overlap between disease modules, pathobiological similarities, and opportunities for drug repurposing

**Fig. 4:**
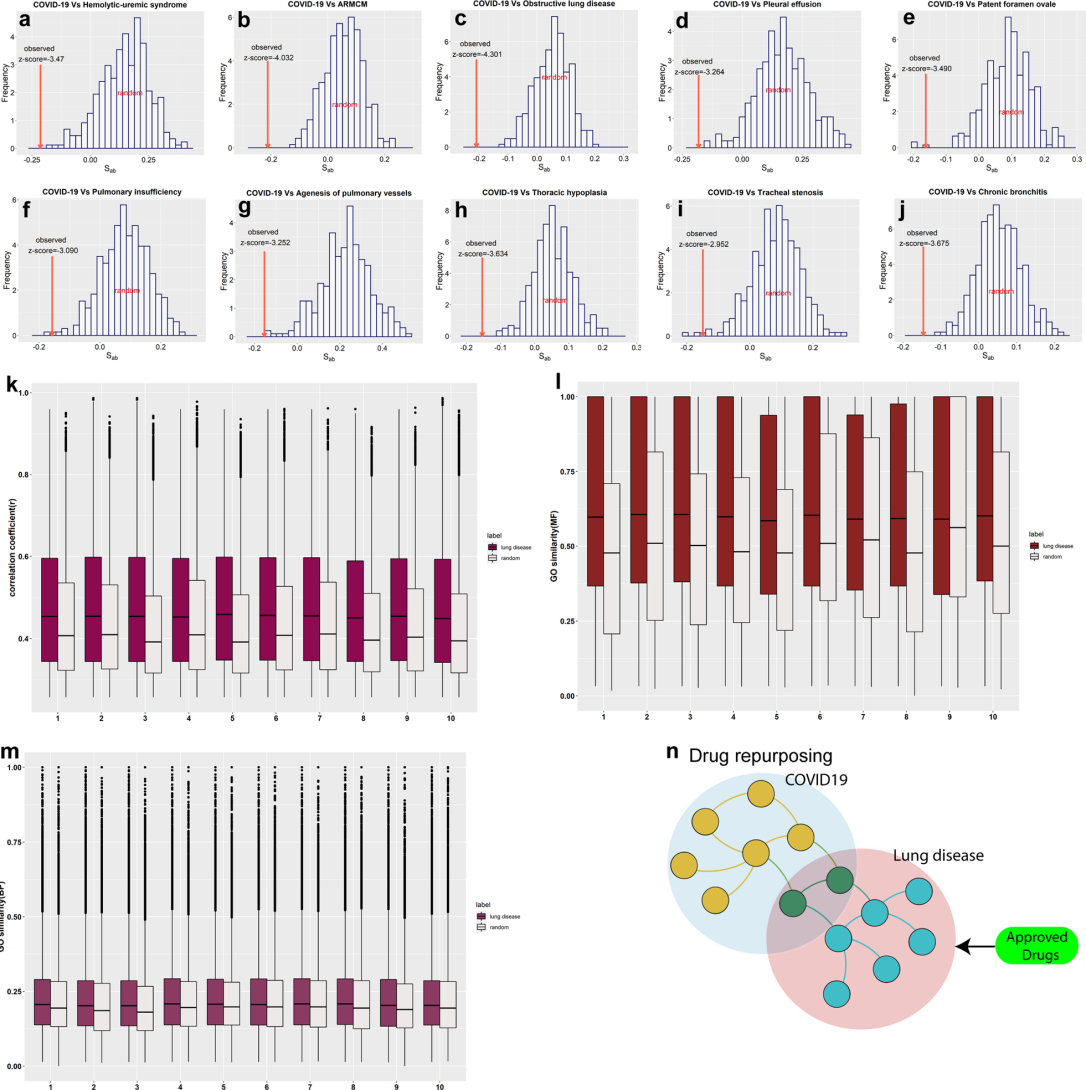
Network-based separation (${S}_{ab}$) and pathobiological similarities. **a**–**j** shows observed ${S}_{ab}$, z-score (red arrow) and distribution of ${S}_{ab}^{ran}$ of top 10 overlapping lung diseases with COVID-19 (here, ARMCM indicates abnormal respiratory motile cilium morphology). **k** Box plot represents the pairwise correlation between genes is significantly (p-value < 0.0001) higher than the random gene sets. **l**, **m** Box plots show the distribution of functional similarities (MF) and GO processes (BP) between the genes involved in lung disease and COVID-19. The GO processes and functional similarity between the genes are significantly high (*p* < 0.0001) compared to the random gene sets (note, in figures **k**, **l**, and **m** 1–10 indicates disease in a similar sequence as it is mentioned in figures **a** to **j**). **n** The strategy of drug repurposing to target the COVID19 module

For a greater understanding of comorbidity, we measured the network-based separation between two disease modules to comprehend their degree of overlap. The network-based separation measure is primarily advantageous because it can predict disease–disease association, even if two diseases share no genes. If two disease modules overlap, then perturbations to one disease can cause disturbance to another, indicating that they have similar clinical characteristics. The magnitude of the overlap indicates the biological and pathobiological similarities between the two disease modules [17]. Network-based separation ($${S}_{ab}$$) (see Materials and Methods) between COVID-19 and all lung diseases was measured. Of the184 lung diseases, 59 demonstrated overlapping modules ($${S}_{ab}<0$$) with COVID-19 (Additional file 5: Table S4). The statistical significance of $${S}_{ab}$$ for each disease pair, that is, COVID-19 and each lung disease, was evaluated using a full randomization model. We observed, for all 59 diseases, the z-score was < 0, indicating that these diseases are closely overlapped with COVID-19 than expected by chance. Figure 4a–j shows the top 10 closely overlapping lung disease modules with COVID-19 (e.g., hemolytic-uremic syndrome ($${S}_{ab}=-0.2142$$), abnormal respiratory motile cilium morphology (ARMCM;$${S}_{ab}=-0.21138$$), obstructive lung disease ($${S}_{ab}=-0.21022$$), pleural effusion ($${S}_{ab}=-0.18216$$), patent foramen ovale (PFO,$${S}_{ab}=-0.1619$$), and pulmonary insufficiency ($${S}_{ab}=-0.15694$$). Thus, patients with these disorders are probably more vulnerable to COVID-19 symptoms or vice versa because of overlapping disease modules. The same set of genes induce ARMCM, absent respiratory ciliary axoneme radial spokes, and respiratory insufficiency, which are caused because of defective ciliary clearance; therefore, we considered only ARMCM in the top 10 list. According to the network-based separation measure, almost 32% of lung diseases have overlapping modules with COVID-19 and the remaining 68% are topologically separated. To understand the biological relationship and pathobiological similarities, the expression correlation and semantic similarity **(**molecular functions and biological processes) of genes involved in COVID-19 and overlapping lung diseases ($${S}_{ab}<0$$).) were measured. Gene coexpression and semantic similarity were significantly (p-value < 0.0001) higher compared to those in the random control (Fig. 4k–m), indicating the biological and pathobiological similarities between COVID-19 and overlapping lung diseases. To further investigate the similarities in clinical features, results from recent publications were explored. Reports have raised concerns about lung injuries linked to COVID-19 [27, 28]. A higher percentage of COVID-19 patients in severe conditions are more likely to develop chronic obstructive pulmonary disease (COPD) and impairment of diffusion capacity [4, 29]. Many lung diseases ($${S}_{ab}<0)$$ (Additional file 5: Table S4) with a overlapping module with COVID-19 are linked to these aforementioned phenotypes. A disease closely associated with COVID-19, haemolytic-uremic syndrome $${(S}_{ab}<0)$$ (Fig. 4a), causes pulmonary hemorrhage, which is linked to kidney failure [30], and studies have recently reported that chronic kidney diseases and chronic pulmonary disease cause adverse outcomes in COVID-19 patients [4, 31]. Abnormal respiratory motile cilium (Fig. 4b) or ciliary dyskinesia ($${S}_{ab}=-0.21138$$) causes chronic respiratory tract infections because the improper movement of mucus restricts the complete elimination of fluid, bacteria, and particles from the lungs, leading to bronchitis (chronic bronchitis, $${S}_{ab}=-0.146)$$(www.ghr.nlm.nih.gov). Lack of respiratory clearance in a patient with ciliary dyskinesia could confer a higher risk of health hazard after SARS-CoV-2 infection. Another study showed that patients with obstructive lung disease ($${S}_{ab}=-0.21$$) (Fig. 4c) and pulmonary emphysema ($${S}_{ab}=-0.09)$$ are at a higher risk of pneumothorax after SARS-CoV-2 infection [32]. Rajendram et al. [33] predicted that PFO (Fig. 4e) may be common in COVID-19 patients because PFO causes pulmonary embolism [34]. Even the disease module of pulmonary embolism overlapped ($${S}_{ab}=-0.008 )$$ with COVID-19. A clinical study in Wuhan, China [35] reported that almost 5% COVID-19 patients had pleural effusion($${S}_{ab}=-0.18 )$$ (Fig. 4d), which is often caused by congestive heart failure and blood clots in lung arteries. Importantly, pleural effusion is commonly associated with age-related respiratory problems and cancer [36]. On the other hand, congestive heart failure, which causes many lung-related diseases [37], also overlapped with the COVID-19 disease module (Additional file 5: Table S4). A meta-analysis by Alqahtani et al. [38] demonstrated that the risk of more severe COVID-19 was higher in patients with COPD (risk of severity = 63%) than in those without (33.4%). Although these results suggest the clinical similarities between COVID-19 and overlapping lung disorders, they are limited and cannot be extrapolated for all overlapping lung disorders without clinical evidence. Furthermore, a genome-wide association study has presented the genetic susceptibility locus in the chromosome of patients with COVID-19 and respiratory failure [39], and genes present in this locus (SLC6A20, LZTFL1, and CCR9) were also associated with different lung disorders ($${S}_{ab}<0)$$ such as pulmonary fibrosis, respiratory distress, asthma, and nephrotic syndrome.

The availability of efficient drugs for the treatment of clinically characterized lung diseases having overlapping neighborhoods with COVID-19 has shown the scope for repurposing these drugs for COVID-19treatment. When two diseases are localized in the same network vicinity and overlap with each other, then targeting one disease can affect another disease module (Fig. 4n), leading to efficient clinical outcomes for both as they have common network neighborhoods [20]. Clinical data from the Clinicaltrials.gov database show that some drugs used for lung diseases, such as methylprednisolone for tracheal stenosis (${S}_{AB}=-0.14915$) and ketamine and budesonide for COPD are in clinical trials for COVID-19 treatment [40]. Therefore, existing drugs that are presently used for treating lung disorders on COVID-19 patients must be tested for better clinical outcomes. Treating a comorbid patient is challenging, but an accurate clinical picture of patients, molecular signature of diseases, and drug target information can improve the present crisis.

### Functional protein modules preferentially hijacked by SARS-CoV-2 are linked to a broad range of lung disorders

**Fig. 5:**
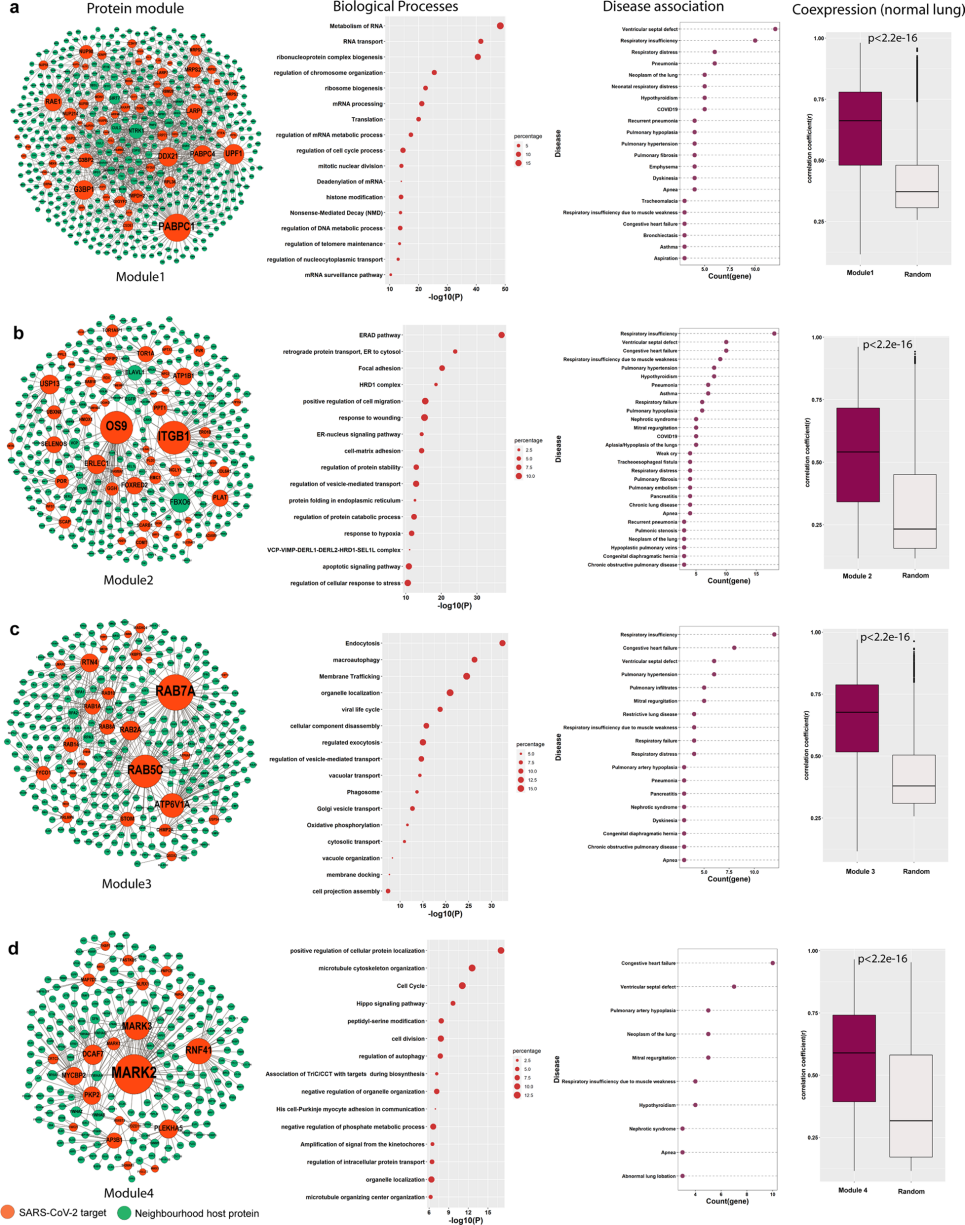
Community detection in STN and functional protein module. **a**–**d** show the modules 1 to 4, pathway and process enrichment analysis of each module, their disease associations, and positive correlation between genes in each module in healthy lung tissue. The pairwise correlation between genes in each module is significantly (p-value < 2.2 × e^−16^) higher than the random gene sets

Modularity in the network refers to the pattern of connectedness in which nodes are grouped into highly connected subsets [41]. A key feature in the PPI network is that tightly connected proteins within a community are mostly involved in similar biological functions [42]. Similarly, genes involved in related diseases are highly connected; moreover, diseases linked to common genes result in the formation of disease modules and comorbidity [43]. We compared various community detection algorithms, that is, fast-greedy, walktrap, louvain, leading eigenvector, and spinglass, to identify protein modules in the STN [19, 44]. Spinglass showed good partitioning with a higher modularity score compared with other algorithms (see “Materials and Methods” section and Additional file 6: Table S5). Our findings are in agreement with those of the study by Rahiminejad et al. [18], where good partitioning of the functional protein module was observed using spinglass in eukaryotes. Of the 21 modules, the top 4 protein modules were selected according to the presence of a large number of SARS-CoV-2 targets (> 20) and a gene ontology semantic similarity score (> 0.2) of biological processes (Additional file 6: Table S6). Numerous viral targets were considered because these modules are largely hijacked and strongly perturbed after infection compared with other functional modules in the network. The modules were named as modules 1, 2, 3, and 4, and each module contains 63, 50, 28, and 23 SARS-CoV-2 target protein**s**, respectively (Fig. 5). The biological process and pathway enrichment analysis showed that module1, the largest module, is mainly enriched with RNA metabolism, including transcription, mRNA processing, transport, mRNA de-adenylation, and surveillance. Presumably, biological processes linked to module1 are hijacked by SARS-CoV-2 in the early stage of infection for viral RNA production. Notably, the components of module1 are linked to 64 disorders, among which the highly connected are respiratory insufficiency, ventricular septal defect, respiratory distress, pneumonia, and lung neoplasm (Fig. 5a, 3rd column, Additional file 7: Table S7). Most module 1-associated diseases are directly connected to COVID-19 (Fig. 3b, Additional file 1: Fig. S2). On the other hand, hijacking module2 can predominantly affect protein degradation (ERAD pathway, HRD1 complex, and regulation of the protein catabolic process), transport, folding, and stability (retrograde protein transport, regulation of protein stability, VCP-VIMP-DERL1-DERL2-HRD1-SEL1L complex, regulation of intracellular transport, regulation of vesicle-mediated transport, and protein folding in the endoplasmic reticulum). Module3 and module4 involve several processes, primarily cellular transport, localization, organization, and cell cycle. Modules 2, 3, and 4 were linked to 79, 60, and 32 different disorders, respectively (Additional file 7: Table S7). The disease association of all four protein modules was significantly higher (p-value < 0.0001) than 1000 random gene sets. Moreover, a broad spectrum of disorders of various classes, such as neoplasms, neurological, and digestive systems, was associated with these modules (Additional file 1: Fig. S4). Gysi et al. [13] predicted that the manifestation of SARS-CoV-2 in different human tissues could cause various disorders. Therefore, not only lung-related disorders, but diseases in other organs can also be a potential threat for COVID-19 patients. To confirm this observation, gene coexpression pattern in functional modules was analyzed. Genes in the same functional module often show a high coexpression profile, which indicates their involvement in similar biological processes. Therefore, we calculated Pearson correlation coefficients of gene pairs using gene expression data of healthy lung tissues from GTEx. The median value of the positive correlation between the genes in all modules was significantly higher (p-value < 0.0001) than that for the random gene set (Fig. 5, 4th column). Therefore, these modules can be identified as coexpression modules that share core transcriptional programs in the lung, which indicates that their perturbation can lead to a similar disease phenotype. Next, we used drug repurposing to find the targets to hit functional modules.

### Drug repurposing to target functional modules

**Fig. 6:**
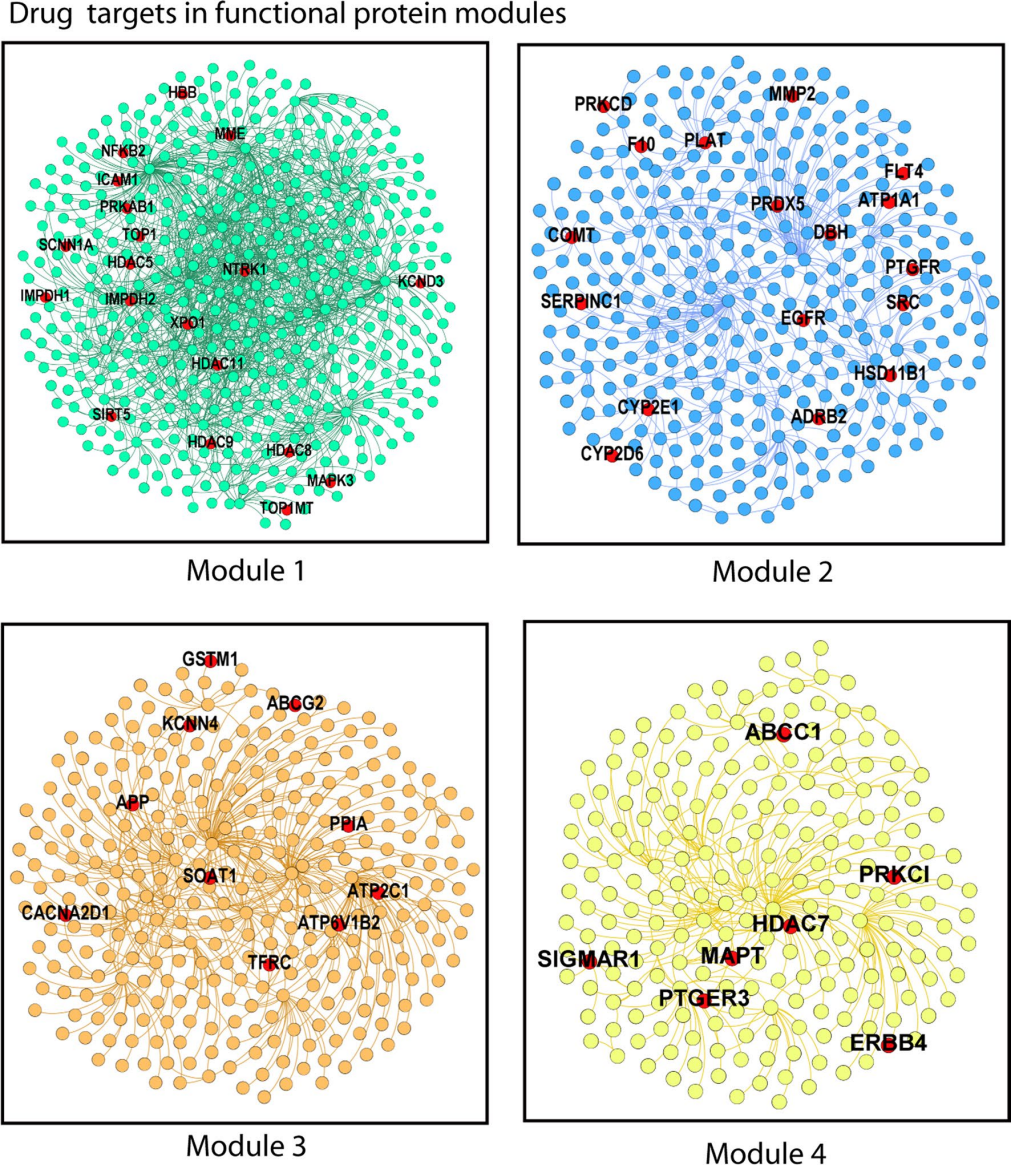
Targetable protein in functional modules: The red nodes in each module indicate the FDA-approved targets

We propose targeting functional protein modules hijacked by SARS-CoV-2, by drug repositioning. There are two main reasons to target these modules. First, the binding of a drug to its target in a module will prevent virus replication. Second, as a module is linked to several lung diseases, targeting a module can reduce severity in patients. From Drug Bank, we identified 56 approved targets (red color nodes in Fig. 6) that can be hit by 144 approved or investigational drugs in the clinical trial (Additional file 8: Table S8) [11]. The list contains10 approved drugs at different stages of clinical trials for COVID-19 treatment, including chloroquine targeting Glutathione S-transferase Mu 1 in module3 (Additional file 1: Fig. S5). However, the efficacy of chloroquine on COVID-19 patients is arguable. Coagulation factor X (F10) was observed in module2, which has recently been implicated as a target due to the potential role of coagulopathy in COVID-19 [45]. To determine the effectiveness of targets, we applied network-based proximity measures to calculate the proximity between the COVID-19 disease module (network among SARS-CoV-2 targets) and FDA-approved targets in the functional module. We used the "closest" (d_c_) measure, representing the closest path length between a target and the nearest SARS-CoV-2 target protein. Then, we calculated z-score (z_c_) to validate the proximity by comparing the observed target–disease protein distance to the random expectation [10]. All 56 targets were proximal (z_c_ <  − 2) to the COVID-19 disease module (Additional file 9: Table S9) compared to the random expectation. Next, approved drugs in clinical trials for COVID-19 treatment (Additional file 1: Fig. S5) were also significantly closer to the COVID-19 disease module (z_c_ < − 2) (Additional file 10: Table S10). The highly connected (hub) targets in the functional module, such asNTRK1 (*k* = 43) and IMPDH2 (*k* = 37) in module1, as well as PLAT (*k* = 17) and COMT (*k* = 10) in module2, can be considered as potential targets for COVID-19 treatment (Additional file 1: Fig. S5). Considering the complexity of COVID-19, different locations in the STN must be targeted as this may help efficiently rewire the cellular network [46] and rescue these functional modules from the virus, thereby reducing virus growth [20]. Many of the target proteins suggested do not directly interact with SARS-CoV-2; instead, they are neighborhood nodes. The binding of drugs to these targets present in the same network vicinity may efficiently perturb the network modules, including viral growth [47]. Notably, this article presents the computation analysis; therefore all drug-target combinations should be tested on SARS-CoV-2-infected cell lines and validated through clinical trials.

## Discussion

Currently, a speedy drug discovery is urgently required to stop the infection and rapid transmission of SARS-CoV-2. Aged COVID-19 patients with comorbidity are at severe health risks worldwide. The present study evidenced the risk of COVID-19 at the onset of various lung-related disorders and the molecular basis of comorbidity by applying the principle of network biology. COVID-19 can be considered as complex disease because of wide-ranging SARS-CoV-2 targets in the host cell, which thus establishes the molecular connection with various lung-related disorders. The disease-gene, disease–disease association map, and network separation analysis have revealed molecular links and clustering of diseases in the same network vicinity, indicating a pathobiological similarity between COVID-19 and various lung disorders. Some diseases closely associated with COVID-19 are haemolytic-uremic syndrome, obstructive lung disease, pleural effusion, and chronic bronchitis. Because of the close association, the pre-existence of these diseases can lead to higher mortality of COVID-19 patients. Severity of one of the common respiratory problems, asthma, which has an overlapping disease module and is directly connected to COVID-19, becomes moderate to high with SARS-CoV-2 infection (www.cdc.gov). These observations provide a detailed understanding of the molecular basis of severe illness in COVID-19 patients with specific lung disorders and help us decipher the patient-specific etiology of COVID-19. Because of multiple molecular connections and overlapping disease modules of COVID-19 with various lung disorders, finding specific targets and potential drugs for COVID-19 patients with pre-existing medical conditions is challenging. The present crisis cannot wait until new drugs arrive; therefore, we proposed two approaches for drug repositioning. The first approach is testing drugs approved for lung diseases having modules overlapping with COVID-19. These drugs can simultaneously affect two disease modules, leading to much-improved treatment outcomes. The second approach is targeting host functional protein modules that are linked with many lung disorders and are primarily hijacked by SARS-CoV-2. Perturbing these modules by repurposing FDA-approved (or investigational) drugs may rescue the host cellular machinery utilized for virus replication. Considering the complexity of SARS-CoV-2 infection, we suggest hitting multiple targets in different functional modules to improve clinical outcomes. However, systematic studies through clinical trials for identifying drug combinations and their targets are highly recommended to increase clinical efficacy and lower toxicity [20]. Moreover, patient-specific high-throughput transcriptomics data or construction of a weighted gene expression networks from SARS-CoV-2-infected lung tissues can further the possibility of target identification, like in other human diseases [48, 49]. In addition, Mendelian randomization study can be performed to understand the causal relationships between lung diseases and susceptibility and severity of COVID-19 [50, 51]. Lastly, the experimental validation of our observation and in vitro or in vivo assays of drug combination and study of pharmacokinetics are warranted to establish a proper treatment strategy.

## Conclusion

In summary, this study used the network biology framework to elucidate the molecular link between lung disorders and COVID-19. The network-based separation measure identified 59 lung diseases topologically overlapped with the COVID-19 module. In addition, the Disease-disease association network showed forty-nine diseases were directly connected to COVID-19. This revealed the cause of severe illness of patients with respiratory problems after SARS-CoV-2 infection. Genes in functional protein modules, hijacked by SARS-CoV-2, are coexpressed and connected to several lung diseases. The perturbation of these modules may block the virus growth in the host cells. Therefore, existing FDA-approved drugs can target the hijacked protein modules to avoid the life-threatening situation of COVID-19 patients with lung disorders.

## Supplementary Information

**Additional file 1**. **Fig.S1**. Dot plot shows the number of genes associated with a lung disorder in LDGN. **Fig. S2**. Dot plot shows the number of shared genes between COVID-19 other lung disorders. **Fig.S3**. The network view of the Jaccard similarity coefficient between lung diseases and COVID19. **Fig.S4**. Heat map shows functional protein modules are associated with different disease classes. **Fig.S5**. Drug repurposing to target functional protein modules.

**Additional file 2**. Table S1. Edge list of SARS-CoV-2 target network (STN) from TissueNet v.2 database.

**Additional file 3**. Table S2. Disease-gene association data of lungs from the ORGANizer database.

**Additional file 4**. Table S3. Disease-gene association data of STN.

**Additional file 5**. Table S4. Network-based separation measure between disease modules and statistical significance.

**Additional file 6**. Table S5 and S6. Comparison of different community detection algorithms applied to SARS-CoV2 target network (STN) and protein modules generated using Spinglass algorithem.

**Additional file 7**. Table S7. Disease association with functional protein modules.

**Additional file 8**. Table S8. Targets in functional protein modules and drugs from DrugBank database.

**Additional file 9**. Table S9. Proximity between targets and COVID-19 disease module.

**Additional file 10**. Table S10. Proximity between drugs in clinical trial and COVID-19 disease module.

## Data Availability

Human targets of SARS-CoV-2 are available at https://www.nature.com/articles/s41586-020-2286-9#Sec36. Lung tissue-specific network is available at TissueNet v.2 (https://netbio.bgu.ac.il/labwebsite/tissuenet-v-2-download/). Disease-gene association data is available in Gene ORGANizer (http://geneorganizer.huji.ac.il/downloads/). Genotype-Tissue Expression (GTEx) data of healthy human lung tissues is available at UCSC Xena (https://xenabrowser.net/datapages/?dataset=gtex_RSEM_Hugo_norm_count&host=https%3A%2F%2Ftoil.xenahubs.net&removeHub=https%3A%2F%2Fxena.treehouse.gi.ucsc.edu%3A443). The datasets generated after analysis are available in Additional files.

## Abbreviations

COVID-19: Coronavirus disease 2019
SARS-CoV-2: Severe acute respiratory syndrome coronavirus 2
PPI: Protein–protein interaction
STN: SARS-CoV-2 target network
DDAN: Disease–disease association network
LDGN: Lung disease–gene network
LCC: Largest connected component
D: Dyadicity
HPO: Human Phenotype Ontology
GO: Gene ontology
