# Supplementary material for: Lung disease network reveals impact of comorbidity on SARS-CoV-2 infection and opportunities of drug repurposing
Source: BMC Med Genomics. 2021 Sep 17;14:226. doi: 10.1186/s12920-021-01079-7 (PMC8447809; doi:10.1186/s12920-021-01079-7)
Supplement: Supplementary file 1 — Additional file 1. Fig.S1. Dot plot shows the number of genes associated with a lung disorder in LDGN. Fig. S2. Dot plot shows the number of shared genes between COVID-19 other lung disorders. Fig.S3. The network view of the Jaccard similarity coefficient between lung diseases and COVID19. Fig.S4. Heat map shows functional protein modules are associated with different disease classes. Fig.S5. Drug repurposing to target functional protein modules. [file 12920_2021_1079_MOESM1_ESM.docx]

**Supplementary Figures**

**Lung Disease Network Reveals Impact of Comorbidity on SARS-CoV-2 Infection and Opportunities of Drug Repurposing**

Asim Bikas Das


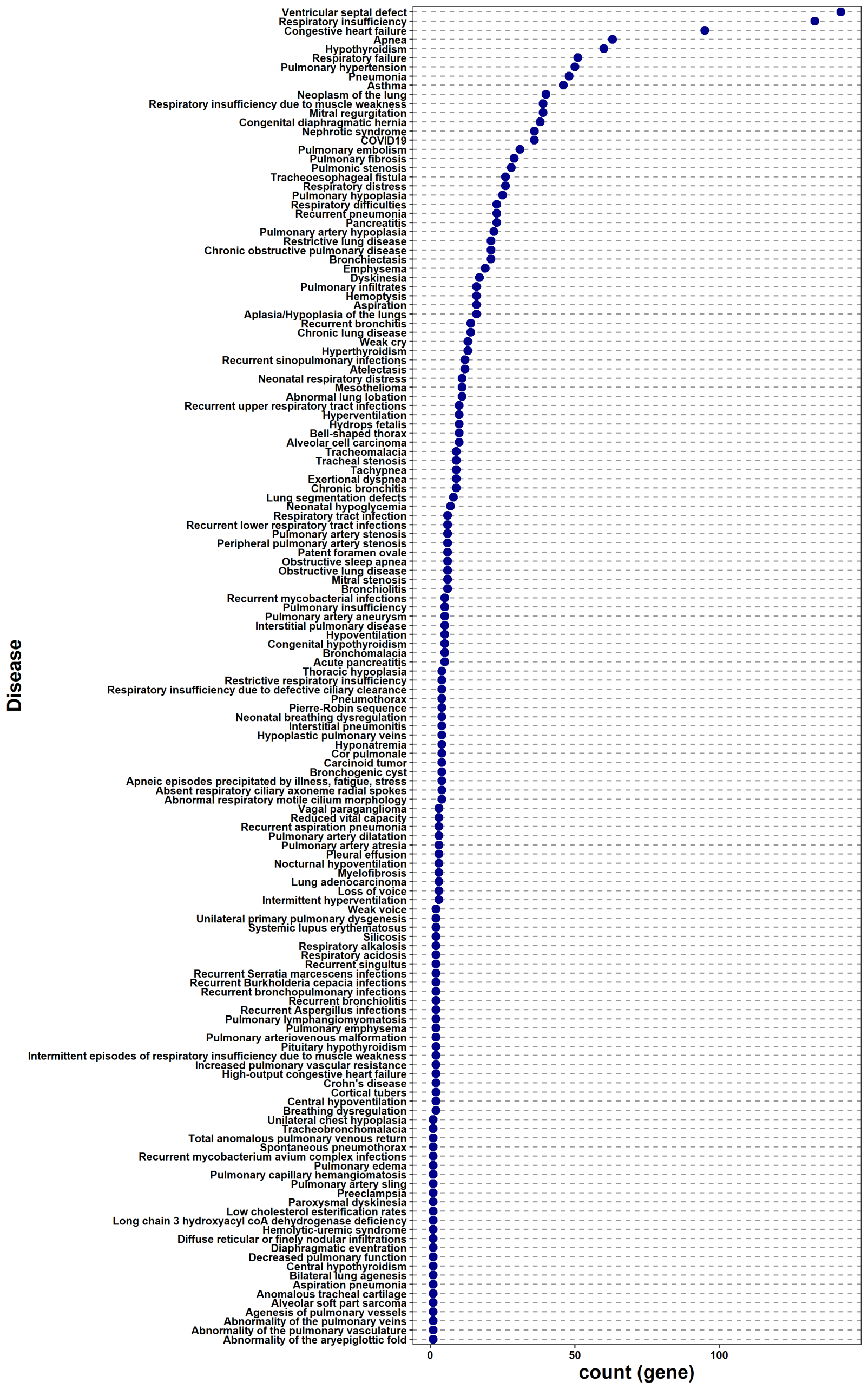


**Supplementary Fig.S1 (extended figure of Fig.2c):** Dot plot shows the number of genes associated with a lung disorder in LDGN.

**
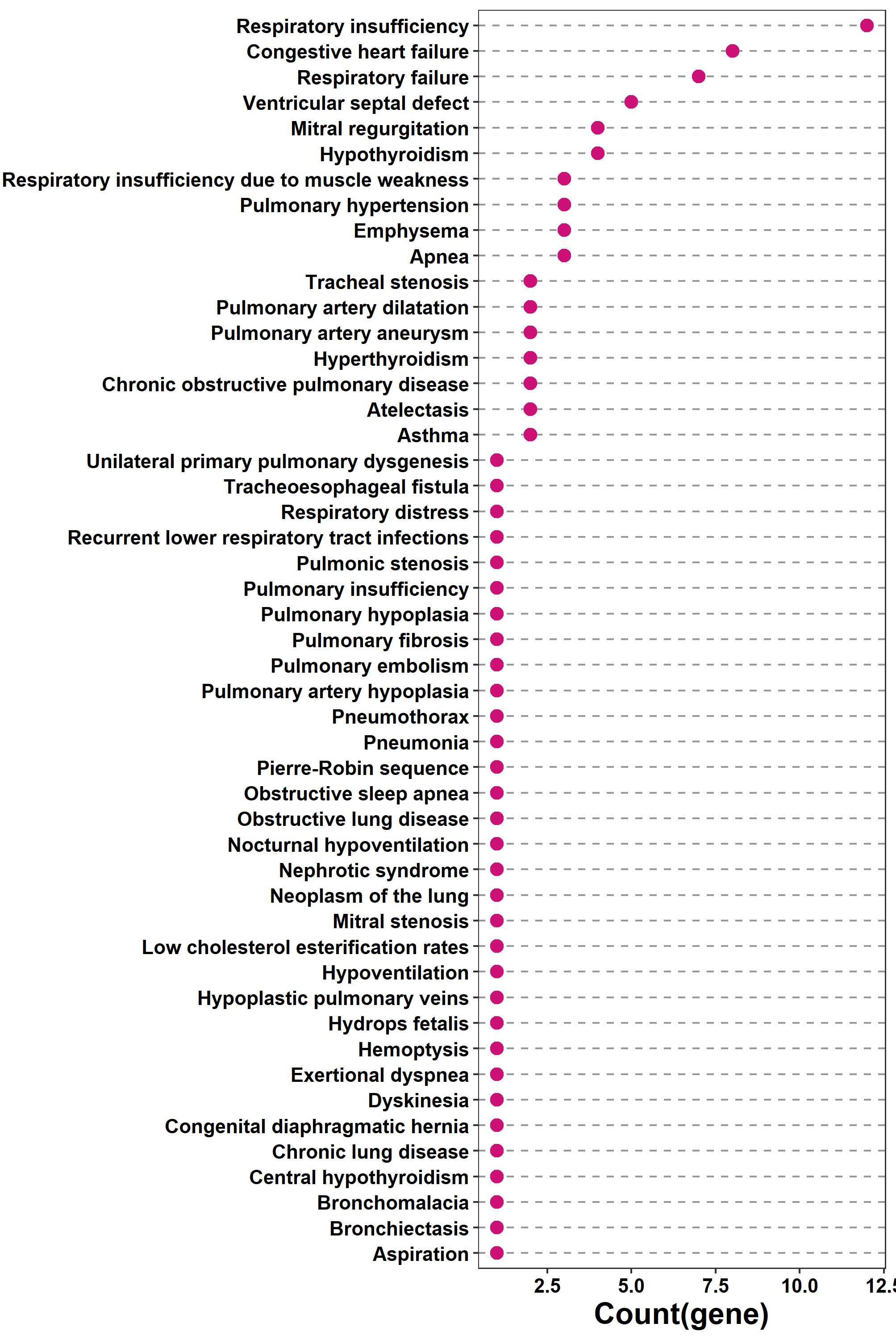
**

**Supplementary Fig. S2** Dot plot shows the number of shared genes between COVID-19 other lung disorders.

**Supplementary Fig.S3** The network view of the Jaccard similarity coefficient between lung diseases and COVID19. The thickness of the edge is proportional to the Jaccard coefficient.

**
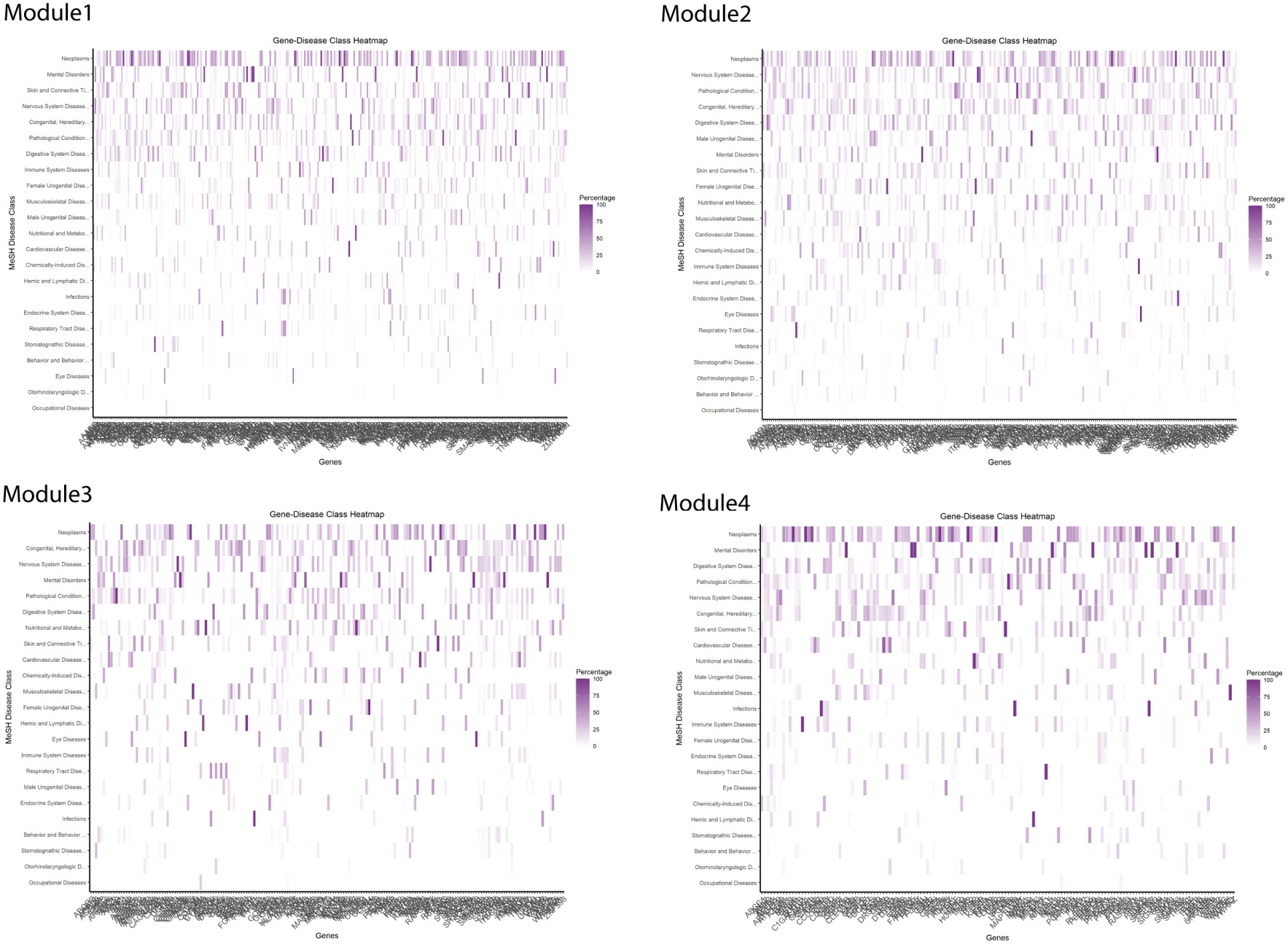
**

**Supplementary Fig.S4** Heat map shows functional protein modules are associated with different disease classes.

**
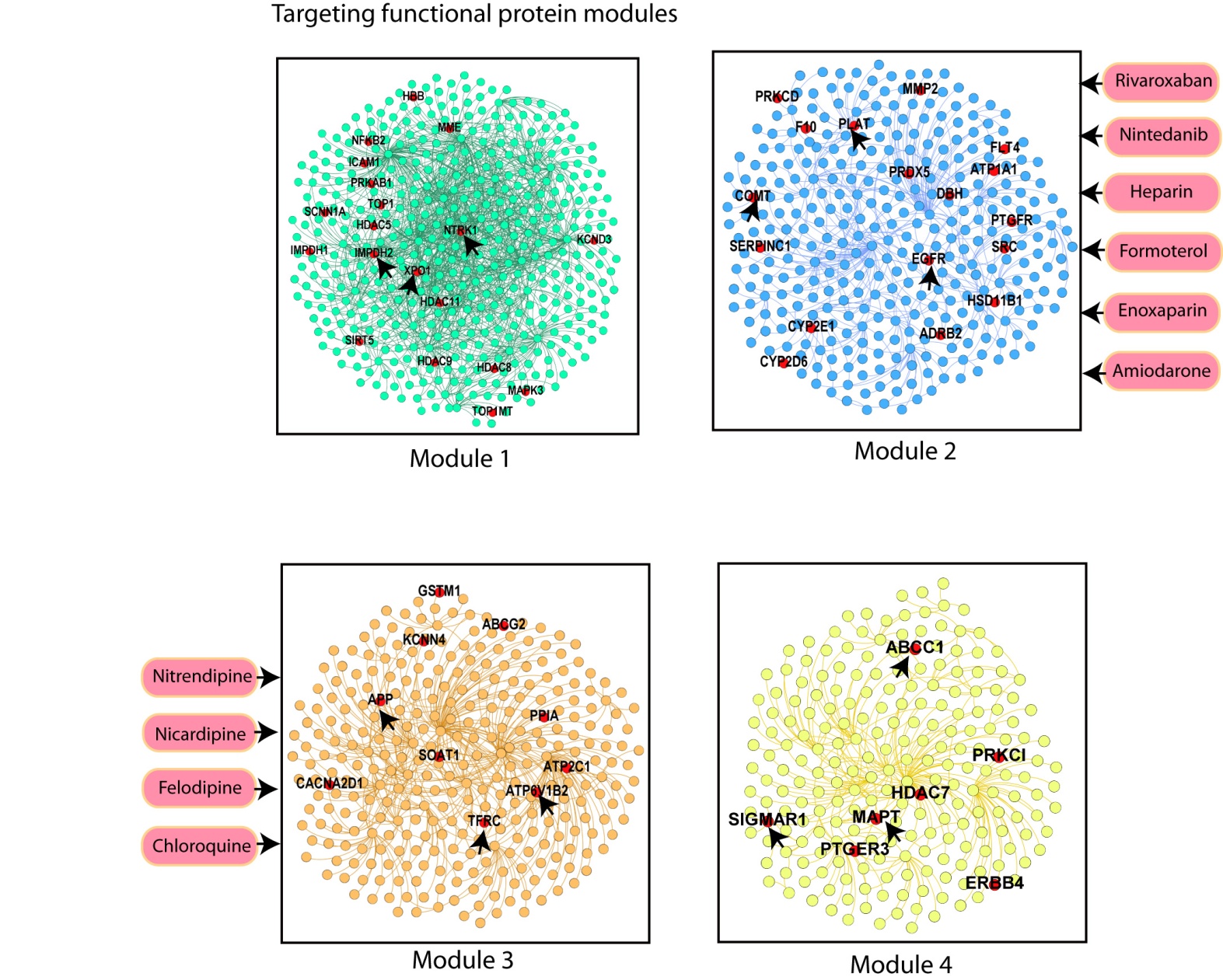
**

**Supplementary Fig. S5 (extended figure of Fig.5)** Drug repurposing to target functional protein modules: The red nodes in each module indicate the FDA-approved targets, and an arrow indicates the highly connected node. Drugs shown in the red box (extreme left and right) are in clinical trials for COVID19 treatment.
