# Supplementary material for: Lung disease network reveals impact of comorbidity on SARS-CoV-2 infection and opportunities of drug repurposing
Source: BMC Med Genomics. 2021 Sep 17;14:226. doi: 10.1186/s12920-021-01079-7 (PMC8447809; doi:10.1186/s12920-021-01079-7)
Supplement: Supplementary file 2 — Additional file 2. Table S1. Edge list of SARS-CoV-2 target network (STN) from TissueNet v.2 database. [file 12920_2021_1079_MOESM2_ESM.pdf]

Supplementary Table 1: Edge list of SARS-CoV-2 target network (STN) from TissueNet v.2 database

Interactor 1	Interactor 2
ZYG11B	PRSS50
ADAMTS1	PRSS50
PIGS	PRSS50
GPAA1	PRSS50
UBXN8	A1BG
ECSIT	A2M
FBXL12	A2M
ADAMTS1	A2M
AP2M1	A2M
ECD	AAR2
SLC7A6OS	AAR2
EFTUD2	AAR2
NCDN	AAR2
EAPP	AAR2
FKBP5	AAR2
KDR	AAR2
CUL3	AAR2
HECTD1	AARSD1
ABCC1	ABCC1
SLC9A3R1	ABCC4
RAB7A	ABCD1
RAB7A	ABCD3
ATP6V1A	ABCE1
GNB1	ABCE1
MDN1	ABCE1
PRKAR2A	ABCE1
ELOC	ABCE1
PRKACA	ABCE1
MFGE8	ABCE1
RALA	ABCE1
RPL36	ABCE1
PSMD8	ABCE1
CUL2	ABCF1
MOV10	ABCF3
RAB7A	ABCG2
UPF1	ABHD16A
RETREG3	ABHD16A
TLE5	ABI2
MOV10	ABR
CEP250	ABR
RAB5C	ABT1
RBX1	ABTB2
ATP5MG	ACAD9
ECSIT	ACAD9
NDUFAF1	ACAD9
ATP5PB	ACAD9
TIMMDC1	ACAD9
ABCF2	ACAD9
NDUFA8	ACAD9
ATP5F1B	ACAD9
ATP5F1C	ACAD9
CDC42	ACAD9
TMEM126B	ACAD9
RPL26L1	ACAD9
ATP5F1A	ACAD9
SIRT4	ACAD9
NDUFS5	ACAD9
FAF2	ACAD9
NDUFS7	ACAD9

MRPL58	ACAD9
DBT	ACAD9
FAS	ACAD9
ATP5F1D	ACAD9
NDUFS2	ACAD9
FOXRED1	ACAD9
ATP5PD	ACAD9
P2RY12	ACAD9
VDAC2	ACAD9
VDAC3	ACAD9
PDSS1	ACAD9
ACADM	ACADM
CUL3	ACADM
HDAC1	ACADM
FAF2	ACADM
GOLGA3	ACBD3
GOLGB1	ACBD3
CUL2	ACLY
MOV10	ACSF3
ACSL4	ACSL3
PPP6R2	ACSL3
NDRG1	ACSL3
MCM2	ACSL3
PPP6R1	ACSL3
CUL7	ACSL3
ESRRB	ACSL3
UPF1	ACSS2
FOXRED2	ACTA1
SUN2	ACTC1
MEPCE	ACTG1
RBX1	ACTG1
RIPK1	ACTG1
FOXRED2	ACTG2
CYB5R3	ACTL6A
RAB14	ACTL6A
SUN2	ACTN4
AKAP9	ACTR3B
MOV10	ADAL
ITGB1	ADAM17
KIF2C	ADAM9
SNX9	ADAM9
RNF2	ADAM9
TMEM25	ADAM9
SH3GL2	ADAM9
PTPRK	ADAM9
MAD2L2	ADAM9
PRKCD	ADAM9
CDH1	ADAM9
VEGFD	ADAM9
CEP250	ADAMTS1
VEGFA	ADAMTS1
NAPSA	ADAMTS1
HPX	ADAMTS1
EIF4E2	ADAMTSL4
UPF1	ADAR
GCC2	ADCYAP1
GGH	ADGRV1
CSNK2A2	ADH1A
PDE4DIP	ADH1B
MOV10	ADIPOR1
FYCO1	ADO

AP2M1	ADRA1B
GNB1	ADRA2A
FAM162A	ADRB2
STOM	ADRB2
UBXN8	ADRB2
CUL2	ADRB2
GNB1	ADRB2
ELOC	ADRB2
RAB18	ADRB2
SLC9A3R1	ADRB2
NUP210	ADRB2
TOMM70	ADRB2
PSMD8	ADRM1
TLE5	AEN
EXOSC8	AEN
PDE4DIP	AEN
BRD4	AFF1
MOV10	AGGF1
RAE1	AGGF1
MOV10	AGL
CUL2	AGL
PABPC1	AGO1
UPF1	AGO1
UPF1	AGO2
TRMT1	AGO2
PABPC1	AGO2
CUL2	AGO2
SUN2	AGO3
PABPC1	AGO4
PABPC4	AGO4
MOV10	AGPAT1
MOV10	AGRN
CLCC1	AGTR1
NUP88	AHCTF1
MOV10	AHCYL1
PLEKHF2	AIDA
STC2	AIFM1
CUL2	AIFM1
TOR1AIP1	AIFM1
SPART	AIFM1
CSNK2A2	AIM2
UPF1	AIM2
MOV10	AIM2
PRKAR2A	AKAP1
PRKACA	AKAP1
PRKAR2B	AKAP1
PRKAR2B	AKAP11
CEP250	AKAP12
RAB2A	AKAP12
TUBGCP3	AKAP12
PRKAR2A	AKAP12
RHOA	AKAP13
PRKAR2B	AKAP13
PRKAR2A	AKAP13
PRKAR2A	AKAP14
PRKAR2B	AKAP14
PRKACA	AKAP14
NINL	AKAP17A
PRKACA	AKAP5
PRKAR2B	AKAP5
PRKAR2A	AKAP5

PRKAR2B	AKAP7
PRKACA	AKAP7
CUL3	AKAP8
CDC5L	AKAP8
MCM2	AKAP8
FBXW11	AKAP8
ELAVL1	AKAP8
CSNK2A1	AKAP8
FOXP3	AKAP8
MATR3	AKAP8
ESR1	AKAP8
FOXJ2	AKAP8
TUBGCP3	AKAP9
PRKAR2B	AKAP9
PRKAR2A	AKAP9
PRKACA	AKAP9
THOC2	AKAP9
KDM1A	AKAP9
WAC	AKAP9
TACC3	AKAP9
CCNE1	AKAP9
GPC4	AKAP9
GSK3A	AKAP9
HUWE1	AKAP9
TRIP10	AKAP9
DYNLL1	AKAP9
PMS1	AKAP9
CDC5L	AKAP9
PCGF1	AKAP9
TDP2	AKAP9
HNRNPM	AKAP9
BLZF1	AKAP9
RNF2	AKAP9
MAPRE1	AKAP9
PKN1	AKAP9
KCNQ1	AKAP9
TULP3	AKAP9
TSG101	AKAP9
PRKACA	AKIP1
RALA	AKR7A2
DNMT1	AKT1
GNB1	AKT1
MARK2	AKT1
SLC9A3R1	AKT1
POFUT1	AKT2
POLA2	AKTIP
HOOK1	AKTIP
POLA1	ALDH1L2
MYCBP2	ALDH1L2
ERP44	ALDH7A1
CUL2	ALDOA
TREML2	ALG11
TSPAN5	ALG11
HAUS7	ALG11
MINDY2	ALG11
SMPD1	ALG11
UBE2G2	ALG11
CACNG4	ALG5
PKD2	ALG5
RPS6KA5	ALG8
SLC39A4	ALG8

MEPCE	ALYREF
CUL2	ALYREF
MARK3	ALYREF
CEP250	ALYREF
PSMD8	AMBRA1
ELOB	AMBRA1
CEP250	AMER1
AP2M1	AMER1
SELENOS	AMFR
USP13	AMFR
OS9	AMFR
CEP250	AMOT
NIN	AMOT
MOV10	AMOTL1
PRKAR2B	AMY1C
PMPCB	AMY1C
NINL	ANAPC2
PABPC1	ANAPC5
ITGB1	ANGPTL4
AP3B1	ANK3
GOLGA2	ANKRD11
CSNK2B	ANKRD28
AKAP8L	ANKRD28
AP2M1	ANKRD28
TBK1	ANKRD28
BRD4	ANKRD37
CUL2	ANKRD39
TUBGCP3	ANKRD40
MOV10	ANKRD49
POPDC2	ANO6
LGALS8	ANO6
ELAVL1	ANO6
FAM189A2	ANO6
WFS1	ANTXR1
FBXL12	ANXA2
ARF6	ANXA2
PLAT	ANXA2
CUL2	ANXA2
TUBGCP3	ANXA5
ATP6V1A	ANXA7
PLD3	ANXA7
DNAJC11	ANXA7
SUN2	AOPEP
AP2M1	AP1G1
AP2M1	AP1G2
HECTD1	AP1M2
PVR	AP1M2
RAB10	AP1S1
AP2M1	AP1S2
GGCX	AP1S2
TIMM29	AP1S2
AP2M1	AP2A1
AP2M1	AP2A2
MOV10	AP2A2
CLTB	AP2A2
MEX3C	AP2A2
ATP1A1	AP2A2
KIF11	AP2A2
TERF2	AP2A2
NUMB	AP2A2
GTSE1	AP2A2

CLTC	AP2A2
BIN1	AP2A2
PPP6R3	AP2A2
EPN1	AP2A2
AAGAB	AP2A2
PAN2	AP2A2
SMAD1	AP2A2
RPA2	AP2A2
IQGAP1	AP2A2
ZBTB46	AP2A2
EPS15	AP2A2
ESR1	AP2A2
GAK	AP2A2
YBX1	AP2A2
EGFR	AP2A2
VCP	AP2A2
RPA3	AP2A2
RNF126	AP2A2
ARRB2	AP2A2
SHC1	AP2A2
NECAP2	AP2A2
LIMA1	AP2A2
DBN1	AP2A2
MCM2	AP2A2
APP	AP2A2
AMPH	AP2A2
FN1	AP2A2
NXF1	AP2A2
DAB2	AP2A2
RPA1	AP2A2
GRB2	AP2A2
MARK2	AP2M1
RAB7A	AP2M1
AP2M1	AP2M1
UBC	AP2M1
DBN1	AP2M1
KRT17	AP2M1
GFAP	AP2M1
CLTC	AP2M1
AP1B1	AP2M1
AP2S1	AP2M1
CACNA1A	AP2M1
PEX14	AP2M1
MED4	AP2M1
STON2	AP2M1
EGFR	AP2M1
ITGA4	AP2M1
TUBA1B	AP2M1
TUBB2A	AP2M1
RRP12	AP2M1
CUL3	AP2M1
COLEC12	AP2M1
SPTBN1	AP2M1
ATG16L1	AP2M1
UTP25	AP2M1
ANLN	AP2M1
SFPQ	AP2M1
SLC25A4	AP2M1
CRY1	AP2M1
APC	AP2M1
KBTBD7	AP2M1

STK11	AP2M1
RNF11	AP2M1
SLC25A5	AP2M1
PPP6R2	AP2M1
PICALM	AP2M1
AAK1	AP2M1
STAU1	AP2M1
CDH3	AP2M1
HNRNPA2B1	AP2M1
RUNDC3A	AP2M1
DAB2	AP2M1
EPS15	AP2M1
TBC1D5	AP2M1
SNX29	AP2M1
NECAP2	AP2M1
EPHA2	AP2M1
GAPDH	AP2M1
SV2A	AP2M1
CD22	AP2M1
CORO1C	AP2M1
ANKRD52	AP2M1
IFNAR1	AP2M1
ABL1	AP2M1
CLEC4M	AP2M1
DVL2	AP2M1
TNKS2	AP2M1
FXR2	AP2M1
SYNJ1	AP2M1
GTSE1	AP2M1
CLINT1	AP2M1
IQGAP1	AP2M1
DDX6	AP2M1
ESR1	AP2M1
NKAP	AP2M1
AP2B1	AP2M1
RALBP1	AP2M1
SHC1	AP2M1
BMP2K	AP2M1
TGOLN2	AP2M1
EPS15L1	AP2M1
ACTB	AP2M1
XPO1	AP2M1
CHD8	AP2M1
MAP4	AP2M1
FAM199X	AP2M1
FN1	AP2M1
EPN1	AP2M1
AP1S1	AP2M1
LIMA1	AP2M1
FCHO2	AP2M1
PPHLN1	AP2M1
DCAF1	AP2M1
HSPA8	AP2M1
A1BG	AP2M1
EEF1A1	AP2M1
NDRG1	AP2M1
NECAP1	AP2M1
MYH9	AP2M1
LY9	AP2M1
MASP1	AP2M1
NUMB	AP2M1

RSPH14	AP2M1
CDH1	AP2M1
PRKACB	AP2M1
NAGPA	AP2M1
FURIN	AP2M1
RPS27A	AP2M1
GLP1R	AP2M1
EHD2	AP2M1
KCNQ1	AP2M1
AP1S3	AP2M1
MEA1	AP2M1
KIF13A	AP2M1
GULP1	AP2M1
ARRB2	AP2M1
CRY2	AP2M1
HSPA9	AP2M1
CALCOCO1	AP2M1
PEX5	AP2M1
REXO1	AP2M1
REPS1	AP2M1
AKAP11	AP2M1
DLG3	AP3B1
PTGER3	AP3B1
SEPTIN7	AP3B1
XPO1	AP3B1
AGO3	AP3B1
EPB41L3	AP3B1
ARF5	AP3B1
SNX11	AP3B1
CREB3	AP3B1
DTNBP1	AP3B1
KIF3A	AP3B1
SLC30A3	AP3B1
SNW1	AP3B1
VTI1B	AP3B1
AP3B1	AP3S2
ZNF318	AP4M1
IMPDH2	AP4M1
RALA	AP4M1
TIMM29	AP4M1
PABPC1	APBB1
RAP1GDS1	APBB2
NUP214	APC
MARK2	APC
PKP2	APC
HECTD1	APC
NUP98	APC
CEP250	APC
MOV10	APEH
NINL	APEX2
SCARB1	APOA1
MAP7D1	APOA1
SCARB1	APOA2
PRKACA	APOBEC3G
CUL2	APOD
ECSIT	APOE
FBXL12	APOE
EIF4E2	APP
NSD2	APP
TMEM97	APP
RPL36	APP

EXOSC3	APP
SIRT5	APP
POLA2	APP
RAB5C	APP
SPART	APP
BCKDK	APP
RAB10	APP
CEP250	APP
PRRC2B	APP
CYB5B	APP
CLIP4	APP
RAB14	APP
MAT2B	APP
ECSIT	APP
DCAF7	APP
MRPS2	APP
ACSL3	APP
ERC1	APP
RBX1	APP
UGGT2	APP
TLE3	APP
SPART	APPL1
MYCBP2	APPL1
RAB5C	APPL2
CEP350	APTX
AP2M1	AQP4
MDN1	AR
TLE5	AR
SCAP	AR
NSD2	AR
IDE	AR
CUL2	AR
RALA	ARF1
RALA	ARF6
RAB1A	ARF6
PABPC4	ARF6
EMC1	ARF6
HYOU1	ARF6
PSMD8	ARF6
MEPCE	ARF6
AP3B1	ARF6
AAR2	ARF6
PLD1	ARF6
PGD	ARF6
SF3B1	ARF6
ACLY	ARF6
RAB11FIP3	ARF6
GGA3	ARF6
SEC11A	ARF6
TGOLN2	ARF6
APP	ARF6
GOT2	ARF6
RAB11FIP5	ARF6
ARRB1	ARF6
SEC13	ARF6
TARDBP	ARF6
KHSRP	ARF6
GLS	ARF6
SNRPA1	ARF6
ARFIP2	ARF6
IMMT	ARF6

PFKP	ARF6
MTHFD1	ARF6
RGS10	ARF6
SF3A1	ARF6
EIF3I	ARF6
ARMT1	ARF6
POLD1	ARF6
EEF1E1	ARF6
ECPAS	ARF6
PLS1	ARF6
DNAAF5	ARF6
GSPT1	ARF6
RPN1	ARF6
PFDN2	ARF6
KIF23	ARF6
SRP14	ARF6
USO1	ARF6
APEX1	ARF6
CYTH1	ARF6
CYTH2	ARF6
UQCRC2	ARF6
SOD2	ARF6
CS	ARF6
GDI2	ARF6
IPO11	ARF6
NCAPG	ARF6
HNRNPM	ARF6
IQGAP1	ARF6
NASP	ARF6
FN1	ARF6
AIMP1	ARF6
EXOC5	ARF6
CHRM3	ARF6
TRIM28	ARF6
BTF3	ARF6
MSH2	ARF6
VCP	ARF6
COPS4	ARF6
NUP93	ARF6
XPO5	ARF6
HSPA4L	ARF6
RABGAP1	ARF6
RTCA	ARF6
ASAP1	ARF6
ARRB2	ARF6
EIF3E	ARF6
DDX19B	ARF6
GANAB	ARF6
RAB3GAP2	ARF6
UBE2D3	ARF6
TRIM23	ARF6
DHPS	ARF6
TARS1	ARF6
GCLM	ARF6
EIF3G	ARF6
SRPRB	ARF6
SMC2	ARF6
HDLBP	ARF6
NUDC	ARF6
OAT	ARF6
CCDC47	ARF6

SERBP1	ARF6
EGFR	ARF6
STAU1	ARF6
NCLN	ARF6
MCM3	ARF6
GGA1	ARF6
DHX9	ARF6
USP6	ARF6
LRPPRC	ARF6
YARS1	ARF6
VHL	ARF6
CACYBP	ARF6
EIF4G1	ARF6
RPN2	ARF6
ATP6V1C1	ARF6
GART	ARF6
AP3S2	ARF6
DDX5	ARF6
NPEPPS	ARF6
GNAQ	ARF6
RAB11FIP4	ARF6
PRKAR2A	ARFGEF2
RHOA	ARHGAP1
TLE5	ARHGAP32
RHOA	ARHGAP35
CSNK2A2	ARHGAP39
RHOA	ARHGAP5
RDX	ARHGDIA
RHOA	ARHGDIA
RHOA	ARHGDIG
RHOA	ARHGEF11
RHOA	ARHGEF12
MARK2	ARHGEF2
RHOA	ARHGEF2
RHOA	ARHGEF25
RHOA	ARHGEF3
RHOA	ARHGEF4
STC2	ARHGEF40
PLEKHA5	ARHGEF9
CUL2	ARID1A
MEPCE	ARID3B
GOLGA2	ARID5A
EIF4E2	ARIH1
CUL2	ARIH1
ELOC	ARIH2
ELOB	ARIH2
RAB18	ARL13B
GRIPAP1	ARL15
GOLGA2	ARL16
MOV10	ARL2
RETREG3	ARL6IP1
RTN4	ARL6IP1
RNF41	ARL6IP4
CSNK2A2	ARL6IP4
RETREG3	ARL6IP5
RTN4	ARL6IP5
ARL6	ARL6IP6
BLVRB	ARL6IP6
PNPO	ARL6IP6
GSTM1	ARL6IP6
NAA50	ARL6IP6

YKT6	ARL6IP6
PYGB	ARL6IP6
CBFB	ARL6IP6
DUSP3	ARL6IP6
UBE2D2	ARL6IP6
PMVK	ARL6IP6
APP	ARL6IP6
SNX3	ARL6IP6
DNAJC11	ARMC1
RAE1	ARMC8
CUL2	ARNT
ELOB	ARNT
GOLGA2	ARNT2
MIPOL1	ARNT2
MOV10	ARPC4
AP3B1	ARRB1
GNB1	ARRB1
PABPC4	ARRB2
PABPC1	ARRB2
CENPF	ARRB2
RPL36	ARRB2
RAB5C	ARRB2
STC2	ARRB2
G3BP2	ARRB2
AP3B1	ARRB2
MDN1	ART3
MARK3	ASB13
ELOB	ASB13
ELOC	ASB13
MOV10	ASB13
ELOC	ASB14
ELOB	ASB14
ELOC	ASB16
ELOB	ASB16
ELOB	ASB3
ELOB	ASB6
ELOB	ASB7
ELOC	ASB7
MOV10	ASB8
ELOC	ASB8
ELOB	ASB8
CSNK2A2	ASCC1
POLA2	ASCC2
ERLEC1	ASGR2
OS9	ASGR2
STOM	ASIC3
HMOX1	ASL
CSNK2A2	ASL
CUL2	ASPRV1
RAB2A	ASS1
FBXL12	ASTN2
EXOSC2	ASXL2
TUBGCP2	ATAD3A
NDUFAF1	ATAD3B
ECSIT	ATAD3B
BRD4	ATAD5
RAE1	ATE1
MAPK8	ATE1
SUV39H1	ATE1
CNN2	ATE1
GRPEL1	ATF2

RAB2A	ATF2
TIMM9	ATF2
RAB7A	ATF2
RTN4	ATF2
WFS1	ATF6
ERLEC1	ATF6
ERLEC1	ATF6B
RAB2A	ATF6B
MOV10	ATF6B
RPL36	ATG12
SUN2	ATG12
AGPS	ATG16L1
MOV10	ATG4B
TBK1	ATG9A
NDUFAF1	ATIC
RTN4	ATL1
RAB14	ATL3
RTN4	ATL3
ITGB1	ATL3
RAB1A	ATL3
RAB5C	ATL3
TLE5	ATN1
PLEKHA5	ATN1
FAM8A1	ATP13A2
MYCBP2	ATP13A2
DCAF7	ATP13A2
ISLR	ATP13A3
LGALS8	ATP13A3
LGALS3	ATP13A3
PPT1	ATP1A1
ATP1B1	ATP1A1
ELAVL1	ATP1B1
GCH1	ATP1B1
NEDD4L	ATP1B1
BRCA1	ATP1B1
BARD1	ATP1B1
LRIF1	ATP1B1
RIF1	ATP1B1
SLC4A1	ATP1B1
EZH2	ATP1B1
TRMT2A	ATP1B1
PSME1	ATP1B1
USP4	ATP1B1
RAB7A	ATP2A2
GGH	ATP2B2
UBXN8	ATP2B2
PPT1	ATP5F1A
POLA2	ATP5F1C
PIGS	ATP5MD
REEP5	ATP5MD
CDK2	ATP5MG
RHOU	ATP5MG
SAP18	ATP5MG
ATP5PB	ATP5MG
EPB41	ATP5MG
GABARAPL1	ATP5MG
TSC22D1	ATP5MG
BLK	ATP5MG
EGFR	ATP5MG
SLC38A1	ATP5MG
RALA	ATP5PF

PPT1	ATP5PO
MIPOL1	ATP5PO
ACAD9	ATP5PO
RHOA	ATP6AP2
RAB7A	ATP6AP2
ATP6V1A	ATP6AP2
RALA	ATP6AP2
CHMP2A	ATP6AP2
RAB14	ATP6AP2
ATP6AP1	ATP6AP2
REEP5	ATP6V0A2
RAB5C	ATP6V0D1
RAB7A	ATP6V0D1
RAB2A	ATP6V0D1
GNB1	ATP6V1A
RAB5C	ATP6V1A
CHMP4B	ATP6V1A
VTI1B	ATP6V1A
CUL3	ATP6V1A
DBN1	ATP6V1A
CUL7	ATP6V1A
ELAVL1	ATP6V1A
ATP6V0A1	ATP6V1A
CD9	ATP6V1A
CHMP2B	ATP6V1A
LAMP2	ATP6V1A
RAB35	ATP6V1A
TFRC	ATP6V1A
ATP6V1D	ATP6V1A
GOLT1B	ATP6V1A
CHMP5	ATP6V1A
ATP6V1H	ATP6V1A
DNAJC5	ATP6V1A
VAPA	ATP6V1A
GNB1	ATP6V1B1
ATP6V1A	ATP6V1B1
RAB2A	ATP6V1B2
RAB5C	ATP6V1B2
ATP6V1A	ATP6V1B2
RAB7A	ATP6V1B2
ATP6V1A	ATP6V1C1
HECTD1	ATP6V1C2
ECSIT	ATP6V1C2
ATP6V1A	ATP6V1C2
ATP6V1A	ATP6V1E1
ATP6V1A	ATP6V1G1
RAB7A	ATP6V1G1
SNIP1	ATR
PLEKHA5	ATXN1
GNB1	ATXN10
TLE5	ATXN1L
PRRC2B	ATXN1L
USP54	ATXN1L
AP2M1	ATXN1L
PABPC1	ATXN2
MOV10	ATXN2
G3BP1	ATXN2L
G3BP2	ATXN2L
AP2M1	ATXN2L
USP13	ATXN3
GOLGA3	ATXN7

RAB10	AURKA
MOGS	AURKB
MRPS27	AURKB
PLEKHA5	AURKB
MARK2	AURKB
TLE3	AUTS2
DCAF7	AUTS2
CSNK2A2	AUTS2
MOV10	AVEN
GNB1	AVPR2
ABCC1	AZGP1
MIB1	AZI2
ZYG11B	AZU1
SLC30A6	B3GNT3
POGLUT2	B3GNT3
GHITM	B3GNT3
SLC30A7	B3GNT3
FAM8A1	B4GALT3
UGGT2	BABAM1
PRKAR2B	BABAM1
UGGT2	BABAM2
MOV10	BACE1
MOV10	BACH1
UBAP2L	BAG3
PSMD8	BAG3
TIMM9	BAG3
MAT2B	BAG3
RAB7A	BAG3
RAB1A	BAG3
TIMM10	BAG3
NEK9	BAG3
PABPC1	BAG3
CUL2	BAG4
MOV10	BAG4
SNCA	BAG5
THAP1	BAG5
STUB1	BAG5
P2RX6	BAG5
AGO3	BAG5
UIMC1	BAG5
FBXO25	BAG5
GBA2	BAG5
DNAJC13	BAG5
NDRG2	BAG5
CUL3	BAG5
HSF2	BAG5
HSPA8	BAG5
FBXW11	BAG5
POLR2F	BAG5
OTUD4	BAG5
MAD1L1	BAG5
AMBRA1	BAG5
TP53	BAG5
LLGL1	BAG5
ALDH3B1	BAG5
DLG5	BAG5
MAP3K1	BAG5
LLGL2	BAG5
MMS19	BAG5
LRRK1	BAG5
CIRBP	BAG5

ABL2	BAG5
MAD2L1	BAG5
RAB8A	BAG6
CNTRL	BAG6
USP13	BAG6
PRIM2	BAG6
CSNK2B	BAG6
SIRT5	BAG6
GTF2F2	BAG6
TLE5	BAHD1
RAP1GDS1	BAIAP2
RTN4	BAIAP2
BRD4	BAIAP2
RHOA	BAIAP2
MYCBP2	BAK1
ATP6V1A	BANF1
BAG5	BANP
AKAP8L	BARD1
DCAF7	BARD1
AKAP8	BARD1
NPC2	BARD1
EXOSC2	BARD1
RHOA	BAZ1A
RAB5C	BBX
MOV10	BCAP31
PABPC1	BCAR3
CSNK2A2	BCAS2
CAB39L	BCKDK
SLC9A3R1	BCL10
RTN4	BCL2
BRD4	BCL2
PLD3	BCL2L1
LARP1	BCL2L1
RTN4	BCL2L1
ELOB	BCL2L11
CUL2	BCL2L11
TLE5	BCL6
CSNK2A2	BCL7B
ELOC	BCOR
DNAJC11	BCOR
RHOA	BCR
CSNK2A2	BCR
AP2M1	BCR
MDN1	BECN1
TLE5	BHLHE40
CSNK2A2	BHLHE40
CSNK2A2	BHLHE41
NEK9	BICD2
RAB5C	BICRAL
RHOA	BIRC2
EXOSC5	BIRC2
MIB1	BLM
PLEKHA5	BLOC1S6
RAB2A	BLZF1
TCF12	BMERB1
AKAP9	BMI1
G3BP1	BMI1
UPF1	BMI1
PABPC4	BMI1
UBAP2L	BMI1
ZC3H7A	BMI1

AKAP8	BMI1
MEPCE	BMI1
VPS11	BMI1
G3BP2	BMI1
PKP2	BMI1
AKAP8L	BMI1
DNMT1	BMPR1B
GFER	BNIPL
NGDN	BORA
NIN	BORCS6
CEP250	BORCS6
FYCO1	BORCS6
CDK5RAP2	BORCS6
CEP135	BORCS6
GOLGB1	BORCS6
BRD2	BRD2
CSNK2A2	BRD2
BRD4	BRD2
RUNX3	BRD2
CSNK2A1	BRD2
NSD3	BRD2
E2F1	BRD2
ELAVL1	BRD2
BRPF1	BRD2
OBSL1	BRD2
SF1	BRD2
IL7R	BRD2
MYC	BRD2
TBP	BRD2
FARP1	BRD2
CDC5L	BRD2
BAG1	BRD2
PWWP2B	BRD2
FAM90A1	BRD2
BCL2	BRD2
E2F2	BRD2
BRD7	BRD2
COX5A	BRD2
POLR2B	BRD2
H2BC21	BRD2
DENND2D	BRD2
TERF2	BRD2
EPB41L1	BRD2
TAGLN2	BRD2
TADA3	BRD2
BRD4	BRD3
NSD2	BRD4
SIRT5	BRD4
STC2	BRD4
CKM	BRD4
AK1	BRD4
KDM5B	BRD4
CHD4	BRD4
MYC	BRD4
MESD	BRD4
CDC5L	BRD4
CHFR	BRD4
TCERG1	BRD4
BICRA	BRD4
SMC1A	BRD4
RFC1	BRD4

SMARCA4	BRD4
CDK9	BRD4
MLLT1	BRD4
ELAVL1	BRD4
MED24	BRD4
RBM25	BRD4
CDK8	BRD4
SIRT1	BRD4
EP300	BRD4
RFC5	BRD4
TWIST1	BRD4
MLF2	BRD4
WNT5A	BRD4
SELE	BRD4
CCNT2	BRD4
BRD9	BRD4
RFC3	BRD4
MED17	BRD4
BAIAP2L1	BRD4
HS1BP3	BRD4
CCNT1	BRD4
CYP1A1	BRD4
RFC2	BRD4
JMJD6	BRD4
MED1	BRD4
REEP5	BRD7
CSNK2A2	BRF1
GOLGB1	BRMS1
FAM162A	BSG
OS9	BSG
MDN1	BSG
NLRX1	BSG
CSNK2A2	BTF3
CUL2	BTG3
SCARB1	BTNL8
CHPF	BTRC
CEP68	BTRC
TUBGCP2	BTRC
MOV10	BTRC
SUN2	BTRC
RBX1	BTRC
PRKACA	BTRC
TUBGCP3	BTRC
MRPS27	BTRC
RAE1	BUB1
AP3B1	BUB1
AP3B1	BUB1B
RAE1	BUB1B
TLE5	BYSL
HACD3	BZW2
EIF2B1	BZW2
ELAVL1	BZW2
STX12	BZW2
CD68	BZW2
BZW1	BZW2
LYPD3	BZW2
GSK3B	BZW2
EIF3A	BZW2
FN1	BZW2
MARK2	C11orf58
EIF4H	C11orf68

CSDE1	C11orf68
SLC30A6	C12orf43
CSNK2A2	C18orf25
EXOSC5	C1D
EXOSC3	C1D
EXOSC8	C1D
TLE5	C1orf109
TLE5	C1orf216
C1orf50	C1orf50
APPBP2	C1orf50
AHCY	C1orf50
WDYHV1	C1orf50
CUL2	C1QBP
RAB5C	C2CD5
PRKACA	C2orf88
ERMP1	C4orf3
CSNK2B	C6orf136
MOV10	C8orf33
BRD4	C8orf33
ETFA	C8orf82
HECTD1	C9orf24
ERMP1	CA14
BCKDK	CAB39
CUL2	CAB39
PCSK6	CACNA1A
USP13	CACYBP
EXOSC5	CALCOCO2
PTBP2	CALCOCO2
TLE5	CALCOCO2
ACADM	CALM1
PCNT	CALM1
RDX	CALM1
MOV10	CALM1
TBK1	CALM1
RALA	CALM1
MYCBP2	CALM1
AKAP9	CALM1
DDX21	CALM1
AKAP9	CALM2
MOV10	CALM3
AKAP9	CALM3
PLAT	CALR
CEP250	CALU
MARK2	CAMK1
HECTD1	CAMKMT
MARK2	CAMSAP2
CSNK2A2	CAMSAP2
RBX1	CAND1
CUL2	CAND1
AKAP8	CAND1
RBM28	CAND1
UPF1	CAND1
SCARB1	CAND1
PABPC1	CAND1
AKAP8L	CAND1
NUP88	CAND1
PABPC4	CAND1
MOV10	CANT1
FOXRED2	CANX
DNAJC11	CAPG
POLA2	CAPN1

FBXL12	CAPN2
TUBGCP3	CAPNS1
MARK2	CAPZA1
MARK3	CAPZA1
RAB1A	CAPZA2
MARK2	CAPZA2
WASHC4	CAPZA2
AP2M1	CAPZA2
MARK2	CAPZB
MARK3	CAPZB
CDK5RAP2	CARD9
CEP135	CARD9
EIF4E2	CARD9
TLE5	CARD9
STC2	CARHSP1
CUL2	CARM1
UPF1	CASC3
FOXRED2	CASK
RBX1	CASP3
MDN1	CASP4
NIN	CASQ2
MYCBP2	CASQ2
MARK3	CASQ2
RAB7A	CAV1
PRKACA	CAV1
RAB2A	CAV1
ABCC1	CAV1
RHOA	CAV1
RAB7A	CAVIN1
RAB2A	CAVIN1
RAB5C	CAVIN1
RAB5C	CAVIN3
RAB7A	CAVIN3
PRKAR2A	CBFA2T3
FBXL12	CBWD3
NINL	CBX1
PRKACA	CBX1
MYCBP2	CBX2
CSNK2A2	CBX2
ATE1	CBX3
PRKACA	CBX3
MYCBP2	CBX4
CSNK2A2	CBX4
POLA1	CBX4
DNMT1	CBX6
CEP135	CBX6
MOV10	CBX6
CEP350	CBX6
MEPCE	CCAR2
MARK1	CCDC102B
RAB7A	CCDC115
CEP135	CCDC13
CEP350	CCDC13
PCNT	CCDC13
CDK5RAP2	CCDC13
NINL	CCDC130
MIB1	CCDC14
CEP135	CCDC14
NINL	CCDC146
MYCBP2	CCDC158
PCNT	CCDC183

GRIPAP1	CCDC183
PLEKHA5	CCDC186
NINL	CCDC33
RIPK1	CCDC50
MOV10	CCDC51
TLE5	CCDC57
CSNK2A2	CCDC59
NIN	CCDC68
AKAP9	CCDC68
FYCO1	CCDC68
CENPF	CCDC68
PSMD8	CCDC74B
PRKAR2B	CCDC77
PABPC1	CCDC8
CEP250	CCDC8
RALA	CCDC8
STOML2	CCDC8
MDN1	CCDC8
ITGB1	CCDC8
ATP1B1	CCDC8
RAB1A	CCDC8
G3BP1	CCDC8
TOR1AIP1	CCDC8
PCNT	CCDC8
ETFA	CCDC8
MARK2	CCDC8
ACSL3	CCDC8
PABPC4	CCDC8
RAB14	CCDC8
AKAP8	CCDC8
GNB1	CCDC8
LMAN2	CCDC8
AGPS	CCDC8
MYCBP2	CCDC8
RAB5C	CCDC8
DDX21	CCDC8
CEP350	CCDC8
MARK2	CCDC82
G3BP1	CCDC84
NUP54	CCDC85B
SLU7	CCDC85B
RBM41	CCDC85B
PRR11	CCDC86
FBL	CCDC86
PRKACA	CCDC88A
PLEKHA5	CCDC88B
PSMD8	CCDC92
PLEKHA5	CCDC92
GOLGA2	CCHCR1
NINL	CCHCR1
CSNK2A2	CCHCR1
LARP4B	CCNA1
RBX1	CCNA2
POLA1	CCNA2
AKAP8	CCND1
RBX1	CCND1
AKAP8	CCND2
RBX1	CCND2
AKAP8	CCND3
RBX1	CCND3
POLA1	CCNE1

RBX1	CCNF
TLE5	CCNJL
STC2	CCNJL
CSNK2A2	CCNL1
CSNK2A2	CCNL2
GRIPAP1	CCNT1
MOV10	CCNYL1
FYCO1	CCT2
MEPCE	CCT2
DCAF7	CCT2
MIPOL1	CCT3
DCAF7	CCT3
GNB1	CCT5
DCAF7	CCT5
DCAF7	CCT6A
DCAF7	CCT8
MEPCE	CCT8
ITGB1	CD151
PVR	CD226
COQ8B	CD27
LOX	CD2AP
MOV10	CD2BP2
TM2D3	CD3E
ERP44	CD40
ITGB1	CD47
RETREG3	CD47
ATP6V1A	CD63
PABPC1	CD81
PVR	CD96
DNAJC11	CD99L2
NINL	CDC20
MARK3	CDC25C
UPF1	CDC37
RAE1	CDC37
MOV10	CDC40
SNIP1	CDC40
DDX21	CDC42BPB
RIPK1	CDC42BPB
MOV10	CDC42SE1
MARK2	CDC5L
CSNK2A2	CDC5L
UPF1	CDC5L
AKAP8L	CDC6
ELOB	CDC73
SLC9A3R1	CDCA5
CENPF	CDCA5
POLA1	CDCA7L
TIMM29	CDK1
CSNK2A2	CDK11B
MEPCE	CDK11B
AP2M1	CDK11B
MARK2	CDK15
CSNK2A2	CDK16
LARP4B	CDK18
ERC1	CDK19
PSMD8	CDK2
UPF1	CDK2
POLA1	CDK2
CYB5R3	CDK2
GORASP1	CDK2
PABPC1	CDK2

RAB5C	CDK2
ERC1	CDK20
POLA1	CDK2AP1
GFER	CDK4
HOOK1	CDK4
CDK5RAP2	CDK5
CDK5RAP2	CDK5R1
EXOSC5	CDK5RAP1
CLIP4	CDK5RAP2
CEP68	CDK5RAP2
AKAP9	CDK5RAP2
PRKAR2B	CDK5RAP2
TUBGCP3	CDK5RAP2
TUBGCP2	CDK5RAP2
PRKACA	CDK5RAP2
TSNAX	CDK5RAP2
TUBG1	CDK5RAP2
MAPRE1	CDK5RAP2
RBM48	CDK5RAP2
DTNBP1	CDK5RAP2
ABCA2	CDK5RAP2
DYNLL1	CDK5RAP2
FGL2	CDK5RAP2
RPGRIP1L	CDK5RAP2
TBC1D4	CDK5RAP2
CHMP4B	CDK5RAP2
ERG28	CDK5RAP2
CRMP1	CDK5RAP2
PTN	CDK5RAP2
CEP152	CDK5RAP2
ZPR1	CDK5RAP2
TRIM37	CDK5RAP2
TUBGCP6	CDK5RAP2
STC2	CDK7
STC2	CDK8
STC2	CDK9
MRPS27	CDK9
EXOSC5	CDK9
ATP6V1A	CDKN1A
MOGS	CDKN1A
MARK2	CDKN1A
RHOA	CDKN1B
FBXL12	CDKN1C
CUL2	CDKN2A
EIF4E2	CDR2
MOV10	CDYL2
HDAC2	CEBPA
AKAP9	CEBPA
MRPS27	CENPA
CENPF	CENPE
CENPF	CENPF
TSC22D1	CENPF
CHMP2B	CENPF
CUL7	CENPF
NFKBIA	CENPF
NDE1	CENPF
NUP107	CENPF
TOP3B	CENPF
NUP133	CENPF
EMC1	CENPH
CRTC3	CENPJ

GOLGA2	CENPP
CENPF	CENPU
GOLGA3	CENPU
FUS	CEP112
MIB1	CEP131
PRKAR2B	CEP131
GOLGB1	CEP135
MIB1	CEP135
ERC1	CEP135
SREK1	CEP135
CEP72	CEP135
FOPNL	CEP135
SIPA1L3	CEP135
AIMP1	CEP135
CEP128	CEP135
TBC1D31	CEP135
SPICE1	CEP135
WDR90	CEP135
PLD1	CEP135
SMAD9	CEP135
CCDC77	CEP135
TTLL5	CEP135
CEP162	CEP135
PIBF1	CEP135
CEP295	CEP135
CCDC18	CEP135
CCDC61	CEP135
OFD1	CEP135
SSX2IP	CEP135
KIAA1328	CEP135
WRAP73	CEP135
ELMO2	CEP135
PCM1	CEP135
CEP131	CEP135
MAPRE1	CEP135
SREK1IP1	CEP135
ZMAT2	CEP135
LZTS2	CEP135
SPATA5	CEP135
CSPP1	CEP135
NUP210	CEP162
MIB1	CEP162
CEP250	CEP170
NIN	CEP170
TUBGCP3	CEP170
AKAP9	CEP170
PRKAR2B	CEP170
PRKAR2A	CEP170
PKP2	CEP250
NINL	CEP250
GOLGA3	CEP250
PABPC1	CEP250
MRPS2	CEP250
PLEKHA5	CEP250
MIB1	CEP250
MOGS	CEP250
FBL	CEP250
YWHAE	CEP250
MBD3	CEP250
RPLP0	CEP250
GALNT18	CEP250

CLSPN	CEP250
MAGED2	CEP250
TRIM32	CEP250
HDAC5	CEP250
F7	CEP250
TUBA1B	CEP250
PRPF3	CEP250
FAM120A	CEP250
HSP90AA1	CEP250
PRKAR1A	CEP250
MYH9	CEP250
RPL18	CEP250
TNRC6A	CEP250
SRSF3	CEP250
N4BP2	CEP250
SSX2IP	CEP250
HSP90AB1	CEP250
RPS13	CEP250
LZTS2	CEP250
HNRNPR	CEP250
LGALS3BP	CEP250
CUL7	CEP250
RCN2	CEP250
ARHGEF2	CEP250
DDX5	CEP250
YBX3	CEP250
RCN1	CEP250
RPL18A	CEP250
RPS19	CEP250
ENO1	CEP250
PIAS1	CEP250
DDX17	CEP250
RPL19	CEP250
NDE1	CEP250
HNRNPM	CEP250
MATR3	CEP250
SNRPD3	CEP250
VASP	CEP250
NEK2	CEP250
RPL5	CEP250
HSPA9	CEP250
SRSF7	CEP250
PPFIBP1	CEP250
TUBB4A	CEP250
PRPF19	CEP250
NOP2	CEP250
HOOK2	CEP250
RPS12	CEP250
RPL3	CEP250
RPS16	CEP250
KAT2B	CEP250
FTL	CEP250
CCDC88A	CEP250
HNRNPC	CEP250
MTMR1	CEP250
ZKSCAN1	CEP250
SIPA1L2	CEP250
RPL24	CEP250
RPL31	CEP250
LAS1L	CEP250
HSPA8	CEP250

MRPS14	CEP250
PARD3B	CEP250
ANKIB1	CEP250
ZC3HAV1	CEP250
ESS2	CEP250
UBR5	CEP250
TRIM37	CEP250
GTF3C3	CEP250
AGO2	CEP250
DDX24	CEP250
MRPS35	CEP250
CLINT1	CEP250
RPS10	CEP250
MRPS34	CEP250
HUWE1	CEP250
PCM1	CEP250
DHX15	CEP250
RPL21	CEP250
SUPT6H	CEP250
RPL6	CEP250
RPS25	CEP250
RPL23	CEP250
ACTB	CEP250
HSPA5	CEP250
DOCK7	CEP250
PCNT	CEP290
CEP135	CEP290
MIB1	CEP290
PLEKHA5	CEP350
CEP250	CEP350
PPP2CB	CEP350
XPO1	CEP350
KIF22	CEP350
BRCA1	CEP350
MAPK1	CEP350
MAPRE1	CEP350
PPARG	CEP350
PPP2R1A	CEP350
KIFAP3	CEP350
PPP2CA	CEP350
CYLD	CEP350
PPP2R3C	CEP350
NR1H3	CEP350
PCM1	CEP350
OFD1	CEP350
PPARD	CEP350
HIF1A	CEP350
ECSIT	CEP55
RALA	CEP55
TUBGCP3	CEP55
MOGS	CEP57
MARK2	CEP57
FBLN5	CEP57
DCAF7	CEP57
GOLGA2	CEP57L1
PRKAR2B	CEP68
PRKAR2B	CEP72
CEP250	CEP78
MARK2	CEP85L
MOV10	CEP95
CSNK2A2	CEPT1

GGH	CERCAM
OS9	CERCAM
RETREG3	CERS6
MARK2	CERT1
EXOSC3	CERT1
BCKDK	CETN2
BCKDK	CETN3
CUL2	CFAP300
CSNK2A2	CFAP97
CRTC3	CFAP97
RAB7A	CFL1
PABPC1	CHAF1A
RAB2A	CHCHD3
RAB7A	CHCHD3
DNAJC11	CHCHD3
TOR1AIP1	CHCHD4
RAB7A	CHCHD6
PCNT	CHD3
MOV10	CHD3
DNMT1	CHEK1
POLA1	CHEK1
NEK9	CHEK1
CSNK2A2	CHEK2
MARK3	CHGB
POLA2	CHM
ATE1	CHM
NUP98	CHM
NUP88	CHM
POLA1	CHM
RAB1A	CHM
PRIM2	CHM
NUP214	CHM
MYCBP2	CHM
RAB7A	CHM
RAB1A	CHML
RAB7A	CHMP1A
ATP6V1A	CHMP1A
RAB5C	CHMP1A
ATP6V1A	CHMP1B
USP54	CHMP1B
CHMP2A	CHMP2A
RAB5C	CHMP2A
RAB2A	CHMP2A
RAB7A	CHMP2A
VTA1	CHMP2A
CHMP4B	CHMP2A
TNPO3	CHMP2A
TGFB3	CHMP2A
ITGA4	CHMP2A
CHMP3	CHMP2A
TRPC4AP	CHMP2A
RAB11A	CHMP2A
STAMBP	CHMP2A
TTC19	CHMP2A
UFD1	CHMP2A
XPO1	CHMP2A
TFG	CHMP2A
VPS4B	CHMP2A
DOCK8	CHMP2A
RAB7A	CHMP2B
ATP6V1A	CHMP3

USP54	CHMP4A
RAB7A	CHMP4B
USP54	CHMP6
AGPS	CHORDC1
MOV10	CHP1
ARHGEF5	CHPF
FBXO6	CHPF
SMAD9	CHPF
NDUFAF2	CHST15
ERC1	CHST15
MOV10	CHTF8
MOV10	CHTOP
ERC1	CHUK
POLA2	CIAO1
AKAP8L	CIAO1
PLEKHA5	CIAO1
POLA1	CIAO1
GOLGA3	CIAO1
PRIM2	CIAO2B
BAG5	CIAO2B
MOV10	CIPC
ELOB	CISH
CUL2	CISH
RHOA	CIT
CUL7	CIT
ELAVL1	CIT
RND3	CIT
ANLN	CIT
EZH2	CIT
SELENOS	CKAP4
RAB5C	CKAP4
RAB7A	CKAP4
MEPCE	CKAP5
MOV10	CKB
MIPOL1	CKS1B
MARK2	CLASP1
MOV10	CLASP2
MARK2	CLASP2
GABRE	CLCC1
CUL3	CLCC1
TMEM30A	CLCC1
UBR2	CLCC1
SLC9A3R1	CLCN3
BRD4	CLDN1
ATE1	CLDN10
MYCBP2	CLEC11A
AKAP8	CLEC11A
NINL	CLEC1B
PLAT	CLEC3B
NUP214	CLEC4G
REEP6	CLEC5A
ECSIT	CLEC5A
YIF1A	CLEC5A
REEP5	CLEC5A
MOV10	CLHC1
ARF6	CLIC1
AKAP9	CLIC1
RAB5C	CLINT1
CLIP4	CLIP1
TULP3	CLIP4
LZTS2	CLIP4

TNNT1	CLIP4
KIF1B	CLIP4
MYH3	CLIP4
PKP2	CLK2
SNIP1	CLK2
PABPC1	CLK2
MOV10	CLK3
PABPC1	CLK3
PABPC4	CLK3
LARP1	CLK3
SNIP1	CLK3
SIGMAR1	CLN3
ATP5MG	CLN3
ERLEC1	CLN5
CYB5B	CLN8
RTN4	CLN8
AP2M1	CLTB
SPART	CLTC
PLEKHA5	CLTC
PRKAR2A	CLTC
CRTC3	CLTC
PRKACA	CLTC
CUL2	CLTC
CUL2	CMBL
MIB1	CMIP
MOV10	CMPK1
CLIP4	CMYA5
RPL36	CNBP
MARK3	CNBP
TOR1A	CNIH4
RHOA	CNKSR1
RALA	CNKSR2
UPF1	CNOT2
PABPC1	CNOT6
PABPC1	CNOT7
ERLEC1	CNTNAP3
MAP3K4	CNTRL
MAPK6	CNTRL
PIGS	CNTROB
GOLGA3	CNTROB
NDUFAF1	COA3
ECSIT	COA3
MOV10	COA3
CEP250	COG3
CSNK2A2	COIL
COMT	COL1A2
MOV10	COL4A3
CSNK2A2	COL5A1
DCN	COL6A1
VHL	COL6A1
COLEC11	COL6A1
PDGFB	COL6A1
MAG	COL6A1
FBXO6	COL6A1
DNAJB11	COL6A1
STC2	COLEC10
INHBE	COLEC10
ELAVL1	COLGALT1
ATF2	COLGALT1
FBXO6	COLGALT1
RNF2	COLGALT1

ELOC	COMMD1
CUL2	COMMD1
EXOSC2	COMMD4
CDC27	COMT
XRN2	COMT
TRIP13	COMT
HEATR3	COMTD1
NUP98	COMTD1
RBX1	COP1
CUL2	COP1
ELOB	COP1
SUN2	COPB1
SUN2	COPG1
MOV10	COPRS
GFER	COPS2
RAB5C	COPS2
CUL2	COPS2
RBX1	COPS3
ELOB	COPS3
CUL2	COPS3
ELOB	COPS4
RBX1	COPS4
TOR1A	COPS4
CUL2	COPS4
LARP4B	COPS5
RBX1	COPS5
MRPS27	COPS5
ELOB	COPS5
UPF1	COPS5
CUL2	COPS5
PABPC1	COPS5
CSDE1	COPS5
FAM98A	COPS5
PABPC4	COPS5
AKAP8	COPS5
SLC30A9	COPS5
MAP7D1	COPS6
PABPC1	COPS6
CUL2	COPS6
ELOC	COPS6
DCAF7	COPS6
ELOB	COPS6
RBX1	COPS6
FBXL12	COPS6
CENPF	COPS6
ZYG11B	COPS6
RAE1	COPS6
RBX1	COPS7A
PRKAR2B	COPS7A
CUL2	COPS7A
ERP44	COPS7B
CUL2	COPS7B
RAE1	COPS8
GFER	COPS8
CUL2	COPS8
CUL2	COPS9
REEP6	COQ8A
SUN2	CORO1C
ELOC	CORO7
RBX1	CORO7
ELOB	CORO7

EXOSC8	COX5A
G3BP1	COX5A
RAB5C	COX5B
RAB5C	COX6B1
RAB2A	COX6C
POLA2	CPE
RAB7A	CPM
CEP250	CPNE7
TUBGCP3	CPNE7
MARK3	CPSF3
MARK3	CPSF4
TLE5	CPSF7
RAB7A	CPT1A
ELOC	CPTP
ERLEC1	CPVL
CYB5R3	CPVL
NAT14	CPVL
TLE5	CRACR2A
GHITM	CRACR2B
RIPK1	CRADD
RBX1	CRBN
TCF12	CRCP
MOV10	CRCP
PRKACA	CREB1
HMOX1	CREB3
CYB5B	CREB3
POLA2	CRELD1
ATP1B1	CRIP2
PPT1	CRIP2
PRKACA	CRK
AP2M1	CRK
PRRC2B	CRK
CEP250	CRTC2
HCCS	CRTC3
PCDHB15	CRTC3
POLD1	CRTC3
XPO1	CRTC3
GTSE1	CRTC3
NEDD1	CRTC3
HSPA5	CRTC3
MAPK8	CRTC3
MCM2	CRTC3
MYCBP2	CRY1
AKAP8	CRY2
MYCBP2	CRY2
PRKAR2B	CRYBG3
CSNK2A2	CRYZ
PABPC1	CSF2
AKAP9	CSNK1D
PRKAR2B	CSNK1D
CUL2	CSNK1E
PRKAR2A	CSNK1E
CSNK2B	CSNK1E
RBX1	CSNK1E
ELOB	CSNK1E
CSNK2A2	CSNK2A1
KDM1A	CSNK2A2
CDK11A	CSNK2A2
CREBBP	CSNK2A2
GCFC2	CSNK2A2
LAMC3	CSNK2A2

LUC7L	CSNK2A2
BCLAF1	CSNK2A2
THRAP3	CSNK2A2
CTDP1	CSNK2A2
YAF2	CSNK2A2
KIF1B	CSNK2A2
RABEP1	CSNK2A2
PHRF1	CSNK2A2
IFT88	CSNK2A2
DNMT1	CSNK2B
AP2M1	CSNK2B
CSNK2B	CSNK2B
MEPCE	CSNK2B
UPF1	CSNK2B
BRD2	CSNK2B
CSNK2A2	CSNK2B
TLE1	CSNK2B
TERF1	CSNK2B
ADD1	CSNK2B
CDK20	CSNK2B
SPSB3	CSNK2B
PPP2CA	CSNK2B
GBP2	CSNK2B
MAPK14	CSNK2B
CBX2	CSNK2B
FN1	CSNK2B
EIF3H	CSNK2B
ATG101	CSNK2B
RNF126	CSNK2B
BRCA1	CSNK2B
CRY1	CSNK2B
RASSF10	CSNK2B
PITPNA	CSNK2B
RNF2	CSNK2B
PFKFB4	CSNK2B
ANGPT2	CSNK2B
NPAS2	CSNK2B
BTRC	CSNK2B
BHLHE40	CSNK2B
SRRM1	CSNK2B
TRIB3	CSNK2B
NMT2	CSNK2B
GRK2	CSNK2B
PPP2R1B	CSNK2B
PTPRK	CSNK2B
ABHD4	CSNK2B
CD163	CSNK2B
LCK	CSNK2B
KDM5C	CSNK2B
PCGF5	CSNK2B
PHF11	CSNK2B
EPB41L3	CSNK2B
ATXN3	CSNK2B
HDAC1	CSNK2B
NRF1	CSNK2B
SIMC1	CSNK2B
CDK11A	CSNK2B
CPNE7	CSNK2B
COPS7A	CSNK2B
SEC11A	CSNK2B
PHC2	CSNK2B

KIF5C	CSNK2B
COPS3	CSNK2B
RNF7	CSNK2B
GAMT	CSNK2B
ARNTL	CSNK2B
FBXL15	CSNK2B
CCDC115	CSNK2B
VRK3	CSNK2B
MTIF2	CSNK2B
CSNK2A1	CSNK2B
TERF2	CSNK2B
RPA1	CSNK2B
STK16	CSNK2B
FAF1	CSNK2B
CDK9	CSNK2B
SDHA	CSNK2B
APLP1	CSNK2B
FGF2	CSNK2B
ITGA4	CSNK2B
PYROXD2	CSNK2B
GCH1	CSNK2B
TAF1D	CSNK2B
RPS6KA5	CSNK2B
PHRF1	CSNK2B
DALRD3	CSNK2B
COIL	CSNK2B
CIPC	CSNK2B
CDK16	CSNK2B
PPP2R1A	CSNK2B
UNK	CSNK2B
RPS6KA2	CSNK2B
CENPB	CSNK2B
OBSL1	CSNK2B
ADH5	CSNK2B
ACTR1B	CSNK2B
RASSF9	CSNK2B
CDC34	CSNK2B
BCCIP	CSNK2B
PRKCZ	CSNK2B
WDR18	CSNK2B
EPS8	CSNK2B
CLOCK	CSNK2B
PPID	CSNK2B
BMI1	CSNK2B
CUL7	CSNK2B
CIC	CSNK2B
STAT5A	CSNK2B
FBXL5	CSNK2B
EIF2S2	CSNK2B
DACT1	CSNK2B
RYBP	CSNK2B
RPS6KA1	CSNK2B
ZNF44	CSNK2B
WFIKKN1	CSNK2B
ZNF784	CSNK2B
CTDP1	CSNK2B
PROC	CSNK2B
PML	CSNK2B
CHRNA4	CSNK2B
PCGF3	CSNK2B
SUMO2	CSNK2B

PPP2R5D	CSNK2B
MCRS1	CSNK2B
SLX4	CSNK2B
POT1	CSNK2B
MPP2	CSNK2B
GABARAPL1	CSNK2B
NCF1	CSNK2B
HEXB	CSNK2B
KDM6B	CSNK2B
EZH2	CSNK2B
SUZ12	CSNK2B
RPAP1	CSNK2B
HIRIP3	CSNK2B
CACNA1A	CSNK2B
FGB	CSNK2B
ACVRL1	CSNK2B
SIRT1	CSNK2B
ZNF71	CSNK2B
CALM1	CSNK2B
NKAPD1	CSNK2B
APC	CSNK2B
PRKAB2	CSNK2B
TFAP2A	CSNK2B
RNF11	CSNK2B
TNFAIP1	CSNK2B
SPOUT1	CSNK2B
VCP	CSNK2B
FTH1	CSNK2B
CBX8	CSNK2B
LRP5L	CSNK2B
PPP1CC	CSNK2B
ZNHIT1	CSNK2B
NFKBIA	CSNK2B
USP43	CSNK2B
GSK3B	CSNK2B
CBX7	CSNK2B
COL4A2	CSNK2B
HEXIM1	CSNK2B
LMO4	CSNK2B
TP53BP2	CSNK2B
PAXIP1	CSNK2B
IFI16	CSNK2B
RPS6KB1	CSNK2B
XPO1	CSNK2B
SYNE4	CSNK2B
PLXNA3	CSNK2B
CDKN1A	CSNK2B
UTP14A	CSNK2B
PDS5A	CSNK2B
WDR1	CSNK2B
STAU1	CSNK2B
CBX4	CSNK2B
TP53	CSNK2B
GLB1	CSNK2B
AUTS2	CSNK2B
OGA	CSNK2B
RPL5	CSNK2B
NOLC1	CSNK2B
ODC1	CSNK2B
NRBP1	CSNK2B
RPS6KA3	CSNK2B

RPS6KB2	CSNK2B
PER2	CSNK2B
ACTR5	CSNK2B
ACTB	CSNK2B
FGF1	CSNK2B
RXRA	CSNK2B
PCCB	CSNK2B
INTS11	CSNK2B
TCEAL7	CSNK2B
RORB	CSNK2B
TSEN54	CSNK2B
NXF1	CSNK2B
TUBB2B	CSNK2B
NTRK1	CSNK2B
HNRNPA2B1	CSNK2B
DENND5A	CSNK2B
LEPR	CSNK2B
CDK2AP1	CSNK2B
PPP3CC	CSNK2B
MOXD1	CSNK2B
RFC1	CSNK2B
PPP2R5E	CSNK2B
GNB5	CSNK2B
WEE1	CSNK2B
HTRA1	CSNK2B
GADD45GIP1	CSNK2B
PER1	CSNK2B
PPP1CA	CSNK2B
MBD4	CSNK2B
NOTCH3	CSNK2B
EIF1B	CSNK2B
MAPKAPK2	CSNK2B
ARAF	CSNK2B
NAP1L1	CSNK2B
CD81	CSNK2B
ATF1	CSNK2B
LYST	CSNK2B
MAST3	CSNK2B
DYNC1H1	CSNK2B
NPM1	CSNK2B
EIF3F	CSNK2B
RPL13	CSNK2B
VCAM1	CSNK2B
BHLHE41	CSNK2B
KIF2C	CSNK2B
WDR48	CSNK2B
ERCC8	CSNK2B
RAD51	CSNK2B
BTF3	CSNK2B
CCNH	CSNK2B
TOP2B	CSNK2B
IKBKB	CSNK2B
CUL2	CSTA
GOLGA2	CSTF2T
CEP68	CTBP1
MARK3	CTBP2
MOV10	CTDNEP1
MAP7D1	CTDSP2
AP2M1	CTLA4
NUP98	CTNNB1
SLC9A3R1	CTNNB1

AP2M1	CTNNB1
MOV10	CTNNB1
NSD2	CTNNB1
AP2M1	CTNND1
CUL2	CTPS1
TUBGCP3	CTPS1
FKBP15	CTR9
ELOB	CTR9
ERGIC1	CTR9
ELOC	CTR9
CUL2	CTSD
ATP6V1A	CTSD
AKAP8L	CUL1
ELOB	CUL2
PABPC4	CUL2
PABPC1	CUL2
RBX1	CUL2
AKAP8L	CUL2
HNRNPM	CUL2
ARID1B	CUL2
HSPA5	CUL2
HNRNPC	CUL2
DDX17	CUL2
SLC25A5	CUL2
TRIM9	CUL2
UBE2D1	CUL2
ACTB	CUL2
TBC1D17	CUL2
ACTA2	CUL2
TUBB1	CUL2
DNAJB6	CUL2
YBX3	CUL2
SEC23A	CUL2
EEF1D	CUL2
MRTO4	CUL2
DNMT3B	CUL2
MKNK2	CUL2
CCDC22	CUL2
DAZAP1	CUL2
NUCB2	CUL2
TFE3	CUL2
CASP14	CUL2
SAMHD1	CUL2
SNRPA	CUL2
RPS16	CUL2
HNRNPUL1	CUL2
FUS	CUL2
GSTP1	CUL2
RPS20	CUL2
ENO1	CUL2
RPL31	CUL2
NUDT1	CUL2
KEAP1	CUL2
PTOV1	CUL2
ASB4	CUL2
ASB2	CUL2
KLC3	CUL2
CDC34	CUL2
PLOD1	CUL2
APPBP2	CUL2
SLC25A3	CUL2

HNRNPH3	CUL2
HSP90AA1	CUL2
RPLP0	CUL2
RPL6	CUL2
SERPINB3	CUL2
RNMT	CUL2
EMD	CUL2
EEF1A2	CUL2
HIF1A	CUL2
CEP152	CUL2
YBX1	CUL2
NDRG1	CUL2
DDX1	CUL2
PKM	CUL2
TIMM13	CUL2
RPL3	CUL2
TNRC6B	CUL2
RPL18	CUL2
DLG3	CUL2
EDRF1	CUL2
RTRAF	CUL2
RTCB	CUL2
TMCC3	CUL2
DCUN1D1	CUL2
KHSRP	CUL2
GDPD3	CUL2
NSFL1C	CUL2
DERL2	CUL2
ELAVL1	CUL2
DNAJA1	CUL2
MTHFD1	CUL2
PHGDH	CUL2
SNRNP70	CUL2
MTHFSD	CUL2
DSP	CUL2
SNW1	CUL2
HSP90AB1	CUL2
SNRPD3	CUL2
RBM3	CUL2
DNAJC10	CUL2
SMC3	CUL2
BRAP	CUL2
OSGEP	CUL2
CIRBP	CUL2
SH3GLB1	CUL2
SDHA	CUL2
CSDE1	CUL3
AKAP8L	CUL3
UPF1	CUL3
DCAF7	CUL4A
PABPC4	CUL4A
PABPC1	CUL4A
RBX1	CUL4A
GNB1	CUL4A
CUL2	CUL4B
LARP1	CUL4B
PABPC1	CUL4B
TLE3	CUL4B
PABPC4	CUL4B
RBX1	CUL4B
MYCBP2	CUL4B

ELOC	CUL5
MRPS2	CUL5
LARP1	CUL5
PABPC1	CUL5
RBX1	CUL5
FAM98A	CUL5
ELOB	CUL5
PABPC4	CUL5
DCAF7	CUL5
CUL2	CUL5
UPF1	CUL5
ERP44	CUL7
RALA	CUL7
RBX1	CUL9
SIRT5	CWC15
EXOSC8	CWC22
FRA10AC1	CWC27
HMGA1	CWC27
ESR2	CWC27
EPB41L3	CWC27
VTI1B	CWC27
DNMT1	CXXC1
ENO1	CYB5B
ELAVL1	CYB5B
ENO1	CYB5R3
MOV10	CYC1
USP13	CYLD
CYB5R3	CYP1A1
POR	CYP2A6
ECSIT	CYP2C8
POR	CYP2C9
ECSIT	CYP2C9
POR	CYP2E1
RBX1	DAB2IP
ITGB1	DAG1
MRPS27	DAP3
CEP250	DAP3
MIB1	DAPK1
HDAC2	DAXX
DNMT1	DAXX
GOLGA2	DAXX
ATP6AP1	DBN1
PPT1	DBT
CEP250	DCAF1
RBX1	DCAF1
CLIP4	DCAF6
CSNK2A2	DCAF7
PRKACA	DCAF7
DYRK1B	DCAF7
GTSE1	DCAF7
TRAF1	DCAF7
CBX7	DCAF7
TRAF3	DCAF7
RNF2	DCAF7
TCP1	DCAF7
YWHAH	DCAF7
YWHAE	DCAF7
FBXW11	DCAF7
CCT4	DCAF7
DIAPH1	DCAF7
AURKA	DCAF7

XPO1	DCAF7
HIPK2	DCAF7
BIRC3	DCAF7
DYRK2	DCAF7
MED23	DCAF7
CEP76	DCAF7
BIRC2	DCAF7
EPAS1	DCAF7
AGO2	DCAF7
CSNK2A1	DCAF7
FAF2	DCAF7
CCT7	DCAF7
MAP3K1	DCAF7
TUBGCP3	DCAF8
FAM189A2	DCAKD
UBC	DCAKD
SRPRB	DCAKD
MCM2	DCAKD
MCOLN3	DCAKD
ELAVL1	DCAKD
GGCX	DCP2
UPF1	DCP2
POLA2	DCP2
IDE	DCP2
CSNK2A2	DCPS
OS9	DCSTAMP
MOV10	DCTN1
ARF6	DCTN1
NINL	DCTN5
DCTPP1	DCTPP1
WDYHV1	DCTPP1
NXF1	DCTPP1
APP	DCTPP1
SDCBP	DCTPP1
NOA1	DCTPP1
CHMP4B	DCTPP1
NEDD8	DCTPP1
PLK1	DCTPP1
VHL	DCTPP1
CUL5	DCTPP1
CRY2	DCTPP1
CUL2	DCUN1D2
ELOB	DCUN1D2
RBX1	DCUN1D2
ELOB	DCUN1D3
CUL2	DCUN1D3
RBX1	DCUN1D3
CUL2	DCUN1D4
RBX1	DCUN1D4
RBX1	DCUN1D5
CUL2	DCUN1D5
RBX1	DDA1
ATP1B1	DDAH2
RBX1	DDB1
MIB1	DDB1
TUBGCP2	DDB1
DCAF7	DDB1
CEP250	DDB1
RBX1	DDB2
MOV10	DDOST
ARF6	DDOST

MOV10	DDR1
G3BP2	DDX10
ERBB2	DDX10
APP	DDX10
BYSL	DDX10
MNDA	DDX10
OBSL1	DDX10
SRFBP1	DDX10
NOP56	DDX10
EED	DDX10
IFI16	DDX10
CAND1	DDX10
EXOSC5	DDX17
NUP88	DDX19A
NUP214	DDX19A
NUP214	DDX19B
HMOX1	DDX19B
RAE1	DDX19B
CEP250	DDX21
ERG	DDX21
RRP1B	DDX21
SRPK2	DDX21
OBSL1	DDX21
LYAR	DDX21
CUL4A	DDX21
CDK2	DDX21
DHX9	DDX21
ZC3H3	DDX21
GABARAPL2	DDX21
PSTPIP1	DDX21
IBTK	DDX21
MCM5	DDX21
CUL7	DDX21
EED	DDX21
YWHAE	DDX21
ITGA4	DDX21
HNRNPA1	DDX21
NOP56	DDX21
PRKAB1	DDX21
SRRM1	DDX21
APTX	DDX21
HDGF	DDX21
TERF2	DDX21
PCK1	DDX21
FBXO6	DDX21
MNDA	DDX21
CTNNBL1	DDX21
CUL3	DDX21
NFKB2	DDX21
EBNA1BP2	DDX21
HUWE1	DDX21
SRPK1	DDX21
RPL10	DDX21
YBX1	DDX21
MAP3K14	DDX21
TERF1	DDX21
PAN2	DDX21
HNRNPC	DDX21
CDC5L	DDX21
MED19	DDX21
KPNA3	DDX21

THUMPD1	DDX21
STAU1	DDX21
SSRP1	DDX21
TARDBP	DDX21
MCM2	DDX21
MACROD1	DDX21
ESR1	DDX21
VCAM1	DDX21
PPP2R2B	DDX21
SMNDC1	DDX21
FN1	DDX21
TOP2A	DDX21
MAST3	DDX21
DOT1L	DDX21
GABARAPL1	DDX21
IVNS1ABP	DDX21
RTCA	DDX21
HNRNPU	DDX21
MEPCE	DDX23
AAR2	DDX23
BCS1L	DDX24
POLA2	DDX24
RAE1	DDX39B
SUN2	DDX3X
TBK1	DDX3X
CUL2	DDX3X
AP2M1	DDX3X
MOV10	DDX41
CEP250	DDX41
MPHOSPH10	DDX41
RBM28	DDX41
MEPCE	DDX42
MOV10	DDX42
CUL2	DDX5
AKAP8	DDX5
CSNK2A2	DDX54
CUL2	DDX56
HECTD1	DDX56
MRPS25	DDX56
TLE5	DDX6
EXOSC5	DECR1
MYCBP2	DEF6
CSNK2A2	DEK
GRPEL1	DENND2D
SELENOS	DERL1
RTN4	DERL1
POR	DERL1
HMOX1	DERL1
EMC1	DERL1
ERLEC1	DERL2
CLIP4	DES
PLEKHA5	DES
MOV10	DES
RBX1	DET1
MOV10	DFFA
CUL2	DFFB
TCF12	DGCR6
NUP54	DGCR6
TLE5	DGCR6
MIPOL1	DGCR6
GOLGA2	DGCR6

RAE1	DGCR8
RHOA	DGKQ
TOR1A	DHCR7
LOX	DHDDS
EXOSC5	DHRS2
CUL2	DHX15
POLA2	DHX34
CEP250	DHX9
AKAP8L	DHX9
CUL2	DHX9
USP13	DIABLO
RHOA	DIAPH1
RHOA	DIAPH3
ELOC	DIO2
ELOB	DIO2
RBX1	DIO2
TLE5	DIP2A
RHOA	DIRAS3
RAP1GDS1	DIRAS3
HECTD1	DIRAS3
EXOSC5	DIS3
EXOSC2	DIS3L
EXOSC5	DIS3L
EXOSC8	DIS3L
EIF4E2	DIS3L
EXOSC3	DIS3L
CIT	DISC1
GNB1	DISC1
AKAP9	DISC1
PCNT	DISC1
PPT1	DLAT
CUL2	DLC1
MARK3	DLG5
MARK2	DLG5
MIB1	DLL1
CCDC86	DLST
USP13	DLST
DNMT1	DMAP1
BRD4	DMAP1
NUP58	DMD
MARK2	DMD
MOV10	DMTN
SDF2	DMWD
UPF1	DNA2
RAB2A	DNAAF2
MOV10	DNAAF5
BCS1L	DNAJA1
ECSIT	DNAJB1
MOV10	DNAJB4
MRPS27	DNAJC1
ATP6V1A	DNAJC13
TIMM17A	DNAJC19
FBXO31	DNAJC19
APP	DNAJC19
TSPAN5	DNAJC19
P2RX4	DNAJC19
PIGS	DNAJC8
MOV10	DNAJC9
HECTD1	DNM3
DNMT1	DNMT1
GADD45A	DNMT1

SFRP5	DNMT1
CBX5	DNMT1
KDM1A	DNMT1
MYH14	DNMT1
CDK2	DNMT1
GSK3B	DNMT1
HSPA5	DNMT1
RPS6KA6	DNMT1
MBD3	DNMT1
BAZ2A	DNMT1
TRIM28	DNMT1
DNMT3B	DNMT1
EED	DNMT1
HELLS	DNMT1
NR2C1	DNMT1
PTN	DNMT1
SFRP4	DNMT1
SUV39H1	DNMT1
EPDR1	DNMT1
HDAC1	DNMT1
GATA5	DNMT1
RELB	DNMT1
EZH2	DNMT1
TCF3	DNMT1
ACTB	DNMT1
RUNX1T1	DNMT1
TIMP3	DNMT1
BRCA1	DNMT1
BRAP	DNMT1
SIRT1	DNMT1
MLH1	DNMT1
DNMT3A	DNMT1
CHD4	DNMT1
CBX1	DNMT1
MYL6	DNMT1
POLD3	DNMT1
RAP1GDS1	DOC2A
MEPCE	DOCK1
RAB2A	DOCK6
REEP5	DOCK6
EXOSC5	DOCK8
IDE	DOHH
RAB10	DOK2
NIN	DOT1L
OS9	DPEP2
CHPF2	DPEP2
ENO1	DPH5
CSNK2A2	DPP9
LYPD3	DPY19L1
LDLRAD4	DPY19L1
FOXRED2	DPY30
PRRC2B	DPY30
PKP2	DROSHA
CSNK2A2	DSC2
PKP2	DSC2
PKP2	DSP
CLIP4	DST
RAB14	DSTYK
RAB5C	DSTYK
NSD2	DTL
MARK2	DTNA

MOV10	DTYMK
MEPCE	DTYMK
EXOSC8	DUSP23
USP13	DVL1
STOM	DVL3
UPF1	DXO
SPART	DYNC1H1
TOR1A	DYNC1H1
ARF6	DYNC1H1
PRKAR2B	DYNC1I2
PABPC1	DYNLL1
PRKACA	DYNLL1
PVR	DYNLT1
DCAF7	DYRK1A
PRKACA	DYRK1A
MOV10	DYRK1A
PRKACA	DYRK1B
OS9	DYSF
MOV10	E2F4
CHMP2A	E2F4
TLE5	EAF1
FBXL12	ECSIT
ECSIT	ECSIT
GCDH	ECSIT
BLMH	ECSIT
CDC37	ECSIT
FAF2	ECSIT
NDUFA8	ECSIT
AP1M2	ECSIT
COA1	ECSIT
DECR1	ECSIT
MTIF2	ECSIT
STRN4	ECSIT
ACADVL	ECSIT
IFIT3	ECSIT
NDUFS1	ECSIT
CLPP	ECSIT
PSEN1	ECSIT
MAVS	ECSIT
ABCC10	ECSIT
FBXW4	ECSIT
TIMMDC1	ECSIT
NDUFAF4	ECSIT
IARS2	ECSIT
LLGL2	ECSIT
MAST1	ECSIT
GCAT	ECSIT
MGME1	ECSIT
RHEB	ECSIT
NFKB1	ECSIT
FAS	ECSIT
RNF32	ECSIT
NDUFS8	ECSIT
NNT	ECSIT
RAB3A	ECSIT
NUP98	ECT2
ALG5	EDA
AP3B1	EDA
CWC27	EDA
UPF1	EDC3
UPF1	EDC4

SEL1L	EDEM3
PKP2	EED
TUBGCP2	EEF1A1
MOV10	EEF1A1
SUN2	EEF1A1
CUL2	EEF1A1
RBX1	EEF1A1
CEP250	EEF1A1
CUL2	EEF1B2
UPF1	EEF2
SUN2	EEF2
RAE1	EEF2K
CSNK2A2	EFCAB14
LOX	EFEMP2
MOV10	EFNA4
PKP2	EFNB2
CUL2	EGFLAM
MOV10	EGFLAM
TARS2	EGFR
BZW2	EGFR
RBM41	EGFR
RAB18	EGFR
ACSL3	EGFR
DNAJC11	EGFR
RAB7A	EGFR
PLD3	EGFR
RAP1GDS1	EGFR
RIPK1	EGFR
OS9	EGFR
SDF2	EGFR
TOR1AIP1	EGFR
ATP1B1	EGFR
NDFIP2	EGFR
PKP2	EGFR
PRKACA	EGFR
TUBGCP2	EGFR
TMEM97	EGFR
FAM98A	EGFR
OS9	EGLN1
DNMT1	EHMT2
COLGALT1	EHMT2
PRRC2B	EHMT2
HDAC2	EHMT2
MOV10	EHMT2
PTGES2	EID3
SLC27A2	EID3
MOV10	EIF1AD
SUN2	EIF1AD
RAB7A	EIF1B
EIF4H	EIF1B
BZW2	EIF2A
CSNK2A2	EIF2B2
CHMP2A	EIF2B5
UPF1	EIF2S1
UPF1	EIF2S2
UPF1	EIF3A
UPF1	EIF3B
CSNK2A2	EIF3B
PRRC2B	EIF3F
TIMM29	EIF3F
EIF4E2	EIF3F

CSNK2A2	EIF3F
CSNK2A2	EIF3H
PRRC2B	EIF3H
EIF4E2	EIF3H
CSNK2A2	EIF3J
MEPCE	EIF4A1
PABPC1	EIF4A1
SUN2	EIF4A1
EIF4H	EIF4A1
PABPC1	EIF4A2
TARS2	EIF4A2
PABPC1	EIF4A3
UPF1	EIF4A3
TLE5	EIF4E2
TUBGCP3	EIF4E2
LZTS2	EIF4E2
SPAG5	EIF4E2
XPO1	EIF4E2
HUWE1	EIF4E2
MDFI	EIF4E2
AMOTL2	EIF4E2
FBXW11	EIF4E2
NECAB2	EIF4E2
WDR83	EIF4E2
YWHAE	EIF4E2
YWHAH	EIF4E2
TMCC2	EIF4E2
MAPRE3	EIF4E2
USP4	EIF4E2
USHBP1	EIF4E2
EIF4E2	EIF4EBP1
CEP250	EIF4EBP3
EIF4E2	EIF4EBP3
EIF4E2	EIF4ENIF1
CEP250	EIF4ENIF1
PABPC1	EIF4G1
PABPC1	EIF4G3
LNX1	EIF4H
CUL7	EIF4H
CUL2	EIF5A
PTBP2	EIF5A2
WFS1	EIF6
POLA2	EIF6
ARF6	EIF6
IMPDH2	EIF6
GRPEL1	EIF6
CSNK2B	EIF6
MAT2B	ELAVL1
SLC30A9	ELAVL1
ERP44	ELAVL1
RALA	ELAVL1
CSDE1	ELAVL1
PLEKHA5	ELAVL1
ECSIT	ELAVL3
AKAP9	ELF3
ETFA	ELF3
RBX1	ELOB
ASB2	ELOB
DCUN1D1	ELOB
NOS2	ELOB
CFTR	ELOB

USP11	ELOB
CUL3	ELOB
USP33	ELOB
ELOA	ELOB
TSC22D1	ELOB
ASB1	ELOB
ORC1	ELOB
CEBPE	ELOB
ASB4	ELOB
MCM2	ELOB
HIF1A	ELOB
APPBP2	ELOB
OSGEP	ELOB
ASB9	ELOB
CUL2	ELOC
RBX1	ELOC
ELOB	ELOC
RAB40B	ELOC
ASB3	ELOC
POP1	ELOC
OSGEP	ELOC
USP33	ELOC
PCK1	ELOC
SGO1	ELOC
MYBBP1A	ELOC
PCGF1	ELOC
EXT2	ELOC
EFNB3	ELOC
TOP2A	ELOC
ARID1B	ELOC
CHMP4B	ELOC
KMT2A	ELOC
NEDD8	ELOC
FBXO7	ELOC
SAT2	ELOC
CUL3	ELOC
TSC22D1	ELOC
NOS2	ELOC
ASB1	ELOC
PPRC1	ELOC
CFTR	ELOC
JTB	ELOC
SPSB2	ELOC
FAF2	ELOC
ASB9	ELOC
CISH	ELOC
NRBP1	ELOC
ID2	ELOC
ELOA	ELOC
LRRC41	ELOC
FUS	ELOC
SNRPN	ELOC
VHL	ELOC
CTDSPL2	ELOC
SKP2	ELOC
COPS5	ELOC
ID1	ELOC
CYP2J2	ELOC
KLHDC3	ELOC
RNF7	ELOC
EIF1B	ELOC

SOCS2	ELOC
COPS4	ELOC
ASB2	ELOC
PAN2	ELOC
WSB1	ELOC
ECT2	ELOC
ID3	ELOC
CENPC	ELOC
CBX5	ELOC
ASB6	ELOC
DCUN1D1	ELOC
COPS3	ELOC
HIF1A	ELOC
APPBP2	ELOC
FAF2	EMC1
ATP1B3	EMC1
FBXO6	EMC1
TSC22D1	EMC1
CUL3	EMC1
CANX	EMC1
EMC2	EMC1
EMC3	EMC1
ENO1	EMC1
DERL2	EMC1
CUL7	EMC1
MOV10	EMC10
AGPS	EMC2
EMC1	EMC4
EMC1	EMC7
EMC1	EMC8
FOXRED2	EMD
AKAP8L	EMD
MOV10	EMSY
CSNK2A2	EMSY
RBX1	ENC1
CUL2	ENO2
RAB10	ENOPH1
NDUFB9	ENPP6
PIGS	ENPP6
CEP250	ENTR1
ELOB	EPAS1
AKAP8L	EPAS1
RAB10	EPB41
RAB2A	EPB41
ELOC	EPB41
RAB5C	EPB41
CSNK2A2	EPB41L3
ERP44	EPB41L4B
GNB1	EPHA7
SLC9A3R1	EPHB1
CUL2	EPOR
ELOB	EPOR
ELOC	EPOR
BCKDK	EPS8
MRPS2	ERAL1
MOV10	ERAP1
MTCH1	ERBB2
ACSL3	ERBB2
ATP6AP1	ERBB2
GNB1	ERBB2
PKP2	ERBB2

DVL2	ERC1
CUL3	ERC1
PLAT	ERG
RPA3	ERGIC1
EFNB1	ERGIC1
SYN1	ERGIC1
SLC30A9	ERGIC3
DNAJC11	ERGIC3
ERGIC1	ERGIC3
FAR2	ERGIC3
HSPA5	ERLEC1
TNFRSF1A	ERLEC1
RAB7A	ERLIN1
RAB7A	ERLIN2
SCAP	ERLIN2
ELAVL1	ERMP1
MCOLN3	ERMP1
ERP44	ERO1A
ERO1B	ERO1A
ERP44	ERO1B
BRCA1	ERO1B
MOV10	ESCO2
GNB1	ESR1
UPF1	ESR1
PABPC1	ESR1
MRPS2	ESR2
GOLGA3	ESR2
MRPS27	ESR2
GORASP1	ESR2
AKAP9	ESR2
RPL36	ESR2
GNB1	ESRRB
PKP2	ESRRB
PABPC1	ETF1
UPF1	ETF1
CUL7	ETFA
FASTKD3	ETFA
DDA1	ETFA
ETFB	ETFA
EIF2S2	ETFA
SIRT4	ETFA
MYC	ETFA
GLS	ETFA
ATG101	ETFA
CDC42	ETFA
NNT	ETFA
PSEN1	ETFA
EIF1B	ETFA
FTSJ1	ETFA
HDAC5	ETFA
HNRNPD	ETFA
PRKAB1	ETFA
NDUFAB1	ETFA
MPG	ETFA
UBA5	ETFA
SMS	ETFA
MOV10	ETFDH
ETFA	ETFRF1
MOV10	ETV5
RAB10	EVI5L
PLEKHA5	EVPL

NINL	EWSR1
CUL2	EWSR1
COMT	EXO1
RALA	EXOC2
ECSIT	EXOC6
MOV10	EXOC7
EXOSC2	EXOSC1
ECSIT	EXOSC1
EXOSC5	EXOSC1
EXOSC8	EXOSC1
EXOSC3	EXOSC1
EXOSC8	EXOSC10
CHPF	EXOSC10
EXOSC3	EXOSC10
PTGES2	EXOSC10
UPF1	EXOSC10
EXOSC5	EXOSC10
MOV10	EXOSC10
EXOSC2	EXOSC10
UPF1	EXOSC2
EXOSC8	EXOSC2
EXOSC3	EXOSC2
EXOSC5	EXOSC2
KHSRP	EXOSC2
PEX1	EXOSC2
METTL22	EXOSC2
ORC1	EXOSC2
NCL	EXOSC2
CAND1	EXOSC2
DIS3	EXOSC2
FBXL4	EXOSC2
MAP1LC3A	EXOSC2
NT5DC3	EXOSC2
RNF8	EXOSC2
EXOSC7	EXOSC2
ST6GALNAC1	EXOSC2
EXOSC9	EXOSC2
EXOSC3	EXOSC3
EXOSC5	EXOSC3
GABARAPL2	EXOSC3
ZCCHC8	EXOSC3
ATP5F1D	EXOSC3
DIS3	EXOSC3
DDX17	EXOSC3
CDK5RAP1	EXOSC3
MTREX	EXOSC3
EXOSC7	EXOSC3
UPF1	EXOSC4
EXOSC3	EXOSC4
EXOSC8	EXOSC4
EXOSC2	EXOSC4
EXOSC5	EXOSC4
ETFA	EXOSC4
EXOSC7	EXOSC5
PKM	EXOSC5
CUL3	EXOSC5
WDR3	EXOSC5
MTREX	EXOSC5
EXOSC2	EXOSC6
CEP250	EXOSC6
EXOSC8	EXOSC6

EXOSC3	EXOSC6
CHPF	EXOSC6
EXOSC5	EXOSC6
EXOSC3	EXOSC8
EXOSC8	EXOSC8
EXOSC5	EXOSC8
ATF2	EXOSC8
PXN	EXOSC8
RPA2	EXOSC8
COL23A1	EXOSC8
TFAP4	EXOSC8
TXNDC9	EXOSC8
ZCCHC8	EXOSC8
CRMP1	EXOSC8
FRG1	EXOSC8
HBS1L	EXOSC8
MTREX	EXOSC8
DIS3	EXOSC8
RASSF1	EXOSC8
XRN1	EXOSC8
MTMR3	EXOSC8
EXOSC7	EXOSC8
MED23	EXOSC8
FOXN3	EXOSC8
RPA3	EXOSC8
METTL22	EXOSC8
XRN2	EXOSC8
EXOSC8	EXOSC9
EXOSC3	EXOSC9
EXOSC5	EXOSC9
HYOU1	EXT2
PABPC4	EZH2
NINL	EZH2
AKAP8	EZH2
POLA2	EZH2
RBX1	EZH2
UPF1	EZH2
PLAT	F10
MOV10	F2RL1
COPS5	F2RL1
ZW10	F2RL1
SLMAP	F2RL1
RINT1	F2RL1
GEMIN6	F2RL1
TUBGCP3	FABP5
RIPK1	FADD
G3BP1	FAF1
CUL2	FAF1
MYCBP2	FAF1
NUP98	FAF1
NGLY1	FAF1
RAE1	FAF1
USP13	FAF2
RAB7A	FAF2
CUL2	FAF2
RAB5C	FAF2
NINL	FAM107A
MOV10	FAM111B
RAB2A	FAM114A1
BAG5	FAM118B
RAE1	FAM122B

TLE5	FAM124A
TLE5	FAM124B
ERP44	FAM13A
MIPOL1	FAM161A
NINL	FAM161A
UBL4A	FAM162A
CPVL	FAM162A
HSP90AA1	FAM162A
SIAE	FAM162A
SLC27A2	FAM177A1
RTN4	FAM210B
POLA2	FAM219A
ATE1	FAM219A
RTN4	FAM241A
MOV10	FAM83G
ERLEC1	FAM8A1
OS9	FAM8A1
GOLT1B	FAM8A1
FAM189A2	FAM8A1
CAPRIN2	FAM8A1
MBTPS2	FAM8A1
SEL1L	FAM8A1
GOLGA2	FAM90A1
EXOSC8	FAM90A1
BRD4	FAM90A1
CUL2	FAM98A
GABARAPL2	FAM98A
RTRAF	FAM98A
CUL1	FAM98A
FUS	FAM98A
YBX1	FAM98A
CAND1	FAM98A
MCM2	FAM98A
CUL3	FAM98A
BCKDK	FAM98B
MOV10	FAM98B
AP2A2	FAN1
POR	FANCC
PIGS	FANCD2
MOV10	FANCM
G3BP2	FARS2
ECSIT	FARSA
AP2M1	FASN
MRPS2	FASTKD3
RAB14	FASTKD3
PMPCB	FASTKD3
NLRX1	FASTKD5
TUBG1	FASTKD5
CCDC85B	FASTKD5
SIRT4	FASTKD5
MASTL	FASTKD5
NTRK1	FASTKD5
HERC2	FASTKD5
PPP4R1	FASTKD5
NCSTN	FASTKD5
MAPK4	FASTKD5
ATL3	FASTKD5
UNC93B1	FASTKD5
IRAK2	FASTKD5
FBXO6	FASTKD5
POU5F1	FASTKD5

CUL2	FBF1
GOLGA2	FBF1
GCC1	FBF1
MIPOL1	FBF1
TLE5	FBF1
RBX1	FBH1
RALA	FBH1
TFAP2C	FBLN5
ATN1	FBLN5
TOP3B	FBLN5
LOXL1	FBLN5
FBN1	FBN1
RHOB	FBN1
FBLN2	FBN1
SPRY2	FBN1
MYOC	FBN1
MFAP2	FBN1
ELN	FBN1
ATXN7	FBN1
VCAN	FBN1
AURKA	FBN2
FBXO6	FBN2
ELN	FBN2
MATN2	FBN2
SFRP4	FBN2
CSNK2A2	FBRS
CSNK2A2	FBRSL1
RBX1	FBXL12
PSEN1	FBXL12
UBE2D3	FBXL12
TTC27	FBXL12
RNH1	FBXL12
CAPN1	FBXL12
GSTP1	FBXL12
FXYD3	FBXL12
ALDH3A1	FBXL12
MAST1	FBXL12
LNX1	FBXL12
ALDH3B1	FBXL12
DOCK8	FBXL12
RNF32	FBXL12
CUL1	FBXL12
HSP90AB1	FBXL12
RCN1	FBXL12
HSP90AA1	FBXL12
CDC37	FBXL12
SKP1	FBXL12
ALDH3A2	FBXL12
RBX1	FBXL15
MARK2	FBXL16
CUL2	FBXL2
ERLEC1	FBXL4
RBX1	FBXL5
RBX1	FBXO11
OS9	FBXO15
RBX1	FBXO17
RBX1	FBXO2
ERO1B	FBXO2
RBX1	FBXO21
RBX1	FBXO22
CDK5RAP2	FBXO25

RBX1	FBXO25
RBX1	FBXO27
RBX1	FBXO3
RBX1	FBXO31
RBX1	FBXO44
MYCBP2	FBXO45
NUP98	FBXO6
PABPC1	FBXO6
FOXRED2	FBXO6
GLA	FBXO6
PIGS	FBXO6
UGGT2	FBXO6
RBX1	FBXO6
DNAJC11	FBXO6
ATP6AP1	FBXO6
ERO1B	FBXO6
POFUT1	FBXO6
COMT	FBXO6
GGCX	FBXO6
PLD3	FBXO6
SCARB1	FBXO6
CHPF2	FBXO6
EDEM3	FBXO6
ATP6V1A	FBXO6
ERLEC1	FBXO6
PRKACA	FBXW11
AKAP8L	FBXW11
PABPC1	FBXW11
RBX1	FBXW2
HECTD1	FBXW7
MYCBP2	FBXW7
RAE1	FBXW7
RBM28	FBXW7
RBX1	FBXW7
CUL2	FBXW8
RBX1	FBXW8
GORASP1	FBXW8
EXOSC5	FCHO1
MOV10	FCHSD1
MOV10	FDPS
CUL2	FEM1A
ELOB	FEM1B
CUL2	FEM1B
RBX1	FEM1B
ELOC	FEM1B
CUL2	FEM1C
MOV10	FEN1
CHPF2	FEN1
MRPS27	FERMT3
EIF4E2	FERMT3
PDE4DIP	FES
NDUFB9	FEZ1
PLAT	FGA
CSNK2A2	FGF1
COMT	FGF2
CSNK2A2	FGF2
PRKAR2A	FGFR1OP
MIB1	FGFR1OP
CEP350	FGFR1OP
TIMM29	FGFR1OP
CDK5RAP2	FGFR1OP

TLE5	FGFR1OP
AKAP9	FGFR1OP
CEP135	FGFR1OP
POLA2	FGFR3
PLAT	FGL1
LARP4B	FHL3
PLEKHA5	FHL3
PLEKHF2	FHL3
EXOSC8	FHOD1
MCM2	FKBP15
NUDT12	FKBP15
RPA3	FKBP15
RHOU	FKBP15
SNAP29	FKBP15
RPA2	FKBP15
XPO1	FKBP15
FKBP7	FKBP7
HGF	FKBP7
AAR2	FKBPL
TARS2	FKBPL
MOV10	FLAD1
CUL2	FLG
CUL2	FLG2
G3BP1	FLNA
AP2M1	FLNA
ITGB1	FLNA
FOXRED2	FLNA
RALA	FLNA
RALA	FLNB
RAB2A	FLOT1
RAB7A	FLOT1
ATP6V1A	FLOT1
RAB5C	FLOT1
RAB2A	FLOT2
RAB5C	FLOT2
RAB7A	FLOT2
STOM	FMNL1
RAE1	FN1
RAB7A	FN1
RAB5C	FN1
COMT	FN1
EIF4H	FN1
MDN1	FN1
PABPC4	FN1
PABPC1	FN1
RAB10	FN1
PLAT	FN1
UPF1	FN1
AKAP9	FNBP1
USP13	FNBP1L
CENPF	FNTB
CUL2	FOS
TLE3	FOXA3
STOM	FOXA3
AKAP8	FOXA3
AKAP8	FOXC2
FYCO1	FOXK1
STOM	FOXL1
NUP98	FOXL1
MDN1	FOXL1
TOMM70	FOXL1

MAP7D1	FOXL1
TLE3	FOXL1
PSMD8	FOXN2
TLE5	FOXP2
NUP98	FOXQ1
LARP1	FOXQ1
CCDC86	FOXQ1
TLE3	FOXQ1
MOV10	FOXQ1
ERLEC1	FOXRED2
SEL1L	FOXRED2
GANAB	FOXRED2
USH2A	FOXRED2
TXNDC16	FOXRED2
DNAJC10	FOXRED2
AKAP8	FOXS1
DCAKD	FOXS1
BAG5	FRMD5
TLE5	FRS3
MARK2	FRYL
POLA2	FRZB
RBX1	FRZB
MIB1	FSD1
TLE5	FSD2
ATP6V1A	FTH1
RHOA	FUBP3
MARK2	FUCA1
MOV10	FUNDC2
USP13	FUNDC2
ERP44	FUS
RAB7A	FUS
BCS1L	FUS
PMPCB	FXN
RTN4	FXR2
ATP1B1	FXYD1
FYCO1	FYCO1
MOV10	FYCO1
MAP1LC3A	FYCO1
POLDIP3	FYCO1
KIF23	FYCO1
APP	FYCO1
LMNA	FYCO1
CCT3	FYCO1
MAP1LC3B	FYCO1
RPA1	FYCO1
CCT6B	FYCO1
GABARAPL1	FYCO1
MLYCD	FYCO1
PPL	FYCO1
EMD	FYCO1
XPO1	FYCO1
GABARAPL2	FYCO1
DDX60	FYCO1
CCT6A	FYCO1
NOL9	FYCO1
CCAR1	FYCO1
SEC24B	FYCO1
TMPO	FYCO1
CCT7	FYCO1
KDM1A	FYCO1
RPA2	FYCO1

CCZ1	FYCO1
TCP1	FYCO1
RPA3	FYCO1
DTNA	FYCO1
MON1B	FYCO1
GOPC	FYCO1
RMC1	FYCO1
SASS6	FYCO1
RAF1	FYCO1
MAP4	FYCO1
SEC23B	FYCO1
NINL	FZR1
G3BP1	G3BP1
PABPC1	G3BP1
G3BP2	G3BP1
NUP214	G3BP1
CENPF	G3BP1
CSK	G3BP1
CAPRIN1	G3BP1
CROCC	G3BP1
WDR36	G3BP1
CAND1	G3BP1
TP53	G3BP1
GABARAPL1	G3BP1
HAT1	G3BP1
FLNB	G3BP1
CUL1	G3BP1
SIRT6	G3BP1
YBX1	G3BP1
PES1	G3BP1
IFIH1	G3BP1
UTP18	G3BP1
GNL2	G3BP1
ITGA4	G3BP1
ARRB1	G3BP1
FN1	G3BP1
KRI1	G3BP1
CTSC	G3BP1
MPP1	G3BP1
NUP188	G3BP1
DTX1	G3BP1
PIN1	G3BP1
CTNNBL1	G3BP1
BCL7C	G3BP1
CUL3	G3BP1
RBM22	G3BP1
HDAC6	G3BP1
BLMH	G3BP1
USP10	G3BP1
RASA1	G3BP1
RPAP2	G3BP1
HNRNPA1	G3BP1
EIF4G1	G3BP1
FXR2	G3BP1
EMD	G3BP1
DHX40	G3BP1
RIOK2	G3BP1
UIMC1	G3BP1
TARDBP	G3BP1
RPTOR	G3BP1
PDPK1	G3BP1

PRKAA1	G3BP1
RNF2	G3BP1
CLNS1A	G3BP1
ESR1	G3BP1
ELAVL1	G3BP1
ASCC3	G3BP1
APP	G3BP1
COPS5	G3BP1
MCM2	G3BP1
GABARAPL2	G3BP1
NUFIP2	G3BP1
CSNK1G2	G3BP1
NUP214	G3BP2
UBAP2	G3BP2
NUP88	G3BP2
ASCC3	G3BP2
NUFIP2	G3BP2
GNL2	G3BP2
CUL1	G3BP2
DDX24	G3BP2
CUL3	G3BP2
GABARAPL2	G3BP2
TERF2	G3BP2
TARDBP	G3BP2
FXR2	G3BP2
USP10	G3BP2
CDK2	G3BP2
STK24	G3BP2
DDX6	G3BP2
PIN1	G3BP2
WDR36	G3BP2
CAND1	G3BP2
ELAVL1	G3BP2
COPS5	G3BP2
EIF1B	G3BP2
ESR1	G3BP2
MDM2	G3BP2
UIMC1	G3BP2
HNRNPA1	G3BP2
RNF2	G3BP2
ING4	G3BP2
RNH1	G3BP2
SNW1	G3BP2
IVNS1ABP	G3BP2
NFKBIA	G3BP2
NUP153	G3BP2
MARK2	GAB1
PABPC4	GABARAPL1
RPL36	GABARAPL1
NUP210	GABARAPL1
RAB7A	GABARAPL1
PABPC1	GABARAPL1
NUP214	GABARAPL1
RAB1A	GABARAPL1
NUP88	GABARAPL1
NEK9	GABARAPL1
RDX	GABARAPL1
ACADM	GABARAPL1
JAKMIP1	GABBR1
PRKACA	GABRB3
ERMP1	GABRE

RAB5C	GADD45GIP1
GOLGB1	GAK
AP2M1	GAK
BAG5	GAK
FOXRED2	GALK1
MOV10	GALNT10
EXOSC2	GALNT13
REEP5	GALNT2
NINL	GAN
RBX1	GAN
PABPC1	GAN
PCNT	GAN
PABPC4	GAN
CUL2	GAPDH
GOLGA2	GATA2
RBX1	GATA2
DNMT1	GATA4
MOV10	GATAD1
HYOU1	GBA
ATP6V1A	GBA
RAB5C	GCA
NINL	GCC1
GOLGA2	GCC1
CPSF7	GCC1
TRIM29	GCC1
ATF4	GCC1
SMARCD1	GCC1
TINF2	GCC1
SPOP	GCC1
NAB2	GCC1
TFIP11	GCC1
LZTS2	GCC1
DNAJA3	GCC1
CEP57	GCC1
NAV1	GCC1
XRCC1	GCC1
ATN1	GCC1
BIRC2	GCC1
LNPEP	GCC1
BRCA1	GCC1
USHBP1	GCC1
OIP5	GCC1
CCDC85B	GCC1
CEP70	GCC1
KIAA1217	GCC1
ZNF276	GCC1
COIL	GCC1
GLI1	GCC1
APP	GCC1
NECAB2	GCC1
LLGL2	GCC1
TMEM132A	GCC1
ABI2	GCC1
ARL1	GCC1
SMC1A	GCC1
AMOTL2	GCC1
GCC2	GCC2
HERC2	GCC2
ZRANB1	GCC2
ARL1	GCC2
KIFAP3	GCC2

PRKAR2A	GCH1
PRKAR2B	GCH1
ERLEC1	GDF11
GDF15	GDF15
STAT5A	GDF15
MDFI	GDF15
MAPK14	GDF15
NDUFB9	GDF9
RAB5C	GDI1
RAB10	GDI1
RAB1A	GDI1
RAB14	GDI1
FAM98A	GDI1
RAB7A	GDI1
RAB8A	GDI1
HECTD1	GEM
NSD2	GEMIN4
SRP54	GEMIN4
MIB1	GEMIN4
F2RL1	GEMIN4
USP13	GET4
MOV10	GET4
TLE5	GFAP
GFER	GFER
COPS5	GFER
ASCC2	GFER
KLHL20	GFER
AMMECR1	GFER
TLE5	GFI1B
FBLN5	GFI1B
ECSIT	GFM1
ERC1	GFOD1
TENT5A	GGCX
FGD1	GGCX
OS9	GGH
GGH	GGH
ATP6V1A	GGH
MCM2	GGH
MKLN1	GGH
DCAF8	GGH
COPS5	GGH
UGDH	GGH
TXNDC16	GGH
FBXO6	GGH
ATP6V1B1	GGH
CUL2	GH1
ERLEC1	GH1
DDX21	GH1
CSDE1	GH1
CSNK2B	GH1
AAR2	GHDC
NT5E	GHITM
UNK	GHITM
SARM1	GHITM
LYPD3	GHITM
FNDC4	GHITM
B4GALT3	GHITM
TOR1B	GHITM
GABRE	GHITM
HSPA5	GHITM
GCNT2	GHITM

EIF4E2	GIGYF1
CEP250	GIGYF1
EIF4E2	GIGYF2
PABPC1	GIGYF2
CEP250	GIGYF2
TUBGCP3	GIGYF2
G3BP1	GIGYF2
CAPRIN1	GIGYF2
DIS3L	GIGYF2
CEP57	GIGYF2
MAGED2	GIGYF2
ATN1	GIGYF2
CNOT9	GIGYF2
HUWE1	GIGYF2
SNRPC	GIGYF2
AURKB	GIGYF2
YWHAB	GIGYF2
DDX20	GIGYF2
RFX5	GIGYF2
HSP90AA1	GIGYF2
XPO1	GIGYF2
MCM2	GIGYF2
PRPF8	GIGYF2
SMN1	GIGYF2
LGALS3BP	GIGYF2
AGO2	GIGYF2
SNRPN	GIGYF2
TNRC6B	GIGYF2
FXR1	GIGYF2
SF3B2	GIGYF2
GEMIN4	GIGYF2
OTUB1	GIGYF2
ZNF598	GIGYF2
ELAVL1	GIGYF2
GRB10	GIGYF2
TARDBP	GIGYF2
CCDC8	GIGYF2
WBP11	GIGYF2
PRPF6	GIGYF2
WDR83	GIGYF2
CEP76	GIGYF2
U2AF2	GIGYF2
USP11	GIGYF2
VCP	GIGYF2
EXOSC7	GIGYF2
SIRT7	GIGYF2
EIF4A2	GIGYF2
CDKN1A	GIGYF2
U2AF1L4	GIGYF2
APC	GIGYF2
NTRK1	GIGYF2
SNRPB	GIGYF2
TP53	GIGYF2
EMD	GIGYF2
MEX3C	GIGYF2
BMI1	GIGYF2
YBX1	GIGYF2
PCK1	GIGYF2
PLEKHA5	GINM1
POLA1	GINS4
MOV10	GIPC3

MEPCE	GLB1
CUL2	GLDC
GNB1	GLI1
RBX1	GLMN
CUL2	GLMN
RALA	GLS2
MOV10	GLUD2
GOLGA2	GLYCTK
PRKAR2A	GNA13
GNB1	GNAI1
GNB1	GNAI2
PRKAR2B	GNAI3
PPT1	GNAQ
GNB1	GNAS
NUDCD3	GNB1
BTK	GNB1
ELAVL1	GNB1
HDAC4	GNB1
FYN	GNB1
TBXA2R	GNB1
GNAI3	GNB1
RAB7A	GNB2
GNB1	GNG10
GNB1	GNG11
GNB1	GNG12
GNB1	GNG2
GNB1	GNG5
GOLGA2	GNG5
GNAI3	GNG5
GNB2	GNG5
GNB4	GNG5
GNB3	GNG5
MDFI	GNG5
AMOTL2	GNG5
GNAI1	GNG5
ABCE1	GNG5
NFKBIA	GNG5
FSD1	GNG5
GNB5	GNG5
GNAI2	GNG5
FYTTD1	GNG5
PPP1R3C	GNG5
EPS15L1	GNG5
TRAF1	GNG5
GNAS	GNG5
GNB1	GNG7
GNB1	GNGT2
EXOSC5	GNL3L
AGPS	GNPAT
UPF1	GNPTG
EXOSC5	GOLGA2
SLU7	GOLGA2
RAB1A	GOLGA2
TLE5	GOLGA2
RBM41	GOLGA2
RAB2A	GOLGA2
PTBP2	GOLGA2
GORASP1	GOLGA2
PARD6B	GOLGA2
LMO2	GOLGA2
BYSL	GOLGA2

NXT2	GOLGA2
IFT27	GOLGA2
VPS28	GOLGA2
CCDC146	GOLGA2
MCM7	GOLGA2
STX12	GOLGA2
GOLT1B	GOLGA2
TXLNA	GOLGA2
SNRPB	GOLGA2
MVP	GOLGA2
TTC23	GOLGA2
CYB5R2	GOLGA2
ARPC3	GOLGA2
CCNH	GOLGA2
TRAF3IP2	GOLGA2
BARD1	GOLGA2
CHCHD3	GOLGA2
ISCU	GOLGA2
MID2	GOLGA2
GSE1	GOLGA2
ZFC3H1	GOLGA2
CSPP1	GOLGA2
C2CD6	GOLGA2
CHCHD2	GOLGA2
CINP	GOLGA2
BCL6B	GOLGA2
SEC16A	GOLGA2
IQCE	GOLGA2
CDC73	GOLGA2
XPO1	GOLGA2
KIFC3	GOLGA2
WASHC3	GOLGA2
C1orf35	GOLGA2
CDKL3	GOLGA2
ZBTB16	GOLGA2
TSC22D4	GOLGA2
TPX2	GOLGA2
FAM184A	GOLGA2
AMOTL2	GOLGA2
PRKAA1	GOLGA2
ERCC3	GOLGA2
RITA1	GOLGA2
SNRPB2	GOLGA2
SNAP47	GOLGA2
FAM214A	GOLGA2
STX7	GOLGA2
KRT18	GOLGA2
LIN7A	GOLGA2
STK26	GOLGA2
CDC5L	GOLGA2
ATP6V1D	GOLGA2
DMTN	GOLGA2
MTFR2	GOLGA2
RSPH14	GOLGA2
PID1	GOLGA2
SPATA2	GOLGA2
LASP1	GOLGA2
GORASP2	GOLGA2
C1orf109	GOLGA2
BAHD1	GOLGA2
FBXL18	GOLGA2

GTPBP10	GOLGA2
PSMA4	GOLGA2
MORF4L2	GOLGA2
SH2D4A	GOLGA2
RSRC2	GOLGA2
LENG1	GOLGA2
ZMAT2	GOLGA2
TSSK3	GOLGA2
CCDC17	GOLGA2
ATXN7	GOLGA2
UBE2I	GOLGA2
AFF4	GOLGA2
PRKAB2	GOLGA2
PRPF31	GOLGA2
NCF2	GOLGA2
GFAP	GOLGA2
DLGAP5	GOLGA2
SHC3	GOLGA2
CCNC	GOLGA2
TMED2	GOLGA2
EIF4A2	GOLGA2
FNDC11	GOLGA2
RAB2B	GOLGA2
FZD1	GOLGA2
ESR1	GOLGA2
ZNF410	GOLGA2
LYPLA1	GOLGA2
TCF19	GOLGA2
HAUS1	GOLGA2
RASSF8	GOLGA2
USO1	GOLGA2
ARL4A	GOLGA2
GEM	GOLGA2
LMO4	GOLGA2
NES	GOLGA2
RAB39B	GOLGA2
NME7	GOLGA2
DCLRE1C	GOLGA2
SNW1	GOLGA2
MORN3	GOLGA2
FAM50B	GOLGA2
DTX2	GOLGA2
LYSMD1	GOLGA2
CDK18	GOLGA2
RBM17	GOLGA2
GATAD2B	GOLGA2
MCM10	GOLGA2
STK25	GOLGA2
USP2	GOLGA2
LGALS3	GOLGA2
PQBP1	GOLGA2
MYEF2	GOLGA2
SNF8	GOLGA2
FAM126B	GOLGA2
PPP1R18	GOLGA2
NOS3	GOLGA2
CORO1A	GOLGA2
SAMD4A	GOLGA2
TPM1	GOLGA2
BCAS2	GOLGA2
FAM193B	GOLGA2

ATP6V1C2	GOLGA2
FAM110A	GOLGA2
LATS1	GOLGA2
ABHD17A	GOLGA2
RBM39	GOLGA2
CCDC150	GOLGA2
WDYHV1	GOLGA2
KIAA1217	GOLGA2
TBP	GOLGA2
MAGOHB	GOLGA2
ALKBH3	GOLGA2
MBD3	GOLGA2
SH3RF2	GOLGA2
PKN1	GOLGA2
PATL1	GOLGA2
TRIM29	GOLGA2
CRMP1	GOLGA2
TFAP4	GOLGA2
EP300	GOLGA2
CCAR1	GOLGA2
ORC1	GOLGA2
PSMA1	GOLGA2
ZNF414	GOLGA2
LCP2	GOLGA2
PIAS2	GOLGA2
TMEM132A	GOLGA2
POLDIP3	GOLGA2
THYN1	GOLGA2
PIN1	GOLGA2
ZFYVE26	GOLGA2
KDM1A	GOLGA2
HDAC4	GOLGA2
SCNM1	GOLGA2
DCTN4	GOLGA2
PPP1R16B	GOLGA2
CDK2	GOLGA2
NDEL1	GOLGA2
LIMS2	GOLGA2
GPKOW	GOLGA2
APC	GOLGA2
RCOR3	GOLGA2
TCL1A	GOLGA2
FAM124B	GOLGA2
ITPKB	GOLGA2
NUDT21	GOLGA2
FAM207A	GOLGA2
NDE1	GOLGA2
RIBC1	GOLGA2
FIP1L1	GOLGA2
STAMBPL1	GOLGA2
DDX6	GOLGA2
STAU1	GOLGA2
MFAP1	GOLGA2
CBX8	GOLGA2
ZG16B	GOLGA2
SNRPC	GOLGA2
IFT20	GOLGA2
YJU2	GOLGA2
EIF3G	GOLGA2
FBXO28	GOLGA2
ZMAT1	GOLGA2

MAGOH	GOLGA2
KANSL1	GOLGA2
SAP30BP	GOLGA2
TTC9C	GOLGA2
YTHDC1	GOLGA2
TUBGCP4	GOLGA2
UBE3C	GOLGA2
ADAP1	GOLGA2
GTSE1	GOLGA2
NEBL	GOLGA2
ABLIM1	GOLGA2
RBL1	GOLGA2
PKP4	GOLGA2
SMARCE1	GOLGA2
ATP6V1G1	GOLGA2
SCEL	GOLGA2
CUL5	GOLGA2
GOLGA3	GOLGA3
TRAF1	GOLGA3
ELAVL1	GOLGA3
GOLGA3	GOLGA7
CLDND1	GOLGA7
TSPAN3	GOLGA7
SUN2	GOLGA7B
CEP126	GOLGB1
USO1	GOLGB1
CCDC93	GOLGB1
CAV2	GOLGB1
MAPK7	GOLGB1
SKIL	GOLGB1
PFN2	GOLGB1
SLC2A3	GOLGB1
TMED10	GOLGB1
DYNLL1	GOLGB1
VTI1B	GOLGB1
OBSL1	GOLGB1
CCDC8	GOLGB1
CUL7	GOLGB1
ECPAS	GOLGB1
NDUFA1	GOLGB1
UBE2I	GOLGB1
CDC5L	GOLGB1
SUN2	GOLT1B
DNAJC11	GOLT1B
RAB5C	GOLT1B
CHPF2	GOLT1B
ATP6AP1	GOLT1B
AGPS	GORASP1
GORASP1	GORASP1
PRKD2	GORASP1
XPO1	GORASP1
TMED2	GORASP1
STX7	GORASP1
HIPK3	GORASP1
MAPK6	GORASP1
RAB2A	GORASP2
TLE5	GORASP2
PIGS	GPAA1
MCOLN3	GPAA1
HTR3C	GPAA1
GRIK5	GPAA1

LYPD3	GPAA1
PIN1	GPAA1
PIGT	GPAA1
EIF3E	GPAA1
GABRE	GPAA1
PIGK	GPAA1
GOLGA2	GPANK1
MOV10	GPAT4
CSNK2A2	GPATCH2
CSNK2A2	GPATCH2L
SNIP1	GPATCH8
TIMM8B	GPATCH8
CSNK2A2	GPC4
ERMP1	GPM6A
PRKACA	GPR161
BZW2	GPR183
REEP5	GPR84
RETREG3	GPR89B
MARK2	GPRASP1
MAP7D1	GPRASP1
CUL2	GPS1
GFER	GPS1
RBX1	GPS1
BRD4	GPSM1
RALA	GPT
MAPK6	GPX1
SELENBP1	GPX1
ABL1	GPX1
SIRT4	GPX1
ABL2	GPX1
TLE5	GRB2
RPL36	GRB2
PABPC1	GRB2
AP2M1	GRB2
GRIPAP1	GRIP1
GNB1	GRK2
MOV10	GRK2
LARP1	GRK5
MRPS27	GRK5
RBX1	GRK5
MOV10	GRK5
AP2A2	GRK5
AP3B1	GRK5
DDX21	GRK5
GRPEL1	GRPEL1
CCAR1	GRPEL1
GRPEL1	GRPEL2
CUL2	GRSF1
MRPS25	GRSF1
MRPS27	GRSF1
MRPS2	GRSF1
PRKACA	GSK3A
PRKACA	GSK3B
UPF1	GSPT1
PABPC1	GSPT1
UPF1	GSPT2
PABPC1	GSPT2
HEATR3	GSTK1
DNAJC11	GSTK1
PKP2	GTF2B
TLE5	GTF2E1

GTF2F2	GTF2E2
MOV10	GTF2E2
TAF5	GTF2F2
MSX2	GTF2F2
HMGA1	GTF2F2
POLR2C	GTF2F2
TBP	GTF2F2
JUN	GTF2F2
POLR2A	GTF2F2
POLR2B	GTF2F2
KLF5	GTF2F2
MED29	GTF2F2
HECW2	GTF2F2
POLR2F	GTF2F2
FAM177A1	GTF2F2
TCEA1	GTF2F2
TAF4	GTF2F2
AHR	GTF2F2
GTF2E1	GTF2F2
POLR2E	GTF2F2
CTDP1	GTF2F2
RPL27A	GTF2F2
TAF10	GTF2F2
POLR2J	GTF2F2
POLR2G	GTF2F2
FOS	GTF2F2
POLR2H	GTF2F2
GTF2F1	GTF2F2
HTATSF1	GTF2F2
ARNT	GTF2F2
AR	GTF2F2
XPO1	GTF2F2
GTF2B	GTF2F2
ATF4	GTF2F2
NUP98	GTF2H5
CEP250	GTF3C5
CSNK2A2	GTPBP4
PRKACA	GTSE1
PKP2	GTSE1
TMEM97	GUK1
CEP250	H1-10
CSNK2A2	H1-2
RBX1	H1-2
CEP250	H1-2
CUL2	H1-2
DNMT1	H2AC20
PTGES2	H2AX
POFUT1	H2AX
PABPC1	H2AX
FKBP10	H2AX
HYOU1	H2AX
TOR1A	H2AX
DDX21	H2AX
DNMT1	H2AX
LOX	H2BC21
CUL2	H2BC21
RAB5C	H3-3A
DNAJC11	H3-3A
DNAJC11	H3-5
DNAJC11	H3C4
CUL2	H4-16

STC2	H4-16
STC2	H4C8
CUL2	H4C8
CEP250	H4C8
CSNK2A2	HADHA
CSNK2A2	HADHB
CSNK2A2	HAGHL
NDUFB9	HAP1
CDK5RAP2	HAP1
MOV10	HARS1
PABPC1	HAUS1
MIPOL1	HAUS1
NINL	HAUS1
CEP250	HAUS1
NUP54	HAUS1
MOGS	HAUS2
MRPS27	HAUS2
RAB5C	HAUS5
CEP250	HAUS5
SLC27A2	HAUS7
SCCPDH	HAUS7
RAB14	HAX1
RAB2A	HAX1
DNAJC11	HAX1
TUBGCP3	HAX1
TUBGCP2	HAX1
RBX1	HAX1
CEP250	HAX1
CEP250	HBA1
ATP6AP1	HBA1
ATP6AP1	HBA2
PABPC1	HBB
CEP250	HBB
UPF1	HBB
PRKAR2B	HBP1
EXOSC3	HBS1L
NSD2	HDAC1
NUP98	HDAC1
AGPS	HDAC11
RBM28	HDAC11
MYCBP2	HDAC11
MDN1	HDAC11
AKAP8	HDAC11
AKAP8L	HDAC11
NUP98	HDAC11
DNMT1	HDAC2
NSD2	HDAC2
HDAC2	HDAC2
MOV10	HDAC2
ZNF318	HDAC2
BRCA1	HDAC2
PARP14	HDAC2
SUZ12	HDAC2
SNAI3	HDAC2
FOXK1	HDAC2
PIAS4	HDAC2
PHF21A	HDAC2
EED	HDAC2
IPO5	HDAC2
USP4	HDAC2
SOX2	HDAC2

RNF2	HDAC2
CCNA2	HDAC2
ZEB2	HDAC2
ENO1	HDAC2
RUNX1	HDAC2
NFATC1	HDAC2
MXD1	HDAC2
MIER3	HDAC2
CIAO1	HDAC2
TWIST1	HDAC2
VHL	HDAC2
CSNK2A1	HDAC2
DNTTIP1	HDAC2
CREB1	HDAC2
CABIN1	HDAC2
MTA2	HDAC2
CCDC8	HDAC2
SAP30L	HDAC2
HCFC1	HDAC2
DEK	HDAC2
PADI4	HDAC2
SYK	HDAC2
PRDM4	HDAC2
RCOR2	HDAC2
SMAD2	HDAC2
DDB1	HDAC2
STAT3	HDAC2
TOP2A	HDAC2
ZNF217	HDAC2
RBL1	HDAC2
HUWE1	HDAC2
MIER2	HDAC2
ESR1	HDAC2
CBFA2T3	HDAC2
PHF12	HDAC2
PHC2	HDAC2
NCOR2	HDAC2
RLIM	HDAC2
SENP1	HDAC2
BCL11B	HDAC2
SIRT1	HDAC2
SUDS3	HDAC2
ZBTB7A	HDAC2
GLI1	HDAC2
ZMYM3	HDAC2
BRMS1L	HDAC2
NPM1	HDAC2
SMN1	HDAC2
LMO4	HDAC2
SIRT6	HDAC2
SMARCA4	HDAC2
SIRT7	HDAC2
MDM2	HDAC2
IRS1	HDAC2
HSPA4	HDAC2
KPNA1	HDAC2
GATA4	HDAC2
PPP1R8	HDAC2
TAF6	HDAC2
FOXL1	HDAC2
MTA3	HDAC2

IKZF3	HDAC2
RELA	HDAC2
CDH1	HDAC2
RBPJ	HDAC2
FOXC2	HDAC2
GATAD2B	HDAC2
AURKA	HDAC2
IKZF1	HDAC2
MBD3	HDAC2
NXF1	HDAC2
TBX3	HDAC2
CUL4B	HDAC2
TAL1	HDAC2
YY1	HDAC2
ZMYND8	HDAC2
UNK	HDAC2
CIR1	HDAC2
KDM1A	HDAC2
ZFP1	HDAC2
GATA3	HDAC2
SS18	HDAC2
FKBP3	HDAC2
CTBP2	HDAC2
RFX5	HDAC2
SUV39H1	HDAC2
GATAD2A	HDAC2
MBTPS1	HDAC2
ZNF219	HDAC2
SS18L1	HDAC2
BUB3	HDAC2
PPP6R3	HDAC2
CTBP1	HDAC2
CCND1	HDAC2
HDAC7	HDAC2
SIN3A	HDAC2
ARRB2	HDAC2
DDX5	HDAC2
HIF1A	HDAC2
NR2C1	HDAC2
SIN3B	HDAC2
SP3	HDAC2
SMARCA5	HDAC2
PTMA	HDAC2
MBD2	HDAC2
EZH2	HDAC2
OBSL1	HDAC2
FOXS1	HDAC2
SNAI2	HDAC2
CDKN1A	HDAC2
JARID2	HDAC2
ING1	HDAC2
MORF4L2	HDAC2
SMARCAD1	HDAC2
GATAD1	HDAC2
PARP1	HDAC2
GFI1	HDAC2
ARID5B	HDAC2
TP53	HDAC2
CHD3	HDAC2
HR	HDAC2
SRRM2	HDAC2

RREB1	HDAC2
NFKBIA	HDAC2
KLF4	HDAC2
RBBP4	HDAC2
TCF3	HDAC2
REST	HDAC2
ARID4B	HDAC2
RCOR3	HDAC2
BAHD1	HDAC2
NFKB1	HDAC2
NARS1	HDAC2
GSE1	HDAC2
SFPQ	HDAC2
MYC	HDAC2
IKZF4	HDAC2
SMARCA2	HDAC2
UBE2E2	HDAC2
CDK2AP2	HDAC2
GFI1B	HDAC2
BANF1	HDAC2
SAP130	HDAC2
NR3C1	HDAC2
ZBTB16	HDAC2
CREBBP	HDAC2
ELMSAN1	HDAC2
PRDM6	HDAC2
PSMA3	HDAC2
SERPINH1	HDAC2
DDX17	HDAC2
APEX1	HDAC2
FOXK2	HDAC2
SNRNP70	HDAC2
KDM5A	HDAC2
CHFR	HDAC2
ZFPM2	HDAC2
RB1	HDAC2
KDM5B	HDAC2
ATR	HDAC2
ING2	HDAC2
MPP1	HDAC2
TSC22D3	HDAC2
RBBP7	HDAC2
FOXP3	HDAC2
NRIP1	HDAC2
MEN1	HDAC2
BCL6	HDAC2
CUL7	HDAC2
UBE2H	HDAC2
CDK2AP1	HDAC2
ESCO1	HDAC2
BRMS1	HDAC2
APPL1	HDAC2
NACC2	HDAC2
KMT2A	HDAC2
TFCP2	HDAC2
MAD1L1	HDAC2
DCAF1	HDAC2
CIITA	HDAC2
CHD4	HDAC2
PARK7	HDAC2
ACTL6A	HDAC2

EMSY	HDAC2
SUMO2	HDAC2
YAF2	HDAC2
KDM5C	HDAC2
SP1	HDAC2
RCOR1	HDAC2
RPL10	HDAC2
NFE2L2	HDAC2
CDYL	HDAC2
HIF1AN	HDAC2
RERE	HDAC2
SMAD7	HDAC2
KLF5	HDAC2
TOP2B	HDAC2
AIM2	HDAC2
SNAI1	HDAC2
TFAP4	HDAC2
MACROH2A2	HDAC2
ELAVL1	HDAC2
CHD5	HDAC2
HDGF	HDAC2
WIZ	HDAC2
SAP30	HDAC2
PML	HDAC2
C16orf87	HDAC2
SMARCC2	HDAC2
MORF4L1	HDAC2
MACROH2A1	HDAC2
SP6	HDAC2
EID2	HDAC2
PPARD	HDAC2
BCL11A	HDAC2
OVOL1	HDAC2
HOPX	HDAC2
SMARCE1	HDAC2
CDK7	HDAC2
HMG20B	HDAC2
RARA	HDAC2
TP73	HDAC2
PELP1	HDAC2
PHC1	HDAC2
EHMT1	HDAC2
ZMYM2	HDAC2
IKZF2	HDAC2
HDAC1	HDAC2
MTA1	HDAC2
FOXJ2	HDAC2
HELLS	HDAC2
PA2G4	HDAC2
PRDM1	HDAC2
RUNX1T1	HDAC2
FOXA3	HDAC2
DNAJA1	HDAC2
SETDB1	HDAC2
HMG20A	HDAC2
SNW1	HDAC2
ESCO2	HDAC2
MYCN	HDAC2
DNMT3B	HDAC2
HSF1	HDAC2
RUVBL2	HDAC2

NONO	HDAC2
CBX7	HDAC2
CDC20	HDAC2
MAF	HDAC2
DNMT1	HDAC3
UPF1	HDAC5
MARK3	HDAC5
GNB1	HDAC5
PABPC1	HDAC5
POLA2	HDAC6
CSNK2A2	HDAC6
NUP98	HDAC8
EXOSC3	HDGF
EXOSC2	HDGF
MRPS27	HDGF
CSNK2A2	HDGFL2
SLC30A6	HDGFL3
SURF2	HEATR3
LYPD3	HEATR3
PTGER3	HEATR3
PNKD	HEATR3
B3GAT3	HEATR3
DSP	HEATR3
NCAPH	HEATR3
ELAVL1	HEATR3
HNRNPD	HEATR3
EGFR	HEATR3
SMS	HEATR3
LNPEP	HEATR3
SCN2B	HEATR3
MYC	HEATR3
CSDE1	HEATR5A
ESR1	HECTD1
NCAPH2	HECTD1
RIOK2	HECTD1
ZRANB1	HECTD1
FBXW11	HECTD1
NCAPD2	HECTD1
CIT	HECW2
MAP7D1	HECW2
TUBGCP2	HECW2
RRP9	HECW2
TUBGCP3	HECW2
CENPF	HECW2
RBM28	HECW2
MARK1	HECW2
SIRT5	HECW2
LARP4B	HECW2
POLA1	HELB
POLA2	HELB
PRKAR2B	HERC2
AKAP9	HERC2
CEP250	HERC2
MARK2	HERC2
NIN	HERC2
SLC30A9	HERC2
MOV10	HEXIM1
MEPCE	HEXIM1
LARP7	HEXIM1
CSNK2A2	HEXIM1
BRD4	HEXIM1

TCF12	HEXIM2
MEPCE	HEXIM2
MYCBP2	HEY1
FBXL12	HGH1
POLA2	HGH1
MOV10	HGS
NUP54	HGS
CIT	HGS
PLD3	HGS
SUN2	HGS
INTS4	HGS
CSDE1	HID1
RBX1	HIF1A
PLEKHA5	HIF1A
COMT	HIGD1A
MOV10	HINFP
TLE3	HINFP
RBX1	HINT1
AKAP8	HIPK4
ACSL3	HIPK4
UPF1	HIRA
CSNK2A2	HIRIP3
HS2ST1	HLA-A
RAB14	HLA-B
SRP72	HLA-B
RHOA	HLA-B
ATP6V1A	HLA-B
RAB5C	HLA-B
INTS4	HLA-B
PABPC1	HLA-B
CYB5B	HLA-B
ARF6	HLA-B
ATP5MG	HLA-B
NUTF2	HLA-B
RAB7A	HLA-B
CSDE1	HLA-B
GRPEL1	HLA-B
DDX21	HLA-B
DCTPP1	HLA-B
RAB2A	HLA-B
TUBGCP2	HLA-B
HMOX1	HLA-C
SCAP	HLA-C
SCARB1	HLA-C
SCARB1	HLA-DPA1
QSOX2	HLA-DPA1
RTN4	HLA-DPB1
SLC30A9	HLA-DQA1
SLC30A9	HLA-DQA2
TLE1	HLA-DQA2
ATE1	HLA-DQB2
ATP1B1	HLA-DRA
TMEM97	HLA-DRA
SCARB1	HLA-DRA
ATP1B1	HLA-DRB1
ATP1B1	HLA-DRB5
REEP6	HMBOX1
TLE5	HMGB1
MOV10	HMGB2
HMOX1	HMOX1
ELAVL1	HMOX1

UFD1	HMOX1
CUL2	HNRNPA0
MEPCE	HNRNPA0
PABPC4	HNRNPA1
MRPS2	HNRNPA1
MRPS27	HNRNPA1
TUBGCP3	HNRNPA1
AKAP8	HNRNPA1
CUL2	HNRNPA1
UPF1	HNRNPA1
PABPC1	HNRNPA1
MARK2	HNRNPA1
CUL2	HNRNPA2B1
CUL2	HNRNPA3
MEPCE	HNRNPAB
CUL2	HNRNPAB
PTBP2	HNRNPAB
MOV10	HNRNPAB
TLE5	HNRNPAB
RBM41	HNRNPC
CUL2	HNRNPD
SIRT5	HNRNPD
SDF2	HNRNPD
PTBP2	HNRNPD
PABPC1	HNRNPD
MARK3	HNRNPDL
CUL2	HNRNPDL
PABPC4	HNRNPDL
CEP250	HNRNPF
CUL2	HNRNPF
TLE5	HNRNPF
CEP250	HNRNPH1
TUBGCP3	HNRNPH1
SUN2	HNRNPH1
MEPCE	HNRNPH1
CUL2	HNRNPH1
RBM41	HNRNPK
CUL2	HNRNPK
MEPCE	HNRNPK
PABPC1	HNRNPK
CUL2	HNRNPR
TUBGCP3	HNRNPU
CEP250	HNRNPU
RBM28	HNRNPU
RPL36	HNRNPU
MRPS2	HNRNPU
PABPC4	HNRNPU
CUL2	HNRNPU
MRPS27	HNRNPU
MRPS25	HNRNPU
CCDC86	HNRNPU
MEPCE	HNRNPU
RAE1	HNRNPUL1
MEPCE	HNRNPUL2
TLE5	HOMEZ
EIF4E2	HOMEZ
HOOK1	HOOK1
TRAK2	HOOK1
TNFSF13B	HOOK1
TFIP11	HOOK1
HERC2	HOOK1

HOOK2	HOOK1
VPS18	HOOK1
USP2	HOOK1
CDC5L	HOOK1
VPS41	HOOK1
LRRC29	HOOK1
FAM160A2	HOOK1
HOOK1	HOOK3
TCF12	HOPX
RBX1	HOXB4
RAB2A	HOXB5
CYB5R3	HOXB5
CEP250	HP1BP3
CSNK2A2	HPS5
RAP1GDS1	HRAS
MOV10	HRAS
RALA	HRAS
CUL2	HRNR
P2RX4	HS2ST1
CKAP4	HS2ST1
TGOLN2	HS2ST1
TBC1D23	HS2ST1
DIAPH3	HS2ST1
PIGU	HS2ST1
ATP12A	HS2ST1
DYM	HS2ST1
TSPAN17	HS2ST1
YWHAZ	HS6ST2
GINM1	HS6ST2
KIF20A	HS6ST2
CHST10	HS6ST2
HSBP1	HSBP1
KIAA1217	HSBP1
HSF1	HSBP1
RB1CC1	HSBP1
VCP	HSBP1
LNX1	HSBP1
IFT57	HSBP1
CCHCR1	HSBP1
SCLT1	HSBP1
WASHC3	HSBP1
LIN54	HSBP1
HSPA4	HSBP1
SDCBP	HSBP1
KIFC3	HSBP1
BORCS6	HSBP1
RAB18	HSD11B1
MRPS2	HSD17B8
CSNK2A2	HSP90AA1
PRKACA	HSP90AA1
PRKACA	HSP90AB1
OS9	HSP90B1
RAE1	HSP90B1
CSNK2A2	HSP90B1
ERLEC1	HSP90B1
CUL2	HSP90B1
MOV10	HSP90B1
CUL2	HSPA1B
AP2M1	HSPA1B
RIPK1	HSPA1L
AP2M1	HSPA1L

CEP250	HSPA1L
CUL2	HSPA1L
CEP250	HSPA2
BAG5	HSPA4
DNMT1	HSPA4
SELENOS	HSPA4
MOV10	HSPA4
MEPCE	HSPA6
AP2M1	HSPA6
RBX1	HSPA8
CUL2	HSPA8
CUL2	HSPA9
GRIPAP1	HSPB1
RBX1	HSPB1
RALA	HSPB1
HECTD1	HSPB1
TUBGCP3	HSPD1
SPART	HSPD1
CUL2	HSPD1
ERMP1	HTR3C
PIGO	HTR3C
DNAJC11	HTT
JAKMIP1	HTT
BZW2	HTT
NDUFB9	HTT
AP2A2	HTT
CUL2	HTT
FBN2	HTT
PTGES2	HTT
SPART	HTT
NUP58	HTT
GNB1	HTT
CSNK2A2	HUWE1
CSDE1	HUWE1
GOLGA2	HYLS1
IDE	HYLS1
OS9	HYOU1
SGK1	HYOU1
SARM1	HYOU1
ESR2	HYOU1
GOLT1B	HYOU1
CLU	HYOU1
YAP1	HYOU1
EZH2	HYOU1
FBXO6	HYOU1
VHL	HYOU1
SCNN1A	HYOU1
CUL7	HYOU1
GABARAPL2	HYOU1
HSPA5	HYOU1
ESR1	HYOU1
RNF2	HYOU1
CDK2	HYOU1
EGFR	HYOU1
HSPA8	HYOU1
PABPC1	ICAM1
RAB18	ICAM2
ERP44	ID1
IGF1	IDE
SIRT4	IDE
IFIH1	IDE

UCHL5	IDE
NR3C1	IDE
NSFL1C	IDE
LARP4B	IFI16
RBM28	IFI16
TOR1AIP1	IFI16
NSD2	IFI16
NUP54	IFI16
LARP1	IFI16
UPF1	IFI16
NUP88	IFI16
CCDC86	IFI16
EXOSC2	IFI16
ELOC	IFI16
NUP58	IFI16
RAE1	IFI16
SUN2	IFI16
RRP9	IFI16
CSNK2A2	IFI16
NGDN	IFI16
NOL10	IFI16
EXOSC3	IFI16
NUP214	IFI16
MPHOSPH10	IFI16
EXOSC8	IFI16
NUP98	IFI16
RAB14	IFI16
MOV10	IFIT1
PABPC4	IFIT1
GRPEL1	IFIT1
PABPC1	IFIT1
PABPC1	IFIT2
PABPC4	IFIT2
PABPC4	IFIT3
PABPC1	IFIT3
ECSIT	IFIT5
G3BP1	IFRD2
MOV10	IFRD2
PABPC1	IGBP1
IDE	IGF2
TUBGCP3	IGF2BP3
RAE1	IGF2BP3
RAB5C	IGF2R
POLA2	IGFBP3
NUP214	IGHG1
HECTD1	IGSF1
PABPC1	IGSF8
RPL36	IGSF8
ERC1	IKBKB
AP2M1	IKZF1
AKAP9	IKZF3
EXOSC5	IKZF3
RHOA	IKZF3
TLE5	IKZF3
TMEM39B	IL13RA1
TRAF3IP2	IL17RA
SYK	IL17RA
SUSD4	IL17RA
MAP3K7	IL17RA
DNASE2B	IL17RA
TRAF6	IL17RA

NDFIP2	IL20RB
NDFIP2	IL2RG
REEP6	IL2RG
BRD4	IL7R
PABPC4	ILF2
UPF1	ILF2
RBM28	ILF2
CUL2	ILF2
PABPC1	ILF3
RAE1	ILF3
CUL2	ILF3
ITGB1	ILK
ELOC	ILK
AKAP8L	ILK
PKP2	ILK
DDX21	ILK
UBAP2L	ILK
MOV10	ILK
AKAP8	ILK
ECSIT	IMMT
DNAJC11	IMMT
RAB7A	IMMT
RAB5C	IMMT
TUBGCP3	IMMT
MPHOSPH10	IMP3
MPHOSPH10	IMP4
RAB5C	IMPACT
REEP5	IMPACT
PUSL1	IMPDH2
ARF6	IMPDH2
IMPDH2	IMPDH2
BCAR3	IMPDH2
ANKRD9	IMPDH2
RPA1	IMPDH2
SKP2	IMPDH2
PEX14	IMPDH2
CDK2	IMPDH2
VHL	IMPDH2
ESR1	IMPDH2
AKT1	IMPDH2
SFN	IMPDH2
OFD1	IMPDH2
TNFRSF10D	IMPDH2
XRCC3	IMPDH2
FTSJ1	IMPDH2
TNIK	IMPDH2
TEPSIN	IMPDH2
FN1	IMPDH2
CUL3	IMPDH2
UBQLN4	IMPDH2
CUL7	IMPDH2
NES	IMPDH2
COPS5	IMPDH2
APIP	IMPDH2
SMCR8	IMPDH2
GABARAPL1	IMPDH2
MCM2	IMPDH2
PIAS4	IMPDH2
HDAC5	IMPDH2
GRB2	IMPDH2
MMGT1	IMPDH2

CRY1	IMPDH2
SIRT6	IMPDH2
CPLANE2	IMPDH2
RPA3	IMPDH2
AHSA1	IMPDH2
IMPDH1	IMPDH2
MAPK10	IMPDH2
RPA2	IMPDH2
GABARAPL2	IMPDH2
SLC25A44	IMPDH2
ATG5	IMPDH2
HUWE1	IMPDH2
FBXO25	IMPDH2
EMC2	IMPDH2
FBXO6	IMPDH2
COPS6	IMPDH2
SEC16A	IMPDH2
ERGIC1	INA
MOV10	INF2
MOV10	ING5
FST	INHBE
ELAVL1	INHBE
MOV10	INKA2
DNAJC11	INPP5B
GOLGA2	INPP5J
POR	INSIG1
SCAP	INSIG1
SCAP	INSIG2
INTS4	INTS1
INTS14	INTS4
PPP2CB	INTS4
FAF2	INTS4
SEM1	INTS4
INTS10	INTS4
RPAP2	INTS4
PYGM	INTS4
PPP2CA	INTS4
SLAMF1	INTS4
INTS3	INTS4
ZMIZ2	INTS4
PPP2R1A	INTS4
USHBP1	INTS4
ISLR	INTS4
INTS6	INTS4
HECW2	INTS4
INTS4	INTS5
RALA	INTS7
AP2M1	INTU
RBX1	IP6K1
MEPCE	IPO9
RAB1A	IQCB1
LOX	IQCB1
RAB14	IQCB1
FYCO1	IQCB1
TUBGCP3	IQGAP1
NSD2	IQGAP1
PRKACA	IQGAP1
TOMM70	IRAK1
FKBP7	IRF2
PRRC2B	IRF4
TBK1	IRF7

G3BP1	ISG15
LMAN2	ISG15
MEPCE	ISG15
UBAP2L	ISG15
TMEM39B	ISLR
BCKDK	ISLR
MDN1	ISLR
RAB5C	IST1
RAB7A	IST1
ATP6V1A	IST1
PABPC1	ITCH
USP13	ITCH
ITGB1	ITGA1
ITGB1	ITGA2
RAB5C	ITGA4
RAB10	ITGA4
PABPC4	ITGA4
UPF1	ITGA4
PABPC1	ITGA4
ITGB1	ITGA5
ITGB1	ITGB1
ECSIT	ITGB1
CD63	ITGB1
LGALS3	ITGB1
ACTN1	ITGB1
CD46	ITGB1
TNFSF13B	ITGB1
UNK	ITGB1
FLT4	ITGB1
MEF2A	ITGB1
LGALS8	ITGB1
ELAVL1	ITGB1
CRKL	ITGB1
LAMA1	ITGB1
FLNB	ITGB1
CD81	ITGB1
MMP2	ITGB1
RPA3	ITGB1
ITGA10	ITGB1
RPA2	ITGB1
RAB21	ITGB1
ITGA8	ITGB1
ITGA4	ITGB1
CD36	ITGB1
RAB5A	ITGB1
RPA1	ITGB1
FBXO2	ITGB1
ATF2	ITGB1
CD9	ITGB1
FN1	ITGB1
ENO1	ITGB1
RANBP9	ITGB1
MAP4K4	ITGB1
CANX	ITGB1
SPP1	ITGB1
ITGA3	ITGB1
EPHA2	ITGB1
LAMTOR5	ITGB1
ITGB1BP1	ITGB1
PXN	ITGB1
THBS1	ITGB1

FBXO6	ITGB1
SEL1L	ITGB1
FHL2	ITGB1
ITGA6	ITGB1
ACAP1	ITGB1
LGALS3BP	ITGB1
ITGA7	ITGB1
ITGA9	ITGB1
OS9	ITGB3BP
POLA2	ITGB3BP
WFS1	ITGB3BP
ERP44	ITPR1
RHOA	ITPR1
RAB7A	ITPRID2
FKBP15	ITPRID2
CSNK2B	ITSN1
AP2M1	ITSN1
GCC1	ITSN1
ARF6	ITSN1
ERC1	ITSN2
AP2M1	ITSN2
PDE4DIP	ITSN2
RNF41	ITSN2
GOLGA2	ITSN2
GOLGB1	ITSN2
UPF1	IVNS1ABP
PABPC4	IVNS1ABP
MOV10	IWS1
CLIP4	JAKMIP2
MOV10	JMJD8
TYSND1	JOSD2
DDX21	JUN
FBXL12	JUN
NAT14	JUN
NINL	JUNB
PKP2	JUP
RAB7A	JUP
CUL2	JUP
GNB1	KALRN
TLE5	KANK2
GOLGA2	KANK2
PLEKHA5	KANSL1
NINL	KANSL1
RAE1	KAT2A
CUL2	KAT2A
DNMT1	KAT5
GOLGA2	KAT5
BRD4	KAT5
CDK5RAP2	KAT5
NINL	KAT5
NINL	KAT7
SLC30A7	KATNA1
AP2M1	KBTBD6
RBX1	KBTBD6
ERC1	KBTBD7
RBX1	KBTBD7
NUP214	KCMF1
RTN4	KCNA2
RHOA	KCNA2
G3BP1	KCND3
POLA2	KCNE3

SLC9A3R1	KCNJ1
RAB7A	KCNN4
USP13	KCTD10
RHOA	KCTD13
USP13	KCTD3
CUL2	KCTD5
HEATR3	KDM1B
REEP5	KDM2A
RAB5C	KDM2A
UPF1	KDM4A
POLA1	KDM4C
PTBP2	KHDRBS1
PTBP2	KHDRBS3
AP2M1	KIAA0232
CEP135	KIAA0753
GCC1	KIAA0753
EXOSC5	KIAA1217
RALA	KIAA2013
RAB5C	KIDINS220
RAB7A	KIDINS220
RBM28	KIF11
PLEKHA5	KIF11
NSD2	KIF11
CEP350	KIF11
ACSL3	KIF11
MARK2	KIF13B
MOV10	KIF18B
ACSL3	KIF20B
ERC1	KIF23
PLEKHA5	KIF3A
ARF6	KIF5B
MEPCE	KIF5B
SPART	KIF5B
FYCO1	KIF5B
PRKAR2B	KIF5B
MARK2	KIF5B
CSNK2A2	KIF5C
TLE5	KIFC3
CLIP4	KIFC3
SPART	KLC2
TIMM8B	KLC2
MRPS2	KLC2
TUBGCP3	KLC2
CUL2	KLF4
ELOB	KLF4
POLA2	KLF6
CUL2	KLHDC10
ELOB	KLHDC2
CUL2	KLHDC2
ELOC	KLHDC2
SELENOS	KLHDC2
ELOB	KLHDC3
CUL2	KLHDC3
TOR1A	KLHL14
PRRC2B	KLHL15
RHOA	KLHL20
RBX1	KLHL3
AAR2	KLHL36
RBX1	KLHL41
RBX1	KLHL7
TIMM29	KLK11

REEP6	KLRF1
REEP5	KLRF1
CSNK2A2	KMT2A
ELOB	KMT2A
ATP1B1	KMT2B
ERP44	KMT2C
MEPCE	KPNA2
MAT2B	KPNA3
MAT2B	KPNA4
MEPCE	KPNA5
MOV10	KPNA5
RAE1	KPNB1
NUTF2	KPNB1
AP2M1	KRT1
CUL2	KRT1
AP2M1	KRT10
CUL2	KRT10
EIF4E2	KRT13
EXOSC5	KRT13
CUL2	KRT13
TLE5	KRT13
CUL2	KRT14
AP2M1	KRT14
TLE5	KRT15
NUP54	KRT15
FOXRED2	KRT18
PKP2	KRT18
EIF4E2	KRT19
PLEKHA5	KRT19
CUL2	KRT2
AP2M1	KRT2
CUL2	KRT5
PKP2	KRT5
AP2M1	KRT5
GOLGA2	KRT6A
CUL2	KRT6A
AP2M1	KRT6A
AP2M1	KRT73
AP2M1	KRT79
PLAT	KRT8
COMT	KRTAP5-9
SCARB1	KRTAP5-9
MARK3	KSR1
PLEKHA5	KTN1
RAB7A	KTN1
RTN4	KTN1
RHOA	KTN1
PLEKHF2	L3MBTL3
NINL	L3MBTL4
CUL2	LAGE3
ELOC	LAGE3
ELOB	LAGE3
PLEKHA5	LAMB2
ATP6V1A	LAMP1
ATP6V1A	LAMTOR1
USP13	LAPTM5
CUL2	LARP1
TNNT1	LARP1
SURF2	LARP1
RNF2	LARP1
CAND1	LARP1

NIFK	LARP1
HNRNPDL	LARP1
YBX1	LARP1
ESR1	LARP1
RPL10	LARP1
EIF1B	LARP1
RPL6	LARP1
NFX1	LARP1
SRPK1	LARP1
TSG101	LARP1
NOP56	LARP1
ITGA4	LARP1
ESR2	LARP1
TARDBP	LARP1
LUC7L2	LARP1
EZH2	LARP1
GABARAPL2	LARP1
HNRNPU	LARP1
YWHAE	LARP1
CNOT2	LARP1
ELAVL1	LARP1
ILF2	LARP1
COPS5	LARP1
ECT2	LARP1
CUL1	LARP1
BARD1	LARP1
ZC3H3	LARP1
LUC7L	LARP1
AURKA	LARP1
HNRNPA1	LARP1
YWHAQ	LARP1
PRR11	LARP1
YWHAH	LARP1
IVNS1ABP	LARP1
CUL3	LARP1
HSP90AA1	LARP4B
YBX1	LARP4B
HSP90AB1	LARP4B
XPO1	LARP4B
STRAP	LARP4B
RBFOX2	LARP4B
ELAVL1	LARP4B
CUL3	LARP4B
MEPCE	LARP7
APP	LARP7
RPS2	LARP7
PAXIP1	LARP7
CDK9	LARP7
RPSA	LARP7
AFF1	LARP7
ZC3H3	LARP7
RPL6	LARP7
MED4	LARP7
CCNT1	LARP7
YBX1	LARP7
RPS8	LARP7
JMJD6	LARP7
MNDA	LARP7
FBL	LARP7
MCM7	LARP7
HNRNPA1	LARP7

TERF2	LARP7
ZC3H8	LARP7
CAND1	LARP7
ORC1	LARP7
TRA2A	LARP7
BARD1	LARP7
SIRT6	LARP7
AURKA	LARP7
NIFK	LARP7
TERF1	LARP7
RNF2	LARP7
IFI16	LARP7
LUC7L2	LARP7
DCAF7	LATS2
AKAP8L	LBR
RETREG3	LCLAT1
GOLGA2	LCOR
NPC2	LDB3
RHOA	LDB3
FAM98A	LDHD
ELOB	LDHD
MAP7D1	LDHD
MARK2	LDHD
PLEKHF2	LDOC1
TLE5	LDOC1
MYCBP2	LGALS3BP
RAE1	LGALS3BP
CUL2	LGALS3BP
ATP13A3	LGALS9
ITGB1	LGALS9
MYCBP2	LGR4
GOLGA3	LGR4
DDX21	LGR4
NUP210	LGR4
GIGYF2	LGR4
FKBP15	LGR4
ZC3H18	LGR4
USP54	LGR4
NPC2	LIG4
GGH	LIG4
RALA	LIMA1
EIF4H	LIN54
FOXRED2	LIN7C
MOV10	LIN9
GOLGA2	LINGO1
EXOSC5	LIPG
TRMT1	LIPH
REEP6	LITAF
COMT	LITAF
MOV10	LMAN2
HEATR3	LMAN2
TTYH3	LMAN2
ATP13A2	LMAN2
CENPH	LMAN2
FRAT1	LMAN2
CACNA2D1	LMAN2
RPA2	LMAN2
GPC3	LMAN2
UBL4A	LMAN2
RPA1	LMAN2
MAPK9	LMAN2

RPA3	LMAN2
NXF1	LMAN2
TOR1AIP1	LMNA
SUN2	LMNA
MOV10	LMNA
TOR1A	LMNA
TLE5	LMO2
HOOK1	LMO2
TLE5	LMO4
ECSIT	LONP1
GORASP1	LONRF1
TFAP2C	LOX
CBL	LOX
ELN	LOX
CUL2	LOXL1
ECSIT	LOXL4
SLC30A7	LPAR1
GPAA1	LPAR1
RETREG3	LPAR1
REEP6	LPAR1
PIGO	LPAR1
REEP5	LPAR1
GOLGB1	LPAR1
SCAP	LPAR1
NDUFAF1	LPAR1
ECSIT	LPAR1
TUBGCP3	LPAR6
PKP2	LPAR6
SLC27A2	LPCAT1
MOV10	LRBA
CEP250	LRCH2
PLAT	LRP1
EXOSC5	LRP4
CRTC3	LRP8
MOV10	LRP8
RTN4	LRPAP1
POLA2	LRPAP1
CEP250	LRPPRC
EDEM3	LRPPRC
RAB7A	LRPPRC
CUL2	LRR1
ELOB	LRR1
RAB5C	LRR1
RBX1	LRR1
ELOC	LRR1
RBX1	LRRC14
CUL2	LRRC14
ELOB	LRRC14
ELOC	LRRC14
MRPS27	LRRC15
NINL	LRRC39
ELOB	LRRC41
RHOA	LRRK2
PRKACA	LRRK2
MOGS	LRRK2
RIPK1	LRRK2
RAB7A	LRRK2
AP3B1	LRRK2
BAG5	LRRK2
CENPF	LRRK2
ATP5MG	LRRK2

AKAP8	LRRK2
MDN1	LRRK2
TOR1AIP1	LRRK2
AP2M1	LRRK2
NINL	LRSAM1
AP3B1	LRSAM1
TUBGCP3	LRWD1
GGH	LRWD1
TUBGCP2	LRWD1
VPS11	LSM1
EXOSC8	LSM1
MOV10	LSM10
RAE1	LSM14A
MEPCE	LSM2
RALA	LSM2
TLE5	LSM2
MEPCE	LSM3
PLEKHA5	LSM4
EXOSC5	LSM5
EXOSC8	LSM7
UPF1	LSM8
BCKDK	LSM8
PLD3	LTN1
PABPC4	LUC7L2
FAM98A	LUC7L2
CSNK2A2	LUC7L2
MEPCE	LUC7L2
NUTF2	LUC7L2
CDK5RAP2	LUC7L2
CUL2	LUC7L3
RDX	LYN
ITGB1	LYN
ACSL3	LYN
CSNK2B	LYN
NDFIP2	LYN
DDX21	LYN
PIGS	LYPD3
WFS1	LYPD3
SLC30A7	LYPD6
TUBGCP3	LYPD6
TCF12	LYSMD1
CEP250	LZTFL1
RBM41	LZTS2
NINL	LZTS2
PRKAR2B	MAATS1
ERC1	MACROD1
TOR1AIP1	MAD2L1
NEK9	MAFF
HDAC2	MAFK
EIF4E2	MAGED1
CEP250	MAGED1
AKAP9	MAGED1
AKAP8L	MAGED1
CDK5RAP2	MAGEH1
UPF1	MAGOH
TLE5	MAGOHB
CSNK2A2	MAK16
EXOSC2	MALSU1
NSD2	MAP1B
PPT1	MAP1B
RAE1	MAP1B

PABPC1	MAP1LC3A
MYCBP2	MAP1LC3A
CSNK2A2	MAP1LC3A
NEK9	MAP1LC3B
NUP88	MAP1LC3B
NUP210	MAP1LC3B
GGH	MAP1LC3B
NEK9	MAP1LC3C
FYCO1	MAP1LC3C
PRKAR2B	MAP2
RHOA	MAP3K1
AKAP8L	MAP3K3
RIPK1	MAP3K3
GNB1	MAP3K3
MARK2	MAP3K3
BAG5	MAP3K3
ERC1	MAP3K7
RBX1	MAP3K7
ECSIT	MAP3K7
RBX1	MAP4K1
RAB8A	MAP4K2
CLIP4	MAP7D1
MAP1LC3A	MAP7D1
ATN1	MAP7D1
LTBR	MAP7D1
FBXW7	MAP7D1
CEP250	MAP7D3
G3BP1	MAPK13
PABPC1	MAPK13
TLE5	MAPK14
ACSL3	MAPK4
ERC1	MAPK4
SUN2	MAPK8
CSNK2A2	MAPK8
MOV10	MAPKAPK2
RAB2A	MAPKAPK3
PRKACA	MAPRE1
ERC1	MAPRE1
MARK2	MAPT
MARK3	MAPT
MARK1	MAPT
MARK2	MARK1
YWHAE	MARK1
RHOA	MARK2
MARK2	MARK2
RSF1	MARK2
GAB2	MARK2
HDAC7	MARK2
MARK4	MARK2
UTP18	MARK2
PKP2	MARK3
MARK2	MARK3
HDAC7	MARK3
HDAC4	MARK3
RASGRF1	MARK3
PKP2	MAST3
MAT2B	MAT1A
ARF6	MAT2A
MAT2B	MAT2A
AKAP8L	MATR3
PLEKHA5	MAX

DNMT1	MBD2
RHOA	MBD5
NINL	MBIP
RAP1GDS1	MBIP
TLE5	MBNL1
RAB7A	MBOAT7
PSMD8	MCC
HYOU1	MCC
DDX21	MCC
EIF4H	MCC
CUL2	MCC
GNB1	MCC
ETFA	MCC
CYB5B	MCC
RAB2A	MCC
CSDE1	MCC
STOML2	MCC
COMT	MCC
RAB7A	MCC
ECSIT	MCCC2
RHOA	MCF2
GNB1	MCF2
RHOA	MCF2L
GNB1	MCF2L
ERP44	MCM2
MAT2B	MCM2
PABPC1	MCM2
AGPS	MCM2
CUL2	MCM3
MIB1	MCM3
RALA	MCM5
ERP44	MCM5
MIPOL1	MCM7
DDX21	MCM7
ELOC	MCM7
MIB1	MCM7
ZC3H18	MCM7
PKP2	MCM7
NUP88	MCM7
CEP250	MCM7
MOV10	MCMBP
DNAJC11	MCOLN3
MOV10	MCRIP2
GCC1	MCRS1
RAB7A	MCU
NSD2	MDC1
PDZD11	MDC1
EXOSC8	MDC1
EXOSC2	MDC1
AGPS	MDM2
RHOA	MDM2
NEK9	MDM2
FAS	MDN1
GSK3A	MDN1
NLE1	MDN1
FTSJ1	MDN1
SNW1	MDN1
CDC5L	MDN1
STK17B	MDN1
CUL7	MDN1
DNMT1	MECP2

STC2	MED1
BRD4	MED12
BRD4	MED14
RHOA	MED20
CSNK2A2	MED20
EXOSC8	MED20
RAB10	MED20
CSNK2A2	MED21
SRP54	MED23
CSNK2A2	MED23
TLE5	MED31
MOGS	MED4
EXOSC8	MED4
CSNK2A2	MED4
RAE1	MED4
TLE5	MED4
CSNK2A2	MED6
RBX1	MED8
MOV10	MED8
CUL2	MED8
ELOB	MED8
ELOC	MED8
UPF1	MEMO1
CSNK2A2	MEPCE
EXOSC2	MEPCE
EXOSC5	MEPCE
PRPF4	MEPCE
UBE2S	MEPCE
USP15	MEPCE
CHMP3	MEPCE
LSM7	MEPCE
HSP90AB1	MEPCE
EFTUD2	MEPCE
KPNA1	MEPCE
SRSF3	MEPCE
RANGAP1	MEPCE
RPS2	MEPCE
KIF11	MEPCE
PRPF6	MEPCE
MAGED2	MEPCE
ACTB	MEPCE
PRPF3	MEPCE
SNRNP200	MEPCE
HNRNPA1	MEPCE
LSM4	MEPCE
CTSA	MEPCE
HNRNPD	MEPCE
PARP1	MEPCE
LUC7L	MEPCE
MATR3	MEPCE
HSPA2	MEPCE
ARHGEF1	MEPCE
CDC73	MEPCE
SART3	MEPCE
RPLP0	MEPCE
TBC1D15	MEPCE
CAND1	MEPCE
HSPA5	MEPCE
LRRC40	MEPCE
YBX3	MEPCE
AK2	MEPCE

KPNA3	MEPCE
TRIP13	MEPCE
SF3B2	MEPCE
GTF2F1	MEPCE
SNRPN	MEPCE
AKAP7	MEPCE
IGF2BP2	MEPCE
LUC7L3	MEPCE
RPAP1	MEPCE
PRPF31	MEPCE
CCT4	MEPCE
ILF2	MEPCE
CAPZB	MEPCE
SNRPD2	MEPCE
ZFR	MEPCE
RFC5	MEPCE
UBE2M	MEPCE
KPNB1	MEPCE
SNU13	MEPCE
SET	MEPCE
HNRNPA2B1	MEPCE
ANP32E	MEPCE
ILF3	MEPCE
BAX	MEPCE
NOP56	MEPCE
SYNCRIP	MEPCE
ANP32A	MEPCE
USP4	MEPCE
MOB1A	MEPCE
TRIM28	MEPCE
SDE2	MEPCE
PHGDH	MEPCE
IGF2BP3	MEPCE
HNRNPC	MEPCE
CCNT1	MEPCE
SNRNP27	MEPCE
DHX9	MEPCE
DDX5	MEPCE
CDK9	MEPCE
HNRNPR	MEPCE
HNRNPUL1	MEPCE
CCNT2	MEPCE
DNM2	MEPCE
MAP2K2	MEPCE
RFC3	MEPCE
SNRPD3	MEPCE
YBX1	MEPCE
SNRPF	MEPCE
SNRPG	MEPCE
SNRPA1	MEPCE
KPNA6	MEPCE
RTRAF	MEPCE
CNOT9	MEPCE
CAPNS1	MEPCE
CUL3	MEPCE
FBL	MEPCE
NTPCR	MEPCE
BARD1	MEPCE
SNRPB	MEPCE
DDX17	MEPCE
SNRPB2	MEPCE

ANP32B	MEPCE
XPO1	MEPCE
IPO5	MEPCE
METTL16	MEPCE
TUBGCP2	METTL18
UGGT2	METTL23
MOV10	METTL3
RTN4	METTL7B
PABPC1	MEX3A
PABPC1	MEX3B
MARK3	MEX3C
MYCBP2	MEX3C
PABPC1	MEX3C
TLE5	MFAP1
ITGB5	MFGE8
PDGFRB	MFGE8
FUS	MFGE8
MYC	MFGE8
RAB10	MFSD1
TLE5	MIA2
MIB1	MIB1
PRKAR2B	MIB1
UBE2D1	MIB1
RANGAP1	MIB1
JAG1	MIB1
MAPRE1	MIB1
TOP3B	MIB1
PER3	MIB1
DYNLL1	MIB1
CDC27	MIB1
PCM1	MIB1
OFD1	MIB1
SNX5	MIB1
UBE2D4	MIB1
TBK1	MIB2
MIB1	MIB2
AAR2	MICA
GGH	MICA
ERLEC1	MICA
RAB10	MICAL1
PIGS	MICALL2
MOV10	MICOS10
TUBGCP2	MICOS10
DNAJC11	MICOS10
HDAC2	MIER1
PKP2	MINDY3
RAE1	MIOS
EIF4E2	MIPOL1
RBM41	MIPOL1
MIPOL1	MIPOL1
SMARCE1	MIPOL1
BYSL	MIPOL1
UTP25	MIPOL1
CDK18	MIPOL1
MFAP1	MIPOL1
TTC12	MIPOL1
LRRC39	MIPOL1
TCHP	MIPOL1
MYLIP	MIPOL1
UBE2K	MIPOL1
BEX2	MIPOL1

RCOR3	MIPOL1
EAF1	MIPOL1
CCDC33	MIPOL1
USH1C	MIPOL1
NEBL	MIPOL1
PPP1R13B	MIPOL1
ENKD1	MIPOL1
PRPF31	MIPOL1
RAB5C	MITD1
ATP6V1A	MITD1
CHMP2A	MITD1
MARK3	MITF
EXOSC8	MKRN1
PABPC1	MKRN1
RBM41	MKRN3
RAB7A	MLEC
GNB1	MLF1
RIPK1	MLF1
CDK5RAP2	MLF1
EXOSC3	MLLT3
CUL2	MLPH
RAB10	MLPH
MOV10	MLST8
USP54	MLST8
TLE5	MLX
SLC9A3R1	MME
IMPDH2	MME
MRPS25	MME
STOML2	MMGT1
EMC1	MMGT1
BRD4	MMGT1
PRIM2	MMS19
LARP4B	MMS19
PIGS	MMS19
POLA1	MMS19
POLA2	MMS19
EXOSC2	MNDA
NOL10	MNDA
LARP4B	MNDA
LARP1	MNDA
PABPC4	MNDA
UPF1	MNDA
MOV10	MNDA
EXOSC3	MNDA
NGDN	MNDA
RBM28	MNDA
CCDC86	MNDA
PABPC1	MNDA
NSD2	MNDA
NUP98	MOB1A
CEP76	MOGS
CHGB	MOGS
USP11	MOGS
UBL4A	MOGS
LGALS3BP	MOGS
MCM2	MOGS
UBA5	MOGS
CUL2	MORC2
MOGS	MORC3
GOLGA2	MORF4L1
TLE5	MORF4L2

EXOSC8	MORN4
TCF12	MORN4
TUBGCP3	MOV10
RETREG3	MOV10
CUL2	MOV10
HYOU1	MOV10
CEP250	MOV10
SCAP	MOV10
TLE5	MOV10
USP13	MOV10
MRPS5	MOV10
TCF12	MOV10
AKAP8L	MOV10
POFUT1	MOV10
ATP6AP1	MOV10
POLA1	MOV10
PRIM2	MOV10
PMPCB	MOV10
CYB5R3	MOV10
DPH5	MOV10
GRPEL1	MOV10
UBXN8	MOV10
MRPS27	MOV10
EXOSC8	MOV10
UPF1	MOV10
EIF4H	MOV10
RAB5C	MOV10
RAP1GDS1	MOV10
PRKACA	MOV10
CYB5B	MOV10
SIGMAR1	MOV10
NUTF2	MOV10
WDR1	MOV10
RAP2A	MOV10
SEC61A1	MOV10
NFATC3	MOV10
APMAP	MOV10
MLF2	MOV10
RFXANK	MOV10
HERC4	MOV10
ANXA7	MOV10
KIF4A	MOV10
TPD52L2	MOV10
MBD1	MOV10
NFYA	MOV10
OBSCN	MOV10
SLC25A39	MOV10
PEBP1	MOV10
RPS5	MOV10
C1orf109	MOV10
GCHFR	MOV10
KLHDC3	MOV10
KLHDC4	MOV10
ASF1B	MOV10
OTUD5	MOV10
UROD	MOV10
PUDP	MOV10
YAP1	MOV10
WWC1	MOV10
CCNG1	MOV10
NIPSNAP2	MOV10

RBMS2	MOV10
STX6	MOV10
NCAPD3	MOV10
DNA2	MOV10
OGDH	MOV10
EXOC2	MOV10
ASCC3	MOV10
IDH3B	MOV10
TMBIM1	MOV10
FBXO38	MOV10
CEP164	MOV10
CNDP2	MOV10
CDKN2A	MOV10
ANAPC1	MOV10
STRIP1	MOV10
TMEM132A	MOV10
SUPT16H	MOV10
OCIAD1	MOV10
ALG1	MOV10
ANXA1	MOV10
GIT2	MOV10
ARFIP2	MOV10
SARAF	MOV10
STRN4	MOV10
RIOK3	MOV10
NOP56	MOV10
ERG28	MOV10
RILPL2	MOV10
RAB5B	MOV10
ACO1	MOV10
PSMB5	MOV10
SH3KBP1	MOV10
HNRNPM	MOV10
SRRD	MOV10
PLCB3	MOV10
E2F2	MOV10
STX2	MOV10
KDM4C	MOV10
RAB32	MOV10
PTBP1	MOV10
ARPC1B	MOV10
EDC4	MOV10
HERC3	MOV10
GPR161	MOV10
RAF1	MOV10
POP1	MOV10
SETDB1	MOV10
CCP110	MOV10
CAT	MOV10
SURF6	MOV10
MAZ	MOV10
ZNF343	MOV10
APBB3	MOV10
GREB1L	MOV10
PSMD3	MOV10
ZNF207	MOV10
PALB2	MOV10
ATG16L1	MOV10
TRIP6	MOV10
CBX8	MOV10
TYK2	MOV10

RPRD1A	MOV10
WDR37	MOV10
PRUNE1	MOV10
MEF2D	MOV10
NME4	MOV10
PGD	MOV10
PLA2G4A	MOV10
UBR4	MOV10
PODXL2	MOV10
TSG101	MOV10
TFIP11	MOV10
OGFOD2	MOV10
HARS2	MOV10
PES1	MOV10
E2F1	MOV10
RBM5	MOV10
C16orf87	MOV10
TEAD2	MOV10
ITPK1	MOV10
TAB1	MOV10
HEBP2	MOV10
SH3BP5	MOV10
C1orf21	MOV10
CDC20	MOV10
ATP6V1B2	MOV10
RAB34	MOV10
HSD3B7	MOV10
YBX1	MOV10
SEMA3F	MOV10
POLR3B	MOV10
GSTM3	MOV10
NTHL1	MOV10
NOP14	MOV10
WLS	MOV10
PEX5	MOV10
FBL	MOV10
GOPC	MOV10
POGZ	MOV10
RPA3	MOV10
HAUS4	MOV10
HJURP	MOV10
CCDC25	MOV10
PNISR	MOV10
ATP6V1F	MOV10
RNF2	MOV10
CHMP1A	MOV10
HIRA	MOV10
USP5	MOV10
CPNE8	MOV10
ATP6V0B	MOV10
TP53BP1	MOV10
RIPK2	MOV10
CDH24	MOV10
ZC3H3	MOV10
YIPF1	MOV10
SAFB2	MOV10
MAP1LC3A	MOV10
NAB1	MOV10
POLDIP3	MOV10
WDR55	MOV10
GOLPH3L	MOV10

SLC39A9	MOV10
XPNPEP1	MOV10
MED29	MOV10
C1orf43	MOV10
CAND1	MOV10
CCT7	MOV10
PACSIN2	MOV10
CREBZF	MOV10
XPO7	MOV10
WDR18	MOV10
PDPR	MOV10
ADAT1	MOV10
RALY	MOV10
HDAC7	MOV10
WBP11	MOV10
MRPL37	MOV10
RPL34	MOV10
LANCL2	MOV10
SLC31A1	MOV10
XRN2	MOV10
EDF1	MOV10
LMO4	MOV10
RNF10	MOV10
ENOPH1	MOV10
ZNF787	MOV10
MORC4	MOV10
PCCB	MOV10
STAU2	MOV10
KHDRBS2	MOV10
KIF23	MOV10
KHDC4	MOV10
ARPC5L	MOV10
MBTPS1	MOV10
MIEN1	MOV10
VASP	MOV10
RAPGEF1	MOV10
PSME3	MOV10
COMMD9	MOV10
TKFC	MOV10
PTCD2	MOV10
GABARAPL1	MOV10
MTFR1	MOV10
SRSF7	MOV10
HNRNPA1	MOV10
CLCN6	MOV10
CEP78	MOV10
FAM168A	MOV10
SP100	MOV10
NSDHL	MOV10
UFD1	MOV10
MARVELD2	MOV10
PCYOX1L	MOV10
TPGS2	MOV10
TXNDC11	MOV10
HMGB3	MOV10
RPS3	MOV10
MTRF1	MOV10
RNASEH2A	MOV10
LIMA1	MOV10
CLTCL1	MOV10
RPL6	MOV10

ESPL1	MOV10
FAM50A	MOV10
LRPPRC	MOV10
STRAP	MOV10
GABARAPL2	MOV10
NVL	MOV10
COPB1	MOV10
MAN2B1	MOV10
SMNDC1	MOV10
ZSCAN29	MOV10
ENDOD1	MOV10
IDH1	MOV10
CPSF1	MOV10
POLR2K	MOV10
M6PR	MOV10
NOP2	MOV10
SMYD5	MOV10
LAMTOR3	MOV10
BARD1	MOV10
MYDGF	MOV10
GOT1	MOV10
RBM3	MOV10
CIAO3	MOV10
KIF21A	MOV10
WFDC1	MOV10
PLLP	MOV10
UBL4A	MOV10
FBXW11	MOV10
MRPL15	MOV10
CPSF7	MOV10
CREB3L4	MOV10
NFKB2	MOV10
YARS1	MOV10
PARP1	MOV10
TES	MOV10
USP22	MOV10
SFXN3	MOV10
EMC4	MOV10
PPM1H	MOV10
CUL3	MOV10
FNBP4	MOV10
SEC31A	MOV10
SRM	MOV10
KANK1	MOV10
UIMC1	MOV10
TRIM29	MOV10
SMARCE1	MOV10
REXO4	MOV10
RELT	MOV10
SLC25A19	MOV10
SLC35B3	MOV10
RPS2	MOV10
CPT1A	MOV10
DHX30	MOV10
SLC39A1	MOV10
MAD2L2	MOV10
NADK	MOV10
ZDHHC6	MOV10
MBD4	MOV10
HDGF	MOV10
HSPD1	MOV10

AOC2	MOV10
PHF14	MOV10
TP53	MOV10
ATP5MC3	MOV10
PPP5C	MOV10
PTPN18	MOV10
FAM104A	MOV10
SFPQ	MOV10
STX12	MOV10
PHF20	MOV10
TNS3	MOV10
RFX5	MOV10
NOD1	MOV10
ILF2	MOV10
ELP2	MOV10
ARMH3	MOV10
PCNP	MOV10
TPM3	MOV10
SSBP4	MOV10
EIF2AK2	MOV10
ZC3H7B	MOV10
SF3A1	MOV10
CEP192	MOV10
NUDC	MOV10
SNRPA1	MOV10
IFIT3	MOV10
TXLNA	MOV10
BOD1	MOV10
RCL1	MOV10
USPL1	MOV10
MTX2	MOV10
NCOA5	MOV10
AARS1	MOV10
KIF2C	MOV10
CENPO	MOV10
MBNL3	MOV10
OBI1	MOV10
RBM25	MOV10
STK10	MOV10
COL7A1	MOV10
ANKHD1	MOV10
USP11	MOV10
ARHGEF2	MOV10
MECR	MOV10
CCDC134	MOV10
VPS35	MOV10
ALDH18A1	MOV10
KDM3B	MOV10
ITPA	MOV10
CSAD	MOV10
ATG12	MOV10
EEF1B2	MOV10
RNF216	MOV10
MVK	MOV10
ADNP2	MOV10
ISOC2	MOV10
DHX15	MOV10
INPP5K	MOV10
SURF4	MOV10
DOCK7	MOV10
FBXO5	MOV10

KLHDC10	MOV10
SPG21	MOV10
GALNT1	MOV10
DESI1	MOV10
FOXRED1	MOV10
DPH7	MOV10
PCID2	MOV10
HSPA9	MOV10
MCM4	MOV10
JMJD4	MOV10
ALAS1	MOV10
IGF2BP3	MOV10
PRR11	MOV10
PTCD1	MOV10
KLHL7	MOV10
AP1S1	MOV10
SYT7	MOV10
CKAP4	MOV10
STUB1	MOV10
ACBD5	MOV10
SNX1	MOV10
EFEMP1	MOV10
AASDHPPT	MOV10
DHX8	MOV10
LUC7L3	MOV10
GEMIN5	MOV10
LRRC61	MOV10
ZRANB1	MOV10
MYO9A	MOV10
UNC13A	MOV10
TDP1	MOV10
NRDC	MOV10
ABCC5	MOV10
BID	MOV10
MED31	MOV10
HADH	MOV10
COQ9	MOV10
CRLF1	MOV10
EMD	MOV10
ADCK1	MOV10
COX5B	MOV10
SURF1	MOV10
CEP85	MOV10
MED28	MOV10
LRRC42	MOV10
LSG1	MOV10
MRPL49	MOV10
ARHGEF4	MOV10
ZNF76	MOV10
EPN1	MOV10
CS	MOV10
SCFD1	MOV10
FBXO30	MOV10
LIN7B	MOV10
LIG3	MOV10
GMEB2	MOV10
ELOVL1	MOV10
PPP1R13B	MOV10
TXLNG	MOV10
ZMYM3	MOV10
PEPD	MOV10

PRDX4	MOV10
CSTF2	MOV10
PPFIBP1	MOV10
USP2	MOV10
DHX9	MOV10
MKNK1	MOV10
TOMM34	MOV10
APH1A	MOV10
PMS1	MOV10
FBXW7	MOV10
POLE3	MOV10
KYAT3	MOV10
RSRC2	MOV10
EBP	MOV10
MACF1	MOV10
DCAF6	MOV10
ACTR1A	MOV10
HLX	MOV10
DHX38	MOV10
SAR1A	MOV10
PTPA	MOV10
BABAM1	MOV10
INTS6	MOV10
EPS15L1	MOV10
CAV1	MOV10
KDM4A	MOV10
ABCD3	MOV10
NT5C3B	MOV10
CENPJ	MOV10
TAF1	MOV10
ABI2	MOV10
PKP4	MOV10
HDHD3	MOV10
SLC25A3	MOV10
CHMP7	MOV10
MRPS34	MOV10
BMP8B	MOV10
PNPLA6	MOV10
ATP5F1A	MOV10
PRMT1	MOV10
TSPAN17	MOV10
COPS5	MOV10
SNRNP40	MOV10
CDC37	MOV10
ARID1A	MOV10
FANCI	MOV10
UMPS	MOV10
TARDBP	MOV10
SRSF1	MOV10
SLC25A4	MOV10
AGO1	MOV10
NTN4	MOV10
ATF4	MOV10
DAGLA	MOV10
PPHLN1	MOV10
ZNFX1	MOV10
C14orf93	MOV10
PSMC4	MOV10
ECHS1	MOV10
CTSL	MOV10
GRWD1	MOV10

MAGT1	MOV10
SNX17	MOV10
COPS4	MOV10
DMAC2	MOV10
SLC25A15	MOV10
CIRBP	MOV10
TBCCD1	MOV10
HSPA5	MOV10
LIG1	MOV10
KIF3B	MOV10
CREB3	MOV10
MPV17	MOV10
TWSG1	MOV10
RPA1	MOV10
RPA2	MOV10
TUBA4A	MOV10
DDX24	MOV10
AHCTF1	MOV10
GRHPR	MOV10
CLK4	MOV10
JMJD6	MOV10
COMMD3	MOV10
MIGA2	MOV10
HNRNPD	MOV10
SNX8	MOV10
ST6GALNAC4	MOV10
MRPL22	MOV10
ITGA6	MOV10
RNF185	MOV10
DLGAP4	MOV10
CCDC97	MOV10
RANBP3	MOV10
TANC1	MOV10
PRPF19	MOV10
XPO5	MOV10
RCN1	MOV10
A1BG	MOV10
UQCRC1	MOV10
DPH1	MOV10
POLDIP2	MOV10
RPTOR	MOV10
ZSCAN32	MOV10
VPS26B	MOV10
LUC7L2	MOV10
ZMYM4	MOV10
NQO2	MOV10
MRPS26	MOV10
NPRL2	MOV10
HMG20A	MOV10
SMARCC2	MOV10
TCTN3	MOV10
NDUFS6	MOV10
NEDD4L	MOV10
ARL2BP	MOV10
KNSTRN	MOV10
FYTTD1	MOV10
PRKCA	MOV10
BCLAF1	MOV10
ANAPC13	MOV10
NFX1	MOV10
PUM3	MOV10

TBRG4	MOV10
DDX59	MOV10
TXN2	MOV10
C9orf40	MOV10
MPDU1	MOV10
TAZ	MOV10
GID4	MOV10
MRPS10	MOV10
UTP4	MOV10
DCAF1	MOV10
GID8	MOV10
ZFR	MOV10
MRPL18	MOV10
RNF144B	MOV10
THRA	MOV10
ABCF2	MOV10
COPA	MOV10
STAU1	MOV10
STBD1	MOV10
BIN1	MOV10
UBC	MOV10
SCAMP1	MOV10
ACAT2	MOV10
RPIA	MOV10
TLN1	MOV10
ANKRD52	MOV10
GGA3	MOV10
ZBTB11	MOV10
TNPO2	MOV10
HNRNPH3	MOV10
TRMT2A	MOV10
DKC1	MOV10
PIP5K1A	MOV10
SMYD2	MOV10
RHOF	MOV10
CRKL	MOV10
MAGI1	MOV10
MTR	MOV10
CDC16	MOV10
ZDHHC7	MOV10
IFNGR1	MOV10
EFCAB11	MOV10
B4GALT4	MOV10
DDAH1	MOV10
NUMA1	MOV10
DSN1	MOV10
KIF22	MOV10
ALDOC	MOV10
DBN1	MOV10
ACTL6A	MOV10
PXN	MOV10
TNFRSF21	MOV10
LETMD1	MOV10
PFKM	MOV10
GNPDA1	MOV10
EMC8	MOV10
ZNF740	MOV10
LAMTOR5	MOV10
BNIP2	MOV10
SZRD1	MOV10
GUK1	MOV10

NDE1	MOV10
POLR1A	MOV10
SLC25A17	MOV10
VPS36	MOV10
SNRPC	MOV10
ZNF174	MOV10
RAB7A	MPG
CSNK2A1	MPHOSPH10
RNF2	MPHOSPH10
EXOSC2	MPHOSPH6
EXOSC3	MPHOSPH6
EXOSC5	MPHOSPH6
EXOSC8	MPHOSPH6
RPL36	MPHOSPH8
MOV10	MPHOSPH8
RHOA	MPRIP
RAE1	MPST
PCNT	MPV17L2
ELOC	MRAS
PMPCB	MRM3
CUL2	MRM3
SLC30A7	MRPL1
NDUFAF1	MRPL1
MOV10	MRPL11
MOV10	MRPL13
USP13	MRPL20
MRPS27	MRPL3
PRIM1	MRPL38
EXOSC5	MRPL41
EXOSC2	MRPL48
GDF15	MRPL50
RAB5C	MRPL55
PMPCA	MRPL58
TARS2	MRPL58
MRPS25	MRPL58
ATP5MG	MRPL58
MRPS5	MRPL58
ETFA	MRPL58
AASS	MRPL58
MOV10	MRPL58
AP2M1	MRPL58
PMPCB	MRPL58
GRPEL1	MRPL58
MRPS27	MRPL58
STOML2	MRPL58
MRPS2	MRPL58
MRPS5	MRPS11
MRPS2	MRPS11
RAB5C	MRPS14
TUBGCP3	MRPS18B
MOV10	MRPS18B
CEP250	MRPS18B
MRPS5	MRPS18C
MRPS2	MRPS18C
MOV10	MRPS18C
ZC3H3	MRPS2
TPD52	MRPS2
C1QBP	MRPS2
TPD52L2	MRPS2
MRPS31	MRPS2
ESR1	MRPS2

MRPS33	MRPS2
CAND1	MRPS2
RPL6	MRPS2
MRPS14	MRPS2
MRPS5	MRPS22
CEP250	MRPS22
MRPS2	MRPS23
CEP250	MRPS23
MOV10	MRPS23
MRPS5	MRPS23
TUBGCP3	MRPS23
TUBGCP3	MRPS25
MRPS31	MRPS25
PCDHB15	MRPS25
C1QBP	MRPS25
COPS5	MRPS25
ERBB3	MRPS25
CAND1	MRPS25
ZC3H3	MRPS25
CEP76	MRPS25
FBXO31	MRPS27
ELAVL1	MRPS27
CAND1	MRPS27
CUL1	MRPS27
CEP76	MRPS27
NECAB2	MRPS27
KHDRBS2	MRPS27
LUC7L	MRPS27
ERBB3	MRPS27
RIOK2	MRPS27
THOC5	MRPS27
C1QBP	MRPS27
FBXW11	MRPS27
CUL3	MRPS27
MYH7B	MRPS27
RPL6	MRPS27
MRPS31	MRPS27
ESR1	MRPS27
TUBGCP3	MRPS28
MRPS2	MRPS28
MRPS2	MRPS5
MRPS25	MRPS5
MRPS27	MRPS5
CUL3	MRPS5
RPL6	MRPS5
ESR2	MRPS5
HNRNPA1	MRPS5
RPS8	MRPS5
DAP3	MRPS5
LUC7L	MRPS5
TP53	MRPS5
MRPS33	MRPS5
MRPS31	MRPS5
MRPS7	MRPS5
MRPS9	MRPS5
DDX56	MRPS5
ZC3H3	MRPS5
CAND1	MRPS5
C1QBP	MRPS5
PTCD3	MRPS5
SNX3	MRPS5

MRPS26	MRPS5
MRPS15	MRPS5
MRPS2	MRPS7
MRPS2	MRPS9
CEP250	MRPS9
DNMT1	MRTFA
MOV10	MSL1
SLC9A3R1	MSN
MOV10	MSRB1
GOLGA2	MSRB3
MOV10	MSRB3
DNMT1	MTA1
DNMT1	MTA2
TIMMDC1	MTCH1
PSEN1	MTCH1
SLAMF1	MTCH1
EDEM1	MTCH1
UBXN8	MTCH2
MOV10	MTCL1
MARK3	MTCL1
MARK2	MTCL1
RETREG3	MT-CO1
TM2D3	MTFP1
ECSIT	MT-ND1
ACAD9	MT-ND6
RAB1A	MTOR
GNB1	MTOR
MOV10	MTOR
RBX1	MTSS1
TLE5	MTUS2
PKP2	MTUS2
MOV10	MXRA7
ARF6	MYADM
RBX1	MYB
ELOB	MYBBP1A
NSD2	MYBBP1A
CLIP4	MYBPC1
LOX	MYC
AKAP8	MYC
TUBGCP2	MYC
MYCBP2	MYC
AKAP8L	MYC
ECSIT	MYC
ERC1	MYC
AASS	MYC
MAP7D1	MYC
CDK5RAP2	MYC
MOGS	MYC
MDN1	MYC
SIRT5	MYC
NEK9	MYC
NUP98	MYC
DNMT1	MYC
ERP44	MYC
CENPF	MYC
PPT1	MYC
MIB1	MYC
ATP6AP1	MYEF2
PIGS	MYH6
ELOC	MYH6
SUN2	MYH9

DNMT1	MYL6B
USP13	MYO15A
AP2M1	MYO18A
MOV10	MYO18A
SLC30A6	MYO18A
ATP6V1A	MYO1B
IMPDH2	MYO1C
RALA	MYO1C
TIMM29	MYO1C
TIMM8B	MYO1C
SUN2	MYO1C
ATP6V1A	MYO1D
MOV10	MYO1E
MOV10	MYO6
RAB5C	MYOF
RAB7A	MYOF
ATP6V1A	MYOF
TUBGCP2	MZT1
ZC3H18	MZT1
MRPS5	MZT1
CENPF	MZT1
TUBGCP3	MZT1
TUBGCP3	MZT2A
CDK5RAP2	MZT2A
TUBGCP2	MZT2A
TUBGCP2	MZT2B
CDK5RAP2	MZT2B
TUBGCP3	MZT2B
PRKAR2B	NAA25
SCARB1	NAAA
OS9	NAAA
SCAP	NAALADL2
TLE5	NAB2
MTCH1	NABP2
UPF1	NADSYN1
ALG8	NAE1
TMEM39B	NAGS
QSOX2	NAP1L1
CUL2	NAP1L1
MOV10	NAP1L4
CEP250	NAP1L4
MARK2	NAP1L5
MOV10	NAXD
RETREG3	NCAM1
HECTD1	NCAPG
HECTD1	NCAPH
UPF1	NCBP1
UPF1	NCBP2
NUP98	NCBP2
GNB1	NCF2
HEATR3	NCK1
CSNK2A2	NCKIPSD
CSNK2A2	NCL
CUL2	NCL
PABPC1	NCL
TCF12	NCOR1
SCAP	NCOR1
AP2M1	NCOR2
ATP6V1A	NCSTN
TIMM29	NCSTN
CSNK2A2	NDE1

CENPF	NDEL1
PKP2	NDEL1
ELAVL1	NDFIP2
ITCH	NDFIP2
NEDD4	NDFIP2
FYN	NDFIP2
MLH1	NDFIP2
NEDD4L	NDFIP2
GOLGA2	NDN
PABPC1	NDRG1
UPF1	NDRG1
ATP1B1	NDRG2
BCKDK	NDUFA10
GHITM	NDUFA11
NDUFAF1	NDUFA12
NDUFB9	NDUFA12
ECSIT	NDUFA3
NDUFB9	NDUFA6
RAB7A	NDUFA7
RAB5C	NDUFA7
RAB7A	NDUFA8
ECSIT	NDUFAF1
TIMMDC1	NDUFAF1
FAF2	NDUFAF1
PPP2CB	NDUFAF1
STK26	NDUFAF1
FAS	NDUFAF1
NDUFS1	NDUFAF1
NDUFA8	NDUFAF1
PNN	NDUFAF1
ATXN3	NDUFAF2
ACAD9	NDUFAF3
NDUFAF1	NDUFAF3
FOXRED2	NDUFAF3
ECSIT	NDUFAF3
ECSIT	NDUFB1
UPF1	NDUFB10
ECSIT	NDUFB11
NDUFAF1	NDUFB11
RAB5C	NDUFB11
ECSIT	NDUFB5
RAB7A	NDUFB7
RAB7A	NDUFB8
ECSIT	NDUFB8
RBBP6	NDUFB9
NDUFA8	NDUFB9
TIMMDC1	NDUFB9
SH3GL3	NDUFB9
MEF2A	NDUFB9
XRN1	NDUFB9
ELP1	NDUFB9
IMMT	NDUFB9
PRPS1	NDUFB9
ECSIT	NDUFS2
MOV10	NDUFS2
ECSIT	NDUFS3
NDUFAF1	NDUFS3
MOV10	NDUFS3
ACAD9	NDUFS3
NDUFB9	NDUFS3
RAB5C	NDUFS5

ECSIT	NDUFS5
NDUFB9	NDUFS5
NDUFAF1	NDUFS5
RAB7A	NDUFS6
RAB7A	NDUFV2
MOV10	NDUFV3
PVR	NECTIN3
ERO1B	NECTIN3
SDF2	NECTIN3
NPTX1	NECTIN3
NUP54	NEDD1
TUBGCP2	NEDD1
TUBGCP3	NEDD1
PABPC1	NEDD8
ELOB	NEDD8
CUL2	NEDD8
RBX1	NEDD8
USP13	NEDD8
TLE5	NEK6
NEK9	NEK7
NEK9	NEK9
DYNLL1	NEK9
HSP90AB1	NEK9
NSMAF	NEK9
GABARAPL2	NEK9
NEK6	NEK9
HSP90AA1	NEK9
HUWE1	NEK9
MAP1LC3A	NEK9
XPO1	NEK9
G3BP2	NEPRO
IDE	NES
RRP9	NES
RHOA	NET1
HS6ST2	NETO2
GLB1	NEU1
VAPA	NEU1
JUNB	NEU1
LIG4	NEU1
CTSA	NEU1
FBXO6	NEU1
EEF1A1	NEU1
HDAC5	NEU1
MAP7D1	NEURL4
CEP250	NEURL4
PABPC4	NF2
GRPEL1	NF2
SLC9A3R1	NF2
MOV10	NF2
AKAP8L	NF2
CLCC1	NF2
G3BP2	NF2
MOV10	NFATC2IP
PLD3	NFKB1
PABPC1	NFKB2
RHOA	NFKBIA
ERC1	NFKBIA
CUL2	NFRKB
PABPC1	NFX1
BECN1	NGDN
RNF2	NGDN

PRKD2	NGDN
KLC3	NGDN
EGFR	NGLY1
TRAFD1	NGLY1
GUCD1	NGLY1
DERL1	NGLY1
SRPK2	NGLY1
B4GALT4	NGLY1
APP	NGLY1
NSFL1C	NGLY1
FBXO6	NGLY1
UBQLN1	NGLY1
RAD23B	NGLY1
TMEM25	NGLY1
DCTPP1	NIF3L1
UPF1	NIFK
CSNK2A2	NIFK
RBM28	NIFK
NIN	NIN
BAX	NIN
RTRAF	NIN
ELAVL1	NIN
GSK3B	NIN
RBM41	NINL
CDC27	NINL
MAD1L1	NINL
MCM10	NINL
GPKOW	NINL
ELOA	NINL
BRCA1	NINL
FOXRED2	NIPSNAP1
MOV10	NIPSNAP1
CSNK2A2	NKAP
CSNK2A2	NKAPD1
CUL2	NKAPL
MOV10	NKRF
CSNK2A2	NKTR
CHPF	NKX3-1
MTCH1	NLRP3
CCDC86	NLRP3
AP3B1	NLRP3
LPAR6	NLRX1
GNAI2	NLRX1
SIRT4	NLRX1
CREB3	NLRX1
HAUS6	NLRX1
MYC	NLRX1
TRAF3	NLRX1
CDIPT	NLRX1
IKBKB	NLRX1
SARM1	NLRX1
RTN4	NME2
G3BP1	NME2
TUBGCP3	NME7
CDK5RAP2	NME7
TUBGCP2	NME7
HS2ST1	NMNAT3
MOV10	NOC2L
CEP250	NOC4L
RIPK1	NOD2
EED	NOL10

MARK3	NOL11
GNB1	NOL11
G3BP1	NOL6
MOV10	NOL6
MOV10	NOL8
CSNK2A2	NOLC1
MOV10	NOLC1
MOV10	NOMO2
CUL2	NONO
RALA	NOS1AP
RAB5C	NOS1AP
AP2M1	NOS1AP
MYCBP2	NOS2
MDN1	NOTCH1
TCF12	NOTCH1
ELOC	NOTCH4
BRCA1	NPC2
NEDD4L	NPC2
DHDDS	NPC2
ECT2	NPC2
USP4	NPC2
MOV10	NPLOC4
UBXN8	NPLOC4
USP13	NPLOC4
SELENOS	NPLOC4
LARP1	NPM1
RPL36	NPM1
IMPDH2	NPM1
MOV10	NPM1
GOLGA2	NPM1
G3BP1	NPM1
FAM98A	NPM1
DDX21	NPM1
NUP98	NPM1
MEPCE	NPM1
CUL2	NPM1
RBM28	NPM1
CENPF	NPM1
PABPC4	NPM1
SRP72	NPM1
LARP4B	NPM1
CCDC86	NPM1
ABCC1	NPM1
GOLGA3	NPM1
MOV10	NPR2
CHST10	NPTX1
BMP7	NPTX1
CD96	NPTX1
PPP2CA	NPTX1
SIAE	NPTX1
TMEM132A	NPTX1
FCGR3B	NPTX1
OGN	NPTX1
ELAVL1	NPTX1
MOV10	NQO1
MYCBP2	NR1D1
CSNK2A2	NR1D2
DNMT1	NR2C2
TM2D3	NR2F2
MOV10	NR2F6
GNB1	NR3C1

POLA2	NR4A1
RTN4	NR4A1
BCKDK	NR5A2
RAP1GDS1	NRAS
ELOB	NRBP1
ERP44	NRDC
MTCH1	NRG1
HMOX1	NRG1
TMEM39B	NRG1
DNMT1	NRIP1
SUN2	NRM
CSNK2A2	NRP1
CCDC86	NSA2
PABPC4	NSA2
NSD2	NSD2
GOLGA3	NSD2
MYCBP2	NSD2
MTREX	NSD2
PRR11	NSD2
ZC3H3	NSD2
RPL6	NSD2
KDM1A	NSD2
THRAP3	NSD2
CCDC88C	NSD2
DDX20	NSD2
GEMIN5	NSD2
RPLP0	NSD2
BRD4	NSD3
RIPK1	NTAN1
DDX21	NTRK1
SLC9A3R1	NTRK1
SCCPDH	NTRK1
MRPS5	NTRK1
RBM28	NTRK1
CSNK2A2	NTRK1
MRPS27	NTRK1
RAP1GDS1	NTRK1
GNB1	NTRK1
ZYG11B	NTRK1
ETFA	NTRK1
MAP7D1	NTRK1
AGPS	NTRK1
ACSL3	NTRK1
MARK2	NTRK1
ACAD9	NTRK1
MEPCE	NTRK1
TCF12	NTRK1
PABPC4	NTRK1
GRPEL1	NTRK1
HDAC2	NTRK1
FAM98A	NTRK1
LARP7	NTRK1
EIF4H	NTRK1
ZC3H18	NTRK1
GGH	NTRK1
NEK9	NTRK1
POR	NTRK1
RALA	NTRK1
CUL2	NTRK1
LMAN2	NTRK1
PABPC1	NTRK1

MOGS	NTRK1
ERP44	NTRK1
GTF2F2	NTRK1
ACADM	NTRK1
LARP1	NTRK1
RAB5C	NTRK1
RTN4	NTRK1
SRP72	NTRK1
TARS2	NTRK1
UBAP2L	NTRK1
UBAP2	NTRK1
GOLGA2	NTRK1
IMPDH2	NTRK1
GOLGA3	NTRK1
LARP4B	NTRK1
TUBGCP3	NTRK1
PRIM2	NTRK1
COLGALT1	NTRK1
NUP210	NTRK1
TOR1AIP1	NTRK1
MARK3	NTRK1
VPS39	NTRK1
G3BP1	NTRK1
SUN2	NTRK1
UPF1	NTRK1
EMC1	NTRK1
PLD3	NTRK1
WASHC4	NTRK1
DDX10	NTRK1
STOML2	NTRK1
SRP54	NTRK1
ABCC1	NTRK1
ATP1B1	NTRK1
ZC3H7A	NTRK1
TLE3	NTRK1
G3BP2	NTRK1
ATP6V1A	NTRK1
MIB1	NTRK1
MRPS2	NTRK1
ERGIC1	NTRK1
AKAP8L	NTRK1
MOV10	NTRK1
DNMT1	NTRK1
CSDE1	NTRK1
AP3B1	NTRK1
IDE	NTRK1
RDX	NTRK1
POLA1	NTRK1
HYOU1	NTRK1
TOMM70	NTRK1
INTS4	NTRK1
DNAJC11	NTRK1
NUP54	NTRK1
NUP88	NTRK1
EDEM3	NTRK1
TBK1	NTRK1
AP2A2	NTRK1
TUBGCP2	NTRK1
VPS11	NTRK1
SBNO1	NTRK1
ITGB1	NTRK1

PSMD8	NTRK1
NUP214	NTRK1
BZW2	NTRK1
NEK9	NUDCD2
NUP98	NUDT21
TLE5	NUDT22
MOV10	NUDT3
REEP5	NUDT3
NUP98	NUMA1
RAE1	NUMA1
NUP98	NUP107
RAE1	NUP107
NUP88	NUP107
NUP98	NUP153
RAE1	NUP153
NUP88	NUP153
NUTF2	NUP153
MOV10	NUP205
NUP58	NUP205
EXOSC5	NUP210
FBXO6	NUP210
TRIP6	NUP210
CUL7	NUP210
UBE2I	NUP210
EED	NUP210
FN1	NUP210
SEH1L	NUP210
NUP153	NUP210
NUP107	NUP210
ITGA4	NUP210
OBSL1	NUP210
GABARAPL2	NUP210
KPNB1	NUP210
CUL2	NUP214
NUP214	NUP214
NUP88	NUP214
RAE1	NUP214
HDAC9	NUP214
NUP153	NUP214
SEH1L	NUP214
HDAC5	NUP214
NUP107	NUP214
UBE2I	NUP214
ITGA4	NUP214
MAP1LC3A	NUP214
OBSL1	NUP214
XPO5	NUP214
CUL7	NUP214
KPNB1	NUP214
HDAC4	NUP214
CLEC11A	NUP214
XPO1	NUP214
CUL3	NUP214
IPO5	NUP214
FBXO6	NUP214
NUP58	NUP35
NUP214	NUP42
RAE1	NUP42
MARK2	NUP42
NUP98	NUP43
NUTF2	NUP54

KPNB1	NUP54
ADD1	NUP54
OBSL1	NUP54
APC	NUP54
NUP107	NUP54
KPNA1	NUP54
AP2B1	NUP54
IFT20	NUP54
HDAC1	NUP54
NUP214	NUP58
GDF15	NUP58
NUP54	NUP58
NUP98	NUP58
NDC1	NUP58
OBSL1	NUP58
NUP188	NUP58
ABCC5	NUP58
TRAF1	NUP58
UBQLN1	NUP58
NUP93	NUP58
PHF21A	NUP58
NUP42	NUP58
KIAA1191	NUP58
ABI3	NUP58
COG2	NUP58
NTPCR	NUP58
APC	NUP58
NUP107	NUP58
MAPK3	NUP58
CUL2	NUP88
RAE1	NUP88
KPNB1	NUP88
CD82	NUP88
GABARAPL2	NUP88
CUL3	NUP88
SEH1L	NUP88
RAE1	NUP93
RAE1	NUP98
NUP88	NUP98
CUL7	NUP98
MAPK8	NUP98
TNPO1	NUP98
KPNB1	NUP98
EED	NUP98
CREBBP	NUP98
NUP133	NUP98
CLEC11A	NUP98
TPR	NUP98
UBE2I	NUP98
PIM2	NUP98
TNPO2	NUP98
EP300	NUP98
HNRNPUL1	NUP98
CDC37	NUP98
RAPGEF3	NUP98
SEH1L	NUP98
CHMP4B	NUP98
NUTF2	NUTF2
ELAVL1	NUTF2
FTSJ1	NUTF2
RHOA	NVL

EXOSC8	NXF1
MRPS5	NXF1
CEP350	NXF1
NUP214	NXF1
TUBGCP3	NXF1
POLA1	NXF1
POFUT1	NXF1
HYOU1	NXF1
DPH5	NXF1
NDUFB9	NXF1
MRPS27	NXF1
PMPCB	NXF1
CYB5R3	NXF1
ALG8	NXF1
FAM98A	NXF1
RAE1	NXF1
ATP6AP1	NXF1
NUP98	NXF1
NUP88	NXF1
NUTF2	NXF1
SIGMAR1	NXF1
VPS11	NXF1
ZC3H18	NXF1
RAB5C	NXF1
EXOSC3	NXF1
AKAP8	NXF1
PRKACA	NXF1
SCAP	NXF1
CEP250	NXF1
RETREG3	NXF1
UBXN8	NXF1
UBAP2L	NXF1
TCF12	NXF1
CYB5B	NXF1
EXOSC2	NXF1
CUL2	NXF1
PABPC4	OAS3
RAB7A	OAZ1
UPF1	OAZ1
MOV10	OAZ2
BCKDK	OBI1
CIT	OBSL1
MDN1	OBSL1
CENPF	OBSL1
RBM28	OBSL1
RAE1	OBSL1
ACSL3	OBSL1
RRP9	OBSL1
PABPC1	OBSL1
CCDC86	OBSL1
NUP98	OBSL1
RAB5C	OCIAD1
RAB14	OCRL
PRKAR2B	OFD1
EXOSC5	OGDH
USP13	OGDH
RAB10	OPTN
RAB14	OPTN
OS9	OS9
FOXRED2	OS9
ERLEC1	OS9

COLGALT1	OS9
NUP210	OS9
RBX1	OS9
UGGT2	OS9
HIF1A	OS9
TRPV4	OS9
SERPINC1	OS9
ACVR1B	OS9
HSPA5	OS9
DNAJB11	OS9
FAF2	OS9
FOXM1	OS9
FBXO6	OS9
SNRPD2	OS9
CLN5	OS9
SEL1L	OS9
CREB3	OS9
DERL2	OS9
EGLN3	OS9
LONP2	OS9
MYH9	OS9
AUP1	OS9
MOV10	OSBP2
TCF12	OSGIN1
TLE5	OSGIN1
EIF4H	OTUB1
ATP6V1A	OTUB1
SRP54	OTUB1
ELOC	OTUB1
CSDE1	OTUB1
ERP44	OTUB1
GLA	OTUD4
EXOSC8	OTUD4
MOV10	OXA1L
NARS2	OXLD1
EMC1	P2RX4
COQ8B	P2RX4
SLC9A3R1	P2RY1
SAAL1	P2RY12
SLC30A7	P2RY12
ECSIT	P2RY12
MOV10	P2RY2
ATP13A3	P2RY8
CSNK2A2	P4HB
CUL2	P4HB
ERO1B	P4HB
MOV10	PA2G4
DDX21	PA2G4
EXOSC5	PA2G4
PSMD8	PAAF1
PABPC1	PABPC1
UPF1	PABPC1
GABARAPL2	PABPC1
MAP3K14	PABPC1
BRCA1	PABPC1
EIF4B	PABPC1
CUL3	PABPC1
ELAVL1	PABPC1
CUL7	PABPC1
DCUN1D1	PABPC1
CRY1	PABPC1

CUL1	PABPC1
SMG6	PABPC1
ZMYND11	PABPC1
CUL3	PABPC4
PRR11	PABPC4
CRY1	PABPC4
TSG101	PABPC4
MCM2	PABPC4
CUL7	PABPC4
EIF2AK2	PABPC4
DCUN1D1	PABPC4
FBXW11	PABPC4
ELAVL1	PABPC4
GABARAPL2	PABPC4
XPO1	PABPC4
CUL1	PABPC4
TNRC6C	PABPC4
RPL6	PABPC4
ZC3H3	PABPC4
CSDE1	PABPC4L
ERP44	PACSIN2
PABPC1	PAIP1
CSDE1	PAIP1
PABPC1	PAIP2
PABPC1	PAIP2B
ZC3H7A	PAK1
GNB1	PAK1
CSDE1	PAK2
GRPEL1	PALLD
DNAJC19	PAM16
ERC1	PAN2
GNB1	PAN2
RPL36	PAN2
ELOB	PAN2
PABPC1	PAN2
PABPC4	PAN2
PABPC1	PAN3
MARK2	PARD3
DCAF7	PARD3
MARK2	PARD6A
AGPS	PARD6B
MARK2	PARD6G
UPF1	PARN
PABPC1	PARN
POLA1	PARP1
DNMT1	PARP1
POLA2	PARP1
NUP98	PARP11
CUL2	PARP14
ECSIT	PARP16
MDN1	PARP16
VPS11	PARVA
TLE5	PARVG
MOV10	PAXBP1
ATP1B1	PAXIP1
NPC2	PAXIP1
ECSIT	PAXIP1
LARP1	PAXIP1
G3BP1	PAXIP1
SCCPDH	PAXIP1
FBLN5	PAXIP1

CSNK2A2	PBK
MOV10	PBX2
RAB7A	PC
CDK5RAP2	PCBD1
CUL2	PCBP1
TUBGCP3	PCBP1
PABPC1	PCBP1
PABPC1	PCBP2
MEPCE	PCBP2
SLU7	PCBP2
CUL2	PCBP2
CEP250	PCBP2
PTBP2	PCBP3
ARF6	PCBP3
CEP350	PCDH7
TLE1	PCDHA4
MOV10	PCF11
ERC1	PCGF1
UPF1	PCGF1
CSNK2A2	PCGF3
CSNK2A2	PCGF5
TUBGCP3	PCID2
DNMT1	PCLAF
PRKAR2B	PCM1
CUL2	PCMT1
ELOB	PCMTD2
CUL2	PCMTD2
ELOC	PCMTD2
MRPS25	PCNA
NSD2	PCNA
DNMT1	PCNA
CUL2	PCNA
CEP68	PCNT
TUBGCP2	PCNT
TUBGCP3	PCNT
MIB1	PCNT
CDK5RAP2	PCNT
PRKAR2A	PCNT
TSNAX	PCNT
CDC5L	PCNT
C7orf50	PCNT
SLC25A4	PCNT
TP53BP1	PCNT
PCM1	PCNT
RBBP7	PCNT
A1BG	PCNT
CHD4	PCNT
CEP162	PCNT
MBD3	PCNT
RPGRIP1L	PCNT
OBSL1	PCNT
MTA2	PCNT
TXNDC11	PCNT
DYNC1LI1	PCNT
PIBF1	PCNT
IMP4	PCNT
CEP72	PCNT
PON2	PCNT
BIRC7	PCNT
MAPRE1	PCNT
DTNBP1	PCNT

FGL2	PCNT
OFD1	PCNT
WDR35	PCNT
CEP131	PCNT
CUL2	PDCD6
MOV10	PDCD6
MARK2	PDCD7
GNB1	PDCL
MOV10	PDE2A
MOV10	PDE4B
CLIP4	PDE4DIP
PRKAR2B	PDE4DIP
PRKACA	PDE4DIP
SCCPDH	PDE4DIP
SCNM1	PDE4DIP
ELAVL1	PDE4DIP
KDM1A	PDE4DIP
CDKN1A	PDE4DIP
ZNF408	PDE4DIP
PRPF31	PDE4DIP
CHCHD3	PDE4DIP
PDE4D	PDE4DIP
FHL1	PDE4DIP
LAMTOR3	PDE4DIP
MTUS2	PDE4DIP
PPP1R18	PDE4DIP
DYNLL1	PDE4DIP
ANKRD11	PDE4DIP
BYSL	PDE4DIP
RNF2	PDE4DIP
GFI1B	PDE4DIP
TK1	PDE4DIP
ZFYVE21	PDE4DIP
MIF4GD	PDE4DIP
OSGEP	PDE4DIP
MLF1	PDE4DIP
C1orf109	PDE4DIP
FAM161A	PDE4DIP
KRAS	PDE4DIP
CEP170	PDE4DIP
KRT18	PDE4DIP
KLC3	PDE4DIP
PRKACB	PDE4DIP
SH2D4A	PDE4DIP
LMO4	PDE4DIP
NAA10	PDE4DIP
ZNF581	PDE4DIP
CCDC146	PDE4DIP
SDCBP	PDE4DIP
FCHSD2	PDE4DIP
TRAF4	PDE4DIP
ZBTB38	PDE4DIP
SMN1	PDE4DIP
AKTIP	PDE4DIP
MAPRE1	PDE4DIP
SHFL	PDE4DIP
SNCA	PDE4DIP
PFDN1	PDE4DIP
KLC4	PDE4DIP
IMMT	PDE4DIP
GADD45G	PDE4DIP

LENG1	PDE4DIP
NEK2	PDE4DIP
VAC14	PDE4DIP
PYGM	PDE4DIP
ARNT2	PDE4DIP
PRNP	PDE4DIP
PIAS4	PDE4DIP
TULP3	PDE4DIP
USO1	PDE4DIP
UBE2J2	PDE4DIP
HAUS1	PDE4DIP
CDC5L	PDE4DIP
TNIK	PDE4DIP
MYBPC2	PDE4DIP
TFIP11	PDE4DIP
RAB18	PDE6D
RHOA	PDE6D
COL6A1	PDGFA
SLC9A3R1	PDGFRA
SLC9A3R1	PDGFRB
PPT1	PDHA1
PPT1	PDHB
ITGB1	PDHB
ERO1B	PDIA2
COL6A1	PDIA3
ITGB1	PDIA3
ERO1B	PDIA3
ERO1B	PDIA4
AP2M1	PDLIM7
ATP6V1A	PDLIM7
GNB1	PDLIM7
ELOB	PDLIM7
PRKACA	PDPK1
MOV10	PDXK
MOV10	PDXP
PLEKHA5	PDZD11
YWHAE	PDZD11
GINM1	PDZD11
SLC9A3R1	PDZK1
SCARB1	PDZK1
TOR1AIP1	PEAK1
CLIP4	PEG10
RIPK1	PELI1
TBK1	PELI1
CUL2	PEPD
AP2M1	PER1
AGPS	PEX14
FKBP7	PEX19
HS2ST1	PEX19
FAR2	PEX19
NAT14	PEX19
SCCPDH	PEX19
AGPS	PEX5
MOV10	PFAS
GNB1	PFDN5
CUL2	PFDN5
ELOB	PFDN5
PSMD8	PFDN5
CUL2	PFKFB3
RBX1	PGAM5
GFER	PGPEP1

RAB5C	PHB
RAB2A	PHB
RAB7A	PHB
RAB2A	PHB2
RAB5C	PHB2
RAB7A	PHB2
HDAC2	PHB2
MRPS27	PHF8
BZW2	PHKG2
PABPC4	PHLDA1
ATP5MG	PHLDA3
SAAL1	PHLDA3
CEP250	PHLDB2
MARK2	PHLPP1
ECSIT	PI4K2A
ERP44	PIAS1
ATP6AP1	PICK1
RIPK1	PIDD1
RAB7A	PIEZO1
PIGS	PIGK
MOV10	PIGN
ATP1B3	PIGO
POPDC2	PIGO
APP	PIGO
PTH1R	PIGO
ART3	PIGO
MCOLN3	PIGO
GABRE	PIGO
DNAJA2	PIGS
TMEM131	PIGS
PIGS	PIGT
PIGS	PIGU
RALA	PIH1D2
MOV10	PIK3R4
MARK3	PIM1
CHPF	PIN1
PABPC1	PIN1
CSNK2A2	PIN1
MARK2	PINK1
TOMM70	PINK1
PABPC4	PINX1
RPL36	PINX1
ELOC	PINX1
PABPC1	PINX1
SRP72	PINX1
FAM98A	PINX1
DDX21	PINX1
CSNK2A2	PIP4K2A
CSNK2A2	PIP4K2C
MOV10	PIP5K1C
AP2A2	PIP5K1C
ARF6	PIP5K1C
DNAJC11	PITPNB
MOV10	PITPNB
CSNK2A2	PITPNM1
SIRT4	PITRM1
AP2M1	PJA1
PRKAR2A	PJA2
AP2M1	PJA2
PRKACA	PKIA
PRKACA	PKIG

RHOA	PKN1
MOV10	PKNOX1
GRN	PKP2
MATR3	PKP2
CUL3	PKP2
PTGER3	PKP2
DSG2	PKP2
PVR	PLA2G3
GOLGA2	PLAAT5
LAMA3	PLAT
LAMA1	PLAT
DCN	PLAT
PLAT	PLAU
ATP6V1A	PLBD1
SLC9A3R1	PLCB1
RALA	PLCD1
RHOA	PLCG1
RHOA	PLD1
RALA	PLD1
CUL7	PLD3
NSD2	PLEC
PLEKHA5	PLEC
CSDE1	PLEKHA3
PLEKHA5	PLEKHA5
UPF1	PLEKHA5
EXTL3	PLEKHA5
PROM1	PLEKHA5
PLEKHA5	PLEKHA6
DNMT1	PLEKHA6
UPF1	PLEKHB2
RTN4	PLEKHF2
GFER	PLEKHF2
MOV10	PLEKHF2
ZNF263	PLEKHF2
DTX2	PLEKHF2
RTN3	PLEKHF2
UBE2Z	PLEKHF2
THOC2	PLEKHF2
CHIC2	PLEKHF2
PRKAR1A	PLEKHF2
FBXO28	PLEKHF2
SFN	PLEKHF2
GNMT	PLEKHF2
NPM2	PLEKHF2
AIMP2	PLEKHF2
MBIP	PLEKHF2
SEPTIN2	PLEKHF2
CAVIN2	PLEKHF2
SEPTIN6	PLEKHF2
RTN1	PLEKHF2
SETBP1	PLEKHF2
APPL1	PLEKHF2
HSPB7	PLEKHF2
TNFAIP8	PLEKHF2
TXNL4B	PLEKHF2
SPEF1	PLEKHF2
BSCL2	PLEKHF2
GLUL	PLEKHF2
ELAVL1	PLEKHF2
MEAF6	PLEKHF2
NONO	PLEKHF2

RADX	PLEKHF2
TSC22D3	PLEKHF2
MAX	PLEKHF2
CCDC85B	PLEKHF2
APP	PLEKHF2
TBX3	PLEKHF2
GPBP1	PLEKHF2
CGGBP1	PLEKHF2
TRAPPC3	PLEKHF2
GPBP1L1	PLEKHF2
RSPO2	PLEKHF2
BEND7	PLEKHF2
USF1	PLEKHF2
TRAF6	PLEKHF2
FRMD8	PLEKHF2
DRAP1	PLEKHF2
PRPSAP1	PLEKHF2
RABAC1	PLEKHF2
ARL14EP	PLEKHF2
DUT	PLEKHF2
CEP44	PLEKHF2
BLOC1S6	PLEKHF2
RHOA	PLEKHG2
GNB1	PLEKHG2
CUL2	PLK1
NINL	PLK1
MIB1	PLK4
OS9	PLOD2
PLOD2	PLOD2
TERF2	PLOD2
TERF1	PLOD2
ITGB1BP2	PLOD2
FBXO6	PLOD2
VHL	PLOD2
MYC	PLOD2
UBL4A	PLOD2
ATG5	PLOD2
HNRNPA1	PLOD2
FOXA1	PLOD2
CSNK2A2	PLRG1
RETREG3	PLSCR1
EXOSC2	PLXNA3
RHOA	PLXNB1
LMAN2	PLXNB2
GFER	PMF1
RBX1	PML
COLGALT1	PML
MARK2	PMPCA
SIRT4	PMPCA
MRPS30	PMPCA
MARK3	PMPCB
SIRT4	PMPCB
ENO1	PMPCB
PPP4R3A	PMPCB
USP13	PMS1
NUP98	PNKD
RBM41	PNMA1
ZNF318	PNMA1
CSNK2A2	PNN
MOV10	PNP
MARK3	PNPLA2

ENO1	POFUT1
CUL2	POGLUT2
TAZ	POGLUT2
CUL3	POGLUT2
SUSD4	POGLUT3
FBXO6	POGLUT3
RAE1	POLA1
POLA2	POLA1
CDC45	POLA1
XRCC5	POLA1
KDM4A	POLA1
MCM2	POLA1
SMC1A	POLA1
ENO1	POLA1
UPF1	POLD1
CENPF	POLH
SUN2	POLR1E
CUL2	POLR2A
STC2	POLR2A
MOV10	POLR2A
MOGS	POLR2G
EXOSC5	POLR2L
PKP2	POLR3A
RAE1	POLR3A
STC2	POLR3F
G3BP2	POM121
GOLGA2	POM121
RETREG3	POMK
TMEM39B	POMK
GHITM	POMK
ALG5	POMK
NAT14	POMK
YIF1A	POMK
SCARB1	PON2
UPF1	POP1
CSNK2A2	POP1
CUL2	POP7
STAT1	POR
BTN2A1	POR
CYP2D6	POR
PGRMC1	POR
DERL2	POR
HMOX1	POT1
RTN4	POT1
PLAT	POT1
ATE1	POU2AF1
TUBGCP3	POU5F1
ERC1	POU5F1
FKBP7	POU5F1
POLA2	POU5F1
COLGALT1	POU5F1
ELOC	POU5F1
UPF1	POU5F1
MAP7D1	POU5F1
COMT	PPA2
CEP350	PPARA
RAB10	PPBP
CUL2	PPFIA1
RAB5C	PPIA
CSDE1	PPIA
MOV10	PPIB

OS9	PPIB
MEPCE	PPIH
TUBGCP3	PPIL1
SLU7	PPIL3
SGTB	PPIL3
ELAVL1	PPIL3
PRSS12	PPIL3
BAG6	PPIL3
SNCA	PPIL3
SYNCRIP	PPIL3
PCBP1	PPIL3
VPS50	PPIL3
BAG3	PPIL3
PIGS	PPM1A
PABPC4	PPM1B
TOR1AIP1	PPP1CA
MPHOSPH10	PPP1CA
ZDHHC5	PPP1CB
CSNK2B	PPP1CB
TOR1AIP1	PPP1CC
GOLGA7	PPP1CC
ZNF318	PPP1CC
PLEKHA5	PPP1R13B
NUTF2	PPP1R13L
TLE5	PPP1R16A
CUL2	PPP1R21
NDUFAF1	PPP1R21
ERC1	PPP1R21
STC2	PPP1R8
POLA1	PPP2CA
RALA	PPP2CA
PABPC1	PPP2CA
MARK3	PPP2CB
PLEKHA5	PPP2CB
RAB18	PPP2R1A
RAB7A	PPP2R1A
MARK2	PPP2R1A
PLEKHA5	PPP2R1A
RAB18	PPP2R1B
POLA1	PPP2R1B
RAB7A	PPP2R1B
RALA	PPP2R1B
ECSIT	PPP2R2A
GRPEL1	PPP2R2B
PABPC1	PPP2R2B
MRPS27	PPP2R2B
ECSIT	PPP2R2D
MARK2	PPP2R5E
PMPCB	PPP4R1
SLC25A13	PPT1
CANX	PPT1
PRDX1	PPT1
DBH	PPT1
CTSD	PPT1
SLC25A1	PPT1
PRDX5	PPT1
ATP5F1B	PPT1
VAPB	PPT1
CRMP1	PPT1
FBXO6	PPT1
RTN4	PRAF2

EXOSC2	PRC1
CUL2	PRDX1
PPT1	PRDX2
CUL2	PRDX2
ECSIT	PRDX2
MARK2	PRDX3
CUL2	PRDX3
NLRX1	PRDX3
CUL2	PRDX4
ADAMTS1	PRG2
FBN2	PRG2
DNAJC11	PRICKLE3
POLA2	PRIM1
MOV10	PRIM1
RAE1	PRIM1
POLA1	PRIM1
XRCC5	PRIM1
STN1	PRIM1
MMS19	PRIM1
RPA3	PRIM1
KLHL20	PRIM1
CHM	PRIM1
EGFR	PRIM1
COPS6	PRIM1
RPA2	PRIM1
NXF1	PRIM1
RPA1	PRIM1
CIAO2B	PRIM1
LIPH	PRIM1
MDC1	PRIM1
CIAO1	PRIM1
POLA2	PRIM2
RAE1	PRIM2
POLA1	PRIM2
STN1	PRIM2
GFOD1	PRIM2
PRPS2	PRIM2
SUV39H1	PRIM2
CIAO1	PRIM2
ELAVL1	PRIM2
KLHL20	PRIM2
KDM1A	PRIM2
SUN2	PRKAA1
MARK1	PRKAA1
TLE5	PRKAA1
MARK3	PRKAA1
MARK2	PRKAA1
G3BP1	PRKAA2
TLE5	PRKAA2
CSDE1	PRKAB1
GRPEL1	PRKAB1
RAB7A	PRKAB1
CYB5R3	PRKAB1
PABPC1	PRKAB1
PRKAR2B	PRKACA
KCNQ1	PRKACA
CUL3	PRKACA
AKAP11	PRKACA
BAD	PRKACA
RANBP9	PRKACA
DCAF7	PRKACB

AKAP9	PRKACB
PRKAR2B	PRKACB
CDK5RAP2	PRKACB
PRKACA	PRKACB
PRKAR2A	PRKACB
PRKACA	PRKAR1A
AP2M1	PRKAR1B
PRKACA	PRKAR1B
PRKAR2B	PRKAR1B
AKAP8	PRKAR2A
PRKACA	PRKAR2A
PRKAR2B	PRKAR2A
MAP1LC3A	PRKAR2A
OFD1	PRKAR2A
MAP2	PRKAR2A
CAV1	PRKAR2A
RUNX1T1	PRKAR2A
PDE4A	PRKAR2A
AKAP3	PRKAR2A
ELAVL1	PRKAR2A
GSK3B	PRKAR2A
MAPRE1	PRKAR2A
XPO1	PRKAR2A
WASF1	PRKAR2A
KCNQ1	PRKAR2A
AKAP11	PRKAR2A
DYNLL1	PRKAR2A
PRKAR2B	PRKAR2B
ITGB1	PRKCA
SLC9A3R1	PRKCA
MARK2	PRKCI
PITRM1	PRKCSH
MARK2	PRKD1
RAE1	PRKD2
RAE1	PRKD3
AP2M1	PRKDC
FOXRED2	PRKDC
RNF41	PRKN
STOML2	PRKN
PSMD8	PRKN
TOMM70	PRKN
IMPDH2	PRKN
BAG5	PRKN
CYB5R3	PRKN
MRPS27	PRKN
CHPF	PRKN
AGPS	PRKN
RPL36	PRKRA
PRKAR2B	PRKX
CUL2	PRMT1
CSNK2A2	PRMT1
SCARB1	PRMT2
TMED5	PRMT6
FYCO1	PRMT6
FKBP7	PRMT6
SLU7	PRMT6
TBK1	PROS1
CSNK2A2	PRPF19
TLE5	PRPF31
POLA2	PRPF38A
CUL2	PRPF39

TUBGCP2	PRPF4
CEP250	PRPF4
ERGIC1	PRPF4
FKBP15	PRPF4
CSNK2A2	PRPF40A
LARP7	PRPF40A
BRD4	PRPF40A
SNIP1	PRPF40A
AAR2	PRPF8
MEPCE	PRPF8
SLU7	PRPF8
DDX21	PRPF8
CDK5RAP2	PRPS1
NSD2	PRPSAP2
UPF1	PRR11
RTN4	PRR13
GPAA1	PRR13
CSNK2A2	PRR14L
TLE5	PRR3
EXOSC2	PRRC2A
TUBGCP3	PRRC2A
UBAP2L	PRRC2A
SMS	PRRC2B
EIF3I	PRRC2B
CD81	PRRC2B
ATN1	PRRC2B
PICALM	PRRC2B
CLEC11A	PRRC2B
USP11	PRRC2B
ATXN1	PRRC2B
CERK	PRRC2B
CSNK2A2	PRRC2C
NDUFB9	PRRT2
CEP250	PRSS1
TCF12	PRSS23
ECSIT	PSEN2
FBXL12	PSEN2
TUBGCP3	PSMA1
WFS1	PSMA5
RBX1	PSMA6
RAB7A	PSMA7
PSMD8	PSMB9
PSMD8	PSMC1
PSMD8	PSMC2
PSMD8	PSMC3
MOV10	PSMC3
PSMD8	PSMC6
MOV10	PSMD1
RHOA	PSMD10
PSMD8	PSMD13
PSMD8	PSMD14
BAG5	PSMD2
GNB1	PSMD2
HMOX1	PSMD2
MOV10	PSMD2
PSMD8	PSMD3
PSMD8	PSMD4
CUL2	PSMD4
CEP250	PSMD6
PSMD8	PSMD6
PSMD8	PSMD7

DVL2	PSMD8
PSMD5	PSMD8
NOS2	PSMD8
PSMC4	PSMD8
HUWE1	PSMD8
PSMC5	PSMD8
TP53BP1	PSMD8
TUBGCP3	PSME3
MOV10	PSME3IP1
REEP5	PSRC1
RPL36	PSTPIP1
BZW2	PSTPIP1
AKAP9	PSTPIP1
PTBP2	PTBP2
QKI	PTBP2
SNRPA	PTBP2
ELAVL1	PTBP2
TOM1	PTBP2
KHSRP	PTBP2
MATR3	PTBP2
FUS	PTBP2
HNRNPC	PTBP2
CDC5L	PTBP2
MRPS2	PTCD3
CEP250	PTCD3
TOR1AIP1	PTCH1
CHPF	PTCH1
HS6ST2	PTCH1
CSNK2A2	PTEN
SLC9A3R1	PTEN
G3BP2	PTEN
NDFIP2	PTEN
FBN2	PTEN
USP13	PTEN
INHBE	PTEN
PTGES2	PTGES2
MYC	PTGES2
NDFIP2	PTGFR
EXOSC2	PTGR2
SLC9A3R1	PTH1R
GNB1	PTK2
MDN1	PTP4A3
CUL2	PTP4A3
DNAJC11	PTP4A3
HEATR3	PTP4A3
MARK2	PTPA
STOM	PTPN1
NSD2	PTPN13
CUL2	PTPN14
MOV10	PTPN9
REEP6	PTPN9
PLAT	PTPRK
SCARB1	PTPRK
ERO1B	PTPRK
COL6A1	PTPRK
TLE5	PTRH2
MOV10	PTRHD1
RAE1	PTTG1
NUP98	PTTG1
PTBP2	PUF60
PABPC1	PUF60

AKAP9	PUF60
PABPC4	PUF60
G3BP1	PUF60
STOM	PUF60
MOV10	PUF60
RAB2A	PUF60
RAP1GDS1	PUF60
MEPCE	PURA
GFOD1	PUSL1
FOXF1	PUSL1
MRPL58	PUSL1
SKAP1	PUSL1
APP	PUSL1
WNK1	PVR
ELAVL1	PVR
PABPC1	PXN
MOV10	PYGO2
MOV10	PYM1
MOV10	QARS1
TCF12	QARS1
TLE5	QARS1
CUL2	QPCT
MOV10	QRICH1
NUP98	QRICH2
XRN1	QSOX2
ORC3	QSOX2
CEPT1	QSOX2
IMPACT	QSOX2
PICK1	QSOX2
NBEAL2	QSOX2
LYPD3	QSOX2
TTC1	QSOX2
FBXO30	QSOX2
C2CD5	QSOX2
TBC1D22A	QSOX2
GINM1	QSOX2
SMARCB1	QSOX2
TUT7	QSOX2
CPVL	QSOX2
PAN2	QSOX2
CEP44	QSOX2
MMS19	QSOX2
ARF6	QTRT1
MOV10	R3HCC1L
PABPC1	R3HCC1L
CUL7	RAB10
ELAVL1	RAB10
RAB14	RAB11FIP1
RAB14	RAB11FIP5
RPA3	RAB14
NUFIP1	RAB14
DGUOK	RAB14
UBL4A	RAB14
ATF2	RAB14
CUL7	RAB14
EED	RAB14
RPA2	RAB14
EXOC5	RAB14
RAB7A	RAB17
PTGER3	RAB18
PSMD7	RAB1A

ADCK1	RAB1A
ELAVL1	RAB1A
UBL4A	RAB1A
GTSE1	RAB1A
CAPNS1	RAB1A
RPA1	RAB1A
SART3	RAB1A
CREB1	RAB1A
UNK	RAB1A
RPA3	RAB1A
DBN1	RAB1A
ARRB1	RAB1A
MICAL1	RAB1A
RABEP1	RAB1A
MAPRE3	RAB1A
HIVEP1	RAB1A
RABAC1	RAB1A
CDKN1A	RAB1A
OCRL	RAB1A
LIMA1	RAB1A
SLC16A8	RAB1A
GOLGA5	RAB1A
TBC1D17	RAB1A
CUL7	RAB1A
RFK	RAB1A
ASB9	RAB1A
FSCN1	RAB1A
RPA2	RAB1A
RAB1A	RAB1B
GOLGA2	RAB1B
RAP1GDS1	RAB1B
BZW2	RAB1B
ATP6V1A	RAB22A
RAB7A	RAB22A
RAB7A	RAB2A
ICA1	RAB2A
CBFB	RAB2A
ACTB	RAB2A
ATP6V1H	RAB2A
TBC1D25	RAB2A
SOAT1	RAB2A
MAPK6	RAB2A
GDI2	RAB2A
RAP1GDS1	RAB31
PRKAR2A	RAB32
RTN4	RAB33A
GOLGA2	RAB33B
RALA	RAB34
GOLGA2	RAB39A
RAB8A	RAB3B
RAB8A	RAB3IL1
ELOC	RAB40A
ELOB	RAB40B
ELOB	RAB40C
ELOC	RAB40C
RAB5C	RAB43
SCCPDH	RAB4A
RAB7A	RAB4A
GRIPAP1	RAB4A
RAB14	RAB4B
RAB7A	RAB4B

RAB7A	RAB5A
SUN2	RAB5A
RAB5C	RAB5A
RAB7A	RAB5B
SUN2	RAB5B
SUN2	RAB5C
ATP6V0A1	RAB5C
CAV1	RAB5C
RPA3	RAB5C
CHMP2B	RAB5C
SLC38A5	RAB5C
LETMD1	RAB5C
GOSR1	RAB5C
ATP2C1	RAB5C
SOAT1	RAB5C
SGSM3	RAB5C
LIMA1	RAB5C
CHCHD3	RAB5C
PIEZO1	RAB5C
OSGEP	RAB5C
SCFD1	RAB5C
STX7	RAB5C
CUL7	RAB5C
TFRC	RAB5C
EEA1	RAB5C
CHMP4B	RAB5C
CLCN7	RAB5C
RAD51	RAB5C
TTC17	RAB5C
NDUFS1	RAB5C
ACBD5	RAB5C
GPRC5A	RAB5C
TBC1D17	RAB5C
NDUFB7	RAB5C
SUGP1	RAB5C
RAB7A	RAB6A
GCC2	RAB6A
GOLGA2	RAB6A
ERC1	RAB6A
ERC1	RAB6B
ATP6AP1	RAB7A
EED	RAB7A
ELAVL1	RAB7A
SOAT1	RAB7A
GTSE1	RAB7A
GPRC5A	RAB7A
ATG5	RAB7A
CUL7	RAB7A
VPS35	RAB7A
NDUFS1	RAB7A
LAP3	RAB7A
ATP2B1	RAB7A
GABARAPL2	RAB7A
NDUFB4	RAB7A
ATP6V0A1	RAB7A
CYBRD1	RAB7A
TFRC	RAB7A
VPS41	RAB7A
RAB10	RAB8A
EPB41	RAB8A
SRPRB	RAB8A

MYO5C	RAB8A
EXOC6	RAB8A
ODF2	RAB8A
GOLT1B	RAB8A
RPA2	RAB8A
MYO5B	RAB8A
OPTN	RAB8A
OCRL	RAB8A
STX3	RAB8A
TBC1D17	RAB8A
RPA1	RAB8A
RAB3IP	RAB8A
ELAVL1	RAB8A
RPA3	RAB8A
SYTL4	RAB8A
SYTL1	RAB8A
UBL4A	RAB8A
SNCA	RAB8A
TFRC	RAB8A
STAU1	RAB8A
PQBP1	RAB8A
GDI2	RAB8A
VAMP3	RAB8A
ITGB1	RAB8B
FOXRED2	RAB9A
NAT14	RABAC1
POR	RABEPK
HOOK1	RABGGTB
RAB1A	RABIF
POLA1	RABL2A
MOV10	RABL6
CIT	RAC1
ERC1	RACGAP1
PCNT	RACGAP1
ELOB	RACK1
ELOC	RACK1
SLC9A3R1	RACK1
ITGB1	RACK1
RHOA	RACK1
CUL2	RACK1
PABPC1	RACK1
LARP4B	RACK1
GNB1	RACK1
G3BP1	RAD21
PSMD8	RAD23A
ECSIT	RAD23A
USP13	RAD23A
NGLY1	RAD23A
ERC1	RAD23A
GCC2	RAD23A
CEP250	RAD23A
MOV10	RAD51D
DCAF7	RAD54L2
DNMT1	RAD9A
GNB1	RADIL
POLA2	RAE1
MYCBP2	RAE1
POLR3H	RAE1
CUL7	RAE1
CUL1	RAE1
DDX24	RAE1

CUL3	RAE1
SMC1A	RAE1
TACC3	RAE1
NUP188	RAE1
POLR3E	RAE1
DYNLL1	RAE1
SEH1L	RAE1
POLR3B	RAE1
UFD1	RAE1
FBXW11	RAE1
GABARAPL2	RAE1
SF3A1	RAE1
SNW1	RAE1
ERBB3	RAE1
CS	RAE1
KHSRP	RAE1
LOX	RAF1
ATP6V1A	RALB
RALA	RALBP1
AP2M1	RALGAPA1
CUL2	RAMAC
NEK9	RAN
NUTF2	RAN
MOV10	RANBP17
CUL2	RANBP2
NUP214	RANBP2
NUP58	RANBP2
GNB1	RAP1A
RHOA	RAP1GDS1
RAP1A	RAP1GDS1
ZNF451	RAP1GDS1
HDAC7	RAP1GDS1
RASL10A	RAP1GDS1
GNAZ	RAP1GDS1
CDC42	RAP1GDS1
RASD2	RAP1GDS1
KRAS	RAP1GDS1
RAC1	RAP1GDS1
GNAI1	RAP1GDS1
SNX24	RAP1GDS1
RAB34	RAP1GDS1
PLEKHB1	RAP1GDS1
KIFAP3	RAP1GDS1
CDK2	RAP1GDS1
SEPTIN6	RAP1GDS1
RRP9	RAP2A
MOV10	RAPGEF6
PITRM1	RARS2
GNB1	RASD1
GNB1	RASD2
RHOA	RASL10A
RHOA	RASSF1
G3BP1	RASSF10
HECTD1	RASSF6
RAB5C	RASSF8
G3BP1	RASSF9
CEP250	RAVER1
POLA1	RB1
DNMT1	RB1
DNMT1	RBBP4
CEP250	RBBP4

TUBGCP3	RBBP4
MIB1	RBBP4
BCS1L	RBFOX2
NSD2	RBM10
MOV10	RBM10
PTBP2	RBM10
CSNK2A2	RBM10
TLE5	RBM10
MOV10	RBM14
CUL2	RBM14
CEP250	RBM14
POLA2	RBM23
DNAJC11	RBM26
EED	RBM28
PRR11	RBM28
NOP56	RBM28
BRCA1	RBM28
GABARAPL2	RBM28
CUL1	RBM28
DHX8	RBM28
ESR1	RBM28
CUL3	RBM28
ZC3H3	RBM28
DNAJC11	RBM4
CUL2	RBM4
PTBP2	RBM4
PABPC1	RBM4
SPAG5	RBM41
TRAF1	RBM41
MAP7D1	RBM48
NSD2	RBM4B
MPHOSPH10	RBM4B
POLA1	RBMS1
MEPCE	RBMX
SEPSECS	RBPJ
TLE5	RBPMS
RAB5C	RBSN
RAB14	RBSN
UBE2D1	RBX1
PTGS2	RBX1
FBXO42	RBX1
MAP3K20	RBX1
CUL3	RBX1
CDC34	RBX1
TAB1	RBX1
ERCC8	RBX1
CUL7	RBX1
KEAP1	RBX1
ING3	RBX1
VRK2	RBX1
DCUN1D1	RBX1
ELAVL1	RBX1
MCM10	RBX1
MAPK8IP2	RBX1
SESN1	RBX1
ZC3HC1	RBX1
FBXW11	RBX1
ARID1B	RBX1
RNF126	RBX1
NEDD4	RBX1
MKNK2	RBX1

KDM2B	RBX1
ESR1	RBX1
CUL1	RBX1
IBTK	RBX1
ELOC	RCAN2
CUL2	RCBTB1
MOV10	RCC1
ARF6	RCC2
NINL	RCOR3
PHLPP2	RDX
MAPK10	RDX
PSME3	RDX
PLEK	RDX
EZR	RDX
ICAM2	RDX
UFD1	RDX
RNF2	RDX
GNA13	RDX
ESR1	RDX
BRCA1	RDX
ELAVL1	RDX
ACSL3	REEP5
REEP6	REEP5
LETMD1	REEP5
RAB5B	REEP5
SMARCA4	REEP5
TNFRSF10B	REEP5
ATP1B3	REEP5
DGKH	REEP5
GOLT1B	REEP5
MTHFSD	REEP5
GTF2H1	REEP5
MED17	REEP5
DHCR24	REEP5
DNAJC8	REEP5
DERL2	REEP5
RABAC1	REEP5
RGS2	REEP5
CNOT1	REEP5
GDE1	REEP5
ARFGAP1	REEP5
MCAT	REEP5
REEP6	REEP6
ZFYVE21	REEP6
TNS2	REEP6
TXN2	REEP6
SNX1	REEP6
RPS20	REEP6
SPG21	REEP6
FAM160A2	REEP6
EXOSC8	REL
TLE5	REL
EIF4E2	REL
EXOSC5	REL
AKAP8L	RELA
TLE5	RELA
BRD4	RELA
ECSIT	RELA
SNIP1	RELA
SIRT5	RELA
CUL2	RELA

CSNK2A2	RELA
HDAC2	REPIN1
CSNK2A2	REPS2
PRRC2B	RERE
SLC27A2	RETREG2
GOLGA3	RETREG2
NDUFAF1	RETREG3
REEP5	RETREG3
ELAVL1	RETREG3
GNPTG	RETREG3
ATP5PB	RETREG3
XYLT2	RETREG3
SLC35F2	RETREG3
POMGNT1	RETREG3
CD63	RETREG3
TTYH3	RETREG3
UBE2A	RETREG3
ATP9A	RETREG3
JPH1	RETREG3
TSPAN17	RETREG3
TSPAN15	RETREG3
ATP12A	RETREG3
RNF170	RETREG3
SEC62	RETREG3
MAP1LC3A	RETREG3
KTN1	RETREG3
STAP1	RETREG3
GABARAPL1	RETREG3
YIPF4	RETREG3
GNPTAB	RETREG3
BRD4	RFC4
MEPCE	RFC4
FYCO1	RFT1
MOV10	RFWD3
HYOU1	RFWD3
RALA	RGL2
RALA	RGL4
ELOB	RGMA
NINL	RGS2
COMT	RGS2
RAB2A	RGS2
DNMT1	RGS6
GNB1	RGS6
GOLGA2	RHNO1
DCUN1D1	RHOA
TBXA2R	RHOA
PKN2	RHOA
TRIO	RHOA
LIMA1	RHOA
CUL3	RHOA
DEF6	RHOA
RBX1	RHOB
CUL2	RHOB
CIT	RHOB
RBX1	RHOBTB3
CIT	RHOC
RALA	RHOF
RAB7A	RHOG
ATP6AP1	RHOU
RHOA	RHPN1
RHOA	RHPN2

NINL	RIBC2
DNAJC11	RIC3
SLC30A7	RIC3
SLC30A6	RIC3
RAB1A	RICTOR
GNB1	RICTOR
RBX1	RICTOR
RAB7A	RILP
VPS11	RILP
VPS39	RILP
MOV10	RILPL1
COMT	RIN1
CSNK2A2	RING1
RBM41	RINT1
RIPK1	RIPK1
CFLAR	RIPK1
EGLN3	RIPK1
HSP90AA1	RIPK1
TAX1BP1	RIPK1
RIPK3	RIPK1
FGL1	RIPK1
RFFL	RIPK1
RNF31	RIPK1
ITCH	RIPK1
IKBKB	RIPK1
TNFSF10	RIPK1
MAP3K8	RIPK1
CLIP3	RIPK1
MAP3K14	RIPK1
MAP3K7	RIPK1
TIMM50	RIPK1
ANXA1	RIPK1
HSPA8	RIPK1
USP4	RIPK1
TNFAIP3	RIPK1
DNAJA1	RIPK1
TNFRSF10B	RIPK1
SLC25A5	RIPK1
BIRC2	RIPK1
HSPA5	RIPK1
TAB2	RIPK1
KHDRBS1	RIPK1
RBCK1	RIPK1
RPL23	RIPK1
CASP8	RIPK1
FAS	RIPK1
AP1M2	RIPK1
CDC37	RIPK1
TNFRSF1A	RIPK1
RNF216	RIPK1
CASP10	RIPK1
DAB2IP	RIPK1
CAD	RIPK1
HERC2	RIPK1
RNF11	RIPK1
TICAM1	RIPK1
OPTN	RIPK1
PYCARD	RIPK1
HSP90AB1	RIPK1
XIAP	RIPK1
HSPA9	RIPK1

RCN2	RIPK1
USP2	RIPK1
TRADD	RIPK1
XPO1	RIPK1
CYLD	RIPK1
TRAF1	RIPK1
TRAF2	RIPK1
ZBP1	RIPK1
TRPC4AP	RIPK1
TUBB4A	RIPK1
TRAF5	RIPK1
RIPK2	RIPK1
TRAF3	RIPK1
TNFRSF1B	RIPK1
BIRC3	RIPK1
NUMBL	RIPK1
GNB1	RIPK3
RHOA	RIPK4
RHOA	RIPOR2
RAB5C	RMDN3
RETREG3	RMND1
PSMD8	RNF11
CSNK2A2	RNF111
MOV10	RNF123
GOLGA2	RNF135
SCAP	RNF139
MOV10	RNF150
NUP54	RNF166
NUP98	RNF166
NUP58	RNF166
CENPF	RNF166
VPS11	RNF181
SRP54	RNF185
PSMD8	RNF185
MOV10	RNF187
CUL2	RNF187
AKAP8	RNF2
COMT	RNF2
CSNK2A2	RNF2
PABPC4	RNF2
RAB10	RNF2
RBM28	RNF2
PKP2	RNF2
AKAP8L	RNF2
NUP88	RNF2
UPF1	RNF2
GOLGA2	RNF213
DNAJC11	RNF214
GOLGA2	RNF214
RAB2A	RNF26
CYB5R3	RNF26
STOM	RNF26
AKAP8L	RNF31
RNF41	RNF41
MARK2	RNF41
KIFC3	RNF41
UPF3A	RNF41
TTC1	RNF41
RFC4	RNF41
ERBB3	RNF41
TSG101	RNF41

N4BP3	RNF41
HOMER2	RNF41
UBE2Z	RNF41
ISCA2	RNF41
LPGAT1	RNF41
ERBB4	RNF41
RNF166	RNF41
RASIP1	RNF41
MARK4	RNF41
LZTS2	RNF41
USP8	RNF41
EVI5	RNF41
NPAT	RNF41
MTCL1	RNF41
UBE2D3	RNF41
LEPR	RNF41
SOGA1	RNF41
CLEC16A	RNF41
RFWD3	RNF41
ASB6	RNF41
CLASP1	RNF41
C1GALT1C1	RNF41
ADRB2	RNF41
BIRC6	RNF41
C1orf109	RNF41
MNAT1	RNF41
SF1	RNF41
APP	RNF41
CREB3	RNF41
P4HA1	RNF41
CACYBP	RNF41
TRIM32	RNF41
IL23A	RNF41
TRIM8	RNF41
RCN1	RNF41
NAV1	RNF41
UBE2K	RNF41
H3-3A	RNF41
ELAVL1	RNF41
SUGT1	RNF41
CEP192	RNF41
AKAP8L	RNF43
ELOB	RNF7
CUL2	RNF7
CUL2	RNPS1
CSNK2A2	RNPS1
ZC3H18	RNPS1
CSNK2B	RNPS1
UPF1	RNPS1
SNIP1	RNPS1
RHOA	ROCK1
RHOA	ROCK2
AKAP9	ROPN1L
MOV10	ROR2
RAB5C	RPA1
PABPC4	RPA1
EXOSC8	RPA1
RAB10	RPA1
RAB2A	RPA1
PABPC1	RPA1
AKAP8	RPA1

RAB7A	RPA1
ERGIC1	RPA1
RTN4	RPA1
FKBP15	RPA1
CSNK2A2	RPA1
RAB14	RPA1
POLA1	RPA1
PABPC4	RPA2
RAB7A	RPA2
PABPC1	RPA2
AKAP8	RPA2
POLA1	RPA2
CSNK2A2	RPA2
RAB2A	RPA2
RTN4	RPA2
ERGIC1	RPA2
RAB5C	RPA2
RAB10	RPA2
POLA1	RPA3
RAB2A	RPA3
CSNK2A2	RPA3
RAB10	RPA3
PABPC1	RPA3
RAB7A	RPA3
AKAP8	RPA3
PABPC4	RPA3
G3BP2	RPF2
RPL36	RPL10
G3BP2	RPL10
MEPCE	RPL10
MRPS27	RPL10
PABPC1	RPL10
CEP250	RPL10
TUBGCP3	RPL10
G3BP1	RPL10
LARP4B	RPL10
FOXRED2	RPL10
RBM28	RPL10
CEP250	RPL10A
DNMT1	RPL10A
CEP250	RPL11
TUBGCP3	RPL11
TUBGCP2	RPL11
CUL2	RPL11
UPF1	RPL11
CEP250	RPL12
CUL2	RPL12
MRPS27	RPL13
DNMT1	RPL13
MOV10	RPL13
CEP250	RPL13
MRPS5	RPL13
CUL2	RPL13
CEP250	RPL13A
CEP250	RPL14
DDX21	RPL14
NSD2	RPL14
RBM28	RPL14
CUL2	RPL14
CEP250	RPL15
CUL2	RPL15

TLE5	RPL18A
CUL2	RPL19
CUL2	RPL21
CUL2	RPL22
G3BP2	RPL22L1
CUL2	RPL23
TUBGCP3	RPL23A
CEP250	RPL23A
CUL2	RPL23A
CUL2	RPL24
TUBGCP3	RPL26
CUL2	RPL27
CEP250	RPL27
CUL2	RPL27A
CUL2	RPL28
CEP250	RPL30
CEP250	RPL35
CUL2	RPL36
EXOSC5	RPL36
CUL1	RPL36
RPL18A	RPL36
NOP56	RPL36
PRKAB1	RPL36
UBL4A	RPL36
EIF1B	RPL36
GABARAPL2	RPL36
CAND1	RPL36
ESR1	RPL36
CUL7	RPL36
ICAM1	RPL36
CDK2	RPL36
CUL3	RPL36
ITGA4	RPL36
RNF2	RPL36
RIPK2	RPL36
CDK5RAP3	RPL36
EZH2	RPL36
FN1	RPL36
TUBGCP3	RPL37A
AP2M1	RPL38
CUL2	RPL38
MEPCE	RPL38
CEP250	RPL4
CUL2	RPL4
CUL2	RPL5
UPF1	RPL6
CUL2	RPL7
CEP250	RPL7
UPF1	RPL7A
CEP250	RPL7A
NSD2	RPL8
RBM28	RPL8
TUBGCP3	RPL8
CEP250	RPL8
CUL2	RPL8
CCDC86	RPL8
CUL2	RPL9
CUL2	RPLP1
CUL2	RPLP2
CEP250	RPLP2
TUBGCP2	RPLP2

SCCPDH	RPN1
OS9	RPN1
FOXRED2	RPN1
RAB7A	RPN2
RHOA	RPN2
UPF1	RPRD2
CEP250	RPS11
CUL2	RPS13
MEPCE	RPS14
CEP250	RPS14
TUBGCP3	RPS14
CUL2	RPS14
CEP250	RPS15A
CUL2	RPS15A
EXOSC5	RPS16
TBK1	RPS18
CUL2	RPS18
CEP250	RPS18
TUBGCP3	RPS18
NGDN	RPS2
MPHOSPH10	RPS2
TUBGCP3	RPS2
CUL2	RPS2
CEP250	RPS2
NOL10	RPS2
CUL2	RPS23
CEP250	RPS23
CUL2	RPS24
CUL2	RPS25
TUBGCP3	RPS27
RTN4	RPS27
CUL2	RPS27
ARF6	RPS29
UPF1	RPS3
MEPCE	RPS3
TUBGCP2	RPS3
CEP250	RPS3
CUL2	RPS3
TUBGCP3	RPS3
CUL2	RPS3A
CEP250	RPS3A
CEP250	RPS4X
CUL2	RPS4X
MEPCE	RPS4X
TUBGCP3	RPS4X
UPF1	RPS6
CUL2	RPS6
CEP250	RPS6
TUBGCP3	RPS6
DNAJC11	RPS6KA5
RBX1	RPS6KB1
G3BP1	RPS6KB2
MOV10	RPS6KB2
EXOSC5	RPS7
TUBGCP3	RPS7
CUL2	RPS8
CEP250	RPS8
NGDN	RPS8
MRPS25	RPS8
MRPS27	RPS8
NOL10	RPS8

TUBGCP3	RPS8
TUBGCP3	RPS9
CUL2	RPS9
MEPCE	RPS9
CEP250	RPS9
GNB1	RPTOR
RAB1A	RPTOR
MOV10	RPUSD4
NPC2	RRAGA
ATP6V1A	RRAGC
ATP6V1A	RRAS
LARP1	RRP1B
CSNK2A2	RRP1B
EED	RRP9
FBL	RRP9
ZNF346	RRP9
CSNK2A1	RRP9
CUL7	RRP9
CSNK2A2	RSRC1
RPL36	RTCA
BCKDK	RTCA
RHOA	RTKN
RTN4	RTN1
RTN4	RTN2
RTN4	RTN3
CYB5R3	RTN4
RTN4	RTN4
HSDL1	RTN4
SCAMP1	RTN4
GOLT1B	RTN4
ZFYVE21	RTN4
WWTR1	RTN4
HUWE1	RTN4
DERL2	RTN4
RPA3	RTN4
TBL2	RTN4
CERT1	RTN4
LRCH4	RTN4
SIRT2	RTN4
SPG21	RTN4
CRMP1	RTN4
PRKAB1	RTN4
UQCRC1	RTN4
SH3PXD2A	RTN4
ATP1B3	RTN4
LAMA4	RTN4
SNX1	RTN4
NAPA	RTN4
CNTNAP1	RTN4
RTN4	RTN4IP1
GOLGA2	RTP5
MIPOL1	RTP5
PDE4DIP	RTP5
RAB14	RUFY1
DNMT1	RUNX1
TLE5	RUNX1
TCF12	RUNX1
GOLGA2	RUSC2
RAB1A	RUSC2
CEP250	RUVBL1
MEPCE	RUVBL1

PABPC1	RUVBL1
STOM	RUVBL1
UPF1	RUVBL1
ARF6	RUVBL1
MEPCE	RUVBL2
UPF1	RUVBL2
AP2M1	RUVBL2
STOM	RUVBL2
ARF6	RUVBL2
PLOD2	RUVBL2
CSNK2A2	RYBP
MIB1	RYK
MOV10	RYK
MOV10	S100A11
ATP6V1A	S100A11
PLEKHF2	S100A13
MOV10	S100A14
MOV10	S100A16
MOV10	S100A4
CNTRL	S100A4
RHOA	S100A4
TUBGCP3	S100A8
CUL2	S100A9
SELENOS	SAA1
MARK3	SAAL1
SCCPDH	SAAL1
SCN2B	SAAL1
ALDH3A2	SAAL1
EGFR	SAAL1
NEDD4	SAAL1
CA14	SAAL1
CD33	SAAL1
VSIG2	SAAL1
P2RX4	SAAL1
CD274	SAAL1
CD27	SAAL1
COMTD1	SAAL1
EFNB2	SAAL1
PNKD	SAAL1
TNFSF13B	SAAL1
CD68	SAAL1
PTGER3	SAAL1
GDF15	SAE1
AKAP9	SAMD3
RBX1	SAMHD1
CUL2	SAP130
ELOB	SAP130
CSNK2A2	SAP18
HDAC2	SAP25
MEPCE	SAP30BP
CSNK2A2	SAP30BP
MOV10	SAP30BP
CSNK2B	SARNP
RAE1	SART1
MEPCE	SART1
TUBGCP2	SCAF11
TUBGCP3	SCAF11
MOV10	SCAF8
GOLGA2	SCAF8
RTN4	SCAMP2
REEP5	SCAMP2

REEP5	SCAMP4
SREBF1	SCAP
MCOLN3	SCAP
NUDC	SCAP
BTNL8	SCAP
SAAL1	SCARA3
SUN2	SCARA3
MOV10	SCARA3
HEATR3	SCARA3
CUL1	SCARB1
ATP1B3	SCARB1
ATP6V1A	SCARB2
USP13	SCCPDH
MINDY2	SCCPDH
MIEN1	SCCPDH
ACTR1B	SCCPDH
WAPL	SCCPDH
BZW1	SCCPDH
DDB2	SCCPDH
PAIP2	SCCPDH
TMEM160	SCCPDH
DNAJB11	SCCPDH
KBTBD7	SCCPDH
HECTD3	SCCPDH
ZZEF1	SCCPDH
GRPEL1	SCLT1
TUBGCP3	SCN2B
BZW2	SCN2B
RETREG3	SCN2B
MDN1	SCN2B
MIPOL1	SCNM1
TLE5	SCNM1
TUBGCP3	SCO2
VPS11	SCRIB
MOV10	SCRIB
GORASP1	SCRN1
PRRC2B	SCRN2
CSNK2A2	SDAD1
CSNK2A2	SDC2
TLE5	SDCBP
REEP6	SDCBP
TUBGCP3	SDE2
DNAJB11	SDF2
CUL2	SDHB
NUP98	SEC13
PKP2	SEC16A
PRKAR2B	SEC16A
AKAP8L	SEC16A
PLEKHA5	SEC16A
CEP250	SEC16A
PRRC2B	SEC16A
SPART	SEC16A
PRKAR2A	SEC16A
SCAP	SEC24B
MOV10	SEC24C
TUBGCP3	SEC61G
GOLGA2	SECISBP2
ERLEC1	SEL1L
UBXN8	SELENOS
HERPUD1	SELENOS
APOB	SELENOS

DERL2	SELENOS
PTGS2	SELENOS
KPNB1	SELENOS
UBE4A	SELENOS
UFD1	SELENOS
CAV1	SELENOS
SELENOK	SELENOS
FAF2	SELENOS
MOV10	SELENOW
HECTD1	SEM1
MDN1	SEM1
PSMD8	SEM1
CUL2	SEM1
CDK5RAP2	SENP2
MOGS	SENP3
CSDE1	SENP3
MOV10	SEPHS2
XPO1	SEPSECS
CPPED1	SEPSECS
ABLIM1	SEPSECS
ARF6	SEPTIN2
PLEKHF2	SEPTIN5
STOM	SERINC2
GHITM	SERINC2
ERLEC1	SERPINA1
OS9	SERPINA1
PLAT	SERPINA5
PLAT	SERPINE1
OS9	SERPINH1
PLAT	SERPINI1
OS9	SERPINI1
RBX1	SERTAD1
ELOC	SERTAD1
RBX1	SESN2
MYCBP2	SESN3
NUP88	SET
GDF15	SETD4
DNMT1	SETD7
ECSIT	SETDB1
POLA2	SETDB1
HECTD1	SETDB1
ZC3H18	SF1
CSNK2A2	SF1
PIGS	SF3A2
RAE1	SF3A2
RAE1	SF3A3
PIGS	SF3B1
CUL2	SF3B3
GOLGA2	SFI1
MOV10	SFMBT2
MARK1	SFN
G3BP2	SFN
MARK3	SFN
RAE1	SFN
PKP2	SFN
DCAF7	SFN
G3BP1	SFN
MARK2	SFN
MYCBP2	SFN
EXOSC5	SFPQ
DNMT1	SFRP2

MOV10	SFXN4
FYCO1	SGF29
TCF12	SGF29
CEP250	SGF29
PABPC1	SGK1
FKBP7	SGTA
HYOU1	SGTB
SIRT5	SGTB
HEATR3	SGTB
ETFA	SGTB
ZNF318	SGTB
NINL	SH2D4A
PLEKHA5	SH3BP5L
NINL	SH3BP5L
TLE5	SH3GL3
PLEKHA5	SH3KBP1
ERC1	SH3KBP1
PABPC4	SH3KBP1
DNMT1	SH3KBP1
PABPC1	SH3KBP1
TUBGCP3	SH3KBP1
TUBGCP2	SH3KBP1
LOX	SH3KBP1
G3BP1	SH3RF2
CSNK2B	SHANK3
MOV10	SHC1
EDEM3	SHH
PTGES2	SHISA2
NUTF2	SHMT2
POLA1	SHMT2
INHBE	SHMT2
TUBGCP3	SHMT2
GRPEL1	SHMT2
AKAP8L	SHMT2
RAB8A	SHMT2
TCF12	SHMT2
PLEKHF2	SHMT2
CEP250	SHMT2
CUL2	SHMT2
PMPCB	SHMT2
VPS11	SHMT2
SCAP	SIAH2
USP13	SIAH2
PDZD11	SIGMAR1
ANK2	SIGMAR1
ITPR3	SIGMAR1
CFTR	SIGMAR1
CEP250	SIK2
PRKACA	SIK3
PON2	SIL1
SMAD9	SIL1
HSPA5	SIL1
CCND3	SIL1
FBXO6	SIL1
NSD2	SIN3A
NSD2	SIN3B
BRD4	SIPA1
CSNK2A2	SIRT1
SIRT5	SIRT3
GLS	SIRT5
HOXB5	SIRT5

IMPDH2	SIRT7
MPHOSPH10	SIRT7
MOV10	SIRT7
DDX10	SIRT7
RRP9	SIRT7
STOML2	SIRT7
ZC3H18	SIRT7
DNMT1	SIRT7
PABPC1	SIRT7
NUP214	SIRT7
NUP210	SIRT7
NOL10	SIRT7
NSD2	SIRT7
HYOU1	SIRT7
HECTD1	SIRT7
MDN1	SIRT7
CENPF	SIRT7
POLA1	SIRT7
NUP88	SIRT7
LARP4B	SIRT7
CCDC86	SIRT7
MYCBP2	SIRT7
ACSL3	SIRT7
PCNT	SIRT7
G3BP1	SIRT7
LARP7	SIRT7
DDX21	SIRT7
LARP1	SIRT7
CSDE1	SIRT7
NUP98	SIRT7
PRRC2B	SIRT7
UPF1	SIRT7
RBM28	SIRT7
CSNK2A2	SIRT7
UBAP2L	SIRT7
PABPC4	SIRT7
ZNF318	SIRT7
SRP72	SIRT7
TLE5	SIX1
ACSL3	SKA1
MYCBP2	SKA1
AP3B1	SKA3
POLA1	SKAP1
CUL2	SKAP1
TRMT1	SKAP1
MYCBP2	SKAP1
PABPC4	SKI
DCAF7	SKI
PABPC1	SKI
TLE5	SKIL
CHPF	SKIL
PMPCA	SKIV2L
RBX1	SKP1
USP13	SKP2
RBX1	SKP2
SLC9A3R1	SKP2
CUL2	SKP2
FBXL12	SLA
MYCBP2	SLAIN2
RAB18	SLAMF1
UPF1	SLBP

NDFIP2	SLC11A2
CUL2	SLC16A1
ERC1	SLC16A6
MOV10	SLC20A2
NOSIP	SLC25A21
FBXO6	SLC25A21
DNASE1L2	SLC25A21
PPIE	SLC25A21
UBXN8	SLC25A4
RAB14	SLC25A41
NARS2	SLC25A41
RALA	SLC25A41
AP2M1	SLC25A41
GNG5	SLC25A41
RAB2A	SLC25A41
AP2M1	SLC25A6
KPNB1	SLC27A2
PRKCSH	SLC27A2
YWHAQ	SLC27A2
PNPLA3	SLC27A2
CCNB1	SLC27A2
CTDSPL2	SLC27A2
ABCD1	SLC27A2
MINDY2	SLC27A2
HPS1	SLC27A2
ERLEC1	SLC27A3
FKBP10	SLC2A4
RAB7A	SLC2A4
SLC30A5	SLC30A6
ACTB	SLC30A6
CD40	SLC30A7
LYPD3	SLC30A7
ELAVL1	SLC30A7
ELOB	SLC33A1
RAB14	SLC33A1
SLC27A2	SLC33A1
MOV10	SLC35A4
RAB5C	SLC35E1
ATP6V1A	SLC35F6
UBXN8	SLC38A1
SLC27A2	SLC39A4
FAR2	SLC39A4
SCCPDH	SLC39A4
ITGB1	SLC3A2
ARF6	SLC3A2
RP2	SLC44A2
RETREG3	SLC4A2
MOV10	SLC4A2
UBXN8	SLC7A1
DNMT1	SLC7A11
SLC9A3R1	SLC9A3R1
NOS2	SLC9A3R1
GNA11	SLC9A3R1
ABCC2	SLC9A3R1
SLC9A3	SLC9A3R1
SLC4A7	SLC9A3R1
PHLPP2	SLC9A3R1
EZR	SLC9A3R1
PAG1	SLC9A3R1
TBC1D10A	SLC9A3R1
CFTR	SLC9A3R1

SLC4A8	SLC9A3R1
TCF3	SLC9A3R1
PRPF3	SLU7
XPO1	SLU7
BAG2	SLU7
SF3B2	SLU7
FOXJ2	SLU7
NSD3	SLU7
NXF1	SLU7
PPIG	SLU7
PRMT5	SLU7
JMJD6	SLU7
CEP70	SLU7
KDM1A	SLU7
ZCCHC10	SLU7
THAP1	SLU7
LZTS2	SLU7
MEPCE	SLX4
LARP7	SLX4
BAG5	SLX4
NUP214	SMAD1
SNIP1	SMAD1
GFER	SMAD2
RHOA	SMAD2
MTCH1	SMAD2
SNIP1	SMAD2
OS9	SMAD2
DCAF7	SMAD2
PSMD8	SMAD2
TCF12	SMAD2
NUP214	SMAD2
WASHC4	SMAD2
RBX1	SMAD3
TCF12	SMAD3
DCAF7	SMAD3
TLE5	SMAD3
PABPC1	SMAD3
NUP214	SMAD3
NUP214	SMAD4
RALA	SMAD4
CUL2	SMAD6
PSMD8	SMAD9
PABPC4	SMAD9
PKP2	SMAD9
RAB5C	SMARCA4
DNMT1	SMARCA5
MDN1	SMARCAD1
ZC3H18	SMARCAD1
RAB5C	SMARCC1
ARF6	SMARCC1
MOV10	SMARCC1
USP13	SMC1A
HECTD1	SMC2
RAE1	SMC3
USP13	SMC3
HECTD1	SMC4
MOV10	SMDT1
PABPC1	SMG1
UPF1	SMG1
MOV10	SMG5
UPF1	SMG5

UPF1	SMG6
UPF1	SMG7
UPF1	SMG8
UPF1	SMG9
MIB1	SMN1
SRP54	SMN1
ATP6V1A	SMN1
NSD2	SMN1
TLE1	SMN2
MIB1	SMN2
ATP6V1A	SMN2
SRP54	SMN2
HDAC2	SMN2
PDE4DIP	SMN2
GIGYF2	SMN2
NSD2	SMN2
NINL	SMNDC1
TAZ	SMOC1
PON2	SMOC1
TCF4	SMOC1
GINM1	SMOC1
MOV10	SMPD1
OS9	SMPD1
SLC27A2	SMPD1
CUL2	SMPD4
MOV10	SMTN
RAE1	SMU1
MRPS27	SMURF1
WFS1	SMURF1
GOLGA3	SMURF1
RHOA	SMURF1
DDX21	SMURF1
SPART	SMURF1
RHOA	SMURF2
AKAP9	SMYD2
TOR1A	SNAPIN
TOR1A	SNCA
G3BP1	SND1
LARP1	SNIP1
SRSF12	SNIP1
ZC3H14	SNIP1
GPALPP1	SNIP1
EIF4A3	SNIP1
SRSF1	SNIP1
CCNB1	SNIP1
DVL2	SNIP1
MYC	SNIP1
CDK6	SNIP1
MAGOH	SNIP1
CRNKL1	SNIP1
CLCN2	SNIP1
RBM22	SNIP1
THRAP3	SNIP1
CHERP	SNIP1
MAX	SNIP1
SRRT	SNIP1
TNIP1	SNIP1
YTHDC1	SNIP1
ZCCHC10	SNIP1
SRPK2	SNIP1
TRA2B	SNIP1

PRPF3	SNIP1
NCBP3	SNIP1
CAD	SNIP1
AQR	SNIP1
CCDC9	SNIP1
FERMT3	SNIP1
EP300	SNIP1
SF3B1	SNIP1
BUD31	SNIP1
MFAP1	SNIP1
CREBBP	SNIP1
MKRN1	SNIP1
NKTR	SNIP1
NCBP1	SNIP1
PPIG	SNIP1
ACIN1	SNIP1
CASC3	SNIP1
PNN	SNIP1
DROSHA	SNIP1
SNW1	SNIP1
SMAD4	SNIP1
BCLAF1	SNIP1
DHX8	SNIP1
TTC14	SNIP1
CCDC12	SNIP1
SRSF9	SNIP1
IK	SNIP1
SRSF6	SNIP1
NKAP	SNIP1
CLASRP	SNIP1
AAR2	SNRNP200
CUL2	SNRNP200
MOV10	SNRNP25
EXOSC8	SNRPB
UPF1	SNRPB
CUL2	SNRPB
CUL2	SNRPC
TLE5	SNRPC
EXOSC8	SNRPC
CUL2	SNRPD1
CUL2	SNRPD2
CUL2	SNRPE
CUL2	SNRPF
DNMT1	SNRPG
UPF1	SNRPN
MARK2	SNTB2
SELENOS	SNTG2
POLA2	SNU13
UPF1	SNW1
CSNK2B	SNX2
RAB7A	SNX3
CSNK2A2	SNX6
ELOB	SOCS1
ELOC	SOCS1
ELOB	SOCS2
ELOC	SOCS3
TBK1	SOCS3
ELOB	SOCS3
ELOB	SOCS4
ELOC	SOCS4
ELOC	SOCS6

POLA1	SOCS6
CDK5RAP2	SOCS6
ELOB	SOCS6
PLEKHA5	SOGA1
MARK2	SOGA1
CLIP4	SON
RBM28	SORT1
GOLGA3	SORT1
UGGT2	SORT1
UPF1	SORT1
SIRT5	SORT1
SRP54	SOX2
MEPCE	SOX2
SRP72	SOX2
TCF12	SOX2
LARP1	SOX2
CSNK2A2	SOX2
RAE1	SOX2
MDN1	SOX2
LOX	SOX2
SEPSECS	SOX2
PRKAR2B	SOX2
BAG5	SOX2
IMPDH2	SOX2
NUP214	SOX2
PLD3	SOX4
TLE5	SOX5
FKBP15	SP1
DNMT1	SP1
DNMT1	SP3
PRKAR2B	SPA17
GNB1	SPAG1
ACSL3	SPART
WWP1	SPART
ITCH	SPART
ACSL4	SPART
LMNB1	SPART
EPS15	SPART
KPNB1	SPART
MED20	SPART
SMURF2	SPART
NCL	SPART
PLIN3	SPART
VIM	SPART
COPA	SPART
HSPA5	SPART
HSPA9	SPART
HADHA	SPART
GOLGA3	SPATA6L
SLC27A2	SPC24
GCC2	SPC25
SLC30A9	SPDEF
NDUFB9	SPG7
CEP250	SPICE1
TUBGCP2	SPINT2
SLC30A7	SPINT2
ALG5	SPINT2
MOV10	SPINT2
SAAL1	SPN
NLRX1	SPNS1
CLCC1	SPNS3

RTN4	SPOP
PLD3	SPP1
CYB5R3	SPP1
GRIPAP1	SPP1
EIF4E2	SPRY2
ELOB	SPSB1
ELOC	SPSB1
ELOB	SPSB2
ELOC	SPSB3
MOV10	SPSB3
ELOC	SPSB4
ELOB	SPSB4
PLEKHA5	SPTAN1
PLEKHA5	SPTBN1
SNIP1	SPTY2D1
MOV10	SQSTM1
MARK2	SQSTM1
CUL2	SQSTM1
SCCPDH	SQSTM1
CSNK2A2	SQSTM1
RIPK1	SQSTM1
HDAC2	SRA1
TBK1	SRC
NDFIP2	SRC
OS9	SREBF2
SCAP	SREBF2
GOLGB1	SREBF2
PABPC1	SREK1
PABPC4	SREK1
RHOA	SRGAP1
SRP54	SRP14
SRP54	SRP54
CDC5L	SRP54
GEMIN2	SRP54
SNW1	SRP54
GEMIN5	SRP54
DDX20	SRP54
XPO1	SRP54
SRP54	SRP68
MEPCE	SRP68
MEPCE	SRP72
CUL3	SRP72
XPO1	SRP72
SKIL	SRP72
NIFK	SRP72
IBTK	SRP72
METTL18	SRP72
YBX1	SRP72
RNF2	SRP72
RPAP1	SRP72
HNRNPA1	SRP72
PYGM	SRP72
TARBP2	SRP72
SNW1	SRP72
BECN1	SRP72
HSPB1	SRP72
MCM2	SRP72
HDGF	SRP72
RPL10	SRP72
FCHSD2	SRP72
ILF2	SRP72

HNRNPU	SRP72
TULP3	SRP72
IFI16	SRP72
CDC5L	SRP72
STAU1	SRP72
CAND1	SRP72
ORC1	SRP72
SRP54	SRP9
PABPC1	SRPK1
PABPC4	SRPK2
TLE5	SRPK2
PABPC1	SRPK2
SELENOS	SRPK2
CCDC86	SRPK3
EXOSC5	SRPK3
DDX21	SRPK3
POR	SRPRB
HMOX1	SRPRB
RAB18	SRPRB
GOLGA3	SRR
CSNK2A2	SRRM1
DDX21	SRRM2
SLU7	SRRM2
SNIP1	SRRM2
CUL2	SRSF1
TUBGCP2	SRSF10
SNIP1	SRSF10
CUL2	SRSF3
CUL2	SRSF6
CUL2	SRSF7
RAB5C	SS18
CUL2	SSB
ARF6	SSR4
NEK9	SSRP1
MIB1	SSX2IP
SAAL1	ST6GALNAC3
ECSIT	STAMBPL1
RAB2A	STAMBPL1
CDK5RAP2	STARD9
USP13	STAT1
VPS39	STAT3
DNMT1	STAT3
BCKDK	STAT3
BRD4	STAT3
MOV10	STAT3
ERMP1	STAT5A
RAB5C	STAU1
PABPC1	STAU1
MOGS	STAU1
PABPC4	STAU1
GOLGA3	STAU1
ACSL3	STAU1
UPF1	STAU1
RAB10	STAU1
UPF1	STAU2
HIF1A	STC2
EIF2B3	STC2
PEBP1	STC2
POLR2C	STC2
AFF4	STC2
ELAVL1	STC2

ADAM11	STC2
PTN	STC2
ACSL3	STING1
TOMM70	STING1
TBK1	STING1
ABCC1	STING1
GNB1	STIP1
ERLEC1	STK11
PLD3	STK11
TLE5	STK16
GORASP1	STK25
RAE1	STK3
CDK5RAP2	STK36
RHOA	STK39
PABPC1	STK4
ERC1	STK4
SRP54	STK4
POLA2	STK40
POLA1	STN1
ATP6V1A	STOM
STOM	STOM
CUL2	STOM
RAB2A	STOM
RAB7A	STOM
ATIC	STOM
FAF2	STOM
VAPA	STOM
GIMAP2	STOM
FN1	STOM
TRIP6	STOM
DBN1	STOM
MYH9	STOM
ASIC2	STOM
TMPO	STOM
ESR1	STOM
PNKD	STOM
EGFR	STOM
GOLT1B	STOM
LIMA1	STOM
SUMO1	STOM
ERGIC3	STOM
SLC2A1	STOM
FBXO6	STOM
ITGA4	STOM
RAPGEF2	STOM
CLTC	STOM
ATP6V1B1	STOM
FAP	STOM
ASIC1	STOM
FGFR1OP2	STOM
RPL13A	STOM
RAB5C	STOML2
RAB7A	STOML2
VAPA	STOML2
UBC	STOML2
APP	STOML2
YWHAE	STOML2
ELAVL1	STOML2
CUL7	STOML2
FBXO6	STOML2
CCNA1	STOML2

MYEF2	STOML2
GOLT1B	STOML2
GLP1R	STOML2
ECT2	STOML2
PRKAR1A	STOML2
HAUS2	STOML2
SIRT4	STOML2
COPS5	STOML2
FTSJ1	STOML2
ATG5	STOML2
PAXIP1	STOML2
NEFM	STOML2
OBSL1	STOML2
TOR1A	STON2
CSDE1	STRAP
TCF12	STRN3
CSDE1	STT3A
RAB7A	STT3B
TLE5	STX11
GORASP1	STX12
G3BP1	STX3
ATP6V1A	STX8
AP2M1	STX8
DNAJC11	STXBP1
TMEM97	STXBP3
FBXL12	STYX
TLE5	STYXL1
UBXN8	SUCLA2
BCKDK	SUMO1
MDN1	SUMO2
DDX21	SUMO2
STOM	SUMO3
FAM98A	SUMO3
MOV10	SUMO3
SUN2	SUN2
LIMA1	SUN2
ACTN1	SUN2
RPS20	SUN2
CFLAR	SUN2
SYNE2	SUN2
FBXW11	SUN2
TP53BP1	SUN2
MOV10	SUPT4H1
LMAN2	SUPT5H
MOV10	SUPT5H
PABPC4	SURF2
PRKAR2B	SURF4
UBXN8	SURF4
AKAP9	SUV39H2
RBX1	SUZ12
ERGIC1	SUZ12
NGDN	SUZ12
PCNT	SUZ12
G3BP2	SUZ12
AKAP9	SUZ12
LARP1	SUZ12
STOML2	SUZ12
AKAP8L	SUZ12
PABPC4	SUZ12
NUP88	SUZ12
CDK5RAP2	SUZ12

PRKAR2A	SUZ12
DNMT1	SUZ12
UPF1	SUZ12
CENPF	SVIL
STOM	SVIL
GNB1	SVIL
SELENOS	SVIP
HOOK1	SYCE1
USP13	SYNCRIP
CUL2	SYNCRIP
GOLGA3	SYNCRIP
SUN2	SYNE1
DNAJC11	SYNE4
CSNK2A2	SYNM
AP2M1	SYNPO
ZDHHC5	SYNPO
MOV10	SYNPO
GNB1	SYNPO
RHOA	SYNPO2
NINL	SYT17
RAB10	SYTL3
WFS1	SYVN1
OS9	SYVN1
ERLEC1	SYVN1
SELENOS	SYVN1
HMOX1	SYVN1
POR	SYVN1
UBXN8	SYVN1
FAM8A1	SYVN1
ATP6V1A	TACSTD2
CLIP4	TAF1
SCCPDH	TAF1
GOLGA3	TAF10
PRKAR2B	TAF12
CSNK2A2	TAF1D
MOV10	TAGLN2
TCF12	TAL1
ARF6	TALDO1
MARK2	TAOK1
MOV10	TAPBP
RIC3	TAPT1
SUCO	TAPT1
TXNDC15	TAPT1
RRP9	TARBP2
TRMT1	TARBP2
RBM28	TARBP2
SRP54	TARBP2
PABPC1	TARDBP
UPF1	TARDBP
PABPC4	TARDBP
CUL2	TARDBP
MRPS27	TARDBP
FAM98A	TARDBP
FASTKD3	TARS2
CUL3	TARS2
CDC37	TARS2
AHSA1	TARS2
CHORDC1	TARS2
AP2M1	TASOR
MOV10	TATDN2
RAB18	TAX1BP1

RAB5C	TBC1D15
CUL2	TBC1D15
UBXN8	TBC1D15
MOV10	TBC1D16
UBXN8	TBC1D17
CUL2	TBC1D4
HOOK1	TBC1D7
HMGXB4	TBCA
OTUB1	TBCA
TERF2IP	TBCA
TBK1	TBK1
TOMM70	TBK1
MIB1	TBK1
TUBB4A	TBK1
YWHAE	TBK1
RBBP5	TBK1
TICAM1	TBK1
DTX4	TBK1
PPP6R1	TBK1
CALM3	TBK1
HSPA5	TBK1
SKP1	TBK1
HMMR	TBK1
FXR2	TBK1
PPP6R2	TBK1
TXLNA	TBK1
STX3	TBK1
CCT6A	TBK1
TANK	TBK1
RPL11	TBK1
TRAF3	TBK1
STX12	TBK1
STAT6	TBK1
HSPA9	TBK1
HSP90AA1	TBK1
HNRNPM	TBK1
LGALS3BP	TBK1
FMR1	TBK1
GAPDH	TBK1
CYLD	TBK1
SYK	TBK1
NCK1	TBK1
TAX1BP1	TBK1
RARA	TBK1
MCM2	TBK1
CCT2	TBK1
HERC2	TBK1
SFPQ	TBK1
TNFAIP3	TBK1
HSPA8	TBK1
RUVBL2	TBK1
PPP6C	TBK1
TRAF1	TBK1
STX11	TBK1
TLR4	TBK1
TCP1	TBK1
CDC42BPG	TBK1
RPS8	TBK1
TNIP1	TBK1
TXLNG	TBK1
RPS5	TBK1

CDC37	TBK1
CCT8	TBK1
RPL7	TBK1
EGFR	TBK1
BBOX1	TBK1
SCLT1	TBK1
HSP90AB1	TBK1
MAP1LC3B	TBK1
AKT1	TBK1
AZI2	TBK1
NEDD4	TBK1
APBA3	TBK1
CCT4	TBK1
RPL13	TBK1
DDX58	TBK1
CUL1	TBK1
XIAP	TBK1
CCT7	TBK1
APP	TBK1
RB1CC1	TBK1
ATP5F1A	TBK1
LDHB	TBK1
CCT3	TBK1
CALCOCO2	TBK1
ESR1	TBK1
EXOC2	TBK1
LETM1	TBK1
RUVBL1	TBK1
BTRC	TBK1
NCOA2	TBK1
CCT5	TBK1
TNIP3	TBK1
IFIT3	TBK1
XPO1	TBK1
TLR3	TBK1
CALM2	TBK1
USP2	TBK1
IRF3	TBK1
MTPAP	TBK1
NONO	TBK1
STX19	TBK1
NFKB1	TBK1
OPTN	TBK1
RNF11	TBK1
NFKB2	TBK1
REL	TBK1
CCDC8	TBK1
DYRK2	TBK1
MAVS	TBK1
TRAF3IP2	TBK1
AGO2	TBK1
TRAF2	TBK1
RELA	TBK1
CEP250	TBKBP1
TBKBP1	TBKBP1
TBK1	TBKBP1
FXR2	TBKBP1
MCM2	TBKBP1
OBSL1	TBKBP1
APBA3	TBKBP1
AZI2	TBKBP1

FMR1	TBKBP1
AGO2	TBKBP1
RB1CC1	TBKBP1
CALCOCO2	TBKBP1
EXOSC5	TBL3
TLE5	TBX3
DNAJC11	TBX3
CEP250	TCAF1
USP13	TCAF1
GOLGA2	TCEA2
MARK3	TCEA2
EXOSC8	TCEA2
GOLGA2	TCEANC
NINL	TCEANC
TCF12	TCF12
BMF	TCF12
TSNAX	TCF12
ELAVL1	TCF12
EP300	TCF12
TAF4	TCF12
XPO1	TCF12
PSMA1	TCF12
TBP	TCF12
TCF3	TCF12
ARMC8	TCF12
FRMD6	TCF12
ID3	TCF12
ID1	TCF12
TCF21	TCF12
SRI	TCF12
ZWINT	TCF12
C1orf109	TCF12
STAT5A	TCF12
CREBBP	TCF12
ID2	TCF12
MAPKBP1	TCF12
RNASEL	TCF12
STK16	TCF12
CDKN2C	TCF12
EDRF1	TCF12
RUNX1T1	TCF12
SPG21	TCF12
NAGK	TCF12
LAMTOR5	TCF12
MYC	TCF12
TCF12	TCF4
TLE5	TCF4
EIF4E2	TCF4
CSNK2A2	TCOF1
GTF2F2	TCTEX1D2
CSNK2B	TCTEX1D2
PABPC1	TDRD3
GOLGA2	TEAD4
PDE4DIP	TEAD4
FYCO1	TECPR1
EXOSC5	TENT4B
CENPF	TERF1
PABPC4	TERF1
HMOX1	TERF1
GRPEL1	TERF1
RTN4	TERF1

CSNK2A2	TERF1
TRMT1	TERF1
WFS1	TERF1
AASS	TERF2
PITRM1	TERF2
MARK3	TERF2
BCKDK	TERF2
PLAT	TERF2
PABPC4	TERF2
CSNK2A2	TERF2
CENPF	TERF2
GGCX	TERF2IP
TOR1AIP1	TERF2IP
HMOX1	TEX264
TOR1A	TEX29
UPF1	TF
CUL2	TFG
ERGIC1	TFG
GORASP1	TGFA
COLGALT1	TGM2
CSNK2A2	TGM2
ITGB1	TGOLN2
RAB14	TGOLN2
INTS4	TGOLN2
RAB18	TGOLN2
GGH	TGOLN2
MDN1	TGOLN2
AKAP9	THAP1
GOLGA2	THAP7
MRPS27	THOC2
MOV10	THOC7
CUL2	TIA1
CUL2	TIAL1
NSD2	TIAM1
PVR	TIGIT
TIMM9	TIMM10
MOV10	TIMM22
TIMM9	TIMM22
TIMM10	TIMM22
AP4S1	TIMM29
FAF2	TIMM29
APOE	TIMM29
FAS	TIMM29
RBFOX2	TIMM29
HADHB	TIMM8B
TIMM13	TIMM8B
RAE1	TJP1
RAE1	TJP2
EXOSC5	TJP2
ATP6V1A	TK1
PLD3	TK1
MOV10	TK1
TLE5	TLE1
TLE1	TLE1
FOXA3	TLE1
VENTX	TLE1
TSC22D1	TLE1
DUSP1	TLE1
CTNNB1	TLE1
SIRT1	TLE1
RUNX2	TLE1

UTY	TLE1
PRDM1	TLE1
RUNX3	TLE1
VHL	TLE1
PFN1	TLE1
NOD2	TLE1
DAZAP2	TLE1
DLEU1	TLE1
FOS	TLE1
UBTF	TLE1
RPA2	TLE1
ZFP64	TLE1
EED	TLE1
ATN1	TLE1
POLE2	TLE1
FXYD6	TLE1
CDKN2C	TLE1
BCL2L1	TLE1
FUBP1	TLE1
ESRRG	TLE1
HESX1	TLE1
NUDT21	TLE1
BTBD2	TLE1
MSX2	TLE1
POLB	TLE1
PEX2	TLE1
RERE	TLE1
SNRPG	TLE1
BID	TLE1
TCF3	TLE1
GSK3B	TLE1
PAFAH1B3	TLE1
MPHOSPH6	TLE1
KDM6A	TLE1
FGL1	TLE1
GSTM4	TLE1
ARL3	TLE1
HDAC3	TLE1
PLSCR4	TLE1
HDAC1	TLE1
NUPR1	TLE1
RUNX1	TLE1
ATF2	TLE1
HMGB1	TLE1
TK1	TLE1
HSPE1	TLE1
HES6	TLE1
FOXA2	TLE1
SS18	TLE1
MEIS2	TLE1
HLA-DQA1	TLE1
EGR1	TLE1
RNF10	TLE1
SMN1	TLE1
APH1A	TLE1
RELA	TLE1
ARL4D	TLE1
RCC1	TLE1
ERH	TLE1
SUZ12	TLE1
EZH2	TLE1

STARD7	TLE1
SERPINB9	TLE1
CUL4B	TLE1
ANXA7	TLE1
HES1	TLE1
EIF2S2	TLE1
HHEX	TLE1
GADD45A	TLE1
ATF3	TLE1
FOXA1	TLE1
SAT1	TLE1
QPRT	TLE1
CDKN1A	TLE1
RAP1B	TLE1
TCF4	TLE1
HOXA1	TLE1
IL6ST	TLE1
CDKN2A	TLE1
TLE2	TLE1
LEF1	TLE1
GRB7	TLE1
PSMD11	TLE1
TLE5	TLE3
ANXA1	TLE3
NDRG1	TLE3
QPRT	TLE3
LHX6	TLE3
PIN1	TLE3
STARD7	TLE3
PEX7	TLE3
FGL1	TLE3
NLK	TLE3
GRN	TLE3
TLE5	TLE5
CCNK	TLE5
RNF31	TLE5
U2AF2	TLE5
MVP	TLE5
CAPN1	TLE5
L3MBTL2	TLE5
SNCAIP	TLE5
NCDN	TLE5
ELAVL1	TLE5
TRAF1	TLE5
BIRC7	TLE5
RIPK1	TLR3
NRG1	TM2D3
C17orf80	TM2D3
IL9R	TM2D3
SPCS1	TM2D3
DDX39A	TM2D3
CLSTN1	TM2D3
BTNL8	TM2D3
OAF	TM2D3
PAXIP1	TM2D3
TSPAN17	TM2D3
SPCS2	TM2D3
G3BP1	TMA16
EMC1	TMBIM4
SUN2	TMBIM4
TLE5	TMCC2

TMED5	TMED10
RAB5C	TMED10
GORASP1	TMED3
MOV10	TMED3
MOV10	TMED4
MOV10	TMED9
RAB2A	TMEM109
MOV10	TMEM11
CSNK2A2	TMEM126A
NDUFAF1	TMEM126B
ECSIT	TMEM126B
SELENOS	TMEM129
MOV10	TMEM141
MOV10	TMEM164
UBXN8	TMEM165
MOV10	TMEM184A
ECSIT	TMEM186
ERP44	TMEM189
ATP6V1A	TMEM192
MOV10	TMEM222
NDFIP2	TMEM231
PLAT	TMEM25
PCSK6	TMEM25
RAB7A	TMEM43
RTN4	TMEM43
ATP6V1A	TMEM63B
ECSIT	TMEM70
RTN4	TMEM70
ELAVL1	TMEM97
NIN	TMOD1
ATP6V1A	TMOD3
EXOSC8	TMPO
AKAP8L	TMPO
TLE1	TMSB4X
RAB7A	TMUB1
HS6ST2	TNF
TM2D3	TNF
RIPK1	TNF
RAB18	TNF
TBK1	TNF
EXOSC5	TNFAIP1
RHOA	TNFAIP1
PABPC4	TNFRSF10D
CSDE1	TNFRSF10D
HYOU1	TNFRSF10D
RPL36	TNFRSF14
RIPK1	TNFRSF25
AKAP9	TNIK
EMC1	TNIK
NINL	TNNT1
CLIP4	TNNT3
PABPC1	TNRC6A
PABPC1	TNRC6B
PABPC1	TNRC6C
PABPC1	TOB1
PABPC1	TOB2
RAB18	TOM1L1
MOV10	TOMM20
MOV10	TOMM40L
TRADD	TOMM70
HSPB1	TOMM70

UNK	TOMM70
MAVS	TOMM70
STAU1	TOMM70
SNAPIN	TOMM70
IRF3	TOMM70
RNF185	TOMM70
HSP90AA1	TOMM70
MCM2	TOMM70
DDX21	TOP1
NUP58	TOP1MT
CUL2	TOP2A
MIB1	TOP3A
TOR1A	TOR1A
LRRC7	TOR1A
TOR1B	TOR1A
CANX	TOR1A
FBXO2	TOR1A
FAM20B	TOR1A
ELAVL1	TOR1A
CUL7	TOR1A
ASPHD2	TOR1A
HSD11B1	TOR1A
TOR1A	TOR1AIP1
COPS7A	TOR1AIP1
CHCHD3	TOR1AIP1
GTF2IRD1	TOR1AIP1
TERF2	TOR1AIP1
MREG	TOR1AIP1
NARF	TOR1AIP1
PPM1A	TOR1AIP1
EIF4G1	TOR1AIP1
PIGT	TOR1AIP1
ELAVL1	TOR1AIP1
TXN	TOR1AIP1
TOR1A	TOR1AIP2
TOR1A	TOR3A
RAE1	TP53
HECTD1	TP53
MRPS27	TP53
PABPC1	TP53
DNMT1	TP53
POLA1	TP53
G3BP2	TP53
EIF4E2	TP53
MRPS25	TP53
DCAF7	TP53
RBX1	TP53
MARK2	TP53
EXOSC8	TP53
MOGS	TP53
MRPS2	TP53
NUTF2	TP53BP2
PRKACA	TP73
CLIP4	TPM1
GOLGA3	TPM1
MRPS27	TRA2A
PABPC4	TRA2A
RRP9	TRA2A
MRPS2	TRA2A
MOV10	TRA2A
MEPCE	TRA2A

MRPS5	TRA2A
UPF1	TRA2A
LARP1	TRA2A
MRPS25	TRA2A
SNIP1	TRA2A
CUL2	TRA2B
DNAJC11	TRABD
RBM41	TRAF2
GOLGA3	TRAF2
TLE5	TRAF2
ABCC1	TRAF3
EIF4E2	TRAF6
CYB5B	TRAF6
RAB5C	TRAF6
RAB10	TRAF6
CSDE1	TRAF6
NLRX1	TRAF6
RTN4	TRAF6
RAB7A	TRAF6
G3BP1	TRAF6
RPL36	TRAF6
HYOU1	TRAF6
PSMD8	TRAF6
HEATR3	TRAF6
DDX21	TRAF6
TOMM70	TRAF6
ELOC	TRAF6
PABPC1	TRAF6
ETFA	TRAF6
TBK1	TRAIP
CEP250	TRAP1
DNAJC11	TRAP1
MOV10	TRAPPC9
TBK1	TRIM10
MOV10	TRIM16
TBK1	TRIM26
MEPCE	TRIM26
TLE5	TRIM26
MTCH1	TRIM26
DNMT1	TRIM27
MAT2B	TRIM27
RBX1	TRIM27
CEP135	TRIM27
RBM41	TRIM27
TBK1	TRIM27
EIF4E2	TRIM27
BAG5	TRIM27
TLE5	TRIM27
NSD2	TRIM28
CUL2	TRIM28
CEP250	TRIM28
CLIP4	TRIM32
TCF12	TRIM33
TLE5	TRIM41
CSNK2A2	TRIM41
CUL2	TRIM44
MOV10	TRIM56
USP13	TRIM63
RBX1	TRIM74
MEPCE	TRIP12
RHOA	TRIP6

MRPS27	TRMT10B
MRPS2	TRMT10B
MRPS5	TRMT10B
MRPS25	TRMT10B
PKP2	TRMT10C
CUL2	TRMT112
ARF6	TRMT112
UPF1	TRMT112
ECSIT	TRMT61B
RHOA	TRPC1
RBX1	TRPC4AP
RAB7A	TRPM7
FYCO1	TRUB2
MOV10	TSC1
RALA	TSC2
ELOC	TSC22D4
RHOA	TSC22D4
RAP1GDS1	TSC22D4
TLE5	TSC22D4
MOV10	TSEN54
UPF1	TSG101
PABPC1	TSG101
TLE5	TSGA10
GOLGA3	TSNAX
MRPS27	TSNAX
NDFIP2	TSPAN3
PDZD11	TSPAN33
ITGB1	TSPAN4
SLC27A2	TSPAN5
NAT14	TSPAN5
RETREG3	TSPAN5
CYB5R3	TSPAN5
ALG8	TSPAN5
CSNK2A2	TSPYL2
NINL	TSPYL4
AAR2	TSSC4
GOLGA2	TSSC4
AP2M1	TSSC4
TLE5	TSSK3
FKBP15	TTBK2
GOLGA2	TTC25
RAB5C	TTC3
CSNK2A2	TTC3
MOV10	TTC39C
GCC2	TTC4
AAR2	TTC5
HECTD1	TTLL1
MOV10	TTLL11
CSNK2A2	TTLL12
CLIP4	TTN
NEK9	TTN
MYCBP2	TTN
SUN2	TTN
CUL2	TTN
ITGB1	TUBA1A
TUBGCP2	TUBA1A
TUBGCP3	TUBA1A
CEP250	TUBA1A
AP2M1	TUBA1A
SPART	TUBA1A
RBX1	TUBA1B

AP2M1	TUBA1C
CUL2	TUBA1C
ATP5MG	TUBA1C
NEK9	TUBA1C
CEP250	TUBA1C
MAP7D1	TUBA1C
CUL2	TUBA4A
CEP250	TUBB
RIPK1	TUBB
TUBGCP3	TUBB
TUBGCP2	TUBB
AP2M1	TUBB
CUL2	TUBB
TBK1	TUBB
CUL2	TUBB2A
GFER	TUBB2A
TUBGCP2	TUBB2A
CEP250	TUBB2B
TUBGCP3	TUBB4B
AP2M1	TUBB4B
CUL2	TUBB4B
CEP250	TUBB4B
TUBGCP2	TUBB4B
MOV10	TUBB6
TUBGCP2	TUBG1
TUBGCP3	TUBG1
NINL	TUBG1
CEP250	TUBG1
PRKACA	TUBG1
TUBGCP3	TUBGCP2
EMD	TUBGCP2
CAPNS1	TUBGCP2
SCO2	TUBGCP2
PHKA1	TUBGCP2
HSPA8	TUBGCP2
LGALS3BP	TUBGCP2
TUBA1B	TUBGCP2
HSPA5	TUBGCP2
TUBB4A	TUBGCP2
RARS1	TUBGCP2
ACTB	TUBGCP2
HSPA9	TUBGCP2
XPO1	TUBGCP2
CDC5L	TUBGCP2
PTGER3	TUBGCP2
TUBGCP6	TUBGCP2
RPS20	TUBGCP2
RPS19	TUBGCP2
HSP90AB1	TUBGCP2
PCGF1	TUBGCP2
RAD51	TUBGCP2
PHKA2	TUBGCP2
SNW1	TUBGCP2
BAG2	TUBGCP2
TUBA4A	TUBGCP2
HECTD1	TUBGCP3
MRPS2	TUBGCP3
RCN2	TUBGCP3
RPS15	TUBGCP3
MAGED2	TUBGCP3
SKP1	TUBGCP3

CDC5L	TUBGCP3
RPL23	TUBGCP3
PRICKLE3	TUBGCP3
ELAVL1	TUBGCP3
SUPT6H	TUBGCP3
DDX5	TUBGCP3
ESS2	TUBGCP3
UBR5	TUBGCP3
LGALS3BP	TUBGCP3
ACSL4	TUBGCP3
RPS25	TUBGCP3
FBXW11	TUBGCP3
HSP90AB1	TUBGCP3
SNW1	TUBGCP3
RPL24	TUBGCP3
CDK17	TUBGCP3
DNAJB6	TUBGCP3
XPO1	TUBGCP3
PPP3CB	TUBGCP3
PRPF19	TUBGCP3
HSPA8	TUBGCP3
ACTB	TUBGCP3
DYNC1I2	TUBGCP3
RPL31	TUBGCP3
HSPA5	TUBGCP3
MTMR1	TUBGCP3
RPS10	TUBGCP3
MRPS14	TUBGCP3
MRPS7	TUBGCP3
DOCK7	TUBGCP3
NINL	TUBGCP4
TUBGCP2	TUBGCP4
TUBGCP3	TUBGCP4
TUBGCP3	TUBGCP6
CUL2	TUFM
MEPCE	TUT1
CUL2	TUT4
ERC1	TWF2
TCF12	TWIST2
CUL2	TXN
EXOSC8	TXNDC17
HDAC2	TXNDC5
XPO1	TYSND1
PEX5	TYSND1
PABPC1	U2AF1
MEPCE	U2AF1
SUN2	UBA1
EMC1	UBAC2
RAB7A	UBAC2
MOV10	UBAP1
RBFOX2	UBAP2
FXR1	UBAP2
CUL3	UBAP2
CDKL5	UBAP2
FUS	UBAP2
QPCT	UBAP2
KLHL20	UBAP2
GLUL	UBAP2
ITCH	UBAP2
ATXN1	UBAP2L
PRMT1	UBAP2L

MED4	UBAP2L
COPS5	UBAP2L
MLH1	UBAP2L
FBXW11	UBAP2L
ITGA4	UBAP2L
FN1	UBAP2L
PLXNA2	UBAP2L
ELAVL1	UBAP2L
ETFBKMT	UBAP2L
NEDD4	UBAP2L
FUS	UBAP2L
KAT2A	UBAP2L
PMS1	UBAP2L
XPO1	UBAP2L
ESR1	UBAP2L
NLK	UBAP2L
OBSL1	UBAP2L
CUL3	UBAP2L
ITCH	UBAP2L
SPART	UBB
TUBGCP2	UBB
CEP250	UBB
MDN1	UBC
G3BP1	UBC
FKBP15	UBC
EXOSC2	UBC
PSMD8	UBC
HECTD1	UBC
SPART	UBC
TMEM39B	UBC
CUL2	UBC
CYB5R3	UBC
IDE	UBC
G3BP2	UBC
TUBGCP2	UBC
EXOSC8	UBC
PABPC4	UBC
USP13	UBC
AAR2	UBC
RIPK1	UBC
CSNK2A2	UBC
RHOA	UBC
MYCBP2	UBC
MIB1	UBC
STOM	UBC
RAE1	UBC
MIB1	UBE2D2
RBX1	UBE2D2
RBX1	UBE2D3
MIB1	UBE2D3
NDUFB9	UBE2E1
UBAP2L	UBE2E3
RBX1	UBE2E3
RBX1	UBE2G1
RBX1	UBE2G2
ERLEC1	UBE2G2
CSDE1	UBE2I
OS9	UBE2J1
GHITM	UBE2J1
ERLEC1	UBE2J1
RBX1	UBE2L3

EIF4E2	UBE2L3
RBX1	UBE2L6
CUL2	UBE2M
RBX1	UBE2M
MIB1	UBE2N
RBX1	UBE2R2
ERP44	UBE2V1
RHOA	UBE2V2
MIB1	UBE2W
MOV10	UBE2Z
GOLGA3	UBE3A
MYCBP2	UBE3A
RAB10	UBL4A
PABPC1	UBL4A
USP13	UBL4A
PABPC4	UBL4A
SIL1	UBQLN1
GNB1	UBQLN1
FKBP7	UBQLN4
CUL2	UBR1
CEP250	UBR4
TUBGCP3	UBR4
PABPC1	UBR5
CUL2	UBXN1
RAE1	UBXN10
TLE5	UBXN11
EIF4E2	UBXN11
NGLY1	UBXN2B
POR	UBXN6
MOV10	UBXN6
ELOC	UBXN7
ELOB	UBXN7
CUL2	UBXN7
RBX1	UBXN7
VDAC3	UBXN8
RHBDD2	UBXN8
MSH2	UBXN8
UFD1	UBXN8
RAB7A	UCHL5
CYB5B	UCHL5
PSMD8	UCHL5
USP13	UFD1
TUBGCP3	UGGT1
APPBP2	UGGT2
HMOX1	UGT8
DNMT1	UHRF2
ERP44	UIMC1
RAB1A	ULK1
G3BP1	ULK1
POLA2	ULK2
MOV10	UNC119B
ABCC1	UNC93B1
PRRC2B	UNK
NUP98	UNK
RAB14	UNK
EIF4H	UNK
CSDE1	UNK
CUL2	UNK
RTN4	UNK
UPF1	UPF1
EXOSC2	UPF2

EXOSC8	UPF2
ITGB1	UPF2
UPF1	UPF2
EXOSC2	UPF3A
UPF1	UPF3A
UPF1	UPF3B
TUBGCP2	UQCC2
MOV10	UQCR10
RTN4	UQCRC2
MOV10	UQCRFS1
RAB7A	UQCRQ
MOV10	URM1
CEP68	USHBP1
AKAP9	USHBP1
AKAP9	USO1
RAB1A	USO1
PRKACA	USO1
PRKAR2B	USO1
CEP68	USO1
TOR1AIP1	USP1
PABPC1	USP10
RAE1	USP11
MYCBP2	USP11
PSMD8	USP14
MOV10	USP18
SAAL1	USP19
SUN2	USP20
MAP7D1	USP20
ACADM	USP20
MARK3	USP21
MARK1	USP21
MARK2	USP21
RIPK1	USP21
CUL2	USP25
PRRC2B	USP3
ERP44	USP32
RALA	USP33
MEPCE	USP39
CDK5RAP2	USP43
ERP44	USP46
PRKACA	USP49
USP13	USP5
EIF4E2	USP54
CHMP2A	USP54
OLFM4	USP54
ATXN1	USP54
CHMP1A	USP54
ATN1	USP54
NXT2	USP54
CLEC11A	USP54
SKAP1	USP54
CHMP4B	USP54
CCNJL	USP54
CHMP2B	USP54
CHMP3	USP54
CHMP4C	USP54
CARD8	USP54
CHMP5	USP54
FBXW11	USP54
EXOSC8	USP6
COMT	USP6NL

NUP98	USP7
MTCH1	USP7
CEP112	USP7
DNMT1	USP7
GOLGB1	USP7
RAE1	USP7
CHMP2A	USP8
MIB1	USP9X
NINL	UTP14A
MARK2	UTRN
VPS39	UVRAG
HECTD1	UVRAG
VPS11	UVRAG
ATP6V1A	VAMP7
ATP6V1A	VAMP8
PRKACA	VAPA
PRKACA	VAPB
PRKAR2B	VAPB
PRKAR2B	VAV2
RHOA	VAV3
UPF1	VCAM1
SCAP	VCAM1
CHMP2A	VCAM1
RAB10	VCAM1
GNB1	VCAM1
STOM	VCAM1
UBAP2L	VCAM1
DNMT1	VCAM1
AP2M1	VCAM1
NUP210	VCAM1
NUP214	VCAM1
MDN1	VCAM1
LARP1	VCAM1
EIF4H	VCAM1
RAB5C	VCAM1
RPL36	VCAM1
ITGB1	VCAM1
GOLGA7	VCAM1
PABPC1	VCAM1
PPT1	VCAM1
PABPC4	VCAM1
G3BP1	VCAM1
RAB10	VCP
MARK2	VCP
CUL2	VCP
ITGB1	VCP
RAB7A	VCP
NUP54	VCP
USP13	VCP
OS9	VCP
NUP58	VCP
DCAF7	VCP
COMT	VCP
HOOK1	VCP
NGLY1	VCP
G3BP2	VCP
SELENOS	VCP
TUBGCP2	VCP
PRKAR2A	VCP
RAB14	VCP
MDN1	VCP

FKBP15	VCP
PPT1	VCP
BZW2	VCP
UBXN8	VCP
MOV10	VCPIP1
UBXN8	VCPIP1
ACAD9	VDAC1
MOV10	VDAC1
FOXRED2	VDAC1
RAB5C	VDAC1
CSNK2B	VDAC1
RAB2A	VDAC1
RAB7A	VDAC1
EMC1	VDAC2
RAB5C	VDAC2
FOXRED2	VDAC2
RAB7A	VDAC2
PPT1	VDAC2
CUL2	VHL
ATE1	VHL
COLGALT1	VHL
ELOB	VHL
RAB2A	VHL
RBX1	VHL
RAB7A	VHL
RAB5C	VHL
GRPEL1	VHL
RAB14	VHL
COMT	VKORC1
REEP5	VKORC1
MOV10	VKORC1L1
MAP7D1	VOPP1
RAB7A	VPS11
MOV10	VPS11
MARK3	VPS11
RDX	VPS11
VPS41	VPS11
EZR	VPS11
KDM1A	VPS11
STX7	VPS11
BCAR1	VPS11
ECPAS	VPS11
VPS8	VPS11
TRIM32	VPS11
LRSAM1	VPS11
STX17	VPS11
RNF166	VPS11
CAMSAP3	VPS11
NOTCH1	VPS11
MON1B	VPS11
MSN	VPS11
TGFBRAP1	VPS11
VPS33A	VPS11
TSG101	VPS11
VPS18	VPS11
RAB7A	VPS16
HOOK1	VPS16
VPS11	VPS16
RAB7A	VPS18
FKBP15	VPS26A
RAB7A	VPS26A

BCS1L	VPS26A
FKBP15	VPS26B
MYCBP2	VPS26B
ATE1	VPS26B
WASHC4	VPS26B
TCF12	VPS28
RAB7A	VPS29
TLE5	VPS37B
GOLGA2	VPS37C
TLE5	VPS37C
HOOK1	VPS39
VPS11	VPS39
RAB7A	VPS39
VPS39	VPS39
STX17	VPS39
CDK2	VPS39
EPN3	VPS39
AP3D1	VPS39
NOTCH1	VPS39
CHMP2A	VPS4A
RALA	VPS4B
RNF41	VPS52
MTCH1	VSIG4
FAR2	VSIG4
TUBGCP3	VSIG4
SLC30A9	VSIG4
RAB18	VSIG4
INTS4	VSIG4
PVR	VTN
CUL2	VWA7
PRKACA	WASF1
WASHC4	WASHC1
FKBP15	WASHC2C
WASHC4	WASHC2C
FKBP15	WASHC4
RPA1	WASHC4
VPS26A	WASHC4
RPA3	WASHC4
RPA2	WASHC4
XPO1	WASHC4
WASHC4	WASHC5
FKBP15	WASHC5
FAM8A1	WBP1
SLC30A7	WBP1
PABPC1	WBP4
NUP54	WDFY2
NUP58	WDFY2
MOV10	WDFY3
RAB5C	WDFY3
MDN1	WDR12
RAE1	WDR24
GNB1	WDR26
RAE1	WDR26
MARK2	WDR46
CSNK2A2	WDR46
EXOSC2	WDR46
MYCBP2	WDR48
CSNK2A2	WDR48
CSNK2A2	WDR5
RAE1	WDR59
EXOSC5	WDR75

RAE1	WDR75
CUL2	WDR77
NSD2	WDR77
MOV10	WDR81
BCKDK	WDTC1
CEP350	WEE1
MAPK6	WFS1
CFTR	WFS1
CSDE1	WNK1
ELOC	WNT7B
ELOB	WSB1
DNMT1	WT1
CSNK2A2	WWOX
PABPC4	WWOX
PABPC1	WWOX
UBAP2	WWOX
SPART	WWOX
DDX21	WWOX
DCTPP1	WWOX
DCAF7	WWOX
CSDE1	WWOX
RTN4	WWP1
NDFIP2	WWP2
MOV10	WWP2
SPART	WWP2
PABPC1	WWP2
TUBGCP2	WWP2
ERC1	XIAP
CEP135	XKR4
CSNK2A2	XPC
POLA2	XPNPEP1
AKAP8L	XPO1
PLEKHA5	XPO1
CSNK2A2	XPO1
EXOSC5	XPO1
UPF1	XPO1
CSDE1	XPO1
GNB1	XPO1
PRKACA	XPO1
DNAJC11	XPO1
POLA2	XPO1
PABPC1	XPO1
RAB7A	XPR1
RAB5C	XPR1
MOV10	XRCC6
POR	XRCC6
PABPC4	XRN1
UPF1	XRN1
MOV10	XXYLT1
NAT14	YAE1
CUL2	YAE1
ERP44	YAP1
RAE1	YAP1
SLC9A3R1	YAP1
UPF1	YBX1
CSDE1	YBX1
MOV10	YDJC
UBAP2L	YEATS2
SLC9A3R1	YES1
ACSL3	YES1
DPEP2	YIF1A

SLAMF7	YIF1A
CACNG4	YIF1A
CDK2AP2	YIF1A
YIPF5	YIF1A
VKORC1	YIF1A
NCSTN	YIF1A
VAPA	YIF1A
ATP13A2	YIF1A
RAB5C	YIPF5
RAB7A	YIPF5
PIGS	YIPF5
MEPCE	YRDC
G3BP1	YTHDF1
MFGE8	YTHDF1
MEPCE	YTHDF2
MYCBP2	YWHAB
LARP1	YWHAB
PLEKHA5	YWHAB
MARK1	YWHAB
USP54	YWHAB
PKP2	YWHAB
DCAF7	YWHAB
CRTC3	YWHAB
MARK2	YWHAB
MARK3	YWHAB
EIF4E2	YWHAB
ITGB1	YWHAB
HECTD1	YWHAB
PKP2	YWHAE
MARK3	YWHAE
MARK2	YWHAE
ERC1	YWHAG
MYCBP2	YWHAG
PABPC1	YWHAG
MARK3	YWHAG
DCAF7	YWHAG
LARP1	YWHAG
ZC3H18	YWHAG
CRTC3	YWHAG
MARK2	YWHAG
MARK1	YWHAG
MYCBP2	YWHAH
ERMP1	YWHAH
PDZD11	YWHAH
RAE1	YWHAH
MARK1	YWHAH
MARK2	YWHAH
CEP250	YWHAH
MARK3	YWHAH
PKP2	YWHAH
CEP250	YWHAQ
PABPC4	YWHAQ
UPF1	YWHAQ
CUL2	YWHAQ
MYCBP2	YWHAQ
PKP2	YWHAQ
MARK2	YWHAQ
MRPS27	YWHAQ
HECTD1	YWHAQ
TUBGCP3	YWHAQ
DNMT1	YWHAQ

RAE1	YWHAQ
MARK3	YWHAQ
MEPCE	YWHAZ
MARK2	YWHAZ
AP3B1	YWHAZ
PABPC4	YWHAZ
MARK1	YWHAZ
PCNT	YWHAZ
MOV10	YWHAZ
DCAF7	YWHAZ
MYCBP2	YWHAZ
LARP1	YWHAZ
CUL2	YWHAZ
CWC27	YWHAZ
MARK3	YWHAZ
MIB1	ZBTB10
TLE5	ZBTB24
MRPS5	ZBTB38
EIF4E2	ZBTB9
CSNK2A2	ZC3H18
MOV10	ZC3H18
PTGER3	ZC3H18
CUL3	ZC3H18
EPB41L3	ZC3H18
CCNA2	ZC3H18
TERF2	ZC3H18
LUC7L2	ZC3H18
TERF1	ZC3H18
MYC	ZC3H18
LUC7L	ZC3H18
SF3A2	ZC3H18
FERMT3	ZC3H18
CARNMT1	ZC3H18
OSBPL10	ZC3H18
UPF1	ZC3H3
STK3	ZC3H7A
BIRC7	ZC3H7A
COL7A1	ZC3H7A
XPO1	ZC3H7A
RAB5C	ZCRB1
GOLGA2	ZDHHC17
YIF1A	ZDHHC17
DBN1	ZDHHC5
BRCA1	ZDHHC5
IQGAP1	ZDHHC5
LIMA1	ZDHHC5
CLDND1	ZDHHC5
GOLGA7B	ZDHHC5
REEP5	ZDHHC7
CUL2	ZER1
PRRC2B	ZER1
ELOB	ZER1
ELOC	ZER1
NINL	ZFC3H1
MRPS25	ZFC3H1
MIB1	ZFC3H1
NINL	ZFHX3
GOLGA2	ZFP2
NUP214	ZFP36
EXOSC8	ZFP36
PABPC1	ZFP36

FBN1	ZFP41
FBN2	ZFP41
SPART	ZFYVE9
PRKAR2B	ZG16B
REEP5	ZMYM3
GGH	ZMYM3
CSNK2A2	ZMYND8
CSNK2A2	ZNF106
CLIP4	ZNF114
GOLGA2	ZNF124
CSNK2A2	ZNF219
MOV10	ZNF248
PLEKHF2	ZNF250
NINL	ZNF250
HOOK1	ZNF250
GOLGA2	ZNF250
CARD8	ZNF318
AXIN1	ZNF318
MIER2	ZNF318
MCM2	ZNF318
CCDC8	ZNF318
MMS19	ZNF318
FAM160B2	ZNF318
ZBTB46	ZNF318
LIPH	ZNF318
AR	ZNF318
FBL	ZNF318
MDM2	ZNF318
PLEKHF2	ZNF398
EXOSC2	ZNF408
GOLGA2	ZNF417
NINL	ZNF417
NINL	ZNF426
MOV10	ZNF460
HDAC2	ZNF461
SLC9A3R1	ZNF468
CYP1A1	ZNF503
DCAF15	ZNF503
MED23	ZNF503
KLC1	ZNF503
CEP170	ZNF503
HOXA1	ZNF503
CCNJL	ZNF503
CGRRF1	ZNF503
PDE4DIP	ZNF512B
OS9	ZNF512B
MOV10	ZNF544
MOV10	ZNF558
EXOSC5	ZNF558
MOV10	ZNF561
GOLGA2	ZNF572
TLE5	ZNF576
GOLGA2	ZNF581
GOLGA2	ZNF587
CSNK2A2	ZNF592
BRD4	ZNF592
RAB1A	ZNF593
GOLGA2	ZNF594
EIF4E2	ZNF598
MOV10	ZNF618
HECTD1	ZNF622

MOV10	ZNF623
NINL	ZNF646
MOV10	ZNF672
CSNK2A2	ZNF687
MRPS27	ZNF707
PRKAR2B	ZNF707
MRPS2	ZNF707
MRPS25	ZNF707
MRPS5	ZNF707
MOV10	ZNF772
GOLGA2	ZNF774
MOV10	ZNF780A
HOOK1	ZNF785
CSDE1	ZNFX1
AAR2	ZNHIT2
UBAP2L	ZP3
CUL2	ZRANB3
HMOX1	ZSCAN20
MRPS27	ZSCAN26
PDE4DIP	ZSCAN26
CUL2	ZYG11B
ELOB	ZYG11B
ELOC	ZYG11B
COPS5	ZYG11B
ELAVL1	ZYG11B
CLEC11A	ZYG11B
GPR137B	ZYG11B
HDAC7	ZYG11B
NECAP2	ZYG11B
NEDD8	ZYG11B
CDCA5	ZYG11B
PRKAR2A	AKAP2
PRKAR2B	AKAP2
PRKACA	AKAP2
