# Supplementary material for: Lung disease network reveals impact of comorbidity on SARS-CoV-2 infection and opportunities of drug repurposing
Source: BMC Med Genomics. 2021 Sep 17;14:226. doi: 10.1186/s12920-021-01079-7 (PMC8447809; doi:10.1186/s12920-021-01079-7)
Supplement: Supplementary file 3 — Additional file 3. Table S2. Disease-gene association data of lungs from the ORGANizer database. [file 12920_2021_1079_MOESM3_ESM.pdf]

Supplementary Table2: Disease-gene association data of lungs from the ORGANizer database

gene	disorder
BUB1	Abnormal lung lobation
BUB1B	Abnormal lung lobation
BUB3	Abnormal lung lobation
CEP57	Abnormal lung lobation
CERS1	Abnormal lung lobation
DHCR7	Abnormal lung lobation
EMG1	Abnormal lung lobation
FOXF1	Abnormal lung lobation
FRAS1	Abnormal lung lobation
FREM2	Abnormal lung lobation
GABRD	Abnormal lung lobation
GDF1	Abnormal lung lobation
GLI3	Abnormal lung lobation
GRIP1	Abnormal lung lobation
HYLS1	Abnormal lung lobation
KCNAB2	Abnormal lung lobation
KIF7	Abnormal lung lobation
LASS1	Abnormal lung lobation
LBR	Abnormal lung lobation
PRDM16	Abnormal lung lobation
RARB	Abnormal lung lobation
SKI	Abnormal lung lobation
STRA6	Abnormal lung lobation
WNT3	Abnormal lung lobation
WT1	Abnormal lung lobation
ARMC4	Abnormal respiratory motile cilium morphology
C14ORF104	Abnormal respiratory motile cilium morphology
C19ORF51	Abnormal respiratory motile cilium morphology
C21ORF59	Abnormal respiratory motile cilium morphology

C2ORF39	Abnormal respiratory motile cilium morphology
CCDC103	Abnormal respiratory motile cilium morphology
CCDC114	Abnormal respiratory motile cilium morphology
CCDC151	Abnormal respiratory motile cilium morphology
CCDC39	Abnormal respiratory motile cilium morphology
CCDC40	Abnormal respiratory motile cilium morphology
CCDC65	Abnormal respiratory motile cilium morphology
CCNO	Abnormal respiratory motile cilium morphology
DNAAF1	Abnormal respiratory motile cilium morphology
DNAAF2	Abnormal respiratory motile cilium morphology
DNAAF3	Abnormal respiratory motile cilium morphology
DNAAF5	Abnormal respiratory motile cilium morphology
DNAH11	Abnormal respiratory motile cilium morphology
DNAH5	Abnormal respiratory motile cilium morphology
DNAI1	Abnormal respiratory motile cilium morphology
DNAI2	Abnormal respiratory motile cilium morphology
DNAL1	Abnormal respiratory motile cilium morphology
DRC1	Abnormal respiratory motile cilium morphology
DYX1C1	Abnormal respiratory motile cilium morphology
FAM187A	Abnormal respiratory motile cilium morphology
GAS8	Abnormal respiratory motile cilium morphology
HEATR2	Abnormal respiratory motile cilium morphology
HYDIN	Abnormal respiratory motile cilium morphology
LRRC50	Abnormal respiratory motile cilium morphology
LRRC6	Abnormal respiratory motile cilium morphology
NME8	Abnormal respiratory motile cilium morphology
OFD1	Abnormal respiratory motile cilium morphology
RPGR	Abnormal respiratory motile cilium morphology
RSPH1	Abnormal respiratory motile cilium morphology
RSPH3	Abnormal respiratory motile cilium morphology
RSPH4A	Abnormal respiratory motile cilium morphology

RSPH9	Abnormal respiratory motile cilium morphology
SPAG1	Abnormal respiratory motile cilium morphology
TXNDC3	Abnormal respiratory motile cilium morphology
ZMYND10	Abnormal respiratory motile cilium morphology
EIF4A3	Abnormality of the aryepiglottic fold
FOXC2	Abnormality of the pulmonary vasculature
PORCN	Abnormality of the pulmonary vasculature
FOXF1	Abnormality of the pulmonary veins
ARMC4	Absent respiratory ciliary axoneme radial spokes
C14ORF104	Absent respiratory ciliary axoneme radial spokes
C19ORF51	Absent respiratory ciliary axoneme radial spokes
C21ORF59	Absent respiratory ciliary axoneme radial spokes
C2ORF39	Absent respiratory ciliary axoneme radial spokes
CCDC103	Absent respiratory ciliary axoneme radial spokes
CCDC114	Absent respiratory ciliary axoneme radial spokes
CCDC151	Absent respiratory ciliary axoneme radial spokes
CCDC39	Absent respiratory ciliary axoneme radial spokes
CCDC40	Absent respiratory ciliary axoneme radial spokes
CCDC65	Absent respiratory ciliary axoneme radial spokes
CCNO	Absent respiratory ciliary axoneme radial spokes
DNAAF1	Absent respiratory ciliary axoneme radial spokes
DNAAF2	Absent respiratory ciliary axoneme radial spokes
DNAAF3	Absent respiratory ciliary axoneme radial spokes
DNAAF5	Absent respiratory ciliary axoneme radial spokes
DNAH11	Absent respiratory ciliary axoneme radial spokes
DNAH5	Absent respiratory ciliary axoneme radial spokes
DNAI1	Absent respiratory ciliary axoneme radial spokes
DNAI2	Absent respiratory ciliary axoneme radial spokes
DNAL1	Absent respiratory ciliary axoneme radial spokes
DRC1	Absent respiratory ciliary axoneme radial spokes
DYX1C1	Absent respiratory ciliary axoneme radial spokes

FAM187A	Absent respiratory ciliary axoneme radial spokes
GAS8	Absent respiratory ciliary axoneme radial spokes
HEATR2	Absent respiratory ciliary axoneme radial spokes
HYDIN	Absent respiratory ciliary axoneme radial spokes
LRRC50	Absent respiratory ciliary axoneme radial spokes
LRRC6	Absent respiratory ciliary axoneme radial spokes
NME8	Absent respiratory ciliary axoneme radial spokes
OFD1	Absent respiratory ciliary axoneme radial spokes
RPGR	Absent respiratory ciliary axoneme radial spokes
RSPH1	Absent respiratory ciliary axoneme radial spokes
RSPH3	Absent respiratory ciliary axoneme radial spokes
RSPH4A	Absent respiratory ciliary axoneme radial spokes
RSPH9	Absent respiratory ciliary axoneme radial spokes
SPAG1	Absent respiratory ciliary axoneme radial spokes
TXNDC3	Absent respiratory ciliary axoneme radial spokes
ZMYND10	Absent respiratory ciliary axoneme radial spokes
AGPAT2	Acute pancreatitis
BSCL2	Acute pancreatitis
CAV1	Acute pancreatitis
FOS	Acute pancreatitis
LMNA	Acute pancreatitis
PPARG	Acute pancreatitis
ABCB6	Agenesis of pulmonary vessels
GDF3	Agenesis of pulmonary vessels
GDF6	Agenesis of pulmonary vessels
ODZ3	Agenesis of pulmonary vessels
RARB	Agenesis of pulmonary vessels
RBP4	Agenesis of pulmonary vessels
SHH	Agenesis of pulmonary vessels
STRA6	Agenesis of pulmonary vessels
TENM3	Agenesis of pulmonary vessels

VSX2	Agenesis of pulmonary vessels
ATP11A	Alveolar cell carcinoma
BRAF	Alveolar cell carcinoma
DLEC1	Alveolar cell carcinoma
DPP9	Alveolar cell carcinoma
DSP	Alveolar cell carcinoma
EGFR	Alveolar cell carcinoma
ERBB2	Alveolar cell carcinoma
FAM13A	Alveolar cell carcinoma
IRF1	Alveolar cell carcinoma
KRAS	Alveolar cell carcinoma
MAP3K8	Alveolar cell carcinoma
MUC5B	Alveolar cell carcinoma
OBFC1	Alveolar cell carcinoma
PARK2	Alveolar cell carcinoma
PARN	Alveolar cell carcinoma
PIK3CA	Alveolar cell carcinoma
PPP2R1B	Alveolar cell carcinoma
RASSF1	Alveolar cell carcinoma
RTEL1	Alveolar cell carcinoma
SFTPA1	Alveolar cell carcinoma
SFTPA2	Alveolar cell carcinoma
SFTPC	Alveolar cell carcinoma
SLC22A18	Alveolar cell carcinoma
TERC	Alveolar cell carcinoma
TERT	Alveolar cell carcinoma
TNFRSF6B	Alveolar cell carcinoma
ABCA3	Alveolar proteinosis
CSF2RA	Alveolar proteinosis
CSF2RB	Alveolar proteinosis
SFTPB	Alveolar proteinosis

SFTPC	Alveolar proteinosis
SLC7A7	Alveolar proteinosis
FOXO1	Alveolar rhabdomyosarcoma
PAX3	Alveolar rhabdomyosarcoma
PAX7	Alveolar rhabdomyosarcoma
ASPSCR1	Alveolar soft part sarcoma
TFE3	Alveolar soft part sarcoma
FGFR2	Anomalous tracheal cartilage
SNRPB	Anomalous tracheal cartilage
ACAD11	Aplasia/Hypoplasia of the lungs
ACE	Aplasia/Hypoplasia of the lungs
AGT	Aplasia/Hypoplasia of the lungs
AGTR1	Aplasia/Hypoplasia of the lungs
C8ORF62	Aplasia/Hypoplasia of the lungs
CEP120	Aplasia/Hypoplasia of the lungs
CHRM3	Aplasia/Hypoplasia of the lungs
CHRNA1	Aplasia/Hypoplasia of the lungs
CHRND	Aplasia/Hypoplasia of the lungs
CHRNG	Aplasia/Hypoplasia of the lungs
COL2A1	Aplasia/Hypoplasia of the lungs
DHCR7	Aplasia/Hypoplasia of the lungs
DOK7	Aplasia/Hypoplasia of the lungs
DYNC2H1	Aplasia/Hypoplasia of the lungs
EVC	Aplasia/Hypoplasia of the lungs
EVC2	Aplasia/Hypoplasia of the lungs
FAM111A	Aplasia/Hypoplasia of the lungs
FANCB	Aplasia/Hypoplasia of the lungs
FGF20	Aplasia/Hypoplasia of the lungs
FGFR3	Aplasia/Hypoplasia of the lungs
FLNA	Aplasia/Hypoplasia of the lungs
FLNB	Aplasia/Hypoplasia of the lungs

FRAS1	Aplasia/Hypoplasia of the lungs
FREM2	Aplasia/Hypoplasia of the lungs
FUZ	Aplasia/Hypoplasia of the lungs
GLE1	Aplasia/Hypoplasia of the lungs
GRIP1	Aplasia/Hypoplasia of the lungs
HOXD13	Aplasia/Hypoplasia of the lungs
IFT140	Aplasia/Hypoplasia of the lungs
IFT172	Aplasia/Hypoplasia of the lungs
IFT80	Aplasia/Hypoplasia of the lungs
ITGA8	Aplasia/Hypoplasia of the lungs
KAT6B	Aplasia/Hypoplasia of the lungs
LETM1	Aplasia/Hypoplasia of the lungs
LMNA	Aplasia/Hypoplasia of the lungs
LOC341378	Aplasia/Hypoplasia of the lungs
MUSK	Aplasia/Hypoplasia of the lungs
MYST4	Aplasia/Hypoplasia of the lungs
NELFA	Aplasia/Hypoplasia of the lungs
NPHP3	Aplasia/Hypoplasia of the lungs
NSDHL	Aplasia/Hypoplasia of the lungs
PHGDH	Aplasia/Hypoplasia of the lungs
PORCN	Aplasia/Hypoplasia of the lungs
PSAT1	Aplasia/Hypoplasia of the lungs
PTH1R	Aplasia/Hypoplasia of the lungs
RAPSN	Aplasia/Hypoplasia of the lungs
RARB	Aplasia/Hypoplasia of the lungs
REN	Aplasia/Hypoplasia of the lungs
RET	Aplasia/Hypoplasia of the lungs
SLC26A2	Aplasia/Hypoplasia of the lungs
STRA6	Aplasia/Hypoplasia of the lungs
TCTN3	Aplasia/Hypoplasia of the lungs
TRIP11	Aplasia/Hypoplasia of the lungs

TRPV4	Aplasia/Hypoplasia of the lungs
TTC21B	Aplasia/Hypoplasia of the lungs
VANGL1	Aplasia/Hypoplasia of the lungs
WDR19	Aplasia/Hypoplasia of the lungs
WDR34	Aplasia/Hypoplasia of the lungs
WDR60	Aplasia/Hypoplasia of the lungs
WHSC1	Aplasia/Hypoplasia of the lungs
WHSC2	Aplasia/Hypoplasia of the lungs
WNT3	Aplasia/Hypoplasia of the lungs
WT1	Aplasia/Hypoplasia of the lungs
ZMPSTE24	Aplasia/Hypoplasia of the lungs
ABCA3	Apnea
ABCD3	Apnea
ADAMTSL2	Apnea
AGRN	Apnea
AHI1	Apnea
AIP	Apnea
ALPL	Apnea
ALS2CR4	Apnea
AMER1	Apnea
ARL13B	Apnea
ASCL1	Apnea
ATP5A1	Apnea
ATP5E	Apnea
ATP5EP2	Apnea
ATP6	Apnea
ATP8	Apnea
ATPAF1	Apnea
ATPAF2	Apnea
B9D1	Apnea
BCHE	Apnea

BDNF	Apnea
BRAT1	Apnea
BTD	Apnea
BUB1	Apnea
BUB1B	Apnea
BUB3	Apnea
C20ORF54	Apnea
C20ORF7	Apnea
C5orf42	Apnea
C7ORF27	Apnea
C8ORF38	Apnea
CC2D2A	Apnea
CDKL5	Apnea
CDKN1C	Apnea
CEP290	Apnea
CEP41	Apnea
CEP57	Apnea
CHRNA1	Apnea
CHRNB1	Apnea
CHRND	Apnea
CHRNE	Apnea
CISD2	Apnea
CLCN7	Apnea
COL3A1	Apnea
COL5A1	Apnea
COX1	Apnea
COX15	Apnea
COX2	Apnea
COX3	Apnea
CPT2	Apnea
CRYAB	Apnea

CSPP1	Apnea
CTSD	Apnea
D2HGDH	Apnea
DOK7	Apnea
DPAGT1	Apnea
DST	Apnea
ECHS1	Apnea
EDN1	Apnea
EDN3	Apnea
EPOR	Apnea
FAM123B	Apnea
FBN1	Apnea
FBP1	Apnea
FGFR2	Apnea
FGFR3	Apnea
FOXG1	Apnea
FOXRED1	Apnea
GBA	Apnea
GDNF	Apnea
GFAP	Apnea
GLRA1	Apnea
GLRB	Apnea
GLUL	Apnea
GNAI3	Apnea
GPHN	Apnea
GPR101	Apnea
GPR172A	Apnea
H19	Apnea
H19-ICR	Apnea
HSPD1	Apnea
HSPD1P1	Apnea

HSPD1P4	Apnea
HSPD1P5	Apnea
HSPD1P6	Apnea
HSPG2	Apnea
IDH2	Apnea
INPP5E	Apnea
JAK2	Apnea
KAT6B	Apnea
KCNQ1OT1	Apnea
KIAA0586	Apnea
KIF7	Apnea
LIAS	Apnea
LIFR	Apnea
LIPT1	Apnea
LOC100131801	Apnea
LOC653348	Apnea
LRP4	Apnea
MECP2	Apnea
MKS1	Apnea
MT-CO1	Apnea
MT-CO3	Apnea
MT-ND1	Apnea
MT-ND5	Apnea
MT-ND6	Apnea
MTFMT	Apnea
MUSK	Apnea
MYST4	Apnea
ND1	Apnea
ND4	Apnea
ND4L	Apnea
ND5	Apnea

ND6	Apnea
NDUFA10	Apnea
NDUFA12	Apnea
NDUFA2	Apnea
NDUFA4	Apnea
NDUFA9	Apnea
NDUFAF2	Apnea
NDUFAF5	Apnea
NDUFAF6	Apnea
NDUFS1	Apnea
NDUFS2	Apnea
NDUFS3	Apnea
NDUFS4	Apnea
NDUFS7	Apnea
NDUFS8	Apnea
NDUFV1	Apnea
NDUFV2	Apnea
NEB	Apnea
NPHP1	Apnea
NSD1	Apnea
NTNG1	Apnea
OFD1	Apnea
PCCA	Apnea
PCCB	Apnea
PCK1	Apnea
PDE6D	Apnea
PDHA1	Apnea
PET100	Apnea
PEX1	Apnea
PEX10	Apnea
PEX11B	Apnea

PEX12	Apnea
PEX13	Apnea
PEX14	Apnea
PEX16	Apnea
PEX19	Apnea
PEX2	Apnea
PEX26	Apnea
PEX3	Apnea
PEX5	Apnea
PEX6	Apnea
PHOX2B	Apnea
PLCB4	Apnea
PRNP	Apnea
RAI1	Apnea
RAPSN	Apnea
RARS2	Apnea
RET	Apnea
RNF125	Apnea
RPGRIP1L	Apnea
RPS6KA3	Apnea
RUNX2	Apnea
SCN4A	Apnea
SDHA	Apnea
SFTPB	Apnea
SH2B3	Apnea
SH3BP2	Apnea
SKI	Apnea
SLC19A3	Apnea
SLC52A2	Apnea
SLC52A3	Apnea
SLC6A5	Apnea

SNX10	Apnea
SOX9	Apnea
SURF1	Apnea
TACO1	Apnea
TCF4	Apnea
TCIRG1	Apnea
TCTN1	Apnea
TCTN2	Apnea
TCTN3	Apnea
TMEM138	Apnea
TMEM216	Apnea
TMEM231	Apnea
TMEM237	Apnea
TMEM67	Apnea
TNFSF11	Apnea
TNXA	Apnea
TNXB	Apnea
TRNF	Apnea
TRNH	Apnea
TRNL1	Apnea
TRNQ	Apnea
TRNS1	Apnea
TRNS2	Apnea
TRNW	Apnea
TSGA14	Apnea
TSPYL1	Apnea
TWIST1	Apnea
WFS1	Apnea
ZC4H2	Apnea
ZNF423	Apnea
ACADSB	Apneic episodes in infancy

SCN4A	Apneic episodes in infancy
AGRN	Apneic episodes precipitated by illness, fatigue, stress
CHAT	Apneic episodes precipitated by illness, fatigue, stress
CHRNA1	Apneic episodes precipitated by illness, fatigue, stress
CHRNB1	Apneic episodes precipitated by illness, fatigue, stress
CHRND	Apneic episodes precipitated by illness, fatigue, stress
CHRNE	Apneic episodes precipitated by illness, fatigue, stress
DOK7	Apneic episodes precipitated by illness, fatigue, stress
LRP4	Apneic episodes precipitated by illness, fatigue, stress
MUSK	Apneic episodes precipitated by illness, fatigue, stress
PDHA1	Apneic episodes precipitated by illness, fatigue, stress
RAPSN	Apneic episodes precipitated by illness, fatigue, stress
SCN4A	Apneic episodes precipitated by illness, fatigue, stress
SLC25A1	Apneic episodes precipitated by illness, fatigue, stress
SNAP25	Apneic episodes precipitated by illness, fatigue, stress
SYT2	Apneic episodes precipitated by illness, fatigue, stress
AGA	Aspartylglucosaminuria
ANG	Aspiration
ATXN2	Aspiration
C9orf72	Aspiration
CHCHD10	Aspiration
CHMP2B	Aspiration
CYTSA	Aspiration
DAO	Aspiration
DCTN1	Aspiration
ERBB4	Aspiration
FIG4	Aspiration
FUS	Aspiration
GLRA1	Aspiration
GLRB	Aspiration
GM2A	Aspiration

GPHN	Aspiration
HEXA	Aspiration
HNRNPA1	Aspiration
HNRNPA1P2	Aspiration
HNRPA1L-2	Aspiration
HNRPA1L3	Aspiration
LOC644037	Aspiration
LOC645691	Aspiration
LOC728643	Aspiration
MATR3	Aspiration
MID1	Aspiration
NEFH	Aspiration
OPTN	Aspiration
PFN1	Aspiration
PON1	Aspiration
PON2	Aspiration
PON3	Aspiration
PPARGC1A	Aspiration
PRPH	Aspiration
SLC6A5	Aspiration
SOD1	Aspiration
SPECC1L	Aspiration
SQSTM1	Aspiration
TARDBP	Aspiration
TBK1	Aspiration
TREM2	Aspiration
UBQLN2	Aspiration
UNC13A	Aspiration
VAPB	Aspiration
VCP	Aspiration
AFF4	Aspiration pneumonia

TBX21	Asthma
CPS1	Asthma
CR1	Asthma
CRHR2	Asthma
CRISPLD2	Asthma
CRP	Asthma
CSF2	Asthma
CSF3	Asthma
ADA	Asthma
ALMS1	Asthma
ARL6	Asthma
ARMC4	Asthma
ARVCF	Asthma
BBIP1	Asthma
BBS1	Asthma
BBS10	Asthma
BBS12	Asthma
BBS2	Asthma
BBS4	Asthma
BBS5	Asthma
BBS7	Asthma
BBS9	Asthma
C14ORF104	Asthma
C19ORF51	Asthma
C21ORF59	Asthma
C2ORF39	Asthma
C2ORF86	Asthma
C7ORF11	Asthma
CASP8	Asthma
CCDC103	Asthma
CCDC114	Asthma

CCDC151	Asthma
CCDC39	Asthma
CCDC40	Asthma
CCDC65	Asthma
CCL11	Asthma
CCNO	Asthma
CD19	Asthma
CD81	Asthma
CDON	Asthma
CDSN	Asthma
CEP290	Asthma
CFTR	Asthma
COMT	Asthma
COX4I2	Asthma
CR2	Asthma
DCTN4	Asthma
DISP1	Asthma
DLL1	Asthma
DNAAF1	Asthma
DNAAF2	Asthma
DNAAF3	Asthma
DNAAF5	Asthma
DNAH11	Asthma
DNAH5	Asthma
DNAI1	Asthma
DNAI2	Asthma
DNAL1	Asthma
DOCK8	Asthma
DRC1	Asthma
DYX1C1	Asthma
ELOVL4	Asthma

ERCC2	Asthma
ERCC3	Asthma
FAM187A	Asthma
FGF8	Asthma
FLG	Asthma
FOXH1	Asthma
GAS1	Asthma
GAS8	Asthma
GLI2	Asthma
GP1BB	Asthma
GRHL2	Asthma
GTF2H5	Asthma
HEATR2	Asthma
HIRA	Asthma
HYDIN	Asthma
ICOS	Asthma
IDS	Asthma
IFT172	Asthma
IFT27	Asthma
KIT	Asthma
LIFR	Asthma
LIG4	Asthma
LOC652799	Asthma
LOC653882	Asthma
LRBA	Asthma
LRRC50	Asthma
LRRC6	Asthma
LZTFL1	Asthma
MKKS	Asthma
MKS1	Asthma
MPLKIP	Asthma

MS4A1	Asthma
NCRNA00081	Asthma
NFKB1	Asthma
NFKB2	Asthma
NKX2-1	Asthma
NME8	Asthma
NODAL	Asthma
NPHP1	Asthma
NSUN2	Asthma
OFD1	Asthma
PEPD	Asthma
PGM3	Asthma
PLCG2	Asthma
PRKCD	Asthma
PTCH1	Asthma
RABL4	Asthma
RNF113A	Asthma
RNU4ATAC	Asthma
RPGR	Asthma
RSPH1	Asthma
RSPH3	Asthma
RSPH4A	Asthma
RSPH9	Asthma
SCN4A	Asthma
SDCCAG8	Asthma
SHH	Asthma
SIX3	Asthma
SPAG1	Asthma
SPINK5	Asthma
STX1A	Asthma
TALDO1	Asthma

TBX1	Asthma
TBX21	Asthma
TDGF1	Asthma
TDGF3	Asthma
TGFB1	Asthma
TGIF1	Asthma
TNFRSF13B	Asthma
TNFRSF13C	Asthma
TNFSF12	Asthma
TNFSF12-TNFSF1	Asthma
TNFSF13	Asthma
TRIM32	Asthma
TSPYL1	Asthma
TTC8	Asthma
TXNDC3	Asthma
UFD1L	Asthma
WDPCP	Asthma
ZIC2	Asthma
ZMYND10	Asthma
CCL2	Asthma
CCL20	Asthma
CCL24	Asthma
CCL3	Asthma
CCL4	Asthma
CCL5	Asthma
CCL7	Asthma
CCL8	Asthma
CCND1	Asthma
CCNG1	Asthma
CCNL2	Asthma
CCR1	Asthma

CCR2	Asthma
CCR3	Asthma
CCR6	Asthma
CCR9	Asthma
CD14	Asthma
CD207	Asthma
CD209	Asthma
CD28	Asthma
CD36	Asthma
CD38	Asthma
CD40LG	Asthma
CD46	Asthma
CD8A	Asthma
CD97	Asthma
CDAN1	Asthma
CDAN3	Asthma
CDH1	Asthma
CDH13	Asthma
CDHR3	Asthma
CDKN1A	Asthma
CDKN1B	Asthma
CDKN1C	Asthma
CDKN2A	Asthma
CEACAM1	Asthma
CEBPB	Asthma
CES1	Asthma
CFLAR	Asthma
CHEK2	Asthma
CHI3L1	Asthma
CHPT1	Asthma
CHRFAM7A	Asthma

CHRNA3	Asthma
CHRNA5	Asthma
CHRNA7	Asthma
CHRNB4	Asthma
CHRNG	Asthma
CILD4	Asthma
CILD8	Asthma
CISH	Asthma
CLCN5	Asthma
CLEC4D	Asthma
CLEC4E	Asthma
CLPTM1L	Asthma
CMA1	Asthma
COL11A2	Asthma
COL1A1	Asthma
COL3A1	Asthma
COL6A1	Asthma
COPD	Asthma
CORO1A	Asthma
CP	Asthma
CSF3	Asthma
CTEPH1	Asthma
CTGF	Asthma
CTLA4	Asthma
CTNNA3	Asthma
ARMC4	Atelectasis
ARVCF	Atelectasis
C14ORF104	Atelectasis
C19ORF51	Atelectasis
C21ORF59	Atelectasis
C2ORF39	Atelectasis

CCDC103	Atelectasis
CCDC114	Atelectasis
CCDC151	Atelectasis
CCDC39	Atelectasis
CCDC40	Atelectasis
CCDC65	Atelectasis
CCNO	Atelectasis
COMT	Atelectasis
DNAAF1	Atelectasis
DNAAF2	Atelectasis
DNAAF3	Atelectasis
DNAAF5	Atelectasis
DNAH11	Atelectasis
DNAH5	Atelectasis
DNAI1	Atelectasis
DNAI2	Atelectasis
DNAL1	Atelectasis
DRC1	Atelectasis
DYX1C1	Atelectasis
EFEMP2	Atelectasis
FAM187A	Atelectasis
FBLN5	Atelectasis
GAS8	Atelectasis
GP1BB	Atelectasis
HEATR2	Atelectasis
HIRA	Atelectasis
HYDIN	Atelectasis
LRRC50	Atelectasis
LRRC6	Atelectasis
NME8	Atelectasis
OCRL	Atelectasis

OFD1	Atelectasis
PAX3	Atelectasis
RPGR	Atelectasis
RSPH1	Atelectasis
RSPH3	Atelectasis
RSPH4A	Atelectasis
RSPH9	Atelectasis
SFTPB	Atelectasis
SFTPC	Atelectasis
SPAG1	Atelectasis
STAT3	Atelectasis
TBX1	Atelectasis
TSC1	Atelectasis
TSC2	Atelectasis
TXNDC3	Atelectasis
UFD1L	Atelectasis
ZMYND10	Atelectasis
AHI1	Bell-shaped thorax
ALS2CR4	Bell-shaped thorax
ARL13B	Bell-shaped thorax
B9D1	Bell-shaped thorax
BMPER	Bell-shaped thorax
C5orf42	Bell-shaped thorax
CEP120	Bell-shaped thorax
CEP41	Bell-shaped thorax
COL11A1	Bell-shaped thorax
COL11A2	Bell-shaped thorax
CSPP1	Bell-shaped thorax
DDR2	Bell-shaped thorax
DYNC2H1	Bell-shaped thorax
FGFR2	Bell-shaped thorax

HHAT	Bell-shaped thorax
IFT140	Bell-shaped thorax
IFT172	Bell-shaped thorax
IFT80	Bell-shaped thorax
INPP5E	Bell-shaped thorax
INPPL1	Bell-shaped thorax
KIAA0586	Bell-shaped thorax
MKS1	Bell-shaped thorax
PAM16	Bell-shaped thorax
PEX1	Bell-shaped thorax
PEX10	Bell-shaped thorax
PEX11B	Bell-shaped thorax
PEX12	Bell-shaped thorax
PEX13	Bell-shaped thorax
PEX14	Bell-shaped thorax
PEX16	Bell-shaped thorax
PEX19	Bell-shaped thorax
PEX2	Bell-shaped thorax
PEX26	Bell-shaped thorax
PEX3	Bell-shaped thorax
PEX5	Bell-shaped thorax
PEX6	Bell-shaped thorax
SNRPB	Bell-shaped thorax
TBX15	Bell-shaped thorax
TCTN1	Bell-shaped thorax
TCTN2	Bell-shaped thorax
TIMM16	Bell-shaped thorax
TMEM237	Bell-shaped thorax
TMEM67	Bell-shaped thorax
TSGA14	Bell-shaped thorax
TTC21B	Bell-shaped thorax

UBE3B	Bell-shaped thorax
WDR19	Bell-shaped thorax
WDR34	Bell-shaped thorax
WDR35	Bell-shaped thorax
WDR60	Bell-shaped thorax
ABCB6	Bilateral lung agenesis
GDF3	Bilateral lung agenesis
GDF6	Bilateral lung agenesis
ODZ3	Bilateral lung agenesis
RARB	Bilateral lung agenesis
RBP4	Bilateral lung agenesis
SHH	Bilateral lung agenesis
STRA6	Bilateral lung agenesis
TENM3	Bilateral lung agenesis
VSX2	Bilateral lung agenesis
WNT4	Bilateral lung agenesis
AHI1	Breathing dysregulation
ALS2CR4	Breathing dysregulation
ARL13B	Breathing dysregulation
B9D1	Breathing dysregulation
C5orf42	Breathing dysregulation
CEP41	Breathing dysregulation
CSPP1	Breathing dysregulation
INPP5E	Breathing dysregulation
KIAA0586	Breathing dysregulation
MKS1	Breathing dysregulation
TCTN1	Breathing dysregulation
TCTN2	Breathing dysregulation
TMEM237	Breathing dysregulation
TMEM67	Breathing dysregulation
TSGA14	Breathing dysregulation

ARMC4	Bronchiectasis
ATM	Bronchiectasis
B2M	Bronchiectasis
BLM	Bronchiectasis
BLNK	Bronchiectasis
C14ORF104	Bronchiectasis
C19ORF51	Bronchiectasis
C21ORF59	Bronchiectasis
C2ORF39	Bronchiectasis
CCDC103	Bronchiectasis
CCDC114	Bronchiectasis
CCDC151	Bronchiectasis
CCDC39	Bronchiectasis
CCDC40	Bronchiectasis
CCDC65	Bronchiectasis
CCNO	Bronchiectasis
CD19	Bronchiectasis
CD79A	Bronchiectasis
CD79B	Bronchiectasis
CD81	Bronchiectasis
CD8A	Bronchiectasis
CDCA7	Bronchiectasis
CFTR	Bronchiectasis
CR2	Bronchiectasis
DCTN4	Bronchiectasis
DNAAF1	Bronchiectasis
DNAAF2	Bronchiectasis
DNAAF3	Bronchiectasis
DNAAF5	Bronchiectasis
DNAH11	Bronchiectasis
DNAH5	Bronchiectasis

DNAI1	Bronchiectasis
DNAI2	Bronchiectasis
DNAL1	Bronchiectasis
DNMT3B	Bronchiectasis
DPCR1	Bronchiectasis
DRC1	Bronchiectasis
DYX1C1	Bronchiectasis
FAM187A	Bronchiectasis
GAS8	Bronchiectasis
HEATR2	Bronchiectasis
HELLS	Bronchiectasis
HYDIN	Bronchiectasis
ICOS	Bronchiectasis
IGH@	Bronchiectasis
IGHG1	Bronchiectasis
IGHG3	Bronchiectasis
IGHM	Bronchiectasis
IGHV3-11	Bronchiectasis
IGHV3-7	Bronchiectasis
IGHV4-31	Bronchiectasis
IGLL1	Bronchiectasis
IL21R	Bronchiectasis
LOC651610	Bronchiectasis
LRBA	Bronchiectasis
LRRC50	Bronchiectasis
LRRC6	Bronchiectasis
LRRC8A	Bronchiectasis
MS4A1	Bronchiectasis
MUC5B	Bronchiectasis
NBN	Bronchiectasis
NFKB1	Bronchiectasis

NFKB2	Bronchiectasis
NME8	Bronchiectasis
OFD1	Bronchiectasis
PGM3	Bronchiectasis
PIK3CD	Bronchiectasis
PIK3R1	Bronchiectasis
PRKCD	Bronchiectasis
RIN2	Bronchiectasis
RPGR	Bronchiectasis
RSPH1	Bronchiectasis
RSPH3	Bronchiectasis
RSPH4A	Bronchiectasis
RSPH9	Bronchiectasis
SCNN1A	Bronchiectasis
SCNN1B	Bronchiectasis
SCNN1G	Bronchiectasis
SPAG1	Bronchiectasis
STX1A	Bronchiectasis
TAP1	Bronchiectasis
TAP2	Bronchiectasis
TAPBP	Bronchiectasis
TCF3	Bronchiectasis
TGFB1	Bronchiectasis
TNFRSF13B	Bronchiectasis
TNFRSF13C	Bronchiectasis
TNFSF12	Bronchiectasis
TNFSF12-TNFSF1	Bronchiectasis
TNFSF13	Bronchiectasis
TXNDC3	Bronchiectasis
ZBTB24	Bronchiectasis
ZMYND10	Bronchiectasis

CYB5R3	Bronchiectasis
CYP1A1	Bronchiectasis
CYP1A2	Bronchiectasis
ARL6	Bronchiolitis
B2M	Bronchiolitis
BBIP1	Bronchiolitis
BBS1	Bronchiolitis
BBS10	Bronchiolitis
BBS12	Bronchiolitis
BBS2	Bronchiolitis
BBS4	Bronchiolitis
BBS5	Bronchiolitis
BBS7	Bronchiolitis
BBS9	Bronchiolitis
C2ORF86	Bronchiolitis
CEP290	Bronchiolitis
IFT172	Bronchiolitis
IFT27	Bronchiolitis
LZTFL1	Bronchiolitis
MKKS	Bronchiolitis
MKS1	Bronchiolitis
NCRNA00081	Bronchiolitis
NPHP1	Bronchiolitis
PLCG2	Bronchiolitis
RABL4	Bronchiolitis
SDCCAG8	Bronchiolitis
TAP1	Bronchiolitis
TAP2	Bronchiolitis
TAPBP	Bronchiolitis
TRIM32	Bronchiolitis
TTC8	Bronchiolitis

WDPCP	Bronchiolitis
UNC119	Bronchiolitis obliterans organizing pneumonia
AKT1	Bronchogenic cyst
BRAF	Bronchogenic cyst
KILLIN	Bronchogenic cyst
KLLN	Bronchogenic cyst
LOC100130320	Bronchogenic cyst
PIK3CA	Bronchogenic cyst
PTCH1	Bronchogenic cyst
PTCH2	Bronchogenic cyst
PTEN	Bronchogenic cyst
PTENP1	Bronchogenic cyst
SDHB	Bronchogenic cyst
SDHC	Bronchogenic cyst
SDHD	Bronchogenic cyst
SUFU	Bronchogenic cyst
CDC6	Bronchomalacia
CDT1	Bronchomalacia
FGFR1	Bronchomalacia
FGFR2	Bronchomalacia
FLNB	Bronchomalacia
HRAS	Bronchomalacia
LTBP4	Bronchomalacia
ORC1	Bronchomalacia
ORC1L	Bronchomalacia
ORC4	Bronchomalacia
ORC4L	Bronchomalacia
ORC6	Bronchomalacia
ORC6L	Bronchomalacia
POR	Bronchomalacia
CDKN1A	Carcinoid tumor

CDKN1B	Carcinoid tumor
CDKN2B	Carcinoid tumor
CDKN2C	Carcinoid tumor
MEN1	Carcinoid tumor
SLC25A20	Cardiorespiratory arrest
TSPYL1	Cardiorespiratory arrest
DMXL2	Central hypothyroidism
NIN	Central hypothyroidism
ASCL1	Central hypoventilation
BDNF	Central hypoventilation
DCTN1	Central hypoventilation
EDN3	Central hypoventilation
GDNF	Central hypoventilation
MECP2	Central hypoventilation
PHOX2B	Central hypoventilation
RET	Central hypoventilation
ARMC4	Chronic bronchitis
C14ORF104	Chronic bronchitis
C19ORF51	Chronic bronchitis
C21ORF59	Chronic bronchitis
C2ORF39	Chronic bronchitis
CCDC103	Chronic bronchitis
CCDC114	Chronic bronchitis
CCDC151	Chronic bronchitis
CCDC39	Chronic bronchitis
CCDC40	Chronic bronchitis
CCDC65	Chronic bronchitis
CCNO	Chronic bronchitis
CDCA7	Chronic bronchitis
CFTR	Chronic bronchitis
DNAAF1	Chronic bronchitis

DNAAF2	Chronic bronchitis
DNAAF3	Chronic bronchitis
DNAAF5	Chronic bronchitis
DNAH11	Chronic bronchitis
DNAH5	Chronic bronchitis
DNAI1	Chronic bronchitis
DNAI2	Chronic bronchitis
DNAL1	Chronic bronchitis
DNMT3B	Chronic bronchitis
DRC1	Chronic bronchitis
DYX1C1	Chronic bronchitis
FAM187A	Chronic bronchitis
GAS8	Chronic bronchitis
HEATR2	Chronic bronchitis
HELLS	Chronic bronchitis
HYDIN	Chronic bronchitis
LRRC50	Chronic bronchitis
LRRC6	Chronic bronchitis
NME8	Chronic bronchitis
OFD1	Chronic bronchitis
RPGR	Chronic bronchitis
RSPH1	Chronic bronchitis
RSPH3	Chronic bronchitis
RSPH4A	Chronic bronchitis
RSPH9	Chronic bronchitis
SCNN1A	Chronic bronchitis
SCNN1B	Chronic bronchitis
SCNN1G	Chronic bronchitis
SPAG1	Chronic bronchitis
TXNDC3	Chronic bronchitis
ZBTB24	Chronic bronchitis

ZMYND10	Chronic bronchitis
CYP1A2	Chronic bronchitis
CYP1B1	Chronic bronchitis
AFF4	Chronic lung disease
BLM	Chronic lung disease
BMP1	Chronic lung disease
CD19	Chronic lung disease
CD81	Chronic lung disease
CFTR	Chronic lung disease
COL1A1	Chronic lung disease
COL1A2	Chronic lung disease
CR2	Chronic lung disease
CREB3L1	Chronic lung disease
CRTAP	Chronic lung disease
DCTN4	Chronic lung disease
FKBP10	Chronic lung disease
ICOS	Chronic lung disease
LEPRE1	Chronic lung disease
LRBA	Chronic lung disease
MS4A1	Chronic lung disease
NFKB1	Chronic lung disease
NFKB2	Chronic lung disease
P3H1	Chronic lung disease
PEPD	Chronic lung disease
PPIB	Chronic lung disease
PRKCD	Chronic lung disease
SERPINF1	Chronic lung disease
SERPINH1	Chronic lung disease
STX1A	Chronic lung disease
TGFB1	Chronic lung disease
TNFRSF13B	Chronic lung disease

TNFRSF13C	Chronic lung disease
TNFSF12	Chronic lung disease
TNFSF12-TNFSF1	Chronic lung disease
TNFSF13	Chronic lung disease
WNT1	Chronic lung disease
ARMC4	Chronic obstructive pulmonary disease
ARVCF	Chronic obstructive pulmonary disease
C14ORF104	Chronic obstructive pulmonary disease
C19ORF51	Chronic obstructive pulmonary disease
C21ORF59	Chronic obstructive pulmonary disease
C2ORF39	Chronic obstructive pulmonary disease
CCDC103	Chronic obstructive pulmonary disease
CCDC114	Chronic obstructive pulmonary disease
CCDC151	Chronic obstructive pulmonary disease
CCDC39	Chronic obstructive pulmonary disease
CCDC40	Chronic obstructive pulmonary disease
CCDC65	Chronic obstructive pulmonary disease
CCNO	Chronic obstructive pulmonary disease
CD19	Chronic obstructive pulmonary disease
CD81	Chronic obstructive pulmonary disease
COMT	Chronic obstructive pulmonary disease
CR2	Chronic obstructive pulmonary disease
CTLA4	Chronic obstructive pulmonary disease
CYBA	Chronic obstructive pulmonary disease
CYBB	Chronic obstructive pulmonary disease
DNAAF1	Chronic obstructive pulmonary disease
DNAAF2	Chronic obstructive pulmonary disease
DNAAF3	Chronic obstructive pulmonary disease
DNAAF5	Chronic obstructive pulmonary disease
DNAH11	Chronic obstructive pulmonary disease
DNAH5	Chronic obstructive pulmonary disease

DNAI1	Chronic obstructive pulmonary disease
DNAI2	Chronic obstructive pulmonary disease
DNAL1	Chronic obstructive pulmonary disease
DNASE1L3	Chronic obstructive pulmonary disease
DRC1	Chronic obstructive pulmonary disease
DYX1C1	Chronic obstructive pulmonary disease
FAM187A	Chronic obstructive pulmonary disease
GAS8	Chronic obstructive pulmonary disease
GLA	Chronic obstructive pulmonary disease
GP1BB	Chronic obstructive pulmonary disease
HEATR2	Chronic obstructive pulmonary disease
HIRA	Chronic obstructive pulmonary disease
HLA-DPB1	Chronic obstructive pulmonary disease
HYDIN	Chronic obstructive pulmonary disease
ICOS	Chronic obstructive pulmonary disease
LRBA	Chronic obstructive pulmonary disease
LRRC50	Chronic obstructive pulmonary disease
LRRC6	Chronic obstructive pulmonary disease
MMP1	Chronic obstructive pulmonary disease
MS4A1	Chronic obstructive pulmonary disease
NCF1	Chronic obstructive pulmonary disease
NCF1C	Chronic obstructive pulmonary disease
NCF2	Chronic obstructive pulmonary disease
NCF4	Chronic obstructive pulmonary disease
NFKB1	Chronic obstructive pulmonary disease
NFKB2	Chronic obstructive pulmonary disease
NME8	Chronic obstructive pulmonary disease
OFD1	Chronic obstructive pulmonary disease
PRKCD	Chronic obstructive pulmonary disease
PRTN3	Chronic obstructive pulmonary disease
PTPN22	Chronic obstructive pulmonary disease

RPGR	Chronic obstructive pulmonary disease
RSPH1	Chronic obstructive pulmonary disease
RSPH3	Chronic obstructive pulmonary disease
RSPH4A	Chronic obstructive pulmonary disease
RSPH9	Chronic obstructive pulmonary disease
SERPINA1	Chronic obstructive pulmonary disease
SPAG1	Chronic obstructive pulmonary disease
TBX1	Chronic obstructive pulmonary disease
TNFRSF13B	Chronic obstructive pulmonary disease
TNFRSF13C	Chronic obstructive pulmonary disease
TNFSF12	Chronic obstructive pulmonary disease
TNFSF12-TNFSF1	Chronic obstructive pulmonary disease
TNFSF13	Chronic obstructive pulmonary disease
TXNDC3	Chronic obstructive pulmonary disease
UFD1L	Chronic obstructive pulmonary disease
WAS	Chronic obstructive pulmonary disease
WIPF1	Chronic obstructive pulmonary disease
ZMYND10	Chronic obstructive pulmonary disease
RUNX3	Chronic obstructive pulmonary disease
SCGB1A1	Chronic obstructive pulmonary disease
SCGB3A2	Chronic obstructive pulmonary disease
SCN4A	Chronic obstructive pulmonary disease
SCNN1A	Chronic obstructive pulmonary disease
SCNN1B	Chronic obstructive pulmonary disease
SCNN1G	Chronic obstructive pulmonary disease
SEC23B	Chronic obstructive pulmonary disease
SELENBP1	Chronic obstructive pulmonary disease
SELP	Chronic obstructive pulmonary disease
SEPP1	Chronic obstructive pulmonary disease
SERPINA10	Chronic obstructive pulmonary disease
ABCB6	Congenital diaphragmatic hernia

ARID1A	Congenital diaphragmatic hernia
ARID1B	Congenital diaphragmatic hernia
B3GAT3	Congenital diaphragmatic hernia
B3GAT3P1	Congenital diaphragmatic hernia
C6ORF59	Congenital diaphragmatic hernia
CD96	Congenital diaphragmatic hernia
CDKN1C	Congenital diaphragmatic hernia
CHRNA1	Congenital diaphragmatic hernia
CHRND	Congenital diaphragmatic hernia
CHRNG	Congenital diaphragmatic hernia
COL1A1	Congenital diaphragmatic hernia
COL5A1	Congenital diaphragmatic hernia
COL5A2	Congenital diaphragmatic hernia
COX7B	Congenital diaphragmatic hernia
DACT1	Congenital diaphragmatic hernia
DHCR7	Congenital diaphragmatic hernia
DIS3L2	Congenital diaphragmatic hernia
DLL3	Congenital diaphragmatic hernia
EFEMP2	Congenital diaphragmatic hernia
EFNB1	Congenital diaphragmatic hernia
FBLN5	Congenital diaphragmatic hernia
GATA1	Congenital diaphragmatic hernia
GATA4	Congenital diaphragmatic hernia
GATA6	Congenital diaphragmatic hernia
GDF3	Congenital diaphragmatic hernia
GDF6	Congenital diaphragmatic hernia
GLI3	Congenital diaphragmatic hernia
GPC3	Congenital diaphragmatic hernia
GPC4	Congenital diaphragmatic hernia
H19	Congenital diaphragmatic hernia
H19-ICR	Congenital diaphragmatic hernia

HCCS	Congenital diaphragmatic hernia
HDAC4	Congenital diaphragmatic hernia
HDAC8	Congenital diaphragmatic hernia
HES7	Congenital diaphragmatic hernia
HOXD13	Congenital diaphragmatic hernia
KCNA1	Congenital diaphragmatic hernia
KCNQ1OT1	Congenital diaphragmatic hernia
KDM6A	Congenital diaphragmatic hernia
KIF7	Congenital diaphragmatic hernia
KMT2D	Congenital diaphragmatic hernia
LETM1	Congenital diaphragmatic hernia
LFNG	Congenital diaphragmatic hernia
LRP2	Congenital diaphragmatic hernia
MESP2	Congenital diaphragmatic hernia
MLL2	Congenital diaphragmatic hernia
NDUFB11	Congenital diaphragmatic hernia
NELFA	Congenital diaphragmatic hernia
NIPBL	Congenital diaphragmatic hernia
NSD1	Congenital diaphragmatic hernia
ODZ3	Congenital diaphragmatic hernia
PORCN	Congenital diaphragmatic hernia
RAD21	Congenital diaphragmatic hernia
RAPSN	Congenital diaphragmatic hernia
RARB	Congenital diaphragmatic hernia
RBP4	Congenital diaphragmatic hernia
RIPPLY2	Congenital diaphragmatic hernia
RPL11	Congenital diaphragmatic hernia
RPL15	Congenital diaphragmatic hernia
RPL15P17	Congenital diaphragmatic hernia
RPL15P18	Congenital diaphragmatic hernia
RPL15P22	Congenital diaphragmatic hernia

RPL15P3	Congenital diaphragmatic hernia
RPL15P7	Congenital diaphragmatic hernia
RPL26	Congenital diaphragmatic hernia
RPL26P16	Congenital diaphragmatic hernia
RPL26P19	Congenital diaphragmatic hernia
RPL26P33	Congenital diaphragmatic hernia
RPL26P6	Congenital diaphragmatic hernia
RPL35A	Congenital diaphragmatic hernia
RPL5	Congenital diaphragmatic hernia
RPL5P1	Congenital diaphragmatic hernia
RPL5P34	Congenital diaphragmatic hernia
RPS10	Congenital diaphragmatic hernia
RPS10P11	Congenital diaphragmatic hernia
RPS10P13	Congenital diaphragmatic hernia
RPS10P22	Congenital diaphragmatic hernia
RPS10P4	Congenital diaphragmatic hernia
RPS10P7	Congenital diaphragmatic hernia
RPS17	Congenital diaphragmatic hernia
RPS17L	Congenital diaphragmatic hernia
RPS19	Congenital diaphragmatic hernia
RPS19P3	Congenital diaphragmatic hernia
RPS24	Congenital diaphragmatic hernia
RPS26	Congenital diaphragmatic hernia
RPS26P2	Congenital diaphragmatic hernia
RPS26P20	Congenital diaphragmatic hernia
RPS26P25	Congenital diaphragmatic hernia
RPS26P31	Congenital diaphragmatic hernia
RPS26P35	Congenital diaphragmatic hernia
RPS26P38	Congenital diaphragmatic hernia
RPS26P39	Congenital diaphragmatic hernia
RPS26P50	Congenital diaphragmatic hernia

RPS26P53	Congenital diaphragmatic hernia
RPS26P54	Congenital diaphragmatic hernia
RPS26P6	Congenital diaphragmatic hernia
RPS26P8	Congenital diaphragmatic hernia
RPS28	Congenital diaphragmatic hernia
RPS28P6	Congenital diaphragmatic hernia
RPS28P9	Congenital diaphragmatic hernia
RPS29	Congenital diaphragmatic hernia
RPS29P11	Congenital diaphragmatic hernia
RPS29P16	Congenital diaphragmatic hernia
RPS29P17	Congenital diaphragmatic hernia
RPS29P3	Congenital diaphragmatic hernia
RPS29P9	Congenital diaphragmatic hernia
RPS7	Congenital diaphragmatic hernia
RPS7P10	Congenital diaphragmatic hernia
RPS7P11	Congenital diaphragmatic hernia
RPS7P4	Congenital diaphragmatic hernia
SH2B1	Congenital diaphragmatic hernia
SHH	Congenital diaphragmatic hernia
SLC2A10	Congenital diaphragmatic hernia
SMARCA4	Congenital diaphragmatic hernia
SMARCB1	Congenital diaphragmatic hernia
SMARCE1	Congenital diaphragmatic hernia
SMC1A	Congenital diaphragmatic hernia
SMC3	Congenital diaphragmatic hernia
SOX11	Congenital diaphragmatic hernia
STRA6	Congenital diaphragmatic hernia
TENM3	Congenital diaphragmatic hernia
TPI1	Congenital diaphragmatic hernia
TPI1P1	Congenital diaphragmatic hernia
TSR2	Congenital diaphragmatic hernia

VSX2	Congenital diaphragmatic hernia
WHSC1	Congenital diaphragmatic hernia
WHSC2	Congenital diaphragmatic hernia
WT1	Congenital diaphragmatic hernia
ZFPM2	Congenital diaphragmatic hernia
DUOX2	Congenital hypothyroidism
DUOXA2	Congenital hypothyroidism
GABRD	Congenital hypothyroidism
GLIS3	Congenital hypothyroidism
IYD	Congenital hypothyroidism
KCNAB2	Congenital hypothyroidism
KDM6A	Congenital hypothyroidism
KMT2D	Congenital hypothyroidism
MLL2	Congenital hypothyroidism
NKX2-1	Congenital hypothyroidism
NKX2-5	Congenital hypothyroidism
PAX8	Congenital hypothyroidism
PDE4D	Congenital hypothyroidism
PRDM16	Congenital hypothyroidism
PRKAR1A	Congenital hypothyroidism
SKI	Congenital hypothyroidism
SLC26A4	Congenital hypothyroidism
SLC5A5	Congenital hypothyroidism
TG	Congenital hypothyroidism
THRA	Congenital hypothyroidism
THRB	Congenital hypothyroidism
TPO	Congenital hypothyroidism
TSHB	Congenital hypothyroidism
TSHR	Congenital hypothyroidism
ABCC6	Congestive heart failure
ABCC9	Congestive heart failure

ACAD9	Congestive heart failure
ACTC1	Congestive heart failure
ACTN2	Congestive heart failure
ACVRL1	Congestive heart failure
ADAMTSL2	Congestive heart failure
ADCY5	Congestive heart failure
AGGF1	Congestive heart failure
AKAP9	Congestive heart failure
AKT2	Congestive heart failure
ALMS1	Congestive heart failure
ANK2	Congestive heart failure
APOA1	Congestive heart failure
ATP5A1	Congestive heart failure
ATP6	Congestive heart failure
ATP8	Congestive heart failure
BAG3	Congestive heart failure
BAZ1B	Congestive heart failure
C3ORF34	Congestive heart failure
CALM1	Congestive heart failure
CALM2	Congestive heart failure
CALM3	Congestive heart failure
CASR	Congestive heart failure
CAV1	Congestive heart failure
CAV3	Congestive heart failure
CCNL2	Congestive heart failure
CCR6	Congestive heart failure
CEP19	Congestive heart failure
CISD2	Congestive heart failure
CLIC2	Congestive heart failure
CLIP2	Congestive heart failure
COL1A1	Congestive heart failure

COL1A2	Congestive heart failure
COX1	Congestive heart failure
COX2	Congestive heart failure
COX3	Congestive heart failure
CP	Congestive heart failure
CRTAP	Congestive heart failure
CRYAB	Congestive heart failure
CSRP3	Congestive heart failure
CTGF	Congestive heart failure
CYTB	Congestive heart failure
DDX58	Congestive heart failure
DES	Congestive heart failure
DMD	Congestive heart failure
DNAJC19	Congestive heart failure
DOLK	Congestive heart failure
DSG2	Congestive heart failure
DSP	Congestive heart failure
DTNA	Congestive heart failure
EFEMP2	Congestive heart failure
ELAC2	Congestive heart failure
ELN	Congestive heart failure
ENG	Congestive heart failure
ENPP1	Congestive heart failure
EPG5	Congestive heart failure
EYA4	Congestive heart failure
FBLN5	Congestive heart failure
FBN1	Congestive heart failure
FGD1	Congestive heart failure
FGF23	Congestive heart failure
FGFR3	Congestive heart failure
FH	Congestive heart failure

FHL2	Congestive heart failure
FKTN	Congestive heart failure
FLNA	Congestive heart failure
FPGT	Congestive heart failure
FXN	Congestive heart failure
GATA1	Congestive heart failure
GATAD1	Congestive heart failure
GBA	Congestive heart failure
GDF2	Congestive heart failure
GJA1	Congestive heart failure
GLA	Congestive heart failure
GLB1	Congestive heart failure
GNA11	Congestive heart failure
GNPTAB	Congestive heart failure
GTF2I	Congestive heart failure
GTF2IRD1	Congestive heart failure
GTPBP3	Congestive heart failure
HADHA	Congestive heart failure
HADHB	Congestive heart failure
HBA1	Congestive heart failure
HBA2	Congestive heart failure
HEXB	Congestive heart failure
HFE	Congestive heart failure
HLA-DRB1	Congestive heart failure
HLA-DRB4	Congestive heart failure
IDS	Congestive heart failure
IFIH1	Congestive heart failure
IGHV4-34	Congestive heart failure
IKBKG	Congestive heart failure
IRF5	Congestive heart failure
JUP	Congestive heart failure

KCNE1	Congestive heart failure
KCNE2	Congestive heart failure
KCNH2	Congestive heart failure
KCNJ5	Congestive heart failure
KCNQ1	Congestive heart failure
KIAA1632	Congestive heart failure
KIF1B	Congestive heart failure
LAMA4	Congestive heart failure
LDB3	Congestive heart failure
LEPRE1	Congestive heart failure
LIMK1	Congestive heart failure
LMNA	Congestive heart failure
LOC100093631	Congestive heart failure
LOC100130320	Congestive heart failure
LOC644589	Congestive heart failure
LOC652522	Congestive heart failure
LOC653348	Congestive heart failure
LTBP4	Congestive heart failure
MAX	Congestive heart failure
MIB1	Congestive heart failure
MT-CO1	Congestive heart failure
MT-CO3	Congestive heart failure
MT-CYB	Congestive heart failure
MT-ND1	Congestive heart failure
MT-ND5	Congestive heart failure
MT-ND6	Congestive heart failure
MYBPC3	Congestive heart failure
MYD88	Congestive heart failure
MYH6	Congestive heart failure
MYH7	Congestive heart failure
MYH7B	Congestive heart failure

MYLK2	Congestive heart failure
MYPN	Congestive heart failure
ND1	Congestive heart failure
ND4	Congestive heart failure
ND4L	Congestive heart failure
ND5	Congestive heart failure
ND6	Congestive heart failure
NEXN	Congestive heart failure
NOS1AP	Congestive heart failure
P3H1	Congestive heart failure
PEX7	Congestive heart failure
PHYH	Congestive heart failure
PLN	Congestive heart failure
PLOD1	Congestive heart failure
PPIB	Congestive heart failure
PRDM16	Congestive heart failure
PRKAG2	Congestive heart failure
PRKAR1A	Congestive heart failure
PSEN1	Congestive heart failure
PSEN2	Congestive heart failure
PSMB8	Congestive heart failure
PTEN	Congestive heart failure
PTENP1	Congestive heart failure
RAB3GAP1	Congestive heart failure
RAB3GAP2	Congestive heart failure
RAF1	Congestive heart failure
RASA1	Congestive heart failure
RBM20	Congestive heart failure
RET	Congestive heart failure
RFC2	Congestive heart failure
RPL11	Congestive heart failure

RPL15	Congestive heart failure
RPL15P17	Congestive heart failure
RPL15P18	Congestive heart failure
RPL15P22	Congestive heart failure
RPL15P3	Congestive heart failure
RPL15P7	Congestive heart failure
RPL26	Congestive heart failure
RPL26P16	Congestive heart failure
RPL26P19	Congestive heart failure
RPL26P33	Congestive heart failure
RPL26P6	Congestive heart failure
RPL35A	Congestive heart failure
RPL5	Congestive heart failure
RPL5P1	Congestive heart failure
RPL5P34	Congestive heart failure
RPS10	Congestive heart failure
RPS10P11	Congestive heart failure
RPS10P13	Congestive heart failure
RPS10P22	Congestive heart failure
RPS10P4	Congestive heart failure
RPS10P7	Congestive heart failure
RPS17	Congestive heart failure
RPS17L	Congestive heart failure
RPS19	Congestive heart failure
RPS19P3	Congestive heart failure
RPS24	Congestive heart failure
RPS26	Congestive heart failure
RPS26P2	Congestive heart failure
RPS26P20	Congestive heart failure
RPS26P25	Congestive heart failure
RPS26P31	Congestive heart failure

RPS26P35	Congestive heart failure
RPS26P38	Congestive heart failure
RPS26P39	Congestive heart failure
RPS26P50	Congestive heart failure
RPS26P53	Congestive heart failure
RPS26P54	Congestive heart failure
RPS26P6	Congestive heart failure
RPS26P8	Congestive heart failure
RPS28	Congestive heart failure
RPS28P6	Congestive heart failure
RPS28P9	Congestive heart failure
RPS29	Congestive heart failure
RPS29P11	Congestive heart failure
RPS29P16	Congestive heart failure
RPS29P17	Congestive heart failure
RPS29P3	Congestive heart failure
RPS29P9	Congestive heart failure
RPS7	Congestive heart failure
RPS7P10	Congestive heart failure
RPS7P11	Congestive heart failure
RPS7P4	Congestive heart failure
SCN4A	Congestive heart failure
SCN4B	Congestive heart failure
SCN5A	Congestive heart failure
SDHA	Congestive heart failure
SDHAF2	Congestive heart failure
SDHB	Congestive heart failure
SDHC	Congestive heart failure
SDHD	Congestive heart failure
SGCD	Congestive heart failure
SH3PXD2B	Congestive heart failure

SLC17A5	Congestive heart failure
SLC19A2	Congestive heart failure
SLC22A5	Congestive heart failure
SLC2A10	Congestive heart failure
SMAD4	Congestive heart failure
SNAP29	Congestive heart failure
SNTA1	Congestive heart failure
TAZ	Congestive heart failure
TBL2	Congestive heart failure
TCAP	Congestive heart failure
TF	Congestive heart failure
TMEM127	Congestive heart failure
TMEM70	Congestive heart failure
TMPO	Congestive heart failure
TNNC1	Congestive heart failure
TNNI3	Congestive heart failure
TNNI3K	Congestive heart failure
TNNT2	Congestive heart failure
TPI1	Congestive heart failure
TPI1P1	Congestive heart failure
TPM1	Congestive heart failure
TRIM37	Congestive heart failure
TRNE	Congestive heart failure
TRNF	Congestive heart failure
TRNH	Congestive heart failure
TRNK	Congestive heart failure
TRNL1	Congestive heart failure
TRNQ	Congestive heart failure
TRNS1	Congestive heart failure
TRNS2	Congestive heart failure
TRNW	Congestive heart failure

TSC1	Congestive heart failure
TSC2	Congestive heart failure
TSR2	Congestive heart failure
TTN	Congestive heart failure
TXNRD2	Congestive heart failure
VCL	Congestive heart failure
VHL	Congestive heart failure
WFS1	Congestive heart failure
WRN	Congestive heart failure
ZMPSTE24	Congestive heart failure
BTK	Cor pulmonale
CFTR	Cor pulmonale
DCTN4	Cor pulmonale
FLNA	Cor pulmonale
SFTPC	Cor pulmonale
SOX9	Cor pulmonale
STX1A	Cor pulmonale
TGFB1	Cor pulmonale
PKD1	Cortical tubers
TSC1	Cortical tubers
TSC2	Cortical tubers
BLNK	Crohn's disease
CD79A	Crohn's disease
CD79B	Crohn's disease
IGH@	Crohn's disease
IGHG1	Crohn's disease
IGHG3	Crohn's disease
IGHM	Crohn's disease
IGHV3-11	Crohn's disease
IGHV3-7	Crohn's disease
IGHV4-31	Crohn's disease

IGLL1	Crohn's disease
LRRC8A	Crohn's disease
PIK3R1	Crohn's disease
TCF3	Crohn's disease
COL6A2	Decreased pulmonary function
PLOD1	Decreased pulmonary function
IKBKAP	Decreased sensitivity to hypoxemia
ABCA3	Desquamative interstitial pneumonitis
SFTPB	Desquamative interstitial pneumonitis
SFTPC	Desquamative interstitial pneumonitis
ABCB6	Diaphragmatic eventration
CHRNG	Diaphragmatic eventration
GDF3	Diaphragmatic eventration
GDF6	Diaphragmatic eventration
IGHMBP2	Diaphragmatic eventration
LRP2	Diaphragmatic eventration
MAMLD1	Diaphragmatic eventration
MEGF10	Diaphragmatic eventration
MTM1	Diaphragmatic eventration
ODZ3	Diaphragmatic eventration
PLOD3	Diaphragmatic eventration
RARB	Diaphragmatic eventration
RBP4	Diaphragmatic eventration
SHH	Diaphragmatic eventration
STRA6	Diaphragmatic eventration
TENM3	Diaphragmatic eventration
VSX2	Diaphragmatic eventration
SMPD1	Diffuse reticular or finely nodular infiltrations
ADCY5	Dyskinesia
B4GALNT1	Dyskinesia
CDKL5	Dyskinesia

DDX3X	Dyskinesia
DNAJC13	Dyskinesia
EIF4G1	Dyskinesia
FOXG1	Dyskinesia
GBA	Dyskinesia
GIGYF2	Dyskinesia
LOC100133770	Dyskinesia
LRRK2	Dyskinesia
MECP2	Dyskinesia
NTNG1	Dyskinesia
PDGFB	Dyskinesia
PDGFRB	Dyskinesia
PNPT1	Dyskinesia
PRRT2	Dyskinesia
RAB39B	Dyskinesia
SLC20A2	Dyskinesia
SLC2A1	Dyskinesia
SLC46A1	Dyskinesia
SLC6A3	Dyskinesia
SNCA	Dyskinesia
VPS35	Dyskinesia
XPR1	Dyskinesia
ACTA2	Emphysema
ALDH18A1	Emphysema
B2M	Emphysema
CD19	Emphysema
CD81	Emphysema
CDC6	Emphysema
CDT1	Emphysema
CR2	Emphysema
DNASE1L3	Emphysema

EFEMP2	Emphysema
ELN	Emphysema
EVC	Emphysema
EVC2	Emphysema
FBLN5	Emphysema
FBN1	Emphysema
FLCN	Emphysema
GAA	Emphysema
GLA	Emphysema
ICOS	Emphysema
LRBA	Emphysema
LTBP4	Emphysema
MFAP5	Emphysema
MS4A1	Emphysema
MYH11	Emphysema
MYLK	Emphysema
NFKB1	Emphysema
NFKB2	Emphysema
ORC1	Emphysema
ORC1L	Emphysema
ORC4	Emphysema
ORC4L	Emphysema
ORC6	Emphysema
ORC6L	Emphysema
PRKCD	Emphysema
PRKG1	Emphysema
SERPINA1	Emphysema
SMAD3	Emphysema
SPINK5	Emphysema
TAP1	Emphysema
TAP2	Emphysema

TAPBP	Emphysema
TGFB2	Emphysema
TGFBR1	Emphysema
TGFBR2	Emphysema
TNFRSF13B	Emphysema
TNFRSF13C	Emphysema
TNFSF12	Emphysema
TNFSF12-TNFSF1	Emphysema
TNFSF13	Emphysema
TSC1	Emphysema
TSC2	Emphysema
EAF2	Emphysema
EDN1	Emphysema
EDNRB	Emphysema
APOPT1	Exertional dyspnea
ATP11A	Exertional dyspnea
ATP6	Exertional dyspnea
ATP8	Exertional dyspnea
C12ORF62	Exertional dyspnea
C14ORF153	Exertional dyspnea
CCDC56	Exertional dyspnea
COX1	Exertional dyspnea
COX10	Exertional dyspnea
COX14	Exertional dyspnea
COX2	Exertional dyspnea
COX20	Exertional dyspnea
COX3	Exertional dyspnea
COX6B1	Exertional dyspnea
CYB5R3	Exertional dyspnea
DNA2	Exertional dyspnea
DPP9	Exertional dyspnea

DSP	Exertional dyspnea
EPOR	Exertional dyspnea
FAM13A	Exertional dyspnea
FAM36A	Exertional dyspnea
FASTKD2	Exertional dyspnea
JAK2	Exertional dyspnea
LOC100131801	Exertional dyspnea
MT-CO1	Exertional dyspnea
MT-CO3	Exertional dyspnea
MUC5B	Exertional dyspnea
OBFC1	Exertional dyspnea
PARN	Exertional dyspnea
PET100	Exertional dyspnea
RTEL1	Exertional dyspnea
SCO1	Exertional dyspnea
SFTPA1	Exertional dyspnea
SFTPA2	Exertional dyspnea
SFTPC	Exertional dyspnea
SH2B3	Exertional dyspnea
TACO1	Exertional dyspnea
TERC	Exertional dyspnea
TERT	Exertional dyspnea
TNFRSF6B	Exertional dyspnea
ADAMTS13	Hemolytic-uremic syndrome
C3	Hemolytic-uremic syndrome
CD46	Hemolytic-uremic syndrome
CFB	Hemolytic-uremic syndrome
CFH	Hemolytic-uremic syndrome
CFHR1	Hemolytic-uremic syndrome
CFHR3	Hemolytic-uremic syndrome
CFHR5	Hemolytic-uremic syndrome

CFI	Hemolytic-uremic syndrome
DGKE	Hemolytic-uremic syndrome
LOC100133511	Hemolytic-uremic syndrome
LOC653879	Hemolytic-uremic syndrome
MMACHC	Hemolytic-uremic syndrome
THBD	Hemolytic-uremic syndrome
ACVRL1	Hemoptysis
C4A	Hemoptysis
CARD9	Hemoptysis
CLEC7A	Hemoptysis
COL3A1	Hemoptysis
COL5A1	Hemoptysis
CTLA4	Hemoptysis
DNASE1L3	Hemoptysis
ENG	Hemoptysis
GDF2	Hemoptysis
HLA-B	Hemoptysis
HLA-C	Hemoptysis
HLA-DPB1	Hemoptysis
HLAB	Hemoptysis
IL10	Hemoptysis
IL12A	Hemoptysis
IL12B	Hemoptysis
IL12RB2	Hemoptysis
IL17F	Hemoptysis
IL17RA	Hemoptysis
IL17RC	Hemoptysis
IL23R	Hemoptysis
MEFV	Hemoptysis
MLX	Hemoptysis
NOD2	Hemoptysis

PRTN3	Hemoptysis
PTPN22	Hemoptysis
SMAD4	Hemoptysis
STAT1	Hemoptysis
STAT4	Hemoptysis
TLR4	Hemoptysis
TRAF3IP2	Hemoptysis
TSC1	Hemoptysis
TSC2	Hemoptysis
ACVRL1	High-output congestive heart failure
ENG	High-output congestive heart failure
GDF2	High-output congestive heart failure
SMAD4	High-output congestive heart failure
Gene Symbol	HPO Phenotype
AGGF1	Hydrops fetalis
BSND	Hydrops fetalis
C15ORF41	Hydrops fetalis
CCBE1	Hydrops fetalis
CDAN1	Hydrops fetalis
CHRNA1	Hydrops fetalis
CHRND	Hydrops fetalis
CHRNG	Hydrops fetalis
CLCNKA	Hydrops fetalis
CLCNKB	Hydrops fetalis
COL11A1	Hydrops fetalis
COL11A2	Hydrops fetalis
COL2A1	Hydrops fetalis
DYNC2H1	Hydrops fetalis
FAT4	Hydrops fetalis
FIG4	Hydrops fetalis
FLNB	Hydrops fetalis

GBA	Hydrops fetalis
GBE1	Hydrops fetalis
GUSB	Hydrops fetalis
HADHA	Hydrops fetalis
HADHB	Hydrops fetalis
HBA1	Hydrops fetalis
HBA2	Hydrops fetalis
IFT80	Hydrops fetalis
KLF1	Hydrops fetalis
LOC341378	Hydrops fetalis
NEK1	Hydrops fetalis
NEU1	Hydrops fetalis
PTH1R	Hydrops fetalis
RAPSN	Hydrops fetalis
RYR1	Hydrops fetalis
SLC17A5	Hydrops fetalis
SLC26A2	Hydrops fetalis
SOX18	Hydrops fetalis
TRIP11	Hydrops fetalis
WDR34	Hydrops fetalis
WDR35	Hydrops fetalis
WDR60	Hydrops fetalis
WNT7A	Hydrops fetalis
AKT1	Hyperthyroidism
ARVCF	Hyperthyroidism
ATP6	Hyperthyroidism
ATP8	Hyperthyroidism
C7ORF10	Hyperthyroidism
CACNA1S	Hyperthyroidism
COMT	Hyperthyroidism
COX1	Hyperthyroidism

COX2	Hyperthyroidism
COX3	Hyperthyroidism
GABRA3	Hyperthyroidism
GNAS	Hyperthyroidism
GP1BB	Hyperthyroidism
HIRA	Hyperthyroidism
KCNJ12	Hyperthyroidism
KCNJ18	Hyperthyroidism
KILLIN	Hyperthyroidism
KLLN	Hyperthyroidism
LOC100130320	Hyperthyroidism
LOC100131509	Hyperthyroidism
MT-CO1	Hyperthyroidism
MT-CO3	Hyperthyroidism
MT-ND1	Hyperthyroidism
MT-ND5	Hyperthyroidism
MT-ND6	Hyperthyroidism
ND1	Hyperthyroidism
ND4	Hyperthyroidism
ND4L	Hyperthyroidism
ND5	Hyperthyroidism
ND6	Hyperthyroidism
PIK3CA	Hyperthyroidism
PTEN	Hyperthyroidism
PTENP1	Hyperthyroidism
SDHB	Hyperthyroidism
SDHC	Hyperthyroidism
SDHD	Hyperthyroidism
SUGCT	Hyperthyroidism
TBX1	Hyperthyroidism
THRB	Hyperthyroidism

TRNF	Hyperthyroidism
TRNH	Hyperthyroidism
TRNL1	Hyperthyroidism
TRNQ	Hyperthyroidism
TRNS1	Hyperthyroidism
TRNS2	Hyperthyroidism
TRNW	Hyperthyroidism
TSHR	Hyperthyroidism
UFD1L	Hyperthyroidism
GNASAS	Hyperthyroidism
GNMT	Hyperthyroidism
GPC3	Hyperthyroidism
GPC5	Hyperthyroidism
GPR44	Hyperthyroidism
GPX1	Hyperthyroidism
GPX3	Hyperthyroidism
GRB7	Hyperthyroidism
AHI1	Hyperventilation
ALS2CR4	Hyperventilation
ARL13B	Hyperventilation
ARX	Hyperventilation
B9D1	Hyperventilation
C5orf42	Hyperventilation
CASK	Hyperventilation
CDKL5	Hyperventilation
CEP41	Hyperventilation
CNTNAP2	Hyperventilation
CSPP1	Hyperventilation
DNM1	Hyperventilation
FBP1	Hyperventilation
FOXG1	Hyperventilation

GNAO1	Hyperventilation
GRIN2B	Hyperventilation
HCN1	Hyperventilation
HLCS	Hyperventilation
INPP5E	Hyperventilation
KCNB1	Hyperventilation
KCNQ2	Hyperventilation
KIAA0586	Hyperventilation
MECP2	Hyperventilation
MKS1	Hyperventilation
NECAP1	Hyperventilation
NRXN1	Hyperventilation
NTNG1	Hyperventilation
PIGA	Hyperventilation
PIGQ	Hyperventilation
PLCB1	Hyperventilation
PNKP	Hyperventilation
SCN2A	Hyperventilation
SCN8A	Hyperventilation
SIK1	Hyperventilation
SLC13A5	Hyperventilation
SLC25A22	Hyperventilation
SPTAN1	Hyperventilation
ST3GAL3	Hyperventilation
STXBP1	Hyperventilation
TCTN1	Hyperventilation
TCTN2	Hyperventilation
TMEM237	Hyperventilation
TMEM67	Hyperventilation
TSGA14	Hyperventilation
WWOX	Hyperventilation

BSND	Hypokalemic hypochloremic metabolic alkalosis
CLCNKA	Hypokalemic hypochloremic metabolic alkalosis
CLCNKB	Hypokalemic hypochloremic metabolic alkalosis
AVPR2	Hyponatremia
BSND	Hyponatremia
CA12	Hyponatremia
CLCNKA	Hyponatremia
CLCNKB	Hyponatremia
CPOX	Hyponatremia
CTNS	Hyponatremia
CYP11B2	Hyponatremia
HMBS	Hyponatremia
IKBKAP	Hyponatremia
NR0B1	Hyponatremia
NR3C2	Hyponatremia
OCRL	Hyponatremia
PRF1	Hyponatremia
SARS2	Hyponatremia
SCN4A	Hyponatremia
SCNN1A	Hyponatremia
SCNN1B	Hyponatremia
SCNN1G	Hyponatremia
SLC26A3	Hyponatremia
STX11	Hyponatremia
STXBP2	Hyponatremia
UNC13D	Hyponatremia
GREM1	Hyponatremia
GSDMB	Hyponatremia
GSN	Hyponatremia
GSR	Hyponatremia
BMP1	Hypoplastic pulmonary veins

COL1A1	Hypoplastic pulmonary veins
COL1A2	Hypoplastic pulmonary veins
CREB3L1	Hypoplastic pulmonary veins
CRTAP	Hypoplastic pulmonary veins
FKBP10	Hypoplastic pulmonary veins
LEPRE1	Hypoplastic pulmonary veins
P3H1	Hypoplastic pulmonary veins
PPIB	Hypoplastic pulmonary veins
SERPINF1	Hypoplastic pulmonary veins
SERPINH1	Hypoplastic pulmonary veins
SP7	Hypoplastic pulmonary veins
SPARC	Hypoplastic pulmonary veins
TMEM38B	Hypoplastic pulmonary veins
WNT1	Hypoplastic pulmonary veins
TRH	Hypothalamic hypothyroidism
ABCC6	Hypothyroidism
ACP5	Hypothyroidism
ADA	Hypothyroidism
AKT1	Hypothyroidism
ALMS1	Hypothyroidism
APC	Hypothyroidism
ARVCF	Hypothyroidism
ATP6	Hypothyroidism
ATP8	Hypothyroidism
BAZ1B	Hypothyroidism
BCOR	Hypothyroidism
BMP4	Hypothyroidism
BUB1	Hypothyroidism
BUB1B	Hypothyroidism
BUB3	Hypothyroidism
CARD9	Hypothyroidism

CD19	Hypothyroidism
CD81	Hypothyroidism
CDKN1C	Hypothyroidism
CDON	Hypothyroidism
CEP57	Hypothyroidism
CHD7	Hypothyroidism
CISD2	Hypothyroidism
CLEC7A	Hypothyroidism
CLIP2	Hypothyroidism
COMT	Hypothyroidism
COX1	Hypothyroidism
COX2	Hypothyroidism
COX3	Hypothyroidism
CP	Hypothyroidism
CR2	Hypothyroidism
DCLRE1C	Hypothyroidism
DISP1	Hypothyroidism
DLL1	Hypothyroidism
DUOX2	Hypothyroidism
DUOXA2	Hypothyroidism
EFEMP2	Hypothyroidism
EIF2AK3	Hypothyroidism
ELN	Hypothyroidism
ENPP1	Hypothyroidism
FBLN5	Hypothyroidism
FGF8	Hypothyroidism
FLII	Hypothyroidism
FMR1	Hypothyroidism
FOXE1	Hypothyroidism
FOXH1	Hypothyroidism
FOXI1	Hypothyroidism

FOXP3	Hypothyroidism
FUCA1	Hypothyroidism
GABRD	Hypothyroidism
GAS1	Hypothyroidism
GATA1	Hypothyroidism
GFAP	Hypothyroidism
GLI2	Hypothyroidism
GNAS	Hypothyroidism
GP1BB	Hypothyroidism
GPR161	Hypothyroidism
GTF2I	Hypothyroidism
GTF2IRD1	Hypothyroidism
H19	Hypothyroidism
H19-ICR	Hypothyroidism
HBB	Hypothyroidism
HESX1	Hypothyroidism
HIRA	Hypothyroidism
HNF1B	Hypothyroidism
HNF4A	Hypothyroidism
HPD	Hypothyroidism
HSD17B3	Hypothyroidism
ICOS	Hypothyroidism
IGSF1	Hypothyroidism
IL17F	Hypothyroidism
IL17RA	Hypothyroidism
IL17RC	Hypothyroidism
IL2RA	Hypothyroidism
IL2RG	Hypothyroidism
IL6R	Hypothyroidism
IL7R	Hypothyroidism
IYD	Hypothyroidism

KANSL1	Hypothyroidism
KAT6B	Hypothyroidism
KCNAB2	Hypothyroidism
KCNJ10	Hypothyroidism
KCNQ1OT1	Hypothyroidism
KIAA1267	Hypothyroidism
KILLIN	Hypothyroidism
KISS1R	Hypothyroidism
KLLN	Hypothyroidism
LCRB	Hypothyroidism
LHX3	Hypothyroidism
LHX4	Hypothyroidism
LIFR	Hypothyroidism
LIG4	Hypothyroidism
LIMK1	Hypothyroidism
LOC100093631	Hypothyroidism
LOC100130320	Hypothyroidism
LRBA	Hypothyroidism
LRP4	Hypothyroidism
MCM8	Hypothyroidism
MS4A1	Hypothyroidism
MT-CO1	Hypothyroidism
MT-CO3	Hypothyroidism
MT-ND1	Hypothyroidism
MT-ND5	Hypothyroidism
MT-ND6	Hypothyroidism
MYST4	Hypothyroidism
NAA10	Hypothyroidism
ND1	Hypothyroidism
ND4	Hypothyroidism
ND4L	Hypothyroidism

ND5	Hypothyroidism
ND6	Hypothyroidism
NFKB1	Hypothyroidism
NFKB2	Hypothyroidism
NKX2-5	Hypothyroidism
NODAL	Hypothyroidism
NPHS1	Hypothyroidism
NSD1	Hypothyroidism
OTX2	Hypothyroidism
PAX8	Hypothyroidism
PIK3CA	Hypothyroidism
PMM2	Hypothyroidism
POU1F1	Hypothyroidism
PRDM16	Hypothyroidism
PRKCD	Hypothyroidism
PROP1	Hypothyroidism
PTCH1	Hypothyroidism
PTEN	Hypothyroidism
PTENP1	Hypothyroidism
PTRH2	Hypothyroidism
RAG1	Hypothyroidism
RAG2	Hypothyroidism
RAI1	Hypothyroidism
RFC2	Hypothyroidism
RMRP	Hypothyroidism
SALL1	Hypothyroidism
SCN4A	Hypothyroidism
SDHB	Hypothyroidism
SDHC	Hypothyroidism
SDHD	Hypothyroidism
SEMA3E	Hypothyroidism

SETBP1	Hypothyroidism
SHH	Hypothyroidism
SIX3	Hypothyroidism
SKI	Hypothyroidism
SLC16A2	Hypothyroidism
SLC26A4	Hypothyroidism
SLC5A5	Hypothyroidism
SOX3	Hypothyroidism
STAT1	Hypothyroidism
STAT3	Hypothyroidism
TBL2	Hypothyroidism
TBX1	Hypothyroidism
TDGF1	Hypothyroidism
TDGF3	Hypothyroidism
TF	Hypothyroidism
TG	Hypothyroidism
TGIF1	Hypothyroidism
THRA	Hypothyroidism
THRB	Hypothyroidism
TNFRSF13B	Hypothyroidism
TNFRSF13C	Hypothyroidism
TNFSF12	Hypothyroidism
TNFSF12-TNFSF1	Hypothyroidism
TNFSF13	Hypothyroidism
TPO	Hypothyroidism
TRAF3IP2	Hypothyroidism
TRHR	Hypothyroidism
TRNF	Hypothyroidism
TRNH	Hypothyroidism
TRNL1	Hypothyroidism
TRNQ	Hypothyroidism

TRNS1	Hypothyroidism
TRNS2	Hypothyroidism
TRNW	Hypothyroidism
TSC1	Hypothyroidism
TSC2	Hypothyroidism
TSHR	Hypothyroidism
UBR1	Hypothyroidism
UFD1L	Hypothyroidism
VIPAR	Hypothyroidism
VIPAS39	Hypothyroidism
VPS33B	Hypothyroidism
WFS1	Hypothyroidism
ZIC2	Hypothyroidism
GSTM1	Hypothyroidism
DCTN1	Hypoventilation
DMD	Hypoventilation
LTBP4	Hypoventilation
MOGS	Hypoventilation
NDN	Hypoventilation
SNRPN	Hypoventilation
SNURF	Hypoventilation
BMPR2	Increased pulmonary vascular resistance
CAV1	Increased pulmonary vascular resistance
SMAD9	Increased pulmonary vascular resistance
AGRN	Intermittent episodes of respiratory insufficiency due to muscle weakness
CHRNA1	Intermittent episodes of respiratory insufficiency due to muscle weakness
CHRNB1	Intermittent episodes of respiratory insufficiency due to muscle weakness
CHRND	Intermittent episodes of respiratory insufficiency due to muscle weakness
CHRNE	Intermittent episodes of respiratory insufficiency due to muscle weakness
DOK7	Intermittent episodes of respiratory insufficiency due to muscle weakness
LRP4	Intermittent episodes of respiratory insufficiency due to muscle weakness

MUSK	Intermittent episodes of respiratory insufficiency due to muscle weakness
RAPSN	Intermittent episodes of respiratory insufficiency due to muscle weakness
SCN4A	Intermittent episodes of respiratory insufficiency due to muscle weakness
SUCLG1	Intermittent hyperpnea at rest
CDKL5	Intermittent hyperventilation
FOXG1	Intermittent hyperventilation
MECP2	Intermittent hyperventilation
NTNG1	Intermittent hyperventilation
TCF4	Intermittent hyperventilation
C16ORF57	Interstitial pneumonitis
C17ORF68	Interstitial pneumonitis
CTC1	Interstitial pneumonitis
DKC1	Interstitial pneumonitis
NHP2	Interstitial pneumonitis
NOP10	Interstitial pneumonitis
PARN	Interstitial pneumonitis
PLCG2	Interstitial pneumonitis
RTEL1	Interstitial pneumonitis
SFTPC	Interstitial pneumonitis
STAT3	Interstitial pneumonitis
TERC	Interstitial pneumonitis
TERT	Interstitial pneumonitis
TINF2	Interstitial pneumonitis
TNFRSF6B	Interstitial pneumonitis
USB1	Interstitial pneumonitis
WRAP53	Interstitial pneumonitis
BTNL2	Interstitial pulmonary disease
FGD1	Interstitial pulmonary disease
GBA	Interstitial pulmonary disease
HLA-DRB1	Interstitial pulmonary disease
HLA-DRB4	Interstitial pulmonary disease

IL1RN	Interstitial pulmonary disease
ITGA3	Interstitial pulmonary disease
MARS	Interstitial pulmonary disease
SCARB2	Interstitial pulmonary disease
SFTPB	Interstitial pulmonary disease
TMEM173	Interstitial pulmonary disease
SLC34A2	Intraalveolar nodular calcifications
HADHA	Long chain 3 hydroxyacyl coA dehydrogenase deficiency
CPT2	Long-chain dicarboxylic aciduria
FH	Loss of voice
LOC100130320	Loss of voice
MAX	Loss of voice
RET	Loss of voice
SDHA	Loss of voice
SDHAF2	Loss of voice
SDHB	Loss of voice
SDHC	Loss of voice
SDHD	Loss of voice
TMEM127	Loss of voice
NPC1	Low cholesterol esterification rates
NPC2	Low cholesterol esterification rates
BAP1	Lung adenocarcinoma
CHEK2	Lung adenocarcinoma
LOC100133012	Lung adenocarcinoma
LOC646096	Lung adenocarcinoma
MDM2	Lung adenocarcinoma
TP53	Lung adenocarcinoma
BRCA2	Lung segmentation defects
BRIP1	Lung segmentation defects
BTBD12	Lung segmentation defects
ERCC4	Lung segmentation defects

FANCA	Lung segmentation defects
FANCB	Lung segmentation defects
FANCC	Lung segmentation defects
FANCD2	Lung segmentation defects
FANCE	Lung segmentation defects
FANCF	Lung segmentation defects
FANCG	Lung segmentation defects
FANCI	Lung segmentation defects
FANCL	Lung segmentation defects
FANCM	Lung segmentation defects
GPC3	Lung segmentation defects
GPC4	Lung segmentation defects
PALB2	Lung segmentation defects
RAD51C	Lung segmentation defects
SLX4	Lung segmentation defects
UBE2T	Lung segmentation defects
CORIN	Maternal hypertension
NR3C2	Maternal hypertension
STOX1	Maternal hypertension
WT1	Mesothelioma
NCR3	Mesothelioma
NF2	Mesothelioma
NFE2L2	Mesothelioma
NFYA	Mesothelioma
NGF	Mesothelioma
NKX2-1	Mesothelioma
NLRP3	Mesothelioma
NLRP7	Mesothelioma
NME1	Mesothelioma
NME1-NME2	Mesothelioma
NME2	Mesothelioma

NOD2	Mesothelioma
NOS1	Mesothelioma
NOS2	Mesothelioma
NOS3	Mesothelioma
NOTCH2	Mesothelioma
NOTCH3	Mesothelioma
NPHS1	Mesothelioma
NPHS2	Mesothelioma
NPPA	Mesothelioma
NPPB	Mesothelioma
NPPC	Mesothelioma
NPSR1	Mesothelioma
NPY	Mesothelioma
NQO1	Mesothelioma
GJA1	Mitral atresia
NKX2-5	Mitral atresia
ABCC9	Mitral regurgitation
ACTC1	Mitral regurgitation
ACTN2	Mitral regurgitation
ADAMTS10	Mitral regurgitation
ADAMTSL2	Mitral regurgitation
AGA	Mitral regurgitation
AKT3	Mitral regurgitation
ALDH18A1	Mitral regurgitation
B3GALT6	Mitral regurgitation
BAG3	Mitral regurgitation
BAZ1B	Mitral regurgitation
CBL	Mitral regurgitation
CCND2	Mitral regurgitation
CHST3	Mitral regurgitation
CLIP2	Mitral regurgitation

COL1A2	Mitral regurgitation
CRYAB	Mitral regurgitation
CSRP3	Mitral regurgitation
DCHS1	Mitral regurgitation
DES	Mitral regurgitation
DMD	Mitral regurgitation
DOLK	Mitral regurgitation
DSG2	Mitral regurgitation
DTNA	Mitral regurgitation
ELN	Mitral regurgitation
FBLN5	Mitral regurgitation
FBN1	Mitral regurgitation
FBN2	Mitral regurgitation
FHL2	Mitral regurgitation
FKTN	Mitral regurgitation
FLNA	Mitral regurgitation
G6PC3	Mitral regurgitation
GATAD1	Mitral regurgitation
GTF2I	Mitral regurgitation
GTF2IRD1	Mitral regurgitation
IDUA	Mitral regurgitation
IRX5	Mitral regurgitation
LAMA4	Mitral regurgitation
LDB3	Mitral regurgitation
LIMK1	Mitral regurgitation
LMNA	Mitral regurgitation
LOC100093631	Mitral regurgitation
LOC653348	Mitral regurgitation
LTBP2	Mitral regurgitation
MIB1	Mitral regurgitation
MYBPC3	Mitral regurgitation

MYH6	Mitral regurgitation
MYH7	Mitral regurgitation
MYH7B	Mitral regurgitation
MYPN	Mitral regurgitation
NEXN	Mitral regurgitation
PDSS1	Mitral regurgitation
PDSS1P1	Mitral regurgitation
PIK3R2	Mitral regurgitation
PLN	Mitral regurgitation
POLG	Mitral regurgitation
PRDM16	Mitral regurgitation
PSEN1	Mitral regurgitation
PSEN2	Mitral regurgitation
RAF1	Mitral regurgitation
RBM20	Mitral regurgitation
RFC2	Mitral regurgitation
RPS6KA3	Mitral regurgitation
SCN5A	Mitral regurgitation
SDHA	Mitral regurgitation
SGCD	Mitral regurgitation
SGOL1	Mitral regurgitation
SMAD3	Mitral regurgitation
SMAD4	Mitral regurgitation
TAZ	Mitral regurgitation
TBL2	Mitral regurgitation
TCAP	Mitral regurgitation
TGFB3	Mitral regurgitation
TK2	Mitral regurgitation
TMPO	Mitral regurgitation
TNNC1	Mitral regurgitation
TNNI3	Mitral regurgitation

TNNT2	Mitral regurgitation
TPM1	Mitral regurgitation
TTN	Mitral regurgitation
TXNRD2	Mitral regurgitation
VCL	Mitral regurgitation
A2ML1	Mitral stenosis
ABCC6	Mitral stenosis
ADAMTSL2	Mitral stenosis
BRAF	Mitral stenosis
CHST3	Mitral stenosis
ENPP1	Mitral stenosis
FAM58A	Mitral stenosis
FBN1	Mitral stenosis
GBA	Mitral stenosis
HGD	Mitral stenosis
KAT6B	Mitral stenosis
KRAS	Mitral stenosis
LOC344593	Mitral stenosis
LOC401218	Mitral stenosis
LOC442113	Mitral stenosis
LOC653348	Mitral stenosis
LZTR1	Mitral stenosis
MYST4	Mitral stenosis
NOTCH2	Mitral stenosis
NRAS	Mitral stenosis
PRKAR1A	Mitral stenosis
PTPN11	Mitral stenosis
RAF1	Mitral stenosis
RASA2	Mitral stenosis
RIT1	Mitral stenosis
SOS1	Mitral stenosis

SOS2	Mitral stenosis
CACNA1S	Mixed respiratory and metabolic acidosis
RYR1	Mixed respiratory and metabolic acidosis
ALMS1	Multinodular goiter
DICER1	Multinodular goiter
CALR	Myelofibrosis
GFI1B	Myelofibrosis
JAK2	Myelofibrosis
MPL	Myelofibrosis
NBEAL2	Myelofibrosis
SH2B3	Myelofibrosis
TBXAS1	Myelofibrosis
TET2	Myelofibrosis
AHI1	Neonatal breathing dysregulation
ALS2CR4	Neonatal breathing dysregulation
ARL13B	Neonatal breathing dysregulation
B9D1	Neonatal breathing dysregulation
C5orf42	Neonatal breathing dysregulation
CC2D2A	Neonatal breathing dysregulation
CEP290	Neonatal breathing dysregulation
CEP41	Neonatal breathing dysregulation
CSPP1	Neonatal breathing dysregulation
INPP5E	Neonatal breathing dysregulation
KIAA0586	Neonatal breathing dysregulation
MKS1	Neonatal breathing dysregulation
TCTN1	Neonatal breathing dysregulation
TCTN2	Neonatal breathing dysregulation
TMEM138	Neonatal breathing dysregulation
TMEM216	Neonatal breathing dysregulation
TMEM231	Neonatal breathing dysregulation
TMEM237	Neonatal breathing dysregulation

TMEM67	Neonatal breathing dysregulation
TSGA14	Neonatal breathing dysregulation
ZNF423	Neonatal breathing dysregulation
AKT2	Neonatal hypoglycemia
C2ORF34	Neonatal hypoglycemia
CAMKMT	Neonatal hypoglycemia
CDKN1C	Neonatal hypoglycemia
DBH	Neonatal hypoglycemia
GLI2	Neonatal hypoglycemia
GYS2	Neonatal hypoglycemia
H19	Neonatal hypoglycemia
H19-ICR	Neonatal hypoglycemia
HESX1	Neonatal hypoglycemia
KCNQ1OT1	Neonatal hypoglycemia
KDM6A	Neonatal hypoglycemia
KMT2D	Neonatal hypoglycemia
LHX4	Neonatal hypoglycemia
MLL2	Neonatal hypoglycemia
NSD1	Neonatal hypoglycemia
OTX2	Neonatal hypoglycemia
POU1F1	Neonatal hypoglycemia
PPM1B	Neonatal hypoglycemia
PREPL	Neonatal hypoglycemia
PRKAG2	Neonatal hypoglycemia
PROP1	Neonatal hypoglycemia
SLC3A1	Neonatal hypoglycemia
SOX3	Neonatal hypoglycemia
ALDH7A1	Neonatal respiratory distress
ARMC4	Neonatal respiratory distress
C14ORF104	Neonatal respiratory distress
C19ORF51	Neonatal respiratory distress

C21ORF59	Neonatal respiratory distress
C2ORF39	Neonatal respiratory distress
CCDC103	Neonatal respiratory distress
CCDC114	Neonatal respiratory distress
CCDC151	Neonatal respiratory distress
CCDC39	Neonatal respiratory distress
CCDC40	Neonatal respiratory distress
CCDC65	Neonatal respiratory distress
CCDC8	Neonatal respiratory distress
CCNO	Neonatal respiratory distress
CHRNG	Neonatal respiratory distress
CUL7	Neonatal respiratory distress
DNAAF1	Neonatal respiratory distress
DNAAF2	Neonatal respiratory distress
DNAAF3	Neonatal respiratory distress
DNAAF5	Neonatal respiratory distress
DNAH11	Neonatal respiratory distress
DNAH5	Neonatal respiratory distress
DNAI1	Neonatal respiratory distress
DNAI2	Neonatal respiratory distress
DNAL1	Neonatal respiratory distress
DRC1	Neonatal respiratory distress
DYX1C1	Neonatal respiratory distress
FAM187A	Neonatal respiratory distress
GAS8	Neonatal respiratory distress
HEATR2	Neonatal respiratory distress
HYDIN	Neonatal respiratory distress
LOC652460	Neonatal respiratory distress
LRRC50	Neonatal respiratory distress
LRRC6	Neonatal respiratory distress
MAMLD1	Neonatal respiratory distress

MTM1	Neonatal respiratory distress
NKX2-1	Neonatal respiratory distress
NME8	Neonatal respiratory distress
NPHS1	Neonatal respiratory distress
OBSL1	Neonatal respiratory distress
OFD1	Neonatal respiratory distress
PLEC	Neonatal respiratory distress
RPGR	Neonatal respiratory distress
RSPH1	Neonatal respiratory distress
RSPH3	Neonatal respiratory distress
RSPH4A	Neonatal respiratory distress
RSPH9	Neonatal respiratory distress
RUNX2	Neonatal respiratory distress
SBDS	Neonatal respiratory distress
SBDSP1	Neonatal respiratory distress
SFTPB	Neonatal respiratory distress
SFTPC	Neonatal respiratory distress
SNRPB	Neonatal respiratory distress
SOX9	Neonatal respiratory distress
SPAG1	Neonatal respiratory distress
TXNDC3	Neonatal respiratory distress
UBE3B	Neonatal respiratory distress
ZC4H2	Neonatal respiratory distress
ZMYND10	Neonatal respiratory distress
AKT1	Neoplasm of the lung
CHEK2	Neoplasm of the lung
DDIT3	Neoplasm of the lung
DIS3L2	Neoplasm of the lung
EWSR1	Neoplasm of the lung
FUS	Neoplasm of the lung
H19	Neoplasm of the lung

HPGD	Neoplasm of the lung
IGHV4-34	Neoplasm of the lung
LOC100133012	Neoplasm of the lung
LOC284685	Neoplasm of the lung
LOC646096	Neoplasm of the lung
LOC652522	Neoplasm of the lung
MBTPS2	Neoplasm of the lung
MDM2	Neoplasm of the lung
NOTCH3	Neoplasm of the lung
PDGFRB	Neoplasm of the lung
POU6F2	Neoplasm of the lung
PTEN	Neoplasm of the lung
PTENP1	Neoplasm of the lung
RB1	Neoplasm of the lung
SLCO2A1	Neoplasm of the lung
STK11	Neoplasm of the lung
TP53	Neoplasm of the lung
TP73	Neoplasm of the lung
TRPV3	Neoplasm of the lung
WRN	Neoplasm of the lung
WT1	Neoplasm of the lung
IFNG	Neoplasm of the lung
IFNGR1	Neoplasm of the lung
IFNL3	Neoplasm of the lung
IFT80	Neoplasm of the lung
IGBP1	Neoplasm of the lung
IGF1	Neoplasm of the lung
IGF1R	Neoplasm of the lung
IGF2	Neoplasm of the lung
IGF2R	Neoplasm of the lung
IKBKG	Neoplasm of the lung

IL10	Neoplasm of the lung
IL10RB	Neoplasm of the lung
IL12B	Neoplasm of the lung
IL13	Neoplasm of the lung
IL17A	Neoplasm of the lung
IL18	Neoplasm of the lung
IL1A	Neoplasm of the lung
IL1B	Neoplasm of the lung
IL1R1	Neoplasm of the lung
IL1R2	Neoplasm of the lung
IL1RL1	Neoplasm of the lung
IL1RN	Neoplasm of the lung
IL2	Neoplasm of the lung
IL23R	Neoplasm of the lung
IL24	Neoplasm of the lung
IL28B	Neoplasm of the lung
IL2RA	Neoplasm of the lung
IL2RB	Neoplasm of the lung
IL33	Neoplasm of the lung
IL4	Neoplasm of the lung
IL5	Neoplasm of the lung
IL5RA	Neoplasm of the lung
IL6	Neoplasm of the lung
IL6ST	Neoplasm of the lung
IL8	Neoplasm of the lung
INS	Neoplasm of the lung
INSR	Neoplasm of the lung
IPCEF1	Neoplasm of the lung
IRAK3	Neoplasm of the lung
IRF1	Neoplasm of the lung
IRGM	Neoplasm of the lung

IRX1	Neoplasm of the lung
IRX2	Neoplasm of the lung
IRX3	Neoplasm of the lung
IRX5	Neoplasm of the lung
ITGB2	Neoplasm of the lung
ITGB6	Neoplasm of the lung
ITLN1	Neoplasm of the lung
JAG1	Neoplasm of the lung
JUN	Neoplasm of the lung
JUNB	Neoplasm of the lung
KEAP1	Neoplasm of the lung
KIF3A	Neoplasm of the lung
KIT	Neoplasm of the lung
KL	Neoplasm of the lung
KLF1	Neoplasm of the lung
KLF2	Neoplasm of the lung
KLHL3	Neoplasm of the lung
KMT2A	Neoplasm of the lung
KNG1	Neoplasm of the lung
KRAS	Neoplasm of the lung
KRT19	Neoplasm of the lung
LCP1	Neoplasm of the lung
LDHC	Neoplasm of the lung
LEPR	Neoplasm of the lung
LMO1	Neoplasm of the lung
LMO2	Neoplasm of the lung
LNCR1	Neoplasm of the lung
LNCR3	Neoplasm of the lung
LNCR4	Neoplasm of the lung
LOC100129500	Neoplasm of the lung
LOC100130902	Neoplasm of the lung

LOC100132369	Neoplasm of the lung
LOC100132771	Neoplasm of the lung
LOC100133583	Neoplasm of the lung
LOC100133678	Neoplasm of the lung
LOC100271831	Neoplasm of the lung
LOC220077	Neoplasm of the lung
LOC441454	Neoplasm of the lung
LOC642132	Neoplasm of the lung
LOC646626	Neoplasm of the lung
LOC652799	Neoplasm of the lung
LOC653882	Neoplasm of the lung
LOC728026	Neoplasm of the lung
LOC731751	Neoplasm of the lung
LOX	Neoplasm of the lung
LOXL2	Neoplasm of the lung
LTB4R	Neoplasm of the lung
LTBR	Neoplasm of the lung
LTC4S	Neoplasm of the lung
LVNC2	Neoplasm of the lung
LYL1	Neoplasm of the lung
LYSMD3	Neoplasm of the lung
MAOB	Neoplasm of the lung
MAP2K7	Neoplasm of the lung
MAP3K8	Neoplasm of the lung
MAPK1	Neoplasm of the lung
MAPK14	Neoplasm of the lung
MAPK3	Neoplasm of the lung
MAPT	Neoplasm of the lung
MASP2	Neoplasm of the lung
MC3R	Neoplasm of the lung
MCL1	Neoplasm of the lung

MECOM	Neoplasm of the lung
MECP2	Neoplasm of the lung
MEGF10	Neoplasm of the lung
MERTK	Neoplasm of the lung
MET	Neoplasm of the lung
MGME1	Neoplasm of the lung
MINA	Neoplasm of the lung
MIR130A	Neoplasm of the lung
MIR143	Neoplasm of the lung
MIR145	Neoplasm of the lung
MIR155	Neoplasm of the lung
MIR155HG	Neoplasm of the lung
MIR31	Neoplasm of the lung
ADA	Nephrotic syndrome
ADAR	Nephrotic syndrome
ADCK4	Nephrotic syndrome
ANLN	Nephrotic syndrome
APOA1	Nephrotic syndrome
ARHGDIA	Nephrotic syndrome
ARL6	Nephrotic syndrome
ATP6	Nephrotic syndrome
ATP8	Nephrotic syndrome
ATRIP	Nephrotic syndrome
BBIP1	Nephrotic syndrome
BBS1	Nephrotic syndrome
BBS10	Nephrotic syndrome
BBS12	Nephrotic syndrome
BBS2	Nephrotic syndrome
BBS4	Nephrotic syndrome
BBS5	Nephrotic syndrome
BBS7	Nephrotic syndrome

BBS9	Nephrotic syndrome
C2ORF86	Nephrotic syndrome
C3	Nephrotic syndrome
CASP10	Nephrotic syndrome
CEP290	Nephrotic syndrome
CFH	Nephrotic syndrome
CHD7	Nephrotic syndrome
CHST14	Nephrotic syndrome
COL4A3	Nephrotic syndrome
COL4A4	Nephrotic syndrome
COL4A5	Nephrotic syndrome
COQ2	Nephrotic syndrome
COQ6	Nephrotic syndrome
COX1	Nephrotic syndrome
COX2	Nephrotic syndrome
COX3	Nephrotic syndrome
CRB2	Nephrotic syndrome
DCLRE1C	Nephrotic syndrome
DGKE	Nephrotic syndrome
DSE	Nephrotic syndrome
EMP2	Nephrotic syndrome
FAS	Nephrotic syndrome
FASLG	Nephrotic syndrome
FGA	Nephrotic syndrome
FN1	Nephrotic syndrome
GATA3	Nephrotic syndrome
GLA	Nephrotic syndrome
GSN	Nephrotic syndrome
IFIH1	Nephrotic syndrome
IFT172	Nephrotic syndrome
IFT27	Nephrotic syndrome

IL2RG	Nephrotic syndrome
IL7R	Nephrotic syndrome
INF2	Nephrotic syndrome
ITGA3	Nephrotic syndrome
LAMB2	Nephrotic syndrome
LIG4	Nephrotic syndrome
LMNB2	Nephrotic syndrome
LMX1B	Nephrotic syndrome
LOC100133511	Nephrotic syndrome
LOC653879	Nephrotic syndrome
LYZ	Nephrotic syndrome
LZTFL1	Nephrotic syndrome
MEFV	Nephrotic syndrome
MKKS	Nephrotic syndrome
MKS1	Nephrotic syndrome
MT-CO1	Nephrotic syndrome
MT-CO3	Nephrotic syndrome
MT-ND1	Nephrotic syndrome
MT-ND5	Nephrotic syndrome
MT-ND6	Nephrotic syndrome
MYO1E	Nephrotic syndrome
NCRNA00081	Nephrotic syndrome
ND1	Nephrotic syndrome
ND4	Nephrotic syndrome
ND4L	Nephrotic syndrome
ND5	Nephrotic syndrome
ND6	Nephrotic syndrome
NLRP3	Nephrotic syndrome
NPHP1	Nephrotic syndrome
NPHS1	Nephrotic syndrome
NPHS2	Nephrotic syndrome

PAX2	Nephrotic syndrome
PDSS2	Nephrotic syndrome
PLCE1	Nephrotic syndrome
PMM2	Nephrotic syndrome
PRKCD	Nephrotic syndrome
PTPRO	Nephrotic syndrome
RABL4	Nephrotic syndrome
RAG1	Nephrotic syndrome
RAG2	Nephrotic syndrome
RMRP	Nephrotic syndrome
RNASEH2A	Nephrotic syndrome
RNASEH2B	Nephrotic syndrome
RNASEH2C	Nephrotic syndrome
SAMHD1	Nephrotic syndrome
SCARB2	Nephrotic syndrome
SDCCAG8	Nephrotic syndrome
SERPINA1	Nephrotic syndrome
SLC17A5	Nephrotic syndrome
SMARCAL1	Nephrotic syndrome
SNAP29	Nephrotic syndrome
TBX18	Nephrotic syndrome
TREX1	Nephrotic syndrome
TRIM32	Nephrotic syndrome
TRNF	Nephrotic syndrome
TRNH	Nephrotic syndrome
TRNL1	Nephrotic syndrome
TRNQ	Nephrotic syndrome
TRNS1	Nephrotic syndrome
TRNS2	Nephrotic syndrome
TRNW	Nephrotic syndrome
TRPC6	Nephrotic syndrome

TTC8	Nephrotic syndrome
WDPCP	Nephrotic syndrome
WDR73	Nephrotic syndrome
WT1	Nephrotic syndrome
ACTA1	Nocturnal hypoventilation
C20ORF54	Nocturnal hypoventilation
COL12A1	Nocturnal hypoventilation
COL6A1	Nocturnal hypoventilation
COL6A2	Nocturnal hypoventilation
COL6A3	Nocturnal hypoventilation
FKRP	Nocturnal hypoventilation
GPR172A	Nocturnal hypoventilation
MYH7	Nocturnal hypoventilation
SEPN1	Nocturnal hypoventilation
SLC52A2	Nocturnal hypoventilation
SLC52A3	Nocturnal hypoventilation
TTN	Nocturnal hypoventilation
ARMC4	Obstructive lung disease
C14ORF104	Obstructive lung disease
C19ORF51	Obstructive lung disease
C21ORF59	Obstructive lung disease
C2ORF39	Obstructive lung disease
CCDC103	Obstructive lung disease
CCDC114	Obstructive lung disease
CCDC151	Obstructive lung disease
CCDC39	Obstructive lung disease
CCDC40	Obstructive lung disease
CCDC65	Obstructive lung disease
CCNO	Obstructive lung disease
DNAAF1	Obstructive lung disease
DNAAF2	Obstructive lung disease

DNAAF3	Obstructive lung disease
DNAAF5	Obstructive lung disease
DNAH11	Obstructive lung disease
DNAH5	Obstructive lung disease
DNAI1	Obstructive lung disease
DNAI2	Obstructive lung disease
DNAL1	Obstructive lung disease
DRC1	Obstructive lung disease
DYX1C1	Obstructive lung disease
FAM187A	Obstructive lung disease
GAS8	Obstructive lung disease
GLA	Obstructive lung disease
HEATR2	Obstructive lung disease
HYDIN	Obstructive lung disease
LRRC50	Obstructive lung disease
LRRC6	Obstructive lung disease
NME8	Obstructive lung disease
OFD1	Obstructive lung disease
RPGR	Obstructive lung disease
RSPH1	Obstructive lung disease
RSPH3	Obstructive lung disease
RSPH4A	Obstructive lung disease
RSPH9	Obstructive lung disease
SPAG1	Obstructive lung disease
TXNDC3	Obstructive lung disease
ZMYND10	Obstructive lung disease
ICAM1	Obstructive lung disease
AHDC1	Obstructive sleep apnea
CREBBP	Obstructive sleep apnea
DNA2	Obstructive sleep apnea
FBN1	Obstructive sleep apnea

HRAS	Obstructive sleep apnea
IDS	Obstructive sleep apnea
IDUA	Obstructive sleep apnea
NFIX	Obstructive sleep apnea
SKI	Obstructive sleep apnea
SLC29A3	Obstructive sleep apnea
SP9	Obstructive sleep apnea
TRPV4	Obstructive sleep apnea
ABCB4	Pancreatitis
ACTG2	Pancreatitis
AGPAT2	Pancreatitis
AP2S1	Pancreatitis
APOC2	Pancreatitis
APOC4-APOC2	Pancreatitis
ATP6	Pancreatitis
ATP8	Pancreatitis
ATP8B1	Pancreatitis
BCKDHA	Pancreatitis
BCKDHB	Pancreatitis
BSCL2	Pancreatitis
C4A	Pancreatitis
CASR	Pancreatitis
CAV1	Pancreatitis
CBS	Pancreatitis
CFTR	Pancreatitis
COX1	Pancreatitis
COX2	Pancreatitis
COX3	Pancreatitis
CPA1	Pancreatitis
CTLA4	Pancreatitis
CTRC	Pancreatitis

DBT	Pancreatitis
FOS	Pancreatitis
G6PC	Pancreatitis
GNA11	Pancreatitis
GPIHBP1	Pancreatitis
HLA-B	Pancreatitis
HLA-C	Pancreatitis
HLA-DPB1	Pancreatitis
HLAB	Pancreatitis
IKZF1	Pancreatitis
IL10	Pancreatitis
IL12A	Pancreatitis
IL12RB2	Pancreatitis
IL23R	Pancreatitis
LMF1	Pancreatitis
LMNA	Pancreatitis
LPL	Pancreatitis
MEFV	Pancreatitis
MT-CO1	Pancreatitis
MT-CO3	Pancreatitis
MT-ND1	Pancreatitis
MT-ND5	Pancreatitis
MT-ND6	Pancreatitis
MUT	Pancreatitis
ND1	Pancreatitis
ND4	Pancreatitis
ND4L	Pancreatitis
ND5	Pancreatitis
ND6	Pancreatitis
NOD2	Pancreatitis
PCCA	Pancreatitis

PCCB	Pancreatitis
PPARG	Pancreatitis
PPM1K	Pancreatitis
PRSS1	Pancreatitis
PRSS2	Pancreatitis
PRTN3	Pancreatitis
PTPN22	Pancreatitis
SLC25A13	Pancreatitis
SLC37A4	Pancreatitis
SLC7A7	Pancreatitis
SPINK1	Pancreatitis
STAT4	Pancreatitis
TLR4	Pancreatitis
TRNF	Pancreatitis
TRNH	Pancreatitis
TRNL1	Pancreatitis
TRNQ	Pancreatitis
TRNS1	Pancreatitis
TRNS2	Pancreatitis
TRNW	Pancreatitis
TRY6	Pancreatitis
KCNMA1	Paroxysmal dyskinesia
PRRT2	Paroxysmal dyskinesia
B3GAT3	Patent foramen ovale
B3GAT3P1	Patent foramen ovale
C8ORF62	Patent foramen ovale
CACNA1D	Patent foramen ovale
CHST14	Patent foramen ovale
COL11A1	Patent foramen ovale
COL11A2	Patent foramen ovale
DSE	Patent foramen ovale

GABRD	Patent foramen ovale
GATA6	Patent foramen ovale
KCNAB2	Patent foramen ovale
LTBP4	Patent foramen ovale
PHGDH	Patent foramen ovale
PRDM16	Patent foramen ovale
PSAT1	Patent foramen ovale
RAI1	Patent foramen ovale
SKI	Patent foramen ovale
STAMBP	Patent foramen ovale
TALDO1	Patent foramen ovale
TBX20	Patent foramen ovale
TGFB3	Patent foramen ovale
TSFM	Patent foramen ovale
BAZ1B	Peripheral pulmonary artery stenosis
C14ORF179	Peripheral pulmonary artery stenosis
CLIP2	Peripheral pulmonary artery stenosis
ELN	Peripheral pulmonary artery stenosis
GTF2I	Peripheral pulmonary artery stenosis
GTF2IRD1	Peripheral pulmonary artery stenosis
IFT122	Peripheral pulmonary artery stenosis
IFT43	Peripheral pulmonary artery stenosis
JAG1	Peripheral pulmonary artery stenosis
LIMK1	Peripheral pulmonary artery stenosis
LOC100093631	Peripheral pulmonary artery stenosis
MGP	Peripheral pulmonary artery stenosis
NOTCH2	Peripheral pulmonary artery stenosis
PIGL	Peripheral pulmonary artery stenosis
RFC2	Peripheral pulmonary artery stenosis
TBL2	Peripheral pulmonary artery stenosis
WDR19	Peripheral pulmonary artery stenosis

WDR35	Peripheral pulmonary artery stenosis
WNT3	Peripheral pulmonary vessel aplasia
AMER1	Pierre-Robin sequence
ARVCF	Pierre-Robin sequence
COL11A1	Pierre-Robin sequence
COL11A2	Pierre-Robin sequence
COL2A1	Pierre-Robin sequence
COMT	Pierre-Robin sequence
EIF4A3	Pierre-Robin sequence
FAM123B	Pierre-Robin sequence
GP1BB	Pierre-Robin sequence
HIRA	Pierre-Robin sequence
PGM1	Pierre-Robin sequence
SOX9	Pierre-Robin sequence
TBX1	Pierre-Robin sequence
UFD1L	Pierre-Robin sequence
ARNT2	Pituitary hypothyroidism
LEPR	Pituitary hypothyroidism
CCBE1	Pleural effusion
CFTR	Pleural effusion
CPA1	Pleural effusion
CTRC	Pleural effusion
FAT4	Pleural effusion
HFE	Pleural effusion
LBR	Pleural effusion
PRSS1	Pleural effusion
PRSS2	Pleural effusion
SPINK1	Pleural effusion
TRY6	Pleural effusion
ATRIP	Pleuritis
MEFV	Pleuritis

TREX1	Pleuritis
DICER1	Pleuropulmonary blastoma
ACP5	Pneumonia
ADA	Pneumonia
ARMC4	Pneumonia
ATP11A	Pneumonia
BTK	Pneumonia
C14ORF104	Pneumonia
C19ORF51	Pneumonia
C21ORF59	Pneumonia
C2ORF39	Pneumonia
CARD11	Pneumonia
CASP8	Pneumonia
CCDC103	Pneumonia
CCDC114	Pneumonia
CCDC151	Pneumonia
CCDC39	Pneumonia
CCDC40	Pneumonia
CCDC65	Pneumonia
CCNO	Pneumonia
CD19	Pneumonia
CD81	Pneumonia
CDCA7	Pneumonia
CFB	Pneumonia
CHD7	Pneumonia
CR2	Pneumonia
DCLRE1C	Pneumonia
DNAAF1	Pneumonia
DNAAF2	Pneumonia
DNAAF3	Pneumonia
DNAAF5	Pneumonia

DNAH11	Pneumonia
DNAH5	Pneumonia
DNAI1	Pneumonia
DNAI2	Pneumonia
DNAL1	Pneumonia
DNMT3B	Pneumonia
DPP9	Pneumonia
DRC1	Pneumonia
DSP	Pneumonia
DYX1C1	Pneumonia
ELF4	Pneumonia
FAM13A	Pneumonia
FAM187A	Pneumonia
GAS8	Pneumonia
HDAC8	Pneumonia
HEATR2	Pneumonia
HELLS	Pneumonia
HYDIN	Pneumonia
ICOS	Pneumonia
IL21R	Pneumonia
IL2RG	Pneumonia
IL7R	Pneumonia
JAK3	Pneumonia
LIG4	Pneumonia
LRBA	Pneumonia
LRRC50	Pneumonia
LRRC6	Pneumonia
MS4A1	Pneumonia
MUC5B	Pneumonia
NFKB1	Pneumonia
NFKB2	Pneumonia

NIPBL	Pneumonia
NME8	Pneumonia
OBFC1	Pneumonia
OFD1	Pneumonia
PARN	Pneumonia
PNP	Pneumonia
PRKCD	Pneumonia
RAD21	Pneumonia
RAG1	Pneumonia
RAG2	Pneumonia
RANBP2	Pneumonia
RMRP	Pneumonia
RNF125	Pneumonia
RPGR	Pneumonia
RSPH1	Pneumonia
RSPH3	Pneumonia
RSPH4A	Pneumonia
RSPH9	Pneumonia
RTEL1	Pneumonia
SFTPA1	Pneumonia
SFTPA2	Pneumonia
SFTPC	Pneumonia
SGCG	Pneumonia
SLC35C1	Pneumonia
SMC1A	Pneumonia
SMC3	Pneumonia
SPAG1	Pneumonia
TERC	Pneumonia
TERT	Pneumonia
TNFRSF13B	Pneumonia
TNFRSF13C	Pneumonia

TNFRSF6B	Pneumonia
TNFSF12	Pneumonia
TNFSF12-TNFSF1	Pneumonia
TNFSF13	Pneumonia
TXNDC3	Pneumonia
WAS	Pneumonia
WIPF1	Pneumonia
ZAP70	Pneumonia
ZBTB24	Pneumonia
ZMYND10	Pneumonia
POMC	Pneumonia
PON1	Pneumonia
POR	Pneumonia
POSTN	Pneumonia
POT1	Pneumonia
POU5F1	Pneumonia
PPARG	Pneumonia
PPFIBP1	Pneumonia
PPP1R14A	Pneumonia
PPP2CA	Pneumonia
PPP2R1B	Pneumonia
PPP2R2A	Pneumonia
PPP2R2C	Pneumonia
PPP2R5A	Pneumonia
PRDX6	Pneumonia
PRF1	Pneumonia
PRKCE	Pneumonia
PRKDC	Pneumonia
PRKG1	Pneumonia
PROC	Pneumonia
PSEN1	Pneumonia

PSEN2	Pneumonia
PTEN	Pneumonia
PTENP1	Pneumonia
PTGDR	Pneumonia
PTGDR2	Pneumonia
PTGER2	Pneumonia
PTGER4	Pneumonia
PTGFR	Pneumonia
PTGIR	Pneumonia
PTGIS	Pneumonia
PTGS2	Pneumonia
PTH1R	Pneumonia
PTHLH	Pneumonia
PTMA	Pneumonia
PTMAP4	Pneumonia
PTPN1	Pneumonia
PTPN2	Pneumonia
PTX3	Pneumonia
PUF60	Pneumonia
PYCARD	Pneumonia
PYHIN1	Pneumonia
ACTA2	Pneumothorax
CHST14	Pneumothorax
DSE	Pneumothorax
FBN1	Pneumothorax
HRAS	Pneumothorax
MFAP5	Pneumothorax
MYH11	Pneumothorax
MYLK	Pneumothorax
PRKG1	Pneumothorax
SMAD3	Pneumothorax

TGFB2	Pneumothorax
TGFBR1	Pneumothorax
TGFBR2	Pneumothorax
CORIN	Preeclampsia
F5	Preeclampsia
PPARG	Preeclampsia
PPP1R3A	Preeclampsia
STOX1	Preeclampsia
CTNS	Primary hypothyroidism
SLC34A2	Progressive pulmonary function impairment
CCBE1	Protein-losing enteropathy
DGAT1	Protein-losing enteropathy
FAT4	Protein-losing enteropathy
MPI	Protein-losing enteropathy
BMPR2	Pulmonary arterial medial hypertrophy
LIFR	Pulmonary arterial medial hypertrophy
ACVRL1	Pulmonary arteriovenous malformation
ENG	Pulmonary arteriovenous malformation
GDF2	Pulmonary arteriovenous malformation
SMAD4	Pulmonary arteriovenous malformation
ACTA2	Pulmonary artery aneurysm
EFEMP2	Pulmonary artery aneurysm
FBLN5	Pulmonary artery aneurysm
FBN1	Pulmonary artery aneurysm
MFAP5	Pulmonary artery aneurysm
MYH11	Pulmonary artery aneurysm
MYLK	Pulmonary artery aneurysm
PRKG1	Pulmonary artery aneurysm
SMAD3	Pulmonary artery aneurysm
TGFB2	Pulmonary artery aneurysm
TGFBR1	Pulmonary artery aneurysm

TGFBR2	Pulmonary artery aneurysm
ABCB6	Pulmonary artery atresia
CERS1	Pulmonary artery atresia
CRELD1	Pulmonary artery atresia
FADD	Pulmonary artery atresia
GDF1	Pulmonary artery atresia
GDF3	Pulmonary artery atresia
GDF6	Pulmonary artery atresia
LASS1	Pulmonary artery atresia
NKX2-5	Pulmonary artery atresia
ODZ3	Pulmonary artery atresia
RARB	Pulmonary artery atresia
RBP4	Pulmonary artery atresia
SHH	Pulmonary artery atresia
STRA6	Pulmonary artery atresia
TENM3	Pulmonary artery atresia
VSX2	Pulmonary artery atresia
EFEMP2	Pulmonary artery dilatation
FBLN5	Pulmonary artery dilatation
FBN1	Pulmonary artery dilatation
ABCC9	Pulmonary artery hypoplasia
ACTC1	Pulmonary artery hypoplasia
ACTN2	Pulmonary artery hypoplasia
BAG3	Pulmonary artery hypoplasia
CRYAB	Pulmonary artery hypoplasia
CSRP3	Pulmonary artery hypoplasia
DES	Pulmonary artery hypoplasia
DMD	Pulmonary artery hypoplasia
DOLK	Pulmonary artery hypoplasia
DSG2	Pulmonary artery hypoplasia
DTNA	Pulmonary artery hypoplasia

FHL2	Pulmonary artery hypoplasia
FKTN	Pulmonary artery hypoplasia
GATAD1	Pulmonary artery hypoplasia
LAMA4	Pulmonary artery hypoplasia
LDB3	Pulmonary artery hypoplasia
LMNA	Pulmonary artery hypoplasia
MGP	Pulmonary artery hypoplasia
MIB1	Pulmonary artery hypoplasia
MYBPC3	Pulmonary artery hypoplasia
MYH6	Pulmonary artery hypoplasia
MYH7	Pulmonary artery hypoplasia
MYH7B	Pulmonary artery hypoplasia
MYPN	Pulmonary artery hypoplasia
NEXN	Pulmonary artery hypoplasia
PLN	Pulmonary artery hypoplasia
PRDM16	Pulmonary artery hypoplasia
PSEN1	Pulmonary artery hypoplasia
PSEN2	Pulmonary artery hypoplasia
RAF1	Pulmonary artery hypoplasia
RBM20	Pulmonary artery hypoplasia
SCN5A	Pulmonary artery hypoplasia
SDHA	Pulmonary artery hypoplasia
SGCD	Pulmonary artery hypoplasia
TAZ	Pulmonary artery hypoplasia
TCAP	Pulmonary artery hypoplasia
TMPO	Pulmonary artery hypoplasia
TNNC1	Pulmonary artery hypoplasia
TNNI3	Pulmonary artery hypoplasia
TNNT2	Pulmonary artery hypoplasia
TPM1	Pulmonary artery hypoplasia
TTN	Pulmonary artery hypoplasia

TXNRD2	Pulmonary artery hypoplasia
VCL	Pulmonary artery hypoplasia
ZEB2	Pulmonary artery sling
ARHGAP31	Pulmonary artery stenosis
C3ORF64	Pulmonary artery stenosis
DLL4	Pulmonary artery stenosis
DOCK6	Pulmonary artery stenosis
ELN	Pulmonary artery stenosis
EOGT	Pulmonary artery stenosis
GPC6	Pulmonary artery stenosis
LTBP4	Pulmonary artery stenosis
NAA10	Pulmonary artery stenosis
NKX2-5	Pulmonary artery stenosis
NOTCH1	Pulmonary artery stenosis
RBPJ	Pulmonary artery stenosis
SLC2A10	Pulmonary artery stenosis
ZEB2	Pulmonary artery stenosis
BMPR2	Pulmonary artery vasoconstriction
BMPR2	Pulmonary aterial intimal fibrosis
EIF2AK4	Pulmonary capillary hemangiomatosis
VHL	Pulmonary capillary hemangiomatosis
PRKAG2	Pulmonary edema
SFTPB	Pulmonary edema
SFTPC	Pulmonary edema
SERPINC1	Pulmonary edema
SERPIND1	Pulmonary edema
ACVRL1	Pulmonary embolism
AGGF1	Pulmonary embolism
AKAP9	Pulmonary embolism
AKT1	Pulmonary embolism
ANK2	Pulmonary embolism

ATP6	Pulmonary embolism
ATP8	Pulmonary embolism
C4A	Pulmonary embolism
CALM1	Pulmonary embolism
CALM2	Pulmonary embolism
CALM3	Pulmonary embolism
CAV3	Pulmonary embolism
CBS	Pulmonary embolism
COX1	Pulmonary embolism
COX2	Pulmonary embolism
COX3	Pulmonary embolism
CTLA4	Pulmonary embolism
ENG	Pulmonary embolism
F2	Pulmonary embolism
GDF2	Pulmonary embolism
GNAQ	Pulmonary embolism
HLA-B	Pulmonary embolism
HLA-C	Pulmonary embolism
HLA-DPB1	Pulmonary embolism
HLAB	Pulmonary embolism
IL10	Pulmonary embolism
IL12A	Pulmonary embolism
IL12RB2	Pulmonary embolism
IL23R	Pulmonary embolism
KCNE1	Pulmonary embolism
KCNE2	Pulmonary embolism
KCNH2	Pulmonary embolism
KCNJ5	Pulmonary embolism
KCNQ1	Pulmonary embolism
MEFV	Pulmonary embolism
MT-CO1	Pulmonary embolism

MT-CO3	Pulmonary embolism
MT-ND1	Pulmonary embolism
MT-ND5	Pulmonary embolism
MT-ND6	Pulmonary embolism
ND1	Pulmonary embolism
ND4	Pulmonary embolism
ND4L	Pulmonary embolism
ND5	Pulmonary embolism
ND6	Pulmonary embolism
NOD2	Pulmonary embolism
NOS1AP	Pulmonary embolism
PLP1	Pulmonary embolism
PROC	Pulmonary embolism
PROS1	Pulmonary embolism
PRTN3	Pulmonary embolism
PTEN	Pulmonary embolism
PTENP1	Pulmonary embolism
PTPN22	Pulmonary embolism
SCN4B	Pulmonary embolism
SCN5A	Pulmonary embolism
SERPINC1	Pulmonary embolism
SMAD4	Pulmonary embolism
SNTA1	Pulmonary embolism
STAT4	Pulmonary embolism
TLR4	Pulmonary embolism
TRNF	Pulmonary embolism
TRNH	Pulmonary embolism
TRNL1	Pulmonary embolism
TRNQ	Pulmonary embolism
TRNS1	Pulmonary embolism
TRNS2	Pulmonary embolism

TRNW	Pulmonary embolism
WAS	Pulmonary embolism
WIPF1	Pulmonary embolism
SFTPB	Pulmonary embolism
SFTPD	Pulmonary embolism
SIRT1	Pulmonary embolism
SKI	Pulmonary embolism
SKIL	Pulmonary embolism
SLC11A1	Pulmonary embolism
SLC11A2	Pulmonary embolism
TSHR	Pulmonary embolism
TSLP	Pulmonary embolism
TTR	Pulmonary embolism
TWIST1	Pulmonary embolism
TXN	Pulmonary embolism
SLC22A18	Pulmonary emphysema
SLC23A1	Pulmonary emphysema
SLC25A38	Pulmonary emphysema
SLC26A4	Pulmonary emphysema
SLC2A1	Pulmonary emphysema
SLC31A1	Pulmonary emphysema
SLC39A13	Pulmonary emphysema
ACD	Pulmonary fibrosis
ALMS1	Pulmonary fibrosis
AP3B1	Pulmonary fibrosis
ASAH1	Pulmonary fibrosis
ATP11A	Pulmonary fibrosis
BMP15	Pulmonary fibrosis
C16ORF57	Pulmonary fibrosis
C17ORF68	Pulmonary fibrosis
CAV1	Pulmonary fibrosis

CCNL2	Pulmonary fibrosis
CCR6	Pulmonary fibrosis
CFTR	Pulmonary fibrosis
CTC1	Pulmonary fibrosis
CTGF	Pulmonary fibrosis
CTLA4	Pulmonary fibrosis
DCTN4	Pulmonary fibrosis
DKC1	Pulmonary fibrosis
DPP9	Pulmonary fibrosis
DSP	Pulmonary fibrosis
FAM13A	Pulmonary fibrosis
FSHR	Pulmonary fibrosis
HLA-DPB1	Pulmonary fibrosis
HLA-DRB1	Pulmonary fibrosis
HLA-DRB4	Pulmonary fibrosis
HPS1	Pulmonary fibrosis
HPS4	Pulmonary fibrosis
IFNG	Pulmonary fibrosis
IRF5	Pulmonary fibrosis
KIAA0319L	Pulmonary fibrosis
MUC5B	Pulmonary fibrosis
NHP2	Pulmonary fibrosis
NOP10	Pulmonary fibrosis
NR5A1	Pulmonary fibrosis
OBFC1	Pulmonary fibrosis
PARN	Pulmonary fibrosis
PRF1	Pulmonary fibrosis
PRTN3	Pulmonary fibrosis
PSMC3IP	Pulmonary fibrosis
PTPN22	Pulmonary fibrosis
RNF168	Pulmonary fibrosis

RTEL1	Pulmonary fibrosis
SBDS	Pulmonary fibrosis
SBDSP1	Pulmonary fibrosis
SFTPA1	Pulmonary fibrosis
SFTPA2	Pulmonary fibrosis
SFTPC	Pulmonary fibrosis
STX1A	Pulmonary fibrosis
TERC	Pulmonary fibrosis
TERT	Pulmonary fibrosis
TGFB1	Pulmonary fibrosis
TINF2	Pulmonary fibrosis
TNFRSF6B	Pulmonary fibrosis
USB1	Pulmonary fibrosis
WRAP53	Pulmonary fibrosis
SLC4A1	Pulmonary fibrosis
SLC6A20	Pulmonary fibrosis
SLC6A4	Pulmonary fibrosis
SLCO1B3	Pulmonary fibrosis
SLEH1	Pulmonary fibrosis
SM1	Pulmonary fibrosis
SMAD2	Pulmonary fibrosis
SMAD3	Pulmonary fibrosis
SMAD7	Pulmonary fibrosis
SMAD9	Pulmonary fibrosis
SMARCC1	Pulmonary fibrosis
SOD1	Pulmonary fibrosis
SOD2	Pulmonary fibrosis
SOD3	Pulmonary fibrosis
SOX2	Pulmonary fibrosis
SP110	Pulmonary fibrosis
SPARC	Pulmonary fibrosis

SPP1	Pulmonary fibrosis
SPRR2B	Pulmonary fibrosis
SPRY2	Pulmonary fibrosis
SPTB	Pulmonary fibrosis
SS3	Pulmonary fibrosis
STAT3	Pulmonary fibrosis
STAT5A	Pulmonary fibrosis
STAT6	Pulmonary fibrosis
STK11	Pulmonary fibrosis
STRA6	Pulmonary fibrosis
ABCD4	Pulmonary hypertension
ACTA2	Pulmonary hypertension
ACVRL1	Pulmonary hypertension
ADAMTSL2	Pulmonary hypertension
AGPAT2	Pulmonary hypertension
ALMS1	Pulmonary hypertension
ARHGAP31	Pulmonary hypertension
ATP11A	Pulmonary hypertension
ATP5A1	Pulmonary hypertension
ATP6	Pulmonary hypertension
ATP8	Pulmonary hypertension
BANF1	Pulmonary hypertension
BMP1	Pulmonary hypertension
BMPR2	Pulmonary hypertension
BSCL2	Pulmonary hypertension
C3ORF64	Pulmonary hypertension
CACNA1D	Pulmonary hypertension
CAV1	Pulmonary hypertension
CCNL2	Pulmonary hypertension
CCR6	Pulmonary hypertension
CHST3	Pulmonary hypertension

CLCN7	Pulmonary hypertension
COL1A1	Pulmonary hypertension
COL1A2	Pulmonary hypertension
COX1	Pulmonary hypertension
COX2	Pulmonary hypertension
COX3	Pulmonary hypertension
COX7B	Pulmonary hypertension
CREB3L1	Pulmonary hypertension
CRTAP	Pulmonary hypertension
CTGF	Pulmonary hypertension
CYTSA	Pulmonary hypertension
DLL4	Pulmonary hypertension
DOCK6	Pulmonary hypertension
DPP9	Pulmonary hypertension
DSP	Pulmonary hypertension
EIF2AK4	Pulmonary hypertension
ENG	Pulmonary hypertension
EOGT	Pulmonary hypertension
FAM13A	Pulmonary hypertension
FBN1	Pulmonary hypertension
FGFR3	Pulmonary hypertension
FIG4	Pulmonary hypertension
FKBP10	Pulmonary hypertension
FLNA	Pulmonary hypertension
FOS	Pulmonary hypertension
FOXF1	Pulmonary hypertension
G6PC3	Pulmonary hypertension
GATA6	Pulmonary hypertension
GBA	Pulmonary hypertension
GDF2	Pulmonary hypertension
GJA1	Pulmonary hypertension

HBB	Pulmonary hypertension
HCCS	Pulmonary hypertension
HLA-B	Pulmonary hypertension
HLA-C	Pulmonary hypertension
HLA-DRB1	Pulmonary hypertension
HLA-DRB4	Pulmonary hypertension
HLAB	Pulmonary hypertension
HSPG2	Pulmonary hypertension
IDUA	Pulmonary hypertension
IKBKG	Pulmonary hypertension
IL12B	Pulmonary hypertension
IRF5	Pulmonary hypertension
JAK2	Pulmonary hypertension
KCNK3	Pulmonary hypertension
KIAA0319L	Pulmonary hypertension
KRT18	Pulmonary hypertension
KRT18P19	Pulmonary hypertension
KRT18P26	Pulmonary hypertension
KRT8	Pulmonary hypertension
KRT8P3	Pulmonary hypertension
KRT8P9	Pulmonary hypertension
LCRB	Pulmonary hypertension
LEPRE1	Pulmonary hypertension
LIFR	Pulmonary hypertension
LIPA	Pulmonary hypertension
LOC149501	Pulmonary hypertension
LOC645870	Pulmonary hypertension
LOC653348	Pulmonary hypertension
MGP	Pulmonary hypertension
MLX	Pulmonary hypertension
MPL	Pulmonary hypertension

MT-CO1	Pulmonary hypertension
MT-CO3	Pulmonary hypertension
MT-ND1	Pulmonary hypertension
MT-ND5	Pulmonary hypertension
MT-ND6	Pulmonary hypertension
MUC5B	Pulmonary hypertension
ND1	Pulmonary hypertension
ND4	Pulmonary hypertension
ND4L	Pulmonary hypertension
ND5	Pulmonary hypertension
ND6	Pulmonary hypertension
NDUFB11	Pulmonary hypertension
NFIX	Pulmonary hypertension
NFU1	Pulmonary hypertension
NOD2	Pulmonary hypertension
NOTCH1	Pulmonary hypertension
OBFC1	Pulmonary hypertension
P3H1	Pulmonary hypertension
PAM16	Pulmonary hypertension
PARN	Pulmonary hypertension
PDSS1	Pulmonary hypertension
PDSS1P1	Pulmonary hypertension
PPARG	Pulmonary hypertension
PPIB	Pulmonary hypertension
RBPJ	Pulmonary hypertension
RTEL1	Pulmonary hypertension
SARS2	Pulmonary hypertension
SCARB2	Pulmonary hypertension
SERPINF1	Pulmonary hypertension
SERPINH1	Pulmonary hypertension
SFTPA1	Pulmonary hypertension

SFTPA2	Pulmonary hypertension
SFTPB	Pulmonary hypertension
SFTPC	Pulmonary hypertension
SLC37A4	Pulmonary hypertension
SMAD4	Pulmonary hypertension
SMAD9	Pulmonary hypertension
SNX10	Pulmonary hypertension
SPECC1L	Pulmonary hypertension
TBX2	Pulmonary hypertension
TBX4	Pulmonary hypertension
TCIRG1	Pulmonary hypertension
TERC	Pulmonary hypertension
TERT	Pulmonary hypertension
THPO	Pulmonary hypertension
TIMM16	Pulmonary hypertension
TNFRSF6B	Pulmonary hypertension
TNFSF11	Pulmonary hypertension
TRNF	Pulmonary hypertension
TRNH	Pulmonary hypertension
TRNL1	Pulmonary hypertension
TRNQ	Pulmonary hypertension
TRNS1	Pulmonary hypertension
TRNS2	Pulmonary hypertension
TRNW	Pulmonary hypertension
WNT1	Pulmonary hypertension
FHIT	Pulmonary hypertension
FLCN	Pulmonary hypertension
FN1	Pulmonary hypertension
FOXA2	Pulmonary hypertension
FOXM1	Pulmonary hypertension
FSTL1	Pulmonary hypertension

G6PD	Pulmonary hypertension
GAA	Pulmonary hypertension
GAST	Pulmonary hypertension
GC	Pulmonary hypertension
GCLM	Pulmonary hypertension
GDF10	Pulmonary hypertension
GDF15	Pulmonary hypertension
GHRH	Pulmonary hypertension
GJB1	Pulmonary hypertension
GLRX5	Pulmonary hypertension
GNAQ	Pulmonary hypertension
GNAS	Pulmonary hypertension
GNAS-AS1	Pulmonary hypertension
AARS2	Pulmonary hypoplasia
ABCB6	Pulmonary hypoplasia
ACAD11	Pulmonary hypoplasia
ACE	Pulmonary hypoplasia
ADGRG6	Pulmonary hypoplasia
AGT	Pulmonary hypoplasia
AGTR1	Pulmonary hypoplasia
AHI1	Pulmonary hypoplasia
ALS2CR4	Pulmonary hypoplasia
ARL13B	Pulmonary hypoplasia
ATP5A1	Pulmonary hypoplasia
ATP5E	Pulmonary hypoplasia
ATP5EP2	Pulmonary hypoplasia
ATPAF1	Pulmonary hypoplasia
ATPAF2	Pulmonary hypoplasia
B9D1	Pulmonary hypoplasia
B9D2	Pulmonary hypoplasia
BCOR	Pulmonary hypoplasia

BMPER	Pulmonary hypoplasia
C2ORF86	Pulmonary hypoplasia
C5orf42	Pulmonary hypoplasia
C8ORF62	Pulmonary hypoplasia
CC2D2A	Pulmonary hypoplasia
CEP120	Pulmonary hypoplasia
CEP290	Pulmonary hypoplasia
CEP41	Pulmonary hypoplasia
CHRNA1	Pulmonary hypoplasia
CHRND	Pulmonary hypoplasia
CHRNG	Pulmonary hypoplasia
CSPP1	Pulmonary hypoplasia
CYTSA	Pulmonary hypoplasia
DHCR7	Pulmonary hypoplasia
DOK7	Pulmonary hypoplasia
DYNC2H1	Pulmonary hypoplasia
ETFA	Pulmonary hypoplasia
ETFB	Pulmonary hypoplasia
ETFDH	Pulmonary hypoplasia
FAM20C	Pulmonary hypoplasia
FAM38B	Pulmonary hypoplasia
FGF20	Pulmonary hypoplasia
FRAS1	Pulmonary hypoplasia
FREM2	Pulmonary hypoplasia
GDF3	Pulmonary hypoplasia
GDF6	Pulmonary hypoplasia
GLE1	Pulmonary hypoplasia
GPR126	Pulmonary hypoplasia
GRIP1	Pulmonary hypoplasia
HSPG2	Pulmonary hypoplasia
IFT140	Pulmonary hypoplasia

IFT172	Pulmonary hypoplasia
IFT80	Pulmonary hypoplasia
INPP5E	Pulmonary hypoplasia
INVS	Pulmonary hypoplasia
ITGA8	Pulmonary hypoplasia
KAT6B	Pulmonary hypoplasia
KIAA0586	Pulmonary hypoplasia
LBR	Pulmonary hypoplasia
LIFR	Pulmonary hypoplasia
LMNA	Pulmonary hypoplasia
LTBP4	Pulmonary hypoplasia
MKKS	Pulmonary hypoplasia
MKS1	Pulmonary hypoplasia
MUSK	Pulmonary hypoplasia
MYST4	Pulmonary hypoplasia
NAA10	Pulmonary hypoplasia
NEK1	Pulmonary hypoplasia
NEK8	Pulmonary hypoplasia
NPHP3	Pulmonary hypoplasia
NSDHL	Pulmonary hypoplasia
ODZ3	Pulmonary hypoplasia
PAX2	Pulmonary hypoplasia
PEX1	Pulmonary hypoplasia
PEX10	Pulmonary hypoplasia
PEX11B	Pulmonary hypoplasia
PEX12	Pulmonary hypoplasia
PEX13	Pulmonary hypoplasia
PEX14	Pulmonary hypoplasia
PEX16	Pulmonary hypoplasia
PEX19	Pulmonary hypoplasia
PEX2	Pulmonary hypoplasia

PEX26	Pulmonary hypoplasia
PEX3	Pulmonary hypoplasia
PEX5	Pulmonary hypoplasia
PEX6	Pulmonary hypoplasia
PHGDH	Pulmonary hypoplasia
PIEZO2	Pulmonary hypoplasia
PKHD1	Pulmonary hypoplasia
PSAT1	Pulmonary hypoplasia
RAPSN	Pulmonary hypoplasia
RARB	Pulmonary hypoplasia
RBP4	Pulmonary hypoplasia
REN	Pulmonary hypoplasia
RET	Pulmonary hypoplasia
RPGRIP1	Pulmonary hypoplasia
RPGRIP1L	Pulmonary hypoplasia
RYR1	Pulmonary hypoplasia
SHH	Pulmonary hypoplasia
SLC26A2	Pulmonary hypoplasia
SPECC1L	Pulmonary hypoplasia
STRA6	Pulmonary hypoplasia
TCTN1	Pulmonary hypoplasia
TCTN2	Pulmonary hypoplasia
TENM3	Pulmonary hypoplasia
TMEM216	Pulmonary hypoplasia
TMEM231	Pulmonary hypoplasia
TMEM237	Pulmonary hypoplasia
TMEM67	Pulmonary hypoplasia
TSGA14	Pulmonary hypoplasia
TTC21B	Pulmonary hypoplasia
VSX2	Pulmonary hypoplasia
WDPCP	Pulmonary hypoplasia

WDR19	Pulmonary hypoplasia
WDR34	Pulmonary hypoplasia
WDR35	Pulmonary hypoplasia
WDR60	Pulmonary hypoplasia
WNT3	Pulmonary hypoplasia
ZMPSTE24	Pulmonary hypoplasia
APOE	Pulmonary infiltrates
BIRC3	Pulmonary infiltrates
C4A	Pulmonary infiltrates
CAV1	Pulmonary infiltrates
CCNL2	Pulmonary infiltrates
CCR6	Pulmonary infiltrates
CTGF	Pulmonary infiltrates
CTLA4	Pulmonary infiltrates
FOXP1	Pulmonary infiltrates
GBA	Pulmonary infiltrates
HLA-B	Pulmonary infiltrates
HLA-C	Pulmonary infiltrates
HLA-DPB1	Pulmonary infiltrates
HLA-DRB1	Pulmonary infiltrates
HLA-DRB4	Pulmonary infiltrates
HLAB	Pulmonary infiltrates
IL10	Pulmonary infiltrates
IL12A	Pulmonary infiltrates
IL12RB2	Pulmonary infiltrates
IL23R	Pulmonary infiltrates
IRF5	Pulmonary infiltrates
LOC100129500	Pulmonary infiltrates
LPIN2	Pulmonary infiltrates
MALT1	Pulmonary infiltrates
MEFV	Pulmonary infiltrates

MYD88	Pulmonary infiltrates
NOD2	Pulmonary infiltrates
PDGFRA	Pulmonary infiltrates
PRTN3	Pulmonary infiltrates
PTPN22	Pulmonary infiltrates
RAB27A	Pulmonary infiltrates
SCARB2	Pulmonary infiltrates
STAT4	Pulmonary infiltrates
TLR4	Pulmonary infiltrates
TSC1	Pulmonary infiltrates
TSC2	Pulmonary infiltrates
CEP120	Pulmonary insufficiency
COL1A1	Pulmonary insufficiency
COL1A2	Pulmonary insufficiency
CRTAP	Pulmonary insufficiency
DYNC2H1	Pulmonary insufficiency
EFEMP2	Pulmonary insufficiency
FBLN5	Pulmonary insufficiency
FOXF1	Pulmonary insufficiency
IFT140	Pulmonary insufficiency
IFT172	Pulmonary insufficiency
IFT80	Pulmonary insufficiency
INVS	Pulmonary insufficiency
LEPRE1	Pulmonary insufficiency
P3H1	Pulmonary insufficiency
PPIB	Pulmonary insufficiency
TTC21B	Pulmonary insufficiency
WDR19	Pulmonary insufficiency
WDR34	Pulmonary insufficiency
WDR60	Pulmonary insufficiency
CCBE1	Pulmonary lymphangiectasia

FAT4	Pulmonary lymphangiectasia
TSC1	Pulmonary lymphangiomyomatosis
TSC2	Pulmonary lymphangiomyomatosis
BMPR2	Pulmonary venoocclusive disease
EIF2AK4	Pulmonary venoocclusive disease
A2ML1	Pulmonic stenosis
ABCB6	Pulmonic stenosis
ADAMTS10	Pulmonic stenosis
ADK	Pulmonic stenosis
ANKS6	Pulmonic stenosis
ARHGAP31	Pulmonic stenosis
B3GALNT2	Pulmonic stenosis
B3GALTL	Pulmonic stenosis
B3GLCT	Pulmonic stenosis
B3GNT1	Pulmonic stenosis
B3GNT2	Pulmonic stenosis
B4GAT1	Pulmonic stenosis
BAZ1B	Pulmonic stenosis
BCOR	Pulmonic stenosis
BRAF	Pulmonic stenosis
C3ORF39	Pulmonic stenosis
C3ORF64	Pulmonic stenosis
CCDC22	Pulmonic stenosis
CERS1	Pulmonic stenosis
CHD7	Pulmonic stenosis
CHST3	Pulmonic stenosis
CLIP2	Pulmonic stenosis
CNTNAP2	Pulmonic stenosis
COL4A1	Pulmonic stenosis
CYP24A1	Pulmonic stenosis
DAG1	Pulmonic stenosis

DLL4	Pulmonic stenosis
DOCK6	Pulmonic stenosis
ELN	Pulmonic stenosis
EOGT	Pulmonic stenosis
FBN1	Pulmonic stenosis
FKRP	Pulmonic stenosis
FKTN	Pulmonic stenosis
G6PC3	Pulmonic stenosis
GATA4	Pulmonic stenosis
GATA6	Pulmonic stenosis
GDF1	Pulmonic stenosis
GDF3	Pulmonic stenosis
GDF6	Pulmonic stenosis
GMPPB	Pulmonic stenosis
GPC3	Pulmonic stenosis
GPC4	Pulmonic stenosis
GTF2I	Pulmonic stenosis
GTF2IRD1	Pulmonic stenosis
HRAS	Pulmonic stenosis
IGFBP7	Pulmonic stenosis
ISPD	Pulmonic stenosis
KANSL1	Pulmonic stenosis
KAT6B	Pulmonic stenosis
KDM6A	Pulmonic stenosis
KIAA0196	Pulmonic stenosis
KIAA1267	Pulmonic stenosis
KMT2D	Pulmonic stenosis
KRAS	Pulmonic stenosis
LARGE	Pulmonic stenosis
LASS1	Pulmonic stenosis
LIMK1	Pulmonic stenosis

LOC100093631	Pulmonic stenosis
LOC344593	Pulmonic stenosis
LOC407835	Pulmonic stenosis
LOC442113	Pulmonic stenosis
LTBP2	Pulmonic stenosis
LZTR1	Pulmonic stenosis
MAP2K1	Pulmonic stenosis
MAP2K2	Pulmonic stenosis
MEGF8	Pulmonic stenosis
MGP	Pulmonic stenosis
MLL2	Pulmonic stenosis
MYST4	Pulmonic stenosis
NAA10	Pulmonic stenosis
NF1	Pulmonic stenosis
NOTCH1	Pulmonic stenosis
NRAS	Pulmonic stenosis
NRXN1	Pulmonic stenosis
ODZ3	Pulmonic stenosis
POMGNT1	Pulmonic stenosis
POMGNT2	Pulmonic stenosis
POMK	Pulmonic stenosis
POMT1	Pulmonic stenosis
POMT2	Pulmonic stenosis
PTPN11	Pulmonic stenosis
RAB23	Pulmonic stenosis
RAF1	Pulmonic stenosis
RARB	Pulmonic stenosis
RASA2	Pulmonic stenosis
RBP4	Pulmonic stenosis
RBPJ	Pulmonic stenosis
RFC2	Pulmonic stenosis

RIT1	Pulmonic stenosis
SEMA3E	Pulmonic stenosis
SGK196	Pulmonic stenosis
SGOL1	Pulmonic stenosis
SHH	Pulmonic stenosis
SHOC2	Pulmonic stenosis
SKIV2L	Pulmonic stenosis
SMAD3	Pulmonic stenosis
SOS1	Pulmonic stenosis
SOS2	Pulmonic stenosis
STRA6	Pulmonic stenosis
TBL2	Pulmonic stenosis
TENM3	Pulmonic stenosis
TMEM5	Pulmonic stenosis
TTC37	Pulmonic stenosis
VSX2	Pulmonic stenosis
ZEB2	Pulmonic stenosis
ZIC3	Pulmonic stenosis
CYBA	Recurrent Aspergillus infections
CYBB	Recurrent Aspergillus infections
NCF1	Recurrent Aspergillus infections
NCF1C	Recurrent Aspergillus infections
NCF2	Recurrent Aspergillus infections
NCF4	Recurrent Aspergillus infections
GBA	Recurrent aspiration pneumonia
HIC1	Recurrent aspiration pneumonia
KDM6A	Recurrent aspiration pneumonia
KMT2D	Recurrent aspiration pneumonia
LOC440917	Recurrent aspiration pneumonia
MLL2	Recurrent aspiration pneumonia
NFIX	Recurrent aspiration pneumonia

PAFAH1B1	Recurrent aspiration pneumonia
YWHAE	Recurrent aspiration pneumonia
ADAM17	Recurrent bronchiolitis
EGFR	Recurrent bronchiolitis
ARMC4	Recurrent bronchitis
ATM	Recurrent bronchitis
B2M	Recurrent bronchitis
BLNK	Recurrent bronchitis
C14ORF104	Recurrent bronchitis
C19ORF51	Recurrent bronchitis
C1QA	Recurrent bronchitis
C1QB	Recurrent bronchitis
C1QC	Recurrent bronchitis
C1R	Recurrent bronchitis
C1S	Recurrent bronchitis
C2	Recurrent bronchitis
C21ORF59	Recurrent bronchitis
C2ORF39	Recurrent bronchitis
C4A	Recurrent bronchitis
C4B	Recurrent bronchitis
CCDC103	Recurrent bronchitis
CCDC114	Recurrent bronchitis
CCDC151	Recurrent bronchitis
CCDC39	Recurrent bronchitis
CCDC40	Recurrent bronchitis
CCDC65	Recurrent bronchitis
CCNO	Recurrent bronchitis
CD19	Recurrent bronchitis
CD79A	Recurrent bronchitis
CD79B	Recurrent bronchitis
CD81	Recurrent bronchitis

CR2	Recurrent bronchitis
DNAAF1	Recurrent bronchitis
DNAAF2	Recurrent bronchitis
DNAAF3	Recurrent bronchitis
DNAAF5	Recurrent bronchitis
DNAH11	Recurrent bronchitis
DNAH5	Recurrent bronchitis
DNAI1	Recurrent bronchitis
DNAI2	Recurrent bronchitis
DNAL1	Recurrent bronchitis
DRC1	Recurrent bronchitis
DYX1C1	Recurrent bronchitis
FAM187A	Recurrent bronchitis
GAS8	Recurrent bronchitis
GNPTAB	Recurrent bronchitis
HEATR2	Recurrent bronchitis
HYDIN	Recurrent bronchitis
ICOS	Recurrent bronchitis
IGH@	Recurrent bronchitis
IGHG1	Recurrent bronchitis
IGHG3	Recurrent bronchitis
IGHM	Recurrent bronchitis
IGHV3-11	Recurrent bronchitis
IGHV3-7	Recurrent bronchitis
IGHV4-31	Recurrent bronchitis
IGLL1	Recurrent bronchitis
IL2RG	Recurrent bronchitis
LOC651610	Recurrent bronchitis
LRBA	Recurrent bronchitis
LRRC50	Recurrent bronchitis
LRRC6	Recurrent bronchitis

LRRC8A	Recurrent bronchitis
MGP	Recurrent bronchitis
MS4A1	Recurrent bronchitis
NBN	Recurrent bronchitis
NFKB1	Recurrent bronchitis
NFKB2	Recurrent bronchitis
NME8	Recurrent bronchitis
OFD1	Recurrent bronchitis
PIK3R1	Recurrent bronchitis
PRKCD	Recurrent bronchitis
RPGR	Recurrent bronchitis
RSPH1	Recurrent bronchitis
RSPH3	Recurrent bronchitis
RSPH4A	Recurrent bronchitis
RSPH9	Recurrent bronchitis
SERPING1	Recurrent bronchitis
SPAG1	Recurrent bronchitis
TAP1	Recurrent bronchitis
TAP2	Recurrent bronchitis
TAPBP	Recurrent bronchitis
TCF3	Recurrent bronchitis
TNFRSF13B	Recurrent bronchitis
TNFRSF13C	Recurrent bronchitis
TNFSF12	Recurrent bronchitis
TNFSF12-TNFSF1	Recurrent bronchitis
TNFSF13	Recurrent bronchitis
TXNDC3	Recurrent bronchitis
ZMYND10	Recurrent bronchitis
CFTR	Recurrent bronchopulmonary infections
DCTN4	Recurrent bronchopulmonary infections
LAMTOR2	Recurrent bronchopulmonary infections

ROBLD3	Recurrent bronchopulmonary infections
STX1A	Recurrent bronchopulmonary infections
TGFB1	Recurrent bronchopulmonary infections
CYBA	Recurrent Burkholderia cepacia infections
CYBB	Recurrent Burkholderia cepacia infections
NCF1	Recurrent Burkholderia cepacia infections
NCF1C	Recurrent Burkholderia cepacia infections
NCF2	Recurrent Burkholderia cepacia infections
NCF4	Recurrent Burkholderia cepacia infections
CFI	Recurrent Haemophilus influenzae infections
RPGR	Recurrent Haemophilus influenzae infections
IKBKAP	Recurrent infections due to aspiration
CIITA	Recurrent lower respiratory tract infections
COL12A1	Recurrent lower respiratory tract infections
COL6A1	Recurrent lower respiratory tract infections
COL6A2	Recurrent lower respiratory tract infections
COL6A3	Recurrent lower respiratory tract infections
FCN3	Recurrent lower respiratory tract infections
LOC731751	Recurrent lower respiratory tract infections
PNP	Recurrent lower respiratory tract infections
PRKDC	Recurrent lower respiratory tract infections
RFX5	Recurrent lower respiratory tract infections
RFXANK	Recurrent lower respiratory tract infections
RFXAP	Recurrent lower respiratory tract infections
WAS	Recurrent lower respiratory tract infections
WIPF1	Recurrent lower respiratory tract infections
CYBB	Recurrent mycobacterial infections
DOCK8	Recurrent mycobacterial infections
IFNGR1	Recurrent mycobacterial infections
IFNGR2	Recurrent mycobacterial infections
IL12RB1	Recurrent mycobacterial infections

ISG15	Recurrent mycobacterial infections
STAT1	Recurrent mycobacterial infections
TYK2	Recurrent mycobacterial infections
GATA2	Recurrent mycobacterium avium complex infections
IKBKG	Recurrent mycobacterium avium complex infections
ADAM17	Recurrent pneumonia
ALMS1	Recurrent pneumonia
ARMC4	Recurrent pneumonia
BLNK	Recurrent pneumonia
C14ORF104	Recurrent pneumonia
C14ORF179	Recurrent pneumonia
C16ORF57	Recurrent pneumonia
C19ORF51	Recurrent pneumonia
C21ORF59	Recurrent pneumonia
C2ORF39	Recurrent pneumonia
CCDC103	Recurrent pneumonia
CCDC114	Recurrent pneumonia
CCDC151	Recurrent pneumonia
CCDC39	Recurrent pneumonia
CCDC40	Recurrent pneumonia
CCDC65	Recurrent pneumonia
CCNO	Recurrent pneumonia
CD19	Recurrent pneumonia
CD79A	Recurrent pneumonia
CD79B	Recurrent pneumonia
CD81	Recurrent pneumonia
CDC6	Recurrent pneumonia
CDT1	Recurrent pneumonia
CFTR	Recurrent pneumonia
COL11A2	Recurrent pneumonia
COL2A1	Recurrent pneumonia

CR2	Recurrent pneumonia
CYBA	Recurrent pneumonia
CYBB	Recurrent pneumonia
DCTN4	Recurrent pneumonia
DDR2	Recurrent pneumonia
DNAAF1	Recurrent pneumonia
DNAAF2	Recurrent pneumonia
DNAAF3	Recurrent pneumonia
DNAAF5	Recurrent pneumonia
DNAH11	Recurrent pneumonia
DNAH5	Recurrent pneumonia
DNAI1	Recurrent pneumonia
DNAI2	Recurrent pneumonia
DNAL1	Recurrent pneumonia
DRC1	Recurrent pneumonia
DYX1C1	Recurrent pneumonia
EGFR	Recurrent pneumonia
FAM187A	Recurrent pneumonia
FMO3	Recurrent pneumonia
GAS8	Recurrent pneumonia
GNPTAB	Recurrent pneumonia
HEATR2	Recurrent pneumonia
HYDIN	Recurrent pneumonia
ICOS	Recurrent pneumonia
IFT122	Recurrent pneumonia
IFT43	Recurrent pneumonia
IGH@	Recurrent pneumonia
IGHG1	Recurrent pneumonia
IGHG3	Recurrent pneumonia
IGHM	Recurrent pneumonia
IGHV3-11	Recurrent pneumonia

IGHV3-7	Recurrent pneumonia
IGHV4-31	Recurrent pneumonia
IGLL1	Recurrent pneumonia
LEP	Recurrent pneumonia
LRBA	Recurrent pneumonia
LRRC50	Recurrent pneumonia
LRRC6	Recurrent pneumonia
LRRC8A	Recurrent pneumonia
MASP2	Recurrent pneumonia
MS4A1	Recurrent pneumonia
NBN	Recurrent pneumonia
NCF1	Recurrent pneumonia
NCF1C	Recurrent pneumonia
NCF2	Recurrent pneumonia
NCF4	Recurrent pneumonia
NFKB1	Recurrent pneumonia
NFKB2	Recurrent pneumonia
NME8	Recurrent pneumonia
OFD1	Recurrent pneumonia
ORC1	Recurrent pneumonia
ORC1L	Recurrent pneumonia
ORC4	Recurrent pneumonia
ORC4L	Recurrent pneumonia
ORC6	Recurrent pneumonia
ORC6L	Recurrent pneumonia
PEPD	Recurrent pneumonia
PIK3R1	Recurrent pneumonia
PLOD1	Recurrent pneumonia
PRKCD	Recurrent pneumonia
RNU4ATAC	Recurrent pneumonia
RPGR	Recurrent pneumonia

RSPH1	Recurrent pneumonia
RSPH3	Recurrent pneumonia
RSPH4A	Recurrent pneumonia
RSPH9	Recurrent pneumonia
SPAG1	Recurrent pneumonia
STX1A	Recurrent pneumonia
TCF3	Recurrent pneumonia
TGFB1	Recurrent pneumonia
TNFRSF11A	Recurrent pneumonia
TNFRSF13B	Recurrent pneumonia
TNFRSF13C	Recurrent pneumonia
TNFSF12	Recurrent pneumonia
TNFSF12-TNFSF1	Recurrent pneumonia
TNFSF13	Recurrent pneumonia
TXNDC3	Recurrent pneumonia
USB1	Recurrent pneumonia
WDR19	Recurrent pneumonia
WDR35	Recurrent pneumonia
ZMYND10	Recurrent pneumonia
CYBA	Recurrent Serratia marcescens infections
CYBB	Recurrent Serratia marcescens infections
NCF1	Recurrent Serratia marcescens infections
NCF1C	Recurrent Serratia marcescens infections
NCF2	Recurrent Serratia marcescens infections
NCF4	Recurrent Serratia marcescens infections
AMT	Recurrent singultus
GCSH	Recurrent singultus
GFAP	Recurrent singultus
GLDC	Recurrent singultus
LOC654085	Recurrent singultus
LOC729080	Recurrent singultus

LOC730107	Recurrent singultus
ARMC4	Recurrent sinopulmonary infections
C14ORF104	Recurrent sinopulmonary infections
C19ORF51	Recurrent sinopulmonary infections
C21ORF59	Recurrent sinopulmonary infections
C2ORF39	Recurrent sinopulmonary infections
CASP8	Recurrent sinopulmonary infections
CCDC103	Recurrent sinopulmonary infections
CCDC114	Recurrent sinopulmonary infections
CCDC151	Recurrent sinopulmonary infections
CCDC39	Recurrent sinopulmonary infections
CCDC40	Recurrent sinopulmonary infections
CCDC65	Recurrent sinopulmonary infections
CCNO	Recurrent sinopulmonary infections
CD19	Recurrent sinopulmonary infections
CD81	Recurrent sinopulmonary infections
CR2	Recurrent sinopulmonary infections
DNAAF1	Recurrent sinopulmonary infections
DNAAF2	Recurrent sinopulmonary infections
DNAAF3	Recurrent sinopulmonary infections
DNAAF5	Recurrent sinopulmonary infections
DNAH11	Recurrent sinopulmonary infections
DNAH5	Recurrent sinopulmonary infections
DNAI1	Recurrent sinopulmonary infections
DNAI2	Recurrent sinopulmonary infections
DNAL1	Recurrent sinopulmonary infections
DOCK8	Recurrent sinopulmonary infections
DRC1	Recurrent sinopulmonary infections
DYX1C1	Recurrent sinopulmonary infections
FAM187A	Recurrent sinopulmonary infections
GAS8	Recurrent sinopulmonary infections

HEATR2	Recurrent sinopulmonary infections
HYDIN	Recurrent sinopulmonary infections
ICOS	Recurrent sinopulmonary infections
LRBA	Recurrent sinopulmonary infections
LRRC50	Recurrent sinopulmonary infections
LRRC6	Recurrent sinopulmonary infections
MS4A1	Recurrent sinopulmonary infections
NFKB1	Recurrent sinopulmonary infections
NFKB2	Recurrent sinopulmonary infections
NME8	Recurrent sinopulmonary infections
OFD1	Recurrent sinopulmonary infections
PIK3CD	Recurrent sinopulmonary infections
PIK3R1	Recurrent sinopulmonary infections
PRKCD	Recurrent sinopulmonary infections
RPGR	Recurrent sinopulmonary infections
RSPH1	Recurrent sinopulmonary infections
RSPH3	Recurrent sinopulmonary infections
RSPH4A	Recurrent sinopulmonary infections
RSPH9	Recurrent sinopulmonary infections
SPAG1	Recurrent sinopulmonary infections
STAT3	Recurrent sinopulmonary infections
TNFRSF13B	Recurrent sinopulmonary infections
TNFRSF13C	Recurrent sinopulmonary infections
TNFSF12	Recurrent sinopulmonary infections
TNFSF12-TNFSF1	Recurrent sinopulmonary infections
TNFSF13	Recurrent sinopulmonary infections
TXNDC3	Recurrent sinopulmonary infections
ZMYND10	Recurrent sinopulmonary infections
CFI	Recurrent streptococcus pneumoniae infections
IKBKG	Recurrent streptococcus pneumoniae infections
IRAK4	Recurrent streptococcus pneumoniae infections

AICDA	Recurrent upper and lower respiratory tract infections
UNG	Recurrent upper and lower respiratory tract infections
ARSB	Recurrent upper respiratory tract infections
CIITA	Recurrent upper respiratory tract infections
CREBBP	Recurrent upper respiratory tract infections
CTLA4	Recurrent upper respiratory tract infections
CXCR4	Recurrent upper respiratory tract infections
DCLRE1C	Recurrent upper respiratory tract infections
EXT1	Recurrent upper respiratory tract infections
GALNS	Recurrent upper respiratory tract infections
GLB1	Recurrent upper respiratory tract infections
GNS	Recurrent upper respiratory tract infections
HGSNAT	Recurrent upper respiratory tract infections
JAK3	Recurrent upper respiratory tract infections
LEP	Recurrent upper respiratory tract infections
NAGLU	Recurrent upper respiratory tract infections
OFD1	Recurrent upper respiratory tract infections
PLG	Recurrent upper respiratory tract infections
PNP	Recurrent upper respiratory tract infections
PRPS1	Recurrent upper respiratory tract infections
PRPS1L1	Recurrent upper respiratory tract infections
RFX5	Recurrent upper respiratory tract infections
RFXANK	Recurrent upper respiratory tract infections
RFXAP	Recurrent upper respiratory tract infections
SFTPC	Recurrent upper respiratory tract infections
SGSH	Recurrent upper respiratory tract infections
TRPS1	Recurrent upper respiratory tract infections
WAS	Recurrent upper respiratory tract infections
WIPF1	Recurrent upper respiratory tract infections
ACTA1	Reduced vital capacity
MYH7	Reduced vital capacity

SEPN1	Reduced vital capacity
TPM2	Reduced vital capacity
TPM3	Reduced vital capacity
TTN	Reduced vital capacity
DECR1	Respiratory acidosis
ITGA3	Respiratory acidosis
ASL	Respiratory alkalosis
ASS1	Respiratory alkalosis
CA5A	Respiratory alkalosis
CPS1	Respiratory alkalosis
OTC	Respiratory alkalosis
CPT2	Respiratory arrest
APOPT1	Respiratory difficulties
ARX	Respiratory difficulties
ATP6	Respiratory difficulties
ATP8	Respiratory difficulties
C12ORF62	Respiratory difficulties
C14ORF153	Respiratory difficulties
C1ORF31	Respiratory difficulties
C2ORF64	Respiratory difficulties
CASK	Respiratory difficulties
CCDC56	Respiratory difficulties
CDKL5	Respiratory difficulties
COA5	Respiratory difficulties
COA6	Respiratory difficulties
COX1	Respiratory difficulties
COX10	Respiratory difficulties
COX14	Respiratory difficulties
COX15	Respiratory difficulties
COX2	Respiratory difficulties
COX20	Respiratory difficulties

COX3	Respiratory difficulties
COX6B1	Respiratory difficulties
CREBBP	Respiratory difficulties
DNM1	Respiratory difficulties
EDA	Respiratory difficulties
EDA2R	Respiratory difficulties
EFTUD2	Respiratory difficulties
FAM36A	Respiratory difficulties
FASTKD2	Respiratory difficulties
GATA1	Respiratory difficulties
GNAO1	Respiratory difficulties
GRIN2B	Respiratory difficulties
HCN1	Respiratory difficulties
KCNB1	Respiratory difficulties
KCNQ2	Respiratory difficulties
LOC100131801	Respiratory difficulties
MT-CO1	Respiratory difficulties
MT-CO3	Respiratory difficulties
NECAP1	Respiratory difficulties
PET100	Respiratory difficulties
PIGA	Respiratory difficulties
PIGQ	Respiratory difficulties
PLCB1	Respiratory difficulties
PLXND1	Respiratory difficulties
PNKP	Respiratory difficulties
REV3L	Respiratory difficulties
RPL11	Respiratory difficulties
RPL15	Respiratory difficulties
RPL15P17	Respiratory difficulties
RPL15P18	Respiratory difficulties
RPL15P22	Respiratory difficulties

RPL15P3	Respiratory difficulties
RPL15P7	Respiratory difficulties
RPL26	Respiratory difficulties
RPL26P16	Respiratory difficulties
RPL26P19	Respiratory difficulties
RPL26P33	Respiratory difficulties
RPL26P6	Respiratory difficulties
RPL35A	Respiratory difficulties
RPL5	Respiratory difficulties
RPL5P1	Respiratory difficulties
RPL5P34	Respiratory difficulties
RPS10	Respiratory difficulties
RPS10P11	Respiratory difficulties
RPS10P13	Respiratory difficulties
RPS10P22	Respiratory difficulties
RPS10P4	Respiratory difficulties
RPS10P7	Respiratory difficulties
RPS17	Respiratory difficulties
RPS17L	Respiratory difficulties
RPS19	Respiratory difficulties
RPS19P3	Respiratory difficulties
RPS24	Respiratory difficulties
RPS26	Respiratory difficulties
RPS26P2	Respiratory difficulties
RPS26P20	Respiratory difficulties
RPS26P25	Respiratory difficulties
RPS26P31	Respiratory difficulties
RPS26P35	Respiratory difficulties
RPS26P38	Respiratory difficulties
RPS26P39	Respiratory difficulties
RPS26P50	Respiratory difficulties

RPS26P53	Respiratory difficulties
RPS26P54	Respiratory difficulties
RPS26P6	Respiratory difficulties
RPS26P8	Respiratory difficulties
RPS28	Respiratory difficulties
RPS28P6	Respiratory difficulties
RPS28P9	Respiratory difficulties
RPS29	Respiratory difficulties
RPS29P11	Respiratory difficulties
RPS29P16	Respiratory difficulties
RPS29P17	Respiratory difficulties
RPS29P3	Respiratory difficulties
RPS29P9	Respiratory difficulties
RPS7	Respiratory difficulties
RPS7P10	Respiratory difficulties
RPS7P11	Respiratory difficulties
RPS7P4	Respiratory difficulties
SCN2A	Respiratory difficulties
SCN8A	Respiratory difficulties
SCO1	Respiratory difficulties
SCO2	Respiratory difficulties
SIK1	Respiratory difficulties
SLC13A5	Respiratory difficulties
SLC25A22	Respiratory difficulties
SPTAN1	Respiratory difficulties
ST3GAL3	Respiratory difficulties
STAT5B	Respiratory difficulties
STT3B	Respiratory difficulties
STXBP1	Respiratory difficulties
TACO1	Respiratory difficulties
TSR2	Respiratory difficulties

WWOX	Respiratory difficulties
ABCA3	Respiratory distress
ACTA1	Respiratory distress
ADAMTS13	Respiratory distress
AGRN	Respiratory distress
ALDH7A1	Respiratory distress
ARL6	Respiratory distress
ARMC4	Respiratory distress
BBIP1	Respiratory distress
BBS1	Respiratory distress
BBS10	Respiratory distress
BBS12	Respiratory distress
BBS2	Respiratory distress
BBS4	Respiratory distress
BBS5	Respiratory distress
BBS7	Respiratory distress
BBS9	Respiratory distress
BMPER	Respiratory distress
BRP44L	Respiratory distress
C14ORF104	Respiratory distress
C19ORF51	Respiratory distress
C21ORF59	Respiratory distress
C2ORF39	Respiratory distress
C2ORF86	Respiratory distress
CCDC103	Respiratory distress
CCDC114	Respiratory distress
CCDC151	Respiratory distress
CCDC39	Respiratory distress
CCDC40	Respiratory distress
CCDC65	Respiratory distress
CCNO	Respiratory distress

CDC6	Respiratory distress
CDT1	Respiratory distress
CEP290	Respiratory distress
CHAT	Respiratory distress
CHRNA1	Respiratory distress
CHRNB1	Respiratory distress
CHRND	Respiratory distress
CHRNE	Respiratory distress
COL2A1	Respiratory distress
CPT2	Respiratory distress
DMPK	Respiratory distress
DNAAF1	Respiratory distress
DNAAF2	Respiratory distress
DNAAF3	Respiratory distress
DNAAF5	Respiratory distress
DNAH11	Respiratory distress
DNAH5	Respiratory distress
DNAI1	Respiratory distress
DNAI2	Respiratory distress
DNAL1	Respiratory distress
DOK7	Respiratory distress
DPM1	Respiratory distress
DRC1	Respiratory distress
DYX1C1	Respiratory distress
ETFA	Respiratory distress
ETFB	Respiratory distress
ETFDH	Respiratory distress
FAM187A	Respiratory distress
FGFR1	Respiratory distress
FGFR2	Respiratory distress
GAS8	Respiratory distress

GBA	Respiratory distress
HACD1	Respiratory distress
HEATR2	Respiratory distress
HYDIN	Respiratory distress
IFT172	Respiratory distress
IFT27	Respiratory distress
IL1RN	Respiratory distress
ITGA3	Respiratory distress
ITGA7	Respiratory distress
KAT6A	Respiratory distress
LRP4	Respiratory distress
LRRC50	Respiratory distress
LRRC6	Respiratory distress
LYRM4	Respiratory distress
LZTFL1	Respiratory distress
MKKS	Respiratory distress
MKS1	Respiratory distress
MMAA	Respiratory distress
MMAB	Respiratory distress
MPC1	Respiratory distress
MUSK	Respiratory distress
MYL2	Respiratory distress
MYST3	Respiratory distress
NAGS	Respiratory distress
NCRNA00081	Respiratory distress
NFS1	Respiratory distress
NKX2-1	Respiratory distress
NME8	Respiratory distress
NPHP1	Respiratory distress
OFD1	Respiratory distress
ORC1	Respiratory distress

ORC1L	Respiratory distress
ORC4	Respiratory distress
ORC4L	Respiratory distress
ORC6	Respiratory distress
ORC6L	Respiratory distress
OTX2	Respiratory distress
PRRX1	Respiratory distress
PTPLA	Respiratory distress
RABL4	Respiratory distress
RAPSN	Respiratory distress
RPGR	Respiratory distress
RSPH1	Respiratory distress
RSPH3	Respiratory distress
RSPH4A	Respiratory distress
RSPH9	Respiratory distress
SCN4A	Respiratory distress
SDCCAG8	Respiratory distress
SEPN1	Respiratory distress
SFTPB	Respiratory distress
SFTPC	Respiratory distress
SLC25A1	Respiratory distress
SNAP25	Respiratory distress
SOX9	Respiratory distress
SPAG1	Respiratory distress
SYT2	Respiratory distress
TPM2	Respiratory distress
TPM3	Respiratory distress
TRIM32	Respiratory distress
TTC8	Respiratory distress
TXNDC3	Respiratory distress
WDPCP	Respiratory distress

ZMYND10	Respiratory distress
TERT	Respiratory distress
TF	Respiratory distress
TFF1	Respiratory distress
TFF2	Respiratory distress
TFPI	Respiratory distress
TFRC	Respiratory distress
TG	Respiratory distress
ABCA3	Respiratory failure
ACVR1	Respiratory failure
ATP6	Respiratory failure
ATP8	Respiratory failure
B3GALNT2	Respiratory failure
B3GNT1	Respiratory failure
B3GNT2	Respiratory failure
B4GAT1	Respiratory failure
BCS1L	Respiratory failure
BOLA3	Respiratory failure
C11ORF83	Respiratory failure
C20ORF7	Respiratory failure
C3ORF39	Respiratory failure
C6ORF125	Respiratory failure
C8ORF38	Respiratory failure
COL4A1	Respiratory failure
COX10	Respiratory failure
COX15	Respiratory failure
COX3	Respiratory failure
CPT2	Respiratory failure
CRYAB	Respiratory failure
CTSD	Respiratory failure
CYC1	Respiratory failure

CYTB	Respiratory failure
DAG1	Respiratory failure
DMD	Respiratory failure
ECHS1	Respiratory failure
ERBB3	Respiratory failure
EXOSC3	Respiratory failure
EXOSC8	Respiratory failure
FANCB	Respiratory failure
FKRP	Respiratory failure
FKTN	Respiratory failure
FLNA	Respiratory failure
FOXRED1	Respiratory failure
HADHA	Respiratory failure
HADHB	Respiratory failure
HRAS	Respiratory failure
IGHMBP2	Respiratory failure
INVS	Respiratory failure
ISPD	Respiratory failure
KBTBD5	Respiratory failure
KLHL40	Respiratory failure
LARGE	Respiratory failure
LIPT1	Respiratory failure
LOC100131149	Respiratory failure
LOC100131801	Respiratory failure
LOC442454	Respiratory failure
LOC644754	Respiratory failure
LOC727947	Respiratory failure
LTBP4	Respiratory failure
LYRM7	Respiratory failure
MCM4	Respiratory failure
MEGF10	Respiratory failure

MT-CYB	Respiratory failure
MT-ND1	Respiratory failure
MT-ND2	Respiratory failure
MT-ND5	Respiratory failure
MT-ND6	Respiratory failure
MTFMT	Respiratory failure
NAIP	Respiratory failure
ND1	Respiratory failure
ND2	Respiratory failure
ND3	Respiratory failure
ND4	Respiratory failure
ND4L	Respiratory failure
ND5	Respiratory failure
ND6	Respiratory failure
NDUFA1	Respiratory failure
NDUFA10	Respiratory failure
NDUFA11	Respiratory failure
NDUFA12	Respiratory failure
NDUFA2	Respiratory failure
NDUFA4	Respiratory failure
NDUFA9	Respiratory failure
NDUFAF1	Respiratory failure
NDUFAF2	Respiratory failure
NDUFAF3	Respiratory failure
NDUFAF4	Respiratory failure
NDUFAF5	Respiratory failure
NDUFAF6	Respiratory failure
NDUFB3	Respiratory failure
NDUFB9	Respiratory failure
NDUFS1	Respiratory failure
NDUFS2	Respiratory failure

NDUFS3	Respiratory failure
NDUFS4	Respiratory failure
NDUFS6	Respiratory failure
NDUFS7	Respiratory failure
NDUFS8	Respiratory failure
NDUFV1	Respiratory failure
NDUFV2	Respiratory failure
NFU1	Respiratory failure
NPC2	Respiratory failure
NUBPL	Respiratory failure
PDHA1	Respiratory failure
PET100	Respiratory failure
POMGNT1	Respiratory failure
POMGNT2	Respiratory failure
POMK	Respiratory failure
POMT1	Respiratory failure
POMT2	Respiratory failure
PSAP	Respiratory failure
PTEN	Respiratory failure
PTENP1	Respiratory failure
RARS2	Respiratory failure
SARS2	Respiratory failure
SCO2	Respiratory failure
SDHA	Respiratory failure
SFTPB	Respiratory failure
SFTPC	Respiratory failure
SGK196	Respiratory failure
SLC19A3	Respiratory failure
SMAD4	Respiratory failure
SMN1	Respiratory failure
SMN2	Respiratory failure

SUCLG1	Respiratory failure
SURF1	Respiratory failure
TACO1	Respiratory failure
TMEM5	Respiratory failure
TRNK	Respiratory failure
TRNL1	Respiratory failure
TRNV	Respiratory failure
TRNW	Respiratory failure
TRPV4	Respiratory failure
TSEN54	Respiratory failure
TSFM	Respiratory failure
TTC19	Respiratory failure
UQCC2	Respiratory failure
UQCC3	Respiratory failure
UQCRB	Respiratory failure
UQCRC2	Respiratory failure
UQCRQ	Respiratory failure
VRK1	Respiratory failure
MAMLD1	Respiratory failure requiring assisted ventilation
MTM1	Respiratory failure requiring assisted ventilation
AAAS	Respiratory insufficiency
ABCA12	Respiratory insufficiency
ABCB6	Respiratory insufficiency
ACE	Respiratory insufficiency
ACOX1	Respiratory insufficiency
ACTA1	Respiratory insufficiency
ACVR1	Respiratory insufficiency
ADAMTSL2	Respiratory insufficiency
ADCY6	Respiratory insufficiency
AGGF1	Respiratory insufficiency
AGK	Respiratory insufficiency

AGRN	Respiratory insufficiency
AGT	Respiratory insufficiency
AGTR1	Respiratory insufficiency
AHI1	Respiratory insufficiency
ALAS2	Respiratory insufficiency
ALMS1	Respiratory insufficiency
ALS2CR4	Respiratory insufficiency
ARL13B	Respiratory insufficiency
ARMC4	Respiratory insufficiency
ASAH1	Respiratory insufficiency
ASCL1	Respiratory insufficiency
ASNS	Respiratory insufficiency
ATP6	Respiratory insufficiency
ATP8	Respiratory insufficiency
ATRX	Respiratory insufficiency
B3GALNT2	Respiratory insufficiency
B3GNT1	Respiratory insufficiency
B3GNT2	Respiratory insufficiency
B4GAT1	Respiratory insufficiency
B9D1	Respiratory insufficiency
BAG3	Respiratory insufficiency
BCS1L	Respiratory insufficiency
BDNF	Respiratory insufficiency
BIN1	Respiratory insufficiency
BTD	Respiratory insufficiency
C14ORF104	Respiratory insufficiency
C19ORF51	Respiratory insufficiency
C20ORF54	Respiratory insufficiency
C20ORF7	Respiratory insufficiency
C21ORF59	Respiratory insufficiency
C2ORF39	Respiratory insufficiency

C3ORF39	Respiratory insufficiency
C5orf42	Respiratory insufficiency
C6ORF59	Respiratory insufficiency
C8ORF38	Respiratory insufficiency
CAV1	Respiratory insufficiency
CCBE1	Respiratory insufficiency
CCDC103	Respiratory insufficiency
CCDC114	Respiratory insufficiency
CCDC151	Respiratory insufficiency
CCDC39	Respiratory insufficiency
CCDC40	Respiratory insufficiency
CCDC65	Respiratory insufficiency
CCNL2	Respiratory insufficiency
CCNO	Respiratory insufficiency
CCR6	Respiratory insufficiency
CDC6	Respiratory insufficiency
CDKL5	Respiratory insufficiency
CDT1	Respiratory insufficiency
CEP120	Respiratory insufficiency
CEP41	Respiratory insufficiency
CHAT	Respiratory insufficiency
CHD7	Respiratory insufficiency
CHRNA1	Respiratory insufficiency
CHRNB1	Respiratory insufficiency
CHRND	Respiratory insufficiency
CHRNE	Respiratory insufficiency
CHRNG	Respiratory insufficiency
CISD2	Respiratory insufficiency
CLCF1	Respiratory insufficiency
CNTNAP1	Respiratory insufficiency
COL11A1	Respiratory insufficiency

COL11A2	Respiratory insufficiency
COL12A1	Respiratory insufficiency
COL1A1	Respiratory insufficiency
COL1A2	Respiratory insufficiency
COL2A1	Respiratory insufficiency
COL3A1	Respiratory insufficiency
COL4A1	Respiratory insufficiency
COL5A1	Respiratory insufficiency
COL6A1	Respiratory insufficiency
COL6A2	Respiratory insufficiency
COL6A3	Respiratory insufficiency
COLQ	Respiratory insufficiency
COQ4	Respiratory insufficiency
COX1	Respiratory insufficiency
COX10	Respiratory insufficiency
COX15	Respiratory insufficiency
COX2	Respiratory insufficiency
COX3	Respiratory insufficiency
COX7B	Respiratory insufficiency
CPS1	Respiratory insufficiency
CRLF1	Respiratory insufficiency
CRTAP	Respiratory insufficiency
CSPP1	Respiratory insufficiency
CTGF	Respiratory insufficiency
CTLA4	Respiratory insufficiency
CTSD	Respiratory insufficiency
CYP27A1	Respiratory insufficiency
DAG1	Respiratory insufficiency
DCTN1	Respiratory insufficiency
DLL3	Respiratory insufficiency
DMPK	Respiratory insufficiency

DNAAF1	Respiratory insufficiency
DNAAF2	Respiratory insufficiency
DNAAF3	Respiratory insufficiency
DNAAF5	Respiratory insufficiency
DNAH11	Respiratory insufficiency
DNAH5	Respiratory insufficiency
DNAI1	Respiratory insufficiency
DNAI2	Respiratory insufficiency
DNAL1	Respiratory insufficiency
DNASE1L3	Respiratory insufficiency
DNM2	Respiratory insufficiency
DOK7	Respiratory insufficiency
DPAGT1	Respiratory insufficiency
DRC1	Respiratory insufficiency
DST	Respiratory insufficiency
DYNC2H1	Respiratory insufficiency
DYX1C1	Respiratory insufficiency
ECHS1	Respiratory insufficiency
EDN3	Respiratory insufficiency
EFEMP2	Respiratory insufficiency
EHMT1	Respiratory insufficiency
EIF4A3	Respiratory insufficiency
EMD	Respiratory insufficiency
EPOR	Respiratory insufficiency
ERF	Respiratory insufficiency
EXOSC3	Respiratory insufficiency
EXOSC8	Respiratory insufficiency
FAM111A	Respiratory insufficiency
FAM187A	Respiratory insufficiency
FAM20C	Respiratory insufficiency
FAT4	Respiratory insufficiency

FBLN5	Respiratory insufficiency
FBN1	Respiratory insufficiency
FGFR1	Respiratory insufficiency
FGFR2	Respiratory insufficiency
FGFR3	Respiratory insufficiency
FHL1	Respiratory insufficiency
FKBP10	Respiratory insufficiency
FKRP	Respiratory insufficiency
FKTN	Respiratory insufficiency
FLNA	Respiratory insufficiency
FLNB	Respiratory insufficiency
FLNC	Respiratory insufficiency
FOXF1	Respiratory insufficiency
FOXG1	Respiratory insufficiency
FOXI1	Respiratory insufficiency
FOXRED1	Respiratory insufficiency
G6PC3	Respiratory insufficiency
GAS8	Respiratory insufficiency
GATA2	Respiratory insufficiency
GBA	Respiratory insufficiency
GDF3	Respiratory insufficiency
GDF6	Respiratory insufficiency
GDNF	Respiratory insufficiency
GFAP	Respiratory insufficiency
GLA	Respiratory insufficiency
GLUL	Respiratory insufficiency
GMPPA	Respiratory insufficiency
GMPPB	Respiratory insufficiency
GPR172A	Respiratory insufficiency
HACD1	Respiratory insufficiency
HBB	Respiratory insufficiency

HCCS	Respiratory insufficiency
HEATR2	Respiratory insufficiency
HES7	Respiratory insufficiency
HLA-B	Respiratory insufficiency
HLA-C	Respiratory insufficiency
HLA-DPB1	Respiratory insufficiency
HLA-DRB1	Respiratory insufficiency
HLA-DRB4	Respiratory insufficiency
HLAB	Respiratory insufficiency
HLCS	Respiratory insufficiency
HSPG2	Respiratory insufficiency
HYDIN	Respiratory insufficiency
IDH1	Respiratory insufficiency
IDH2	Respiratory insufficiency
IFT140	Respiratory insufficiency
IFT172	Respiratory insufficiency
IFT80	Respiratory insufficiency
IGHV4-34	Respiratory insufficiency
IKZF1	Respiratory insufficiency
INPP5E	Respiratory insufficiency
INPPL1	Respiratory insufficiency
IRF5	Respiratory insufficiency
ISPD	Respiratory insufficiency
ITGA7	Respiratory insufficiency
JAK2	Respiratory insufficiency
KBTBD10	Respiratory insufficiency
KCNJ10	Respiratory insufficiency
KIAA0586	Respiratory insufficiency
KIT	Respiratory insufficiency
KLHL41	Respiratory insufficiency
KRT14	Respiratory insufficiency

KRT16	Respiratory insufficiency
KRT16P2	Respiratory insufficiency
KRT17	Respiratory insufficiency
KRT17P3	Respiratory insufficiency
KRT5	Respiratory insufficiency
KRT6A	Respiratory insufficiency
KRT6B	Respiratory insufficiency
LAMA3	Respiratory insufficiency
LAMB2	Respiratory insufficiency
LARGE	Respiratory insufficiency
LBR	Respiratory insufficiency
LCRB	Respiratory insufficiency
LEPRE1	Respiratory insufficiency
LFNG	Respiratory insufficiency
LIAS	Respiratory insufficiency
LIFR	Respiratory insufficiency
LIG4	Respiratory insufficiency
LIPT1	Respiratory insufficiency
LMNA	Respiratory insufficiency
LOC100131801	Respiratory insufficiency
LOC652799	Respiratory insufficiency
LOC653348	Respiratory insufficiency
LOC653882	Respiratory insufficiency
LRP4	Respiratory insufficiency
LRP5	Respiratory insufficiency
LRRC50	Respiratory insufficiency
LRRC6	Respiratory insufficiency
MAMLD1	Respiratory insufficiency
MCCC1	Respiratory insufficiency
MCCC2	Respiratory insufficiency
MECP2	Respiratory insufficiency

MESP2	Respiratory insufficiency
MKS1	Respiratory insufficiency
MPL	Respiratory insufficiency
MT-CO1	Respiratory insufficiency
MT-CO3	Respiratory insufficiency
MT-ND1	Respiratory insufficiency
MT-ND2	Respiratory insufficiency
MT-ND5	Respiratory insufficiency
MT-ND6	Respiratory insufficiency
MTFMT	Respiratory insufficiency
MTM1	Respiratory insufficiency
MTRR	Respiratory insufficiency
MUSK	Respiratory insufficiency
MUT	Respiratory insufficiency
MYBPC1	Respiratory insufficiency
MYD88	Respiratory insufficiency
MYH7	Respiratory insufficiency
MYL2	Respiratory insufficiency
NBN	Respiratory insufficiency
ND1	Respiratory insufficiency
ND2	Respiratory insufficiency
ND3	Respiratory insufficiency
ND4	Respiratory insufficiency
ND4L	Respiratory insufficiency
ND5	Respiratory insufficiency
ND6	Respiratory insufficiency
NDUFA10	Respiratory insufficiency
NDUFA12	Respiratory insufficiency
NDUFA2	Respiratory insufficiency
NDUFA4	Respiratory insufficiency
NDUFA9	Respiratory insufficiency

NDUFAF2	Respiratory insufficiency
NDUFAF5	Respiratory insufficiency
NDUFAF6	Respiratory insufficiency
NDUFB11	Respiratory insufficiency
NDUFS1	Respiratory insufficiency
NDUFS2	Respiratory insufficiency
NDUFS3	Respiratory insufficiency
NDUFS4	Respiratory insufficiency
NDUFS7	Respiratory insufficiency
NDUFS8	Respiratory insufficiency
NDUFV1	Respiratory insufficiency
NDUFV2	Respiratory insufficiency
NFIX	Respiratory insufficiency
NME8	Respiratory insufficiency
NOD2	Respiratory insufficiency
NSUN2	Respiratory insufficiency
NTNG1	Respiratory insufficiency
OCRL	Respiratory insufficiency
ODZ3	Respiratory insufficiency
OFD1	Respiratory insufficiency
ORAI1	Respiratory insufficiency
ORC1	Respiratory insufficiency
ORC1L	Respiratory insufficiency
ORC4	Respiratory insufficiency
ORC4L	Respiratory insufficiency
ORC6	Respiratory insufficiency
ORC6L	Respiratory insufficiency
OTX2	Respiratory insufficiency
P3H1	Respiratory insufficiency
PDHA1	Respiratory insufficiency
PET100	Respiratory insufficiency

PEX1	Respiratory insufficiency
PEX10	Respiratory insufficiency
PEX11B	Respiratory insufficiency
PEX12	Respiratory insufficiency
PEX13	Respiratory insufficiency
PEX14	Respiratory insufficiency
PEX16	Respiratory insufficiency
PEX19	Respiratory insufficiency
PEX2	Respiratory insufficiency
PEX26	Respiratory insufficiency
PEX3	Respiratory insufficiency
PEX5	Respiratory insufficiency
PEX6	Respiratory insufficiency
PEX7	Respiratory insufficiency
PHOX2B	Respiratory insufficiency
PHYH	Respiratory insufficiency
PIP5K1C	Respiratory insufficiency
PKHD1	Respiratory insufficiency
PLOD1	Respiratory insufficiency
PLOD2	Respiratory insufficiency
PMP22	Respiratory insufficiency
POLR1C	Respiratory insufficiency
POLR1D	Respiratory insufficiency
POMGNT1	Respiratory insufficiency
POMGNT2	Respiratory insufficiency
POMK	Respiratory insufficiency
POMT1	Respiratory insufficiency
POMT2	Respiratory insufficiency
PPIB	Respiratory insufficiency
PRKCSH	Respiratory insufficiency
PRPS1	Respiratory insufficiency

PRPS1L1	Respiratory insufficiency
PRRX1	Respiratory insufficiency
PRTN3	Respiratory insufficiency
PSAP	Respiratory insufficiency
PSMB8	Respiratory insufficiency
PTPLA	Respiratory insufficiency
PTPN22	Respiratory insufficiency
PURA	Respiratory insufficiency
RAPSN	Respiratory insufficiency
RARB	Respiratory insufficiency
RARS2	Respiratory insufficiency
RBP4	Respiratory insufficiency
REN	Respiratory insufficiency
RET	Respiratory insufficiency
RFT1	Respiratory insufficiency
RIPPLY2	Respiratory insufficiency
RMRP	Respiratory insufficiency
RNASEH1	Respiratory insufficiency
RNU4ATAC	Respiratory insufficiency
RPGR	Respiratory insufficiency
RRM2B	Respiratory insufficiency
RSPH1	Respiratory insufficiency
RSPH3	Respiratory insufficiency
RSPH4A	Respiratory insufficiency
RSPH9	Respiratory insufficiency
RYR1	Respiratory insufficiency
SCN4A	Respiratory insufficiency
SCO2	Respiratory insufficiency
SDHA	Respiratory insufficiency
SEC63	Respiratory insufficiency
SEMA3E	Respiratory insufficiency

SEPN1	Respiratory insufficiency
SEPT9	Respiratory insufficiency
SF3B4	Respiratory insufficiency
SGK196	Respiratory insufficiency
SH2B3	Respiratory insufficiency
SHH	Respiratory insufficiency
SLC19A3	Respiratory insufficiency
SLC25A1	Respiratory insufficiency
SLC25A3	Respiratory insufficiency
SLC25A4	Respiratory insufficiency
SLC26A2	Respiratory insufficiency
SLC26A4	Respiratory insufficiency
SLC2A10	Respiratory insufficiency
SLC52A2	Respiratory insufficiency
SLC52A3	Respiratory insufficiency
SLC7A7	Respiratory insufficiency
SMAD4	Respiratory insufficiency
SNAP25	Respiratory insufficiency
SOX9	Respiratory insufficiency
SPAG1	Respiratory insufficiency
SPEG	Respiratory insufficiency
STIM1	Respiratory insufficiency
STRA6	Respiratory insufficiency
SURF1	Respiratory insufficiency
SYNE1	Respiratory insufficiency
SYNE2	Respiratory insufficiency
SYT2	Respiratory insufficiency
TACO1	Respiratory insufficiency
TBX2	Respiratory insufficiency
TBX4	Respiratory insufficiency
TCOF1	Respiratory insufficiency

TCTN1	Respiratory insufficiency
TCTN2	Respiratory insufficiency
TENM3	Respiratory insufficiency
TET2	Respiratory insufficiency
TMEM231	Respiratory insufficiency
TMEM237	Respiratory insufficiency
TMEM43	Respiratory insufficiency
TMEM5	Respiratory insufficiency
TMEM67	Respiratory insufficiency
TMEM70	Respiratory insufficiency
TNNT1	Respiratory insufficiency
TPM2	Respiratory insufficiency
TPM3	Respiratory insufficiency
TRNF	Respiratory insufficiency
TRNH	Respiratory insufficiency
TRNK	Respiratory insufficiency
TRNL1	Respiratory insufficiency
TRNQ	Respiratory insufficiency
TRNS1	Respiratory insufficiency
TRNS2	Respiratory insufficiency
TRNV	Respiratory insufficiency
TRNW	Respiratory insufficiency
TSC1	Respiratory insufficiency
TSC2	Respiratory insufficiency
TSEN54	Respiratory insufficiency
TSGA14	Respiratory insufficiency
TTC21B	Respiratory insufficiency
TTN	Respiratory insufficiency
TXNDC3	Respiratory insufficiency
UBE3B	Respiratory insufficiency
VRK1	Respiratory insufficiency

VSX2	Respiratory insufficiency
WAS	Respiratory insufficiency
WDR19	Respiratory insufficiency
WDR34	Respiratory insufficiency
WDR35	Respiratory insufficiency
WDR60	Respiratory insufficiency
WFS1	Respiratory insufficiency
WIPF1	Respiratory insufficiency
ZMPSTE24	Respiratory insufficiency
ZMYND10	Respiratory insufficiency
SYK	Respiratory insufficiency
TBKBP1	Respiratory insufficiency
TBX21	Respiratory insufficiency
TBXA2R	Respiratory insufficiency
TCN1	Respiratory insufficiency
TCN2	Respiratory insufficiency
TEP1	Respiratory insufficiency
TERC	Respiratory insufficiency
TERT	Respiratory insufficiency
TGFA	Respiratory insufficiency
TGFB1	Respiratory insufficiency
ARMC4	Respiratory insufficiency due to defective ciliary clearance
C14ORF104	Respiratory insufficiency due to defective ciliary clearance
C19ORF51	Respiratory insufficiency due to defective ciliary clearance
C21ORF59	Respiratory insufficiency due to defective ciliary clearance
C2ORF39	Respiratory insufficiency due to defective ciliary clearance
CCDC103	Respiratory insufficiency due to defective ciliary clearance
CCDC114	Respiratory insufficiency due to defective ciliary clearance
CCDC151	Respiratory insufficiency due to defective ciliary clearance
CCDC39	Respiratory insufficiency due to defective ciliary clearance
CCDC40	Respiratory insufficiency due to defective ciliary clearance

CCDC65	Respiratory insufficiency due to defective ciliary clearance
CCNO	Respiratory insufficiency due to defective ciliary clearance
DNAAF1	Respiratory insufficiency due to defective ciliary clearance
DNAAF2	Respiratory insufficiency due to defective ciliary clearance
DNAAF3	Respiratory insufficiency due to defective ciliary clearance
DNAAF5	Respiratory insufficiency due to defective ciliary clearance
DNAH11	Respiratory insufficiency due to defective ciliary clearance
DNAH5	Respiratory insufficiency due to defective ciliary clearance
DNAI1	Respiratory insufficiency due to defective ciliary clearance
DNAI2	Respiratory insufficiency due to defective ciliary clearance
DNAL1	Respiratory insufficiency due to defective ciliary clearance
DRC1	Respiratory insufficiency due to defective ciliary clearance
DYX1C1	Respiratory insufficiency due to defective ciliary clearance
FAM187A	Respiratory insufficiency due to defective ciliary clearance
GAS8	Respiratory insufficiency due to defective ciliary clearance
HEATR2	Respiratory insufficiency due to defective ciliary clearance
HYDIN	Respiratory insufficiency due to defective ciliary clearance
LRRC50	Respiratory insufficiency due to defective ciliary clearance
LRRC6	Respiratory insufficiency due to defective ciliary clearance
NME8	Respiratory insufficiency due to defective ciliary clearance
OFD1	Respiratory insufficiency due to defective ciliary clearance
RPGR	Respiratory insufficiency due to defective ciliary clearance
RSPH1	Respiratory insufficiency due to defective ciliary clearance
RSPH3	Respiratory insufficiency due to defective ciliary clearance
RSPH4A	Respiratory insufficiency due to defective ciliary clearance
RSPH9	Respiratory insufficiency due to defective ciliary clearance
SPAG1	Respiratory insufficiency due to defective ciliary clearance
TXNDC3	Respiratory insufficiency due to defective ciliary clearance
ZMYND10	Respiratory insufficiency due to defective ciliary clearance
ACTA1	Respiratory insufficiency due to muscle weakness
AGRN	Respiratory insufficiency due to muscle weakness

AIFM1	Respiratory insufficiency due to muscle weakness
ALS2	Respiratory insufficiency due to muscle weakness
ANG	Respiratory insufficiency due to muscle weakness
APOPT1	Respiratory insufficiency due to muscle weakness
ASAH1	Respiratory insufficiency due to muscle weakness
ATP6	Respiratory insufficiency due to muscle weakness
ATP8	Respiratory insufficiency due to muscle weakness
ATXN2	Respiratory insufficiency due to muscle weakness
B3GALNT2	Respiratory insufficiency due to muscle weakness
B3GNT1	Respiratory insufficiency due to muscle weakness
B3GNT2	Respiratory insufficiency due to muscle weakness
B4GAT1	Respiratory insufficiency due to muscle weakness
BIN1	Respiratory insufficiency due to muscle weakness
C12ORF62	Respiratory insufficiency due to muscle weakness
C14ORF153	Respiratory insufficiency due to muscle weakness
C3ORF39	Respiratory insufficiency due to muscle weakness
C9orf72	Respiratory insufficiency due to muscle weakness
CCDC56	Respiratory insufficiency due to muscle weakness
CFL2	Respiratory insufficiency due to muscle weakness
CHAT	Respiratory insufficiency due to muscle weakness
CHCHD10	Respiratory insufficiency due to muscle weakness
CHMP2B	Respiratory insufficiency due to muscle weakness
CHRNA1	Respiratory insufficiency due to muscle weakness
CHRNB1	Respiratory insufficiency due to muscle weakness
CHRND	Respiratory insufficiency due to muscle weakness
CHRNE	Respiratory insufficiency due to muscle weakness
CNTN1	Respiratory insufficiency due to muscle weakness
COL12A1	Respiratory insufficiency due to muscle weakness
COL4A1	Respiratory insufficiency due to muscle weakness
COL6A1	Respiratory insufficiency due to muscle weakness
COL6A2	Respiratory insufficiency due to muscle weakness

COL6A3	Respiratory insufficiency due to muscle weakness
COLQ	Respiratory insufficiency due to muscle weakness
COX1	Respiratory insufficiency due to muscle weakness
COX10	Respiratory insufficiency due to muscle weakness
COX14	Respiratory insufficiency due to muscle weakness
COX2	Respiratory insufficiency due to muscle weakness
COX20	Respiratory insufficiency due to muscle weakness
COX3	Respiratory insufficiency due to muscle weakness
COX6B1	Respiratory insufficiency due to muscle weakness
CRYAB	Respiratory insufficiency due to muscle weakness
DAG1	Respiratory insufficiency due to muscle weakness
DAO	Respiratory insufficiency due to muscle weakness
DCTN1	Respiratory insufficiency due to muscle weakness
DES	Respiratory insufficiency due to muscle weakness
DOK7	Respiratory insufficiency due to muscle weakness
ERBB4	Respiratory insufficiency due to muscle weakness
FAM36A	Respiratory insufficiency due to muscle weakness
FASTKD2	Respiratory insufficiency due to muscle weakness
FHL1	Respiratory insufficiency due to muscle weakness
FIG4	Respiratory insufficiency due to muscle weakness
FKRP	Respiratory insufficiency due to muscle weakness
FKTN	Respiratory insufficiency due to muscle weakness
FUS	Respiratory insufficiency due to muscle weakness
GAA	Respiratory insufficiency due to muscle weakness
HACD1	Respiratory insufficiency due to muscle weakness
HNRNPA1	Respiratory insufficiency due to muscle weakness
HNRNPA1P2	Respiratory insufficiency due to muscle weakness
HNRPA1L-2	Respiratory insufficiency due to muscle weakness
HNRPA1L3	Respiratory insufficiency due to muscle weakness
ISPD	Respiratory insufficiency due to muscle weakness
ITGA7	Respiratory insufficiency due to muscle weakness

LAMA2	Respiratory insufficiency due to muscle weakness
LAMB2	Respiratory insufficiency due to muscle weakness
LARGE	Respiratory insufficiency due to muscle weakness
LMNA	Respiratory insufficiency due to muscle weakness
LMOD3	Respiratory insufficiency due to muscle weakness
LOC100131801	Respiratory insufficiency due to muscle weakness
LOC644037	Respiratory insufficiency due to muscle weakness
LOC645691	Respiratory insufficiency due to muscle weakness
LOC728643	Respiratory insufficiency due to muscle weakness
LRP4	Respiratory insufficiency due to muscle weakness
MATR3	Respiratory insufficiency due to muscle weakness
MT-CO1	Respiratory insufficiency due to muscle weakness
MT-CO3	Respiratory insufficiency due to muscle weakness
MUSK	Respiratory insufficiency due to muscle weakness
MYL2	Respiratory insufficiency due to muscle weakness
NEB	Respiratory insufficiency due to muscle weakness
NEFH	Respiratory insufficiency due to muscle weakness
OPTN	Respiratory insufficiency due to muscle weakness
ORAI1	Respiratory insufficiency due to muscle weakness
PET100	Respiratory insufficiency due to muscle weakness
PFN1	Respiratory insufficiency due to muscle weakness
PLEKHG5	Respiratory insufficiency due to muscle weakness
POLG	Respiratory insufficiency due to muscle weakness
POMGNT1	Respiratory insufficiency due to muscle weakness
POMGNT2	Respiratory insufficiency due to muscle weakness
POMK	Respiratory insufficiency due to muscle weakness
POMT1	Respiratory insufficiency due to muscle weakness
POMT2	Respiratory insufficiency due to muscle weakness
PON1	Respiratory insufficiency due to muscle weakness
PON2	Respiratory insufficiency due to muscle weakness
PON3	Respiratory insufficiency due to muscle weakness

PPARGC1A	Respiratory insufficiency due to muscle weakness
PRPH	Respiratory insufficiency due to muscle weakness
PTPLA	Respiratory insufficiency due to muscle weakness
PUS1	Respiratory insufficiency due to muscle weakness
RAPSN	Respiratory insufficiency due to muscle weakness
SCN4A	Respiratory insufficiency due to muscle weakness
SCO1	Respiratory insufficiency due to muscle weakness
SEPN1	Respiratory insufficiency due to muscle weakness
SGK196	Respiratory insufficiency due to muscle weakness
SIGMAR1	Respiratory insufficiency due to muscle weakness
SLC25A1	Respiratory insufficiency due to muscle weakness
SNAP25	Respiratory insufficiency due to muscle weakness
SOD1	Respiratory insufficiency due to muscle weakness
SPEG	Respiratory insufficiency due to muscle weakness
SPG11	Respiratory insufficiency due to muscle weakness
SQSTM1	Respiratory insufficiency due to muscle weakness
SUCLA2	Respiratory insufficiency due to muscle weakness
SYT2	Respiratory insufficiency due to muscle weakness
TACO1	Respiratory insufficiency due to muscle weakness
TARDBP	Respiratory insufficiency due to muscle weakness
TBK1	Respiratory insufficiency due to muscle weakness
TK2	Respiratory insufficiency due to muscle weakness
TMEM5	Respiratory insufficiency due to muscle weakness
TNPO3	Respiratory insufficiency due to muscle weakness
TPI1	Respiratory insufficiency due to muscle weakness
TPI1P1	Respiratory insufficiency due to muscle weakness
TPM2	Respiratory insufficiency due to muscle weakness
TPM3	Respiratory insufficiency due to muscle weakness
TREM2	Respiratory insufficiency due to muscle weakness
TRMU	Respiratory insufficiency due to muscle weakness
TRNE	Respiratory insufficiency due to muscle weakness

TTN	Respiratory insufficiency due to muscle weakness
UBA1	Respiratory insufficiency due to muscle weakness
UBQLN2	Respiratory insufficiency due to muscle weakness
UNC13A	Respiratory insufficiency due to muscle weakness
VAPB	Respiratory insufficiency due to muscle weakness
VCP	Respiratory insufficiency due to muscle weakness
YARS2	Respiratory insufficiency due to muscle weakness
ALAD	Respiratory paralysis
CPOX	Respiratory paralysis
HMBS	Respiratory paralysis
IKBKB	Respiratory tract infection
PEX1	Respiratory tract infection
PEX10	Respiratory tract infection
PEX11B	Respiratory tract infection
PEX12	Respiratory tract infection
PEX13	Respiratory tract infection
PEX14	Respiratory tract infection
PEX16	Respiratory tract infection
PEX19	Respiratory tract infection
PEX2	Respiratory tract infection
PEX26	Respiratory tract infection
PEX3	Respiratory tract infection
PEX5	Respiratory tract infection
PEX6	Respiratory tract infection
SLC12A6	Respiratory tract infection
ACD	Restrictive lung disease
ACP5	Restrictive lung disease
C16ORF57	Restrictive lung disease
C17ORF68	Restrictive lung disease
CD19	Restrictive lung disease
CD81	Restrictive lung disease

COL2A1	Restrictive lung disease
CR2	Restrictive lung disease
CSF2RA	Restrictive lung disease
CSF2RB	Restrictive lung disease
CTC1	Restrictive lung disease
CTLA4	Restrictive lung disease
DDR2	Restrictive lung disease
DKC1	Restrictive lung disease
DNASE1L3	Restrictive lung disease
FAM38B	Restrictive lung disease
FLNB	Restrictive lung disease
GALNS	Restrictive lung disease
GBA	Restrictive lung disease
GLB1	Restrictive lung disease
HLA-B	Restrictive lung disease
HLA-C	Restrictive lung disease
HLA-DPB1	Restrictive lung disease
HLAB	Restrictive lung disease
HPS1	Restrictive lung disease
HPS4	Restrictive lung disease
ICOS	Restrictive lung disease
IGHMBP2	Restrictive lung disease
IKZF1	Restrictive lung disease
LRBA	Restrictive lung disease
MEGF10	Restrictive lung disease
MS4A1	Restrictive lung disease
NFKB1	Restrictive lung disease
NFKB2	Restrictive lung disease
NHP2	Restrictive lung disease
NLRP3	Restrictive lung disease
NOP10	Restrictive lung disease

PARN	Restrictive lung disease
PIEZO2	Restrictive lung disease
PRKCD	Restrictive lung disease
PRTN3	Restrictive lung disease
PTPN22	Restrictive lung disease
RTEL1	Restrictive lung disease
SCARB2	Restrictive lung disease
SGCG	Restrictive lung disease
TERC	Restrictive lung disease
TERT	Restrictive lung disease
TINF2	Restrictive lung disease
TNFRSF13B	Restrictive lung disease
TNFRSF13C	Restrictive lung disease
TNFRSF6B	Restrictive lung disease
TNFSF12	Restrictive lung disease
TNFSF12-TNFSF1	Restrictive lung disease
TNFSF13	Restrictive lung disease
TSC1	Restrictive lung disease
TSC2	Restrictive lung disease
USB1	Restrictive lung disease
WRAP53	Restrictive lung disease
ACTA1	Restrictive respiratory insufficiency
C6ORF59	Restrictive respiratory insufficiency
DLL3	Restrictive respiratory insufficiency
DUX4	Restrictive respiratory insufficiency
FKRP	Restrictive respiratory insufficiency
FKTN	Restrictive respiratory insufficiency
FRG1	Restrictive respiratory insufficiency
GMPPB	Restrictive respiratory insufficiency
HES7	Restrictive respiratory insufficiency
ISPD	Restrictive respiratory insufficiency

LARGE	Restrictive respiratory insufficiency
LFNG	Restrictive respiratory insufficiency
LOC100288687	Restrictive respiratory insufficiency
MESP2	Restrictive respiratory insufficiency
MYH7	Restrictive respiratory insufficiency
POMGNT1	Restrictive respiratory insufficiency
POMT1	Restrictive respiratory insufficiency
POMT2	Restrictive respiratory insufficiency
RIPPLY2	Restrictive respiratory insufficiency
SEPN1	Restrictive respiratory insufficiency
SLC12A6	Restrictive respiratory insufficiency
SLC34A2	Restrictive respiratory insufficiency
SMCHD1	Restrictive respiratory insufficiency
STAC3	Restrictive respiratory insufficiency
TTN	Restrictive respiratory insufficiency
TGFB1	Sarcoidosis
TGFB2	Sarcoidosis
TGFBR1	Sarcoidosis
TGFBR2	Sarcoidosis
THAS	Sarcoidosis
TIMP1	Silicosis
TIMP3	Silicosis
TINF2	Silicosis
TIRAP	Silicosis
ADAMTS2	Spontaneous neonatal pneumothorax
COL3A1	Spontaneous pneumothorax
COL5A1	Spontaneous pneumothorax
FLCN	Spontaneous pneumothorax
ATRIP	Systemic lupus erythematosus
C1QA	Systemic lupus erythematosus
C1QB	Systemic lupus erythematosus

C1QC	Systemic lupus erythematosus
C1R	Systemic lupus erythematosus
C1S	Systemic lupus erythematosus
C2	Systemic lupus erythematosus
C4A	Systemic lupus erythematosus
C4B	Systemic lupus erythematosus
C5	Systemic lupus erythematosus
C6	Systemic lupus erythematosus
C7	Systemic lupus erythematosus
C8A	Systemic lupus erythematosus
C8B	Systemic lupus erythematosus
C8G	Systemic lupus erythematosus
C9	Systemic lupus erythematosus
DNASE1L3	Systemic lupus erythematosus
ITGAM	Systemic lupus erythematosus
MASP2	Systemic lupus erythematosus
PEPD	Systemic lupus erythematosus
PRKCD	Systemic lupus erythematosus
SERPING1	Systemic lupus erythematosus
TREX1	Systemic lupus erythematosus
ABCA3	Tachypnea
ABCD4	Tachypnea
ACADVL	Tachypnea
ACTA2	Tachypnea
AHI1	Tachypnea
ALS2CR4	Tachypnea
ARL13B	Tachypnea
B9D1	Tachypnea
BTD	Tachypnea
C5orf42	Tachypnea
CA5A	Tachypnea

CASR	Tachypnea
CC2D2A	Tachypnea
CEP290	Tachypnea
CEP41	Tachypnea
CSF2RA	Tachypnea
CSF2RB	Tachypnea
CSPP1	Tachypnea
HLCS	Tachypnea
IGHMBP2	Tachypnea
INPP5E	Tachypnea
KIAA0586	Tachypnea
LRPPRC	Tachypnea
MEGF10	Tachypnea
MKS1	Tachypnea
OXCT1	Tachypnea
PAM16	Tachypnea
PCCA	Tachypnea
PCCB	Tachypnea
SFTPB	Tachypnea
SFTPC	Tachypnea
TCTN1	Tachypnea
TCTN2	Tachypnea
TIMM16	Tachypnea
TMEM138	Tachypnea
TMEM216	Tachypnea
TMEM231	Tachypnea
TMEM237	Tachypnea
TMEM67	Tachypnea
TSGA14	Tachypnea
ZNF423	Tachypnea
BMPER	Thoracic hypoplasia

CANT1	Thoracic hypoplasia
CEP120	Thoracic hypoplasia
COL11A1	Thoracic hypoplasia
COL11A2	Thoracic hypoplasia
DDR2	Thoracic hypoplasia
DOK7	Thoracic hypoplasia
DYNC2H1	Thoracic hypoplasia
EHHADH	Thoracic hypoplasia
FAM20C	Thoracic hypoplasia
GBA	Thoracic hypoplasia
GNE	Thoracic hypoplasia
HSD17B4	Thoracic hypoplasia
HSPG2	Thoracic hypoplasia
IFT140	Thoracic hypoplasia
IFT172	Thoracic hypoplasia
IFT80	Thoracic hypoplasia
MATN3	Thoracic hypoplasia
MUSK	Thoracic hypoplasia
NEK1	Thoracic hypoplasia
RAPSN	Thoracic hypoplasia
SLC26A2	Thoracic hypoplasia
SNRPB	Thoracic hypoplasia
SOX9	Thoracic hypoplasia
TTC21B	Thoracic hypoplasia
WDR19	Thoracic hypoplasia
WDR34	Thoracic hypoplasia
WDR35	Thoracic hypoplasia
WDR60	Thoracic hypoplasia
XYLT1	Thoracic hypoplasia
DHCR24	Total anomalous pulmonary venous return
ADAMTSL2	Tracheal stenosis

AFF4	Tracheal stenosis
COL2A1	Tracheal stenosis
DHCR7	Tracheal stenosis
EBP	Tracheal stenosis
FBN1	Tracheal stenosis
FLNA	Tracheal stenosis
FLNB	Tracheal stenosis
FOXI1	Tracheal stenosis
FRAS1	Tracheal stenosis
FREM2	Tracheal stenosis
GRIP1	Tracheal stenosis
HOXD13	Tracheal stenosis
HYLS1	Tracheal stenosis
IDS	Tracheal stenosis
IDUA	Tracheal stenosis
KCNJ10	Tracheal stenosis
KIF7	Tracheal stenosis
LOC653348	Tracheal stenosis
MGP	Tracheal stenosis
PCNT	Tracheal stenosis
RMRP	Tracheal stenosis
SLC26A4	Tracheal stenosis
WNT3	Tracheal stenosis
EHMT1	Tracheobronchomalacia
IDS	Tracheobronchomalacia
SOX9	Tracheobronchomalacia
BAZ1B	Tracheoesophageal fistula
BRCA2	Tracheoesophageal fistula
BRIP1	Tracheoesophageal fistula
BTBD12	Tracheoesophageal fistula
C16ORF57	Tracheoesophageal fistula

C17ORF68	Tracheoesophageal fistula
CHD7	Tracheoesophageal fistula
CLIP2	Tracheoesophageal fistula
COL7A1	Tracheoesophageal fistula
CTC1	Tracheoesophageal fistula
CYBA	Tracheoesophageal fistula
CYBB	Tracheoesophageal fistula
CYTSA	Tracheoesophageal fistula
DKC1	Tracheoesophageal fistula
ELN	Tracheoesophageal fistula
ERCC4	Tracheoesophageal fistula
FANCA	Tracheoesophageal fistula
FANCB	Tracheoesophageal fistula
FANCC	Tracheoesophageal fistula
FANCD2	Tracheoesophageal fistula
FANCE	Tracheoesophageal fistula
FANCF	Tracheoesophageal fistula
FANCG	Tracheoesophageal fistula
FANCI	Tracheoesophageal fistula
FANCL	Tracheoesophageal fistula
FANCM	Tracheoesophageal fistula
FBN2	Tracheoesophageal fistula
FERMT1	Tracheoesophageal fistula
FGF20	Tracheoesophageal fistula
FGFR1	Tracheoesophageal fistula
FOXF1	Tracheoesophageal fistula
FUZ	Tracheoesophageal fistula
GLI2	Tracheoesophageal fistula
GLI3	Tracheoesophageal fistula
GTF2I	Tracheoesophageal fistula
GTF2IRD1	Tracheoesophageal fistula

HESX1	Tracheoesophageal fistula
HOXD13	Tracheoesophageal fistula
ITGA8	Tracheoesophageal fistula
LAMA3	Tracheoesophageal fistula
LHX4	Tracheoesophageal fistula
LIMK1	Tracheoesophageal fistula
LOC100093631	Tracheoesophageal fistula
MMP1	Tracheoesophageal fistula
MYCN	Tracheoesophageal fistula
NCF1	Tracheoesophageal fistula
NCF1C	Tracheoesophageal fistula
NCF2	Tracheoesophageal fistula
NCF4	Tracheoesophageal fistula
NHP2	Tracheoesophageal fistula
NOP10	Tracheoesophageal fistula
NOTCH3	Tracheoesophageal fistula
OTX2	Tracheoesophageal fistula
PALB2	Tracheoesophageal fistula
PARN	Tracheoesophageal fistula
PDGFRB	Tracheoesophageal fistula
POLR1C	Tracheoesophageal fistula
POLR1D	Tracheoesophageal fistula
POU1F1	Tracheoesophageal fistula
PROKR2	Tracheoesophageal fistula
PROP1	Tracheoesophageal fistula
RAD51C	Tracheoesophageal fistula
RET	Tracheoesophageal fistula
RFC2	Tracheoesophageal fistula
RTEL1	Tracheoesophageal fistula
SEMA3E	Tracheoesophageal fistula
SLX4	Tracheoesophageal fistula

SOX2	Tracheoesophageal fistula
SOX3	Tracheoesophageal fistula
SPECC1L	Tracheoesophageal fistula
TBL2	Tracheoesophageal fistula
TCOF1	Tracheoesophageal fistula
TERC	Tracheoesophageal fistula
TERT	Tracheoesophageal fistula
TINF2	Tracheoesophageal fistula
TNFRSF6B	Tracheoesophageal fistula
UBE2T	Tracheoesophageal fistula
USB1	Tracheoesophageal fistula
VANGL1	Tracheoesophageal fistula
WNT7A	Tracheoesophageal fistula
WRAP53	Tracheoesophageal fistula
ZIC3	Tracheoesophageal fistula
AMER1	Tracheomalacia
BMPER	Tracheomalacia
CDC6	Tracheomalacia
CDT1	Tracheomalacia
COL2A1	Tracheomalacia
FAM123B	Tracheomalacia
FGFR2	Tracheomalacia
FLNB	Tracheomalacia
HDAC4	Tracheomalacia
HRAS	Tracheomalacia
KIF22	Tracheomalacia
LTBP4	Tracheomalacia
ORC1	Tracheomalacia
ORC1L	Tracheomalacia
ORC4	Tracheomalacia
ORC4L	Tracheomalacia

ORC6	Tracheomalacia
ORC6L	Tracheomalacia
OTX2	Tracheomalacia
PAX3	Tracheomalacia
PRRX1	Tracheomalacia
RAB3GAP1	Tracheomalacia
RAB3GAP2	Tracheomalacia
SNRPB	Tracheomalacia
SOX9	Tracheomalacia
MBTPS2	Unilateral chest hypoplasia
ARVCF	Unilateral primary pulmonary dysgenesis
COMT	Unilateral primary pulmonary dysgenesis
GP1BB	Unilateral primary pulmonary dysgenesis
HIRA	Unilateral primary pulmonary dysgenesis
TBX1	Unilateral primary pulmonary dysgenesis
UFD1L	Unilateral primary pulmonary dysgenesis
FH	Vagal paraganglioma
LOC100130320	Vagal paraganglioma
MAX	Vagal paraganglioma
RET	Vagal paraganglioma
SDHA	Vagal paraganglioma
SDHAF2	Vagal paraganglioma
SDHB	Vagal paraganglioma
SDHC	Vagal paraganglioma
SDHD	Vagal paraganglioma
TMEM127	Vagal paraganglioma
IGHMBP2	Ventilator dependence with inability to wean
MEGF10	Ventilator dependence with inability to wean
A2ML1	Ventricular septal defect
ABCB6	Ventricular septal defect
ACTC1	Ventricular septal defect

ACVR2B	Ventricular septal defect
ADAMTS10	Ventricular septal defect
AKT3	Ventricular septal defect
AMER1	Ventricular septal defect
ANKRD11	Ventricular septal defect
APC2	Ventricular septal defect
ARHGAP31	Ventricular septal defect
ARID1A	Ventricular septal defect
ARID1B	Ventricular septal defect
ARVCF	Ventricular septal defect
ARX	Ventricular septal defect
ASXL1	Ventricular septal defect
B3GALT6	Ventricular septal defect
B3GALTL	Ventricular septal defect
B3GLCT	Ventricular septal defect
B9D1	Ventricular septal defect
B9D2	Ventricular septal defect
BAZ1B	Ventricular septal defect
BCOR	Ventricular septal defect
BCR	Ventricular septal defect
BRAF	Ventricular septal defect
BRCA2	Ventricular septal defect
BRIP1	Ventricular septal defect
BTBD12	Ventricular septal defect
BUB1	Ventricular septal defect
BUB1B	Ventricular septal defect
BUB3	Ventricular septal defect
C14ORF19	Ventricular septal defect
C20ORF7	Ventricular septal defect
C2ORF86	Ventricular septal defect
C3ORF64	Ventricular septal defect

C5orf42	Ventricular septal defect
C7ORF11	Ventricular septal defect
C8ORF38	Ventricular septal defect
C8ORF62	Ventricular septal defect
CACNA1D	Ventricular septal defect
CANT1	Ventricular septal defect
CC2D2A	Ventricular septal defect
CCBE1	Ventricular septal defect
CCDC22	Ventricular septal defect
CCND2	Ventricular septal defect
CD96	Ventricular septal defect
CEP120	Ventricular septal defect
CEP290	Ventricular septal defect
CEP57	Ventricular septal defect
CERS1	Ventricular septal defect
CHD7	Ventricular septal defect
CHRM3	Ventricular septal defect
CHST14	Ventricular septal defect
CHST3	Ventricular septal defect
CKAP2L	Ventricular septal defect
CLIP2	Ventricular septal defect
COL11A2	Ventricular septal defect
COL2A1	Ventricular septal defect
COMT	Ventricular septal defect
COX15	Ventricular septal defect
COX7B	Ventricular septal defect
CRB2	Ventricular septal defect
CREBBP	Ventricular septal defect
CRKL	Ventricular septal defect
CSPP1	Ventricular septal defect
CYTSA	Ventricular septal defect

DDX11	Ventricular septal defect
DHCR7	Ventricular septal defect
DLL4	Ventricular septal defect
DOCK6	Ventricular septal defect
DSE	Ventricular septal defect
DSG1	Ventricular septal defect
DTNA	Ventricular septal defect
DYNC2H1	Ventricular septal defect
ECE1	Ventricular septal defect
ECHS1	Ventricular septal defect
EFTUD2	Ventricular septal defect
EHMT1	Ventricular septal defect
ELN	Ventricular septal defect
EOGT	Ventricular septal defect
ERBB3	Ventricular septal defect
ERCC2	Ventricular septal defect
ERCC3	Ventricular septal defect
ERCC4	Ventricular septal defect
ESCO2	Ventricular septal defect
EVC	Ventricular septal defect
EVC2	Ventricular septal defect
FADD	Ventricular septal defect
FAM123B	Ventricular septal defect
FAM38B	Ventricular septal defect
FANCA	Ventricular septal defect
FANCB	Ventricular septal defect
FANCC	Ventricular septal defect
FANCD2	Ventricular septal defect
FANCE	Ventricular septal defect
FANCF	Ventricular septal defect
FANCG	Ventricular septal defect

FANCI	Ventricular septal defect
FANCL	Ventricular septal defect
FANCM	Ventricular septal defect
FAT4	Ventricular septal defect
FBN1	Ventricular septal defect
FBN2	Ventricular septal defect
FGFR2	Ventricular septal defect
FIG4	Ventricular septal defect
FLNB	Ventricular septal defect
FOXC2	Ventricular septal defect
FOXF1	Ventricular septal defect
FOXRED1	Ventricular septal defect
FTO	Ventricular septal defect
GABRD	Ventricular septal defect
GATA1	Ventricular septal defect
GATA3	Ventricular septal defect
GATA4	Ventricular septal defect
GDF1	Ventricular septal defect
GDF3	Ventricular septal defect
GDF6	Ventricular septal defect
GJA1	Ventricular septal defect
GLI3	Ventricular septal defect
GP1BB	Ventricular septal defect
GPC3	Ventricular septal defect
GPC4	Ventricular septal defect
GPC6	Ventricular septal defect
GTF2H5	Ventricular septal defect
GTF2I	Ventricular septal defect
GTF2IRD1	Ventricular septal defect
HCCS	Ventricular septal defect
HDAC8	Ventricular septal defect

HIRA	Ventricular septal defect
HOXA13	Ventricular septal defect
HOXD13	Ventricular septal defect
HRAS	Ventricular septal defect
HYLS1	Ventricular septal defect
IFT140	Ventricular septal defect
IFT172	Ventricular septal defect
IFT80	Ventricular septal defect
IGBP1	Ventricular septal defect
IMPAD1	Ventricular septal defect
JAG1	Ventricular septal defect
KANSL1	Ventricular septal defect
KAT6A	Ventricular septal defect
KAT6B	Ventricular septal defect
KCNAB2	Ventricular septal defect
KDM6A	Ventricular septal defect
KIAA0196	Ventricular septal defect
KIAA1267	Ventricular septal defect
KIF7	Ventricular septal defect
KMT2D	Ventricular septal defect
KRAS	Ventricular septal defect
LASS1	Ventricular septal defect
LBR	Ventricular septal defect
LDB3	Ventricular septal defect
LETM1	Ventricular septal defect
LIMK1	Ventricular septal defect
LIPT1	Ventricular septal defect
LMNA	Ventricular septal defect
LOC100093631	Ventricular septal defect
LOC100128265	Ventricular septal defect
LOC100131801	Ventricular septal defect

LOC344593	Ventricular septal defect
LOC442113	Ventricular septal defect
LONP1	Ventricular septal defect
LRP2	Ventricular septal defect
LRP5	Ventricular septal defect
LTBP2	Ventricular septal defect
LZTR1	Ventricular septal defect
MAPK1	Ventricular septal defect
MED12	Ventricular septal defect
MEGF8	Ventricular septal defect
MEIS2	Ventricular septal defect
MEOX1	Ventricular septal defect
MGAT2	Ventricular septal defect
MGP	Ventricular septal defect
MIB1	Ventricular septal defect
MKKS	Ventricular septal defect
MKS1	Ventricular septal defect
MLL2	Ventricular septal defect
MPLKIP	Ventricular septal defect
MTFMT	Ventricular septal defect
MYBPC3	Ventricular septal defect
MYH7	Ventricular septal defect
MYH7B	Ventricular septal defect
MYST3	Ventricular septal defect
MYST4	Ventricular septal defect
NAA10	Ventricular septal defect
NAIP	Ventricular septal defect
NDUFA10	Ventricular septal defect
NDUFA12	Ventricular septal defect
NDUFA2	Ventricular septal defect
NDUFA4	Ventricular septal defect

NDUFA9	Ventricular septal defect
NDUFAF2	Ventricular septal defect
NDUFAF5	Ventricular septal defect
NDUFAF6	Ventricular septal defect
NDUFB11	Ventricular septal defect
NDUFS1	Ventricular septal defect
NDUFS2	Ventricular septal defect
NDUFS3	Ventricular septal defect
NDUFS4	Ventricular septal defect
NDUFS7	Ventricular septal defect
NDUFS8	Ventricular septal defect
NDUFV1	Ventricular septal defect
NDUFV2	Ventricular septal defect
NEK1	Ventricular septal defect
NELFA	Ventricular septal defect
NIPBL	Ventricular septal defect
NKX2-5	Ventricular septal defect
NODAL	Ventricular septal defect
NOTCH1	Ventricular septal defect
NOTCH2	Ventricular septal defect
NOTCH3	Ventricular septal defect
NR2F2	Ventricular septal defect
NRAS	Ventricular septal defect
NSD1	Ventricular septal defect
NSDHL	Ventricular septal defect
ODZ3	Ventricular septal defect
OFD1	Ventricular septal defect
PALB2	Ventricular septal defect
PDE6D	Ventricular septal defect
PDHA1	Ventricular septal defect
PET100	Ventricular septal defect

PEX1	Ventricular septal defect
PEX10	Ventricular septal defect
PEX11B	Ventricular septal defect
PEX12	Ventricular septal defect
PEX13	Ventricular septal defect
PEX14	Ventricular septal defect
PEX16	Ventricular septal defect
PEX19	Ventricular septal defect
PEX2	Ventricular septal defect
PEX26	Ventricular septal defect
PEX3	Ventricular septal defect
PEX5	Ventricular septal defect
PEX6	Ventricular septal defect
PHGDH	Ventricular septal defect
PIEZO2	Ventricular septal defect
PIGL	Ventricular septal defect
PIK3CA	Ventricular septal defect
PIK3R2	Ventricular septal defect
PORCN	Ventricular septal defect
PQBP1	Ventricular septal defect
PRDM16	Ventricular septal defect
PSAT1	Ventricular septal defect
PTPN11	Ventricular septal defect
RAB23	Ventricular septal defect
RAD21	Ventricular septal defect
RAD51C	Ventricular septal defect
RAF1	Ventricular septal defect
RARB	Ventricular septal defect
RASA2	Ventricular septal defect
RBM8A	Ventricular septal defect
RBP4	Ventricular septal defect

RBPJ	Ventricular septal defect
RFC2	Ventricular septal defect
RIT1	Ventricular septal defect
RNF113A	Ventricular septal defect
ROR2	Ventricular septal defect
RPGRIP1	Ventricular septal defect
RPGRIP1L	Ventricular septal defect
RPL11	Ventricular septal defect
RPL15	Ventricular septal defect
RPL15P17	Ventricular septal defect
RPL15P18	Ventricular septal defect
RPL15P22	Ventricular septal defect
RPL15P3	Ventricular septal defect
RPL15P7	Ventricular septal defect
RPL26	Ventricular septal defect
RPL26P16	Ventricular septal defect
RPL26P19	Ventricular septal defect
RPL26P33	Ventricular septal defect
RPL26P6	Ventricular septal defect
RPL35A	Ventricular septal defect
RPL5	Ventricular septal defect
RPL5P1	Ventricular septal defect
RPL5P34	Ventricular septal defect
RPS10	Ventricular septal defect
RPS10P11	Ventricular septal defect
RPS10P13	Ventricular septal defect
RPS10P22	Ventricular septal defect
RPS10P4	Ventricular septal defect
RPS10P7	Ventricular septal defect
RPS17	Ventricular septal defect
RPS17L	Ventricular septal defect

RPS19	Ventricular septal defect
RPS19P3	Ventricular septal defect
RPS24	Ventricular septal defect
RPS26	Ventricular septal defect
RPS26P2	Ventricular septal defect
RPS26P20	Ventricular septal defect
RPS26P25	Ventricular septal defect
RPS26P31	Ventricular septal defect
RPS26P35	Ventricular septal defect
RPS26P38	Ventricular septal defect
RPS26P39	Ventricular septal defect
RPS26P50	Ventricular septal defect
RPS26P53	Ventricular septal defect
RPS26P54	Ventricular septal defect
RPS26P6	Ventricular septal defect
RPS26P8	Ventricular septal defect
RPS28	Ventricular septal defect
RPS28P6	Ventricular septal defect
RPS28P9	Ventricular septal defect
RPS29	Ventricular septal defect
RPS29P11	Ventricular septal defect
RPS29P16	Ventricular septal defect
RPS29P17	Ventricular septal defect
RPS29P3	Ventricular septal defect
RPS29P9	Ventricular septal defect
RPS7	Ventricular septal defect
RPS7P10	Ventricular septal defect
RPS7P11	Ventricular septal defect
RPS7P4	Ventricular septal defect
SALL1	Ventricular septal defect
SALL4	Ventricular septal defect

SDHA	Ventricular septal defect
SEMA3E	Ventricular septal defect
SETD2	Ventricular septal defect
SF3B4	Ventricular septal defect
SHANK3	Ventricular septal defect
SHH	Ventricular septal defect
SHOC2	Ventricular septal defect
SKI	Ventricular septal defect
SKIV2L	Ventricular septal defect
SLC19A2	Ventricular septal defect
SLC19A3	Ventricular septal defect
SLC29A3	Ventricular septal defect
SLX4	Ventricular septal defect
SMARCA4	Ventricular septal defect
SMARCB1	Ventricular septal defect
SMARCE1	Ventricular septal defect
SMC1A	Ventricular septal defect
SMC3	Ventricular septal defect
SMN1	Ventricular septal defect
SMN2	Ventricular septal defect
SNRPB	Ventricular septal defect
SOS1	Ventricular septal defect
SOS2	Ventricular septal defect
SOX11	Ventricular septal defect
SOX2	Ventricular septal defect
SP9	Ventricular septal defect
SPECC1L	Ventricular septal defect
STAMBP	Ventricular septal defect
STRA6	Ventricular septal defect
SURF1	Ventricular septal defect
TACO1	Ventricular septal defect

TALDO1	Ventricular septal defect
TAZ	Ventricular septal defect
TBL2	Ventricular septal defect
TBX1	Ventricular septal defect
TBX3	Ventricular septal defect
TBX5	Ventricular septal defect
TCTN2	Ventricular septal defect
TCTN3	Ventricular septal defect
TENM3	Ventricular septal defect
TFAP2B	Ventricular septal defect
TGDS	Ventricular septal defect
TGFB3	Ventricular septal defect
TMEM216	Ventricular septal defect
TMEM231	Ventricular septal defect
TMEM67	Ventricular septal defect
TNNT2	Ventricular septal defect
TP63	Ventricular septal defect
TPM1	Ventricular septal defect
TSR2	Ventricular septal defect
TTC21B	Ventricular septal defect
TTC37	Ventricular septal defect
TTC7A	Ventricular septal defect
TXNL4A	Ventricular septal defect
UBE2T	Ventricular septal defect
UBR1	Ventricular septal defect
UFD1L	Ventricular septal defect
UMPS	Ventricular septal defect
UPF3B	Ventricular septal defect
VIPAR	Ventricular septal defect
VIPAS39	Ventricular septal defect
VPS13B	Ventricular septal defect

VPS33B	Ventricular septal defect
VSX2	Ventricular septal defect
WDPCP	Ventricular septal defect
WDR19	Ventricular septal defect
WDR34	Ventricular septal defect
WDR35	Ventricular septal defect
WDR60	Ventricular septal defect
WHSC1	Ventricular septal defect
WHSC2	Ventricular septal defect
WT1	Ventricular septal defect
XYLT1	Ventricular septal defect
XYLT2	Ventricular septal defect
ZDHHC9	Ventricular septal defect
ZEB2	Ventricular septal defect
ZIC3	Ventricular septal defect
ACTA1	Weak cry
AGRN	Weak cry
CHAT	Weak cry
CHRNA1	Weak cry
CHRNB1	Weak cry
CHRND	Weak cry
CHRNE	Weak cry
COLQ	Weak cry
COQ9	Weak cry
CRYAB	Weak cry
CYTSA	Weak cry
DOK7	Weak cry
HACD1	Weak cry
HDAC8	Weak cry
IGHMBP2	Weak cry
ITGA7	Weak cry

LAMA3	Weak cry
LAMB2	Weak cry
LRP4	Weak cry
MEGF10	Weak cry
MUSK	Weak cry
MYL2	Weak cry
NIPBL	Weak cry
PTPLA	Weak cry
RAD21	Weak cry
RAPSN	Weak cry
SCN4A	Weak cry
SEPN1	Weak cry
SLC25A1	Weak cry
SMC1A	Weak cry
SMC3	Weak cry
SNAP25	Weak cry
SPECC1L	Weak cry
SYT2	Weak cry
TPM2	Weak cry
TPM3	Weak cry
C20ORF54	Weak voice
DCTN1	Weak voice
GPR172A	Weak voice
HSPG2	Weak voice
SLC52A2	Weak voice
SLC52A3	Weak voice
TRIM37	Weak voice
