# Supplementary material for: Lung disease network reveals impact of comorbidity on SARS-CoV-2 infection and opportunities of drug repurposing
Source: BMC Med Genomics. 2021 Sep 17;14:226. doi: 10.1186/s12920-021-01079-7 (PMC8447809; doi:10.1186/s12920-021-01079-7)
Supplement: Supplementary file 4 — Additional file 4. Table S3. Disease-gene association data of STN. [file 12920_2021_1079_MOESM4_ESM.pdf]

Supplementary Table 3: Disease-gene association data of STN	
**Gene**	**Disorder**
BUB1	Abnormal lung lobation
BUB1B	Abnormal lung lobation
BUB3	Abnormal lung lobation
CEP57	Abnormal lung lobation
DHCR7	Abnormal lung lobation
FOXF1	Abnormal lung lobation
GRIP1	Abnormal lung lobation
HYLS1	Abnormal lung lobation
LBR	Abnormal lung lobation
SKI	Abnormal lung lobation
WT1	Abnormal lung lobation
DNAAF2	Abnormal respiratory motile cilium morphology
DNAAF5	Abnormal respiratory motile cilium morphology
OFD1	Abnormal respiratory motile cilium morphology
SPAG1	Abnormal respiratory motile cilium morphology
EIF4A3	Abnormality of the aryepiglottic fold
FOXC2	Abnormality of the pulmonary vasculature
FOXF1	Abnormality of the pulmonary veins
DNAAF2	Absent respiratory ciliary axoneme radial spokes
DNAAF5	Absent respiratory ciliary axoneme radial spokes
OFD1	Absent respiratory ciliary axoneme radial spokes
SPAG1	Absent respiratory ciliary axoneme radial spokes
BSCL2	Acute pancreatitis
CAV1	Acute pancreatitis
FOS	Acute pancreatitis
LMNA	Acute pancreatitis
PPARG	Acute pancreatitis
SHH	Agenesis of pulmonary vessels
DPP9	Alveolar cell carcinoma
DSP	Alveolar cell carcinoma
EGFR	Alveolar cell carcinoma
ERBB2	Alveolar cell carcinoma
FAM13A	Alveolar cell carcinoma
KRAS	Alveolar cell carcinoma
MAP3K8	Alveolar cell carcinoma
PARN	Alveolar cell carcinoma
PPP2R1B	Alveolar cell carcinoma
RASSF1	Alveolar cell carcinoma
TFE3	Alveolar soft part sarcoma
SNRPB	Anomalous tracheal cartilage
AGTR1	Aplasia/Hypoplasia of the lungs
CHRM3	Aplasia/Hypoplasia of the lungs

DHCR7	Aplasia/Hypoplasia of the lungs
FGFR3	Aplasia/Hypoplasia of the lungs
FLNA	Aplasia/Hypoplasia of the lungs
FLNB	Aplasia/Hypoplasia of the lungs
GRIP1	Aplasia/Hypoplasia of the lungs
ITGA8	Aplasia/Hypoplasia of the lungs
LETM1	Aplasia/Hypoplasia of the lungs
LMNA	Aplasia/Hypoplasia of the lungs
NSDHL	Aplasia/Hypoplasia of the lungs
PHGDH	Aplasia/Hypoplasia of the lungs
PTH1R	Aplasia/Hypoplasia of the lungs
TCTN3	Aplasia/Hypoplasia of the lungs
TRPV4	Aplasia/Hypoplasia of the lungs
WT1	Aplasia/Hypoplasia of the lungs
ABCD3	Apnea
AGRN	Apnea
AMER1	Apnea
ARL13B	Apnea
BUB1	Apnea
BUB1B	Apnea
BUB3	Apnea
CDKL5	Apnea
CDKN1C	Apnea
CEP290	Apnea
CEP57	Apnea
CLCN7	Apnea
COL5A1	Apnea
CSPP1	Apnea
CTSD	Apnea
DST	Apnea
ECHS1	Apnea
EPOR	Apnea
FBN1	Apnea
FGFR3	Apnea
FOXRED	Apnea
GBA	Apnea
GFAP	Apnea
GLUL	Apnea
GNAI3	Apnea
HSPD1	Apnea
LRP4	Apnea
MECP2	Apnea
MT-CO1	Apnea
MT-ND1	Apnea

MT-ND6	Apnea
NDUFA1(	Apnea
NDUFA1:	Apnea
NDUFAF:	Apnea
NDUFS1	Apnea
NDUFS2	Apnea
NDUFS3	Apnea
NDUFS7	Apnea
NDUFS8	Apnea
NDUFV2	Apnea
OFD1	Apnea
PCCB	Apnea
PCK1	Apnea
PDE6D	Apnea
PDHA1	Apnea
PEX1	Apnea
PEX14	Apnea
PEX19	Apnea
PEX2	Apnea
PEX5	Apnea
PRNP	Apnea
RARS2	Apnea
RPGRIP1	Apnea
RPS6KA3	Apnea
RUNX2	Apnea
SDHA	Apnea
SKI	Apnea
SURF1	Apnea
TCF4	Apnea
TCTN3	Apnea
TMEM23	Apnea
TWIST1	Apnea
WFS1	Apnea
AGRN	Apneic episodes precipitated by illness, fatigue, stress
LRP4	Apneic episodes precipitated by illness, fatigue, stress
PDHA1	Apneic episodes precipitated by illness, fatigue, stress
SLC25A1	Apneic episodes precipitated by illness, fatigue, stress
ATXN2	Aspiration
CHMP2B	Aspiration
DCTN1	Aspiration
ERBB4	Aspiration
FUS	Aspiration
HNRNPA	Aspiration
MATR3	Aspiration

OPTN	Aspiration
PFN1	Aspiration
PON2	Aspiration
SQSTM1	Aspiration
TARDBP	Aspiration
TBK1	Aspiration
UNC13A	Aspiration
VAPB	Aspiration
VCP	Aspiration
AFF4	Aspiration pneumonia
CSF2	Asthma
ARL6	Asthma
CASP8	Asthma
CD81	Asthma
CEP290	Asthma
CFTR	Asthma
COMT	Asthma
DCTN4	Asthma
DLL1	Asthma
DNAAF2	Asthma
DNAAF5	Asthma
DOCK8	Asthma
ERCC3	Asthma
FLG	Asthma
GTF2H5	Asthma
HIRA	Asthma
IFT27	Asthma
LIG4	Asthma
LRBA	Asthma
LZTFL1	Asthma
NFKB1	Asthma
NFKB2	Asthma
OFD1	Asthma
PEPD	Asthma
PRKCD	Asthma
PTCH1	Asthma
SHH	Asthma
SPAG1	Asthma
TALDO1	Asthma
TRIM32	Asthma
CCND1	Asthma
CCNG1	Asthma
CCNL2	Asthma
CD36	Asthma

CD46	Asthma
CDH1	Asthma
CDKN1A	Asthma
CDKN1B	Asthma
CDKN1C	Asthma
CDKN2A	Asthma
CFLAR	Asthma
CHEK2	Asthma
CISH	Asthma
COL6A1	Asthma
CORO1A	Asthma
CTLA4	Asthma
COMT	Atelectasis
DNAAF2	Atelectasis
DNAAF5	Atelectasis
EFEMP2	Atelectasis
FBLN5	Atelectasis
HIRA	Atelectasis
OCRL	Atelectasis
OFD1	Atelectasis
SPAG1	Atelectasis
STAT3	Atelectasis
TSC1	Atelectasis
TSC2	Atelectasis
ARL13B	Bell-shaped thorax
CSPP1	Bell-shaped thorax
PAM16	Bell-shaped thorax
PEX1	Bell-shaped thorax
PEX14	Bell-shaped thorax
PEX19	Bell-shaped thorax
PEX2	Bell-shaped thorax
PEX5	Bell-shaped thorax
SNRPB	Bell-shaped thorax
WDR35	Bell-shaped thorax
SHH	Bilateral lung agenesis
ARL13B	Breathing dysregulation
CSPP1	Breathing dysregulation
BLM	Bronchiectasis
CD81	Bronchiectasis
CFTR	Bronchiectasis
DCTN4	Bronchiectasis
DNAAF2	Bronchiectasis
DNAAF5	Bronchiectasis
DNMT3B	Bronchiectasis

HELLS	Bronchiectasis
IGHG1	Bronchiectasis
LRBA	Bronchiectasis
NFKB1	Bronchiectasis
NFKB2	Bronchiectasis
OFD1	Bronchiectasis
PRKCD	Bronchiectasis
SCNN1A	Bronchiectasis
SPAG1	Bronchiectasis
TAPBP	Bronchiectasis
TCF3	Bronchiectasis
ZBTB24	Bronchiectasis
CYB5R3	Bronchiectasis
CYP1A1	Bronchiectasis
ARL6	Bronchiolitis
CEP290	Bronchiolitis
IFT27	Bronchiolitis
LZTFL1	Bronchiolitis
TAPBP	Bronchiolitis
TRIM32	Bronchiolitis
AKT1	Bronchogenic cyst
PTCH1	Bronchogenic cyst
PTEN	Bronchogenic cyst
SDHB	Bronchogenic cyst
CDC6	Bronchomalacia
FLNB	Bronchomalacia
HRAS	Bronchomalacia
ORC1	Bronchomalacia
POR	Bronchomalacia
CDKN1A	Carcinoid tumor
CDKN1B	Carcinoid tumor
CDKN2C	Carcinoid tumor
MEN1	Carcinoid tumor
NIN	Central hypothyroidism
DCTN1	Central hypoventilation
MECP2	Central hypoventilation
CFTR	Chronic bronchitis
DNAAF2	Chronic bronchitis
DNAAF5	Chronic bronchitis
DNMT3B	Chronic bronchitis
HELLS	Chronic bronchitis
OFD1	Chronic bronchitis
SCNN1A	Chronic bronchitis
SPAG1	Chronic bronchitis

ZBTB24	Chronic bronchitis
AFF4	Chronic lung disease
BLM	Chronic lung disease
CD81	Chronic lung disease
CFTR	Chronic lung disease
COL1A2	Chronic lung disease
DCTN4	Chronic lung disease
FKBP10	Chronic lung disease
LRBA	Chronic lung disease
NFKB1	Chronic lung disease
NFKB2	Chronic lung disease
PEPD	Chronic lung disease
PPIB	Chronic lung disease
PRKCD	Chronic lung disease
SERPINH	Chronic lung disease
CD81	Chronic obstructive pulmonary disease
COMT	Chronic obstructive pulmonary disease
CTLA4	Chronic obstructive pulmonary disease
DNAAF2	Chronic obstructive pulmonary disease
DNAAF5	Chronic obstructive pulmonary disease
GLA	Chronic obstructive pulmonary disease
HIRA	Chronic obstructive pulmonary disease
HLA-DPE	Chronic obstructive pulmonary disease
LRBA	Chronic obstructive pulmonary disease
NCF1	Chronic obstructive pulmonary disease
NCF2	Chronic obstructive pulmonary disease
NFKB1	Chronic obstructive pulmonary disease
NFKB2	Chronic obstructive pulmonary disease
OFD1	Chronic obstructive pulmonary disease
PRKCD	Chronic obstructive pulmonary disease
SERPINA	Chronic obstructive pulmonary disease
SPAG1	Chronic obstructive pulmonary disease
RUNX3	Chronic obstructive pulmonary disease
SCNN1A	Chronic obstructive pulmonary disease
SEC23B	Chronic obstructive pulmonary disease
SELENBI	Chronic obstructive pulmonary disease
ARID1A	Congenital diaphragmatic hernia
ARID1B	Congenital diaphragmatic hernia
B3GAT3	Congenital diaphragmatic hernia
CD96	Congenital diaphragmatic hernia
CDKN1C	Congenital diaphragmatic hernia
COL5A1	Congenital diaphragmatic hernia
DACT1	Congenital diaphragmatic hernia
DHCR7	Congenital diaphragmatic hernia

EFEMP2	Congenital diaphragmatic hernia
EFNB1	Congenital diaphragmatic hernia
FBLN5	Congenital diaphragmatic hernia
GATA4	Congenital diaphragmatic hernia
GPC3	Congenital diaphragmatic hernia
GPC4	Congenital diaphragmatic hernia
HCCS	Congenital diaphragmatic hernia
HDAC4	Congenital diaphragmatic hernia
HDAC8	Congenital diaphragmatic hernia
KDM6A	Congenital diaphragmatic hernia
LETM1	Congenital diaphragmatic hernia
NDUFB11	Congenital diaphragmatic hernia
RAD21	Congenital diaphragmatic hernia
RPL11	Congenital diaphragmatic hernia
RPL15	Congenital diaphragmatic hernia
RPL26	Congenital diaphragmatic hernia
RPL5	Congenital diaphragmatic hernia
RPS10	Congenital diaphragmatic hernia
RPS19	Congenital diaphragmatic hernia
RPS24	Congenital diaphragmatic hernia
RPS29	Congenital diaphragmatic hernia
RPS7	Congenital diaphragmatic hernia
SHH	Congenital diaphragmatic hernia
SMARCA	Congenital diaphragmatic hernia
SMARCB	Congenital diaphragmatic hernia
SMARCE	Congenital diaphragmatic hernia
SMC1A	Congenital diaphragmatic hernia
SMC3	Congenital diaphragmatic hernia
WT1	Congenital diaphragmatic hernia
ZFPM2	Congenital diaphragmatic hernia
KDM6A	Congenital hypothyroidism
PDE4D	Congenital hypothyroidism
PRKAR1A	Congenital hypothyroidism
SKI	Congenital hypothyroidism
THRA	Congenital hypothyroidism
ACAD9	Congestive heart failure
ACTC1	Congestive heart failure
ACVRL1	Congestive heart failure
AGGF1	Congestive heart failure
AKAP9	Congestive heart failure
AKT2	Congestive heart failure
ANK2	Congestive heart failure
APOA1	Congestive heart failure
BAG3	Congestive heart failure

CALM1	Congestive heart failure
CALM2	Congestive heart failure
CALM3	Congestive heart failure
CAV1	Congestive heart failure
CCNL2	Congestive heart failure
COL1A2	Congestive heart failure
DDX58	Congestive heart failure
DES	Congestive heart failure
DMD	Congestive heart failure
DNAJC19	Congestive heart failure
DSG2	Congestive heart failure
DSP	Congestive heart failure
DTNA	Congestive heart failure
EFEMP2	Congestive heart failure
ELN	Congestive heart failure
FBLN5	Congestive heart failure
FBN1	Congestive heart failure
FGD1	Congestive heart failure
FGFR3	Congestive heart failure
FHL2	Congestive heart failure
FLNA	Congestive heart failure
FXN	Congestive heart failure
GATAD1	Congestive heart failure
GBA	Congestive heart failure
GLA	Congestive heart failure
GLB1	Congestive heart failure
GNA11	Congestive heart failure
GNPTAB	Congestive heart failure
GTF2IRD	Congestive heart failure
HADHA	Congestive heart failure
HADHB	Congestive heart failure
HBA1	Congestive heart failure
HBA2	Congestive heart failure
HEXB	Congestive heart failure
HLA-DRI	Congestive heart failure
IFIH1	Congestive heart failure
JUP	Congestive heart failure
KCNQ1	Congestive heart failure
KIF1B	Congestive heart failure
LAMA4	Congestive heart failure
LDB3	Congestive heart failure
LMNA	Congestive heart failure
MAX	Congestive heart failure
MIB1	Congestive heart failure

MT-CO1	Congestive heart failure
MT-ND1	Congestive heart failure
MT-ND6	Congestive heart failure
MYH6	Congestive heart failure
MYH7B	Congestive heart failure
NOS1AP	Congestive heart failure
PEX7	Congestive heart failure
PLOD1	Congestive heart failure
PPIB	Congestive heart failure
PRKAR1A	Congestive heart failure
PSEN1	Congestive heart failure
PSEN2	Congestive heart failure
PTEN	Congestive heart failure
RAB3GA	Congestive heart failure
RAF1	Congestive heart failure
RASA1	Congestive heart failure
RFC2	Congestive heart failure
RPL11	Congestive heart failure
RPL15	Congestive heart failure
RPL26	Congestive heart failure
RPL5	Congestive heart failure
RPS10	Congestive heart failure
RPS19	Congestive heart failure
RPS24	Congestive heart failure
RPS29	Congestive heart failure
RPS7	Congestive heart failure
SDHA	Congestive heart failure
SDHB	Congestive heart failure
SMAD4	Congestive heart failure
SNAP29	Congestive heart failure
TAZ	Congestive heart failure
TBL2	Congestive heart failure
TF	Congestive heart failure
TMEM70	Congestive heart failure
TMPO	Congestive heart failure
TPM1	Congestive heart failure
TRIM37	Congestive heart failure
TSC1	Congestive heart failure
TSC2	Congestive heart failure
TTN	Congestive heart failure
VHL	Congestive heart failure
WFS1	Congestive heart failure
BTK	Cor pulmonale
CFTR	Cor pulmonale

DCTN4	Cor pulmonale
FLNA	Cor pulmonale
TSC1	Cortical tubers
TSC2	Cortical tubers
IGHG1	Crohn's disease
TCF3	Crohn's disease
PLOD1	Decreased pulmonary function
SHH	Diaphragmatic eventration
SMPD1	Diffuse reticular or finely nodular infiltrations
CDKL5	Dyskinesia
DDX3X	Dyskinesia
DNAJC13	Dyskinesia
EIF4G1	Dyskinesia
GBA	Dyskinesia
GIGYF2	Dyskinesia
LRRK2	Dyskinesia
MECP2	Dyskinesia
PDGFB	Dyskinesia
PDGFRB	Dyskinesia
PRRT2	Dyskinesia
RAB39B	Dyskinesia
SLC20A2	Dyskinesia
SLC2A1	Dyskinesia
SNCA	Dyskinesia
VPS35	Dyskinesia
XPR1	Dyskinesia
ACTA2	Emphysema
ALDH18A	Emphysema
CD81	Emphysema
CDC6	Emphysema
EFEMP2	Emphysema
ELN	Emphysema
FBLN5	Emphysema
FBN1	Emphysema
GLA	Emphysema
LRBA	Emphysema
NFKB1	Emphysema
NFKB2	Emphysema
ORC1	Emphysema
PRKCD	Emphysema
SERPINA	Emphysema
SMAD3	Emphysema
TAPBP	Emphysema
TSC1	Emphysema

TSC2	Emphysema
COX6B1	Exertional dyspnea
CYB5R3	Exertional dyspnea
DNA2	Exertional dyspnea
DPP9	Exertional dyspnea
DSP	Exertional dyspnea
EPOR	Exertional dyspnea
FAM13A	Exertional dyspnea
MT-CO1	Exertional dyspnea
PARN	Exertional dyspnea
CD46	Hemolytic-uremic syndrome
ACVRL1	Hemoptysis
CARD9	Hemoptysis
COL5A1	Hemoptysis
CTLA4	Hemoptysis
HLA-B	Hemoptysis
HLA-C	Hemoptysis
HLA-DPB	Hemoptysis
IL17RA	Hemoptysis
MLX	Hemoptysis
NOD2	Hemoptysis
SMAD4	Hemoptysis
STAT1	Hemoptysis
TLR4	Hemoptysis
TRAF3IP2	Hemoptysis
TSC1	Hemoptysis
TSC2	Hemoptysis
ACVRL1	High-output congestive heart failure
SMAD4	High-output congestive heart failure
AGGF1	Hydrops fetalis
FLNB	Hydrops fetalis
GBA	Hydrops fetalis
HADHA	Hydrops fetalis
HADHB	Hydrops fetalis
HBA1	Hydrops fetalis
HBA2	Hydrops fetalis
NEU1	Hydrops fetalis
PTH1R	Hydrops fetalis
WDR35	Hydrops fetalis
AKT1	Hyperthyroidism
COMT	Hyperthyroidism
GNAS	Hyperthyroidism
HIRA	Hyperthyroidism
MT-CO1	Hyperthyroidism

MT-ND1	Hyperthyroidism
MT-ND6	Hyperthyroidism
PTEN	Hyperthyroidism
SDHB	Hyperthyroidism
GNMT	Hyperthyroidism
GPC3	Hyperthyroidism
GPX1	Hyperthyroidism
GRB7	Hyperthyroidism
ARL13B	Hyperventilation
CASK	Hyperventilation
CDKL5	Hyperventilation
CSPP1	Hyperventilation
MECP2	Hyperventilation
NECAP1	Hyperventilation
PLCB1	Hyperventilation
SPTAN1	Hyperventilation
STXBP1	Hyperventilation
WWOX	Hyperventilation
AVPR2	Hyponatremia
OCRL	Hyponatremia
SCNN1A	Hyponatremia
STX11	Hyponatremia
COL1A2	Hypoplastic pulmonary veins
FKBP10	Hypoplastic pulmonary veins
PPIB	Hypoplastic pulmonary veins
SERPINH	Hypoplastic pulmonary veins
AKT1	Hypothyroidism
APC	Hypothyroidism
BCOR	Hypothyroidism
BUB1	Hypothyroidism
BUB1B	Hypothyroidism
BUB3	Hypothyroidism
CARD9	Hypothyroidism
CD81	Hypothyroidism
CDKN1C	Hypothyroidism
CEP57	Hypothyroidism
COMT	Hypothyroidism
DCLRE1(	Hypothyroidism
DLL1	Hypothyroidism
EFEMP2	Hypothyroidism
ELN	Hypothyroidism
FBLN5	Hypothyroidism
FMR1	Hypothyroidism
FOXP3	Hypothyroidism

FUCA1	Hypothyroidism
GFAP	Hypothyroidism
GNAS	Hypothyroidism
GPR161	Hypothyroidism
GTF2IRD	Hypothyroidism
HBB	Hypothyroidism
HESX1	Hypothyroidism
HIRA	Hypothyroidism
IGSF1	Hypothyroidism
IL17RA	Hypothyroidism
IL2RG	Hypothyroidism
IL7R	Hypothyroidism
KANSL1	Hypothyroidism
LIG4	Hypothyroidism
LRBA	Hypothyroidism
LRP4	Hypothyroidism
MT-CO1	Hypothyroidism
MT-ND1	Hypothyroidism
MT-ND6	Hypothyroidism
NAA10	Hypothyroidism
NFKB1	Hypothyroidism
NFKB2	Hypothyroidism
PRKCD	Hypothyroidism
PTCH1	Hypothyroidism
PTEN	Hypothyroidism
PTRH2	Hypothyroidism
RFC2	Hypothyroidism
SDHB	Hypothyroidism
SETBP1	Hypothyroidism
SHH	Hypothyroidism
SKI	Hypothyroidism
STAT1	Hypothyroidism
STAT3	Hypothyroidism
TBL2	Hypothyroidism
TF	Hypothyroidism
THRA	Hypothyroidism
TRAF3IP2	Hypothyroidism
TSC1	Hypothyroidism
TSC2	Hypothyroidism
UBR1	Hypothyroidism
WFS1	Hypothyroidism
GSTM1	Hypothyroidism
DCTN1	Hypoventilation
DMD	Hypoventilation

MOGS	Hypoventilation
NDN	Hypoventilation
SNRPN	Hypoventilation
CAV1	Increased pulmonary vascular resistance
SMAD9	Increased pulmonary vascular resistance
AGRN	Intermittent episodes of respiratory insufficiency due to muscle weakness
LRP4	Intermittent episodes of respiratory insufficiency due to muscle weakness
CDKL5	Intermittent hyperventilation
MECP2	Intermittent hyperventilation
TCF4	Intermittent hyperventilation
DKC1	Interstitial pneumonitis
PARN	Interstitial pneumonitis
STAT3	Interstitial pneumonitis
TINF2	Interstitial pneumonitis
FGD1	Interstitial pulmonary disease
GBA	Interstitial pulmonary disease
HLA-DRE	Interstitial pulmonary disease
ITGA3	Interstitial pulmonary disease
SCARB2	Interstitial pulmonary disease
HADHA	Long chain 3 hydroxyacyl coA dehydrogenase deficiency
MAX	Loss of voice
SDHA	Loss of voice
SDHB	Loss of voice
NPC2	Low cholesterol esterification rates
CHEK2	Lung adenocarcinoma
MDM2	Lung adenocarcinoma
TP53	Lung adenocarcinoma
FANCC	Lung segmentation defects
FANCD2	Lung segmentation defects
FANCI	Lung segmentation defects
FANCM	Lung segmentation defects
GPC3	Lung segmentation defects
GPC4	Lung segmentation defects
PALB2	Lung segmentation defects
SLX4	Lung segmentation defects
WT1	Mesothelioma
NF2	Mesothelioma
NFE2L2	Mesothelioma
NFYA	Mesothelioma
NLRP3	Mesothelioma
NME2	Mesothelioma
NOD2	Mesothelioma
NOS2	Mesothelioma
NOS3	Mesothelioma

NOTCH3	Mesothelioma
NQO1	Mesothelioma
ACTC1	Mitral regurgitation
ALDH18A	Mitral regurgitation
BAG3	Mitral regurgitation
CBL	Mitral regurgitation
CCND2	Mitral regurgitation
COL1A2	Mitral regurgitation
DES	Mitral regurgitation
DMD	Mitral regurgitation
DSG2	Mitral regurgitation
DTNA	Mitral regurgitation
ELN	Mitral regurgitation
FBLN5	Mitral regurgitation
FBN1	Mitral regurgitation
FBN2	Mitral regurgitation
FHL2	Mitral regurgitation
FLNA	Mitral regurgitation
GATAD1	Mitral regurgitation
GTF2IRD	Mitral regurgitation
LAMA4	Mitral regurgitation
LDB3	Mitral regurgitation
LMNA	Mitral regurgitation
MIB1	Mitral regurgitation
MYH6	Mitral regurgitation
MYH7B	Mitral regurgitation
PDSS1	Mitral regurgitation
PSEN1	Mitral regurgitation
PSEN2	Mitral regurgitation
RAF1	Mitral regurgitation
RFC2	Mitral regurgitation
RPS6KA3	Mitral regurgitation
SDHA	Mitral regurgitation
SMAD3	Mitral regurgitation
SMAD4	Mitral regurgitation
TAZ	Mitral regurgitation
TBL2	Mitral regurgitation
TGFB3	Mitral regurgitation
TMPO	Mitral regurgitation
TPM1	Mitral regurgitation
TTN	Mitral regurgitation
FBN1	Mitral stenosis
GBA	Mitral stenosis
KRAS	Mitral stenosis

NRAS	Mitral stenosis
PRKAR1A	Mitral stenosis
RAF1	Mitral stenosis
CALR	Myelofibrosis
GFI1B	Myelofibrosis
NBEAL2	Myelofibrosis
ARL13B	Neonatal breathing dysregulation
CEP290	Neonatal breathing dysregulation
CSPP1	Neonatal breathing dysregulation
TMEM23	Neonatal breathing dysregulation
AKT2	Neonatal hypoglycemia
CAMKMT	Neonatal hypoglycemia
CDKN1C	Neonatal hypoglycemia
DBH	Neonatal hypoglycemia
HESX1	Neonatal hypoglycemia
KDM6A	Neonatal hypoglycemia
PPM1B	Neonatal hypoglycemia
ALDH7A	Neonatal respiratory distress
CCDC8	Neonatal respiratory distress
CUL7	Neonatal respiratory distress
DNAAF2	Neonatal respiratory distress
DNAAF5	Neonatal respiratory distress
OBSL1	Neonatal respiratory distress
OFD1	Neonatal respiratory distress
PLEC	Neonatal respiratory distress
RUNX2	Neonatal respiratory distress
SNRPB	Neonatal respiratory distress
SPAG1	Neonatal respiratory distress
AKT1	Neoplasm of the lung
CHEK2	Neoplasm of the lung
EWSR1	Neoplasm of the lung
FUS	Neoplasm of the lung
MBTPS2	Neoplasm of the lung
MDM2	Neoplasm of the lung
NOTCH3	Neoplasm of the lung
PDGFRB	Neoplasm of the lung
PTEN	Neoplasm of the lung
RB1	Neoplasm of the lung
STK11	Neoplasm of the lung
TP53	Neoplasm of the lung
TP73	Neoplasm of the lung
WT1	Neoplasm of the lung
IFNGR1	Neoplasm of the lung
IGBP1	Neoplasm of the lung

IGF1	Neoplasm of the lung
IGF2	Neoplasm of the lung
IGF2R	Neoplasm of the lung
IL6ST	Neoplasm of the lung
JAG1	Neoplasm of the lung
JUN	Neoplasm of the lung
JUNB	Neoplasm of the lung
KEAP1	Neoplasm of the lung
KIF3A	Neoplasm of the lung
KLHL3	Neoplasm of the lung
KMT2A	Neoplasm of the lung
KRAS	Neoplasm of the lung
KRT19	Neoplasm of the lung
LEPR	Neoplasm of the lung
LMO2	Neoplasm of the lung
LOX	Neoplasm of the lung
LTBR	Neoplasm of the lung
MAP3K8	Neoplasm of the lung
MAPK1	Neoplasm of the lung
MAPK14	Neoplasm of the lung
MAPK3	Neoplasm of the lung
MAPT	Neoplasm of the lung
MECP2	Neoplasm of the lung
MGME1	Neoplasm of the lung
ADAR	Nephrotic syndrome
ANLN	Nephrotic syndrome
APOA1	Nephrotic syndrome
ARHGDI/	Nephrotic syndrome
ARL6	Nephrotic syndrome
CASP10	Nephrotic syndrome
CEP290	Nephrotic syndrome
COL4A3	Nephrotic syndrome
DCLRE1(	Nephrotic syndrome
FAS	Nephrotic syndrome
FGA	Nephrotic syndrome
FN1	Nephrotic syndrome
GATA3	Nephrotic syndrome
GLA	Nephrotic syndrome
IFIH1	Nephrotic syndrome
IFT27	Nephrotic syndrome
IL2RG	Nephrotic syndrome
IL7R	Nephrotic syndrome
INF2	Nephrotic syndrome
ITGA3	Nephrotic syndrome

LAMB2	Nephrotic syndrome
LIG4	Nephrotic syndrome
LZTFL1	Nephrotic syndrome
MT-CO1	Nephrotic syndrome
MT-ND1	Nephrotic syndrome
MT-ND6	Nephrotic syndrome
MYO1E	Nephrotic syndrome
NLRP3	Nephrotic syndrome
PRKCD	Nephrotic syndrome
RNASEH:	Nephrotic syndrome
SAMHD1	Nephrotic syndrome
SCARB2	Nephrotic syndrome
SERPINA	Nephrotic syndrome
SNAP29	Nephrotic syndrome
TRIM32	Nephrotic syndrome
WT1	Nephrotic syndrome
ACTA1	Nocturnal hypoventilation
COL6A1	Nocturnal hypoventilation
TTN	Nocturnal hypoventilation
DNAAF2	Obstructive lung disease
DNAAF5	Obstructive lung disease
GLA	Obstructive lung disease
OFD1	Obstructive lung disease
SPAG1	Obstructive lung disease
ICAM1	Obstructive lung disease
CREBBP	Obstructive sleep apnea
DNA2	Obstructive sleep apnea
FBN1	Obstructive sleep apnea
HRAS	Obstructive sleep apnea
SKI	Obstructive sleep apnea
TRPV4	Obstructive sleep apnea
ACTG2	Pancreatitis
AP2S1	Pancreatitis
BSCL2	Pancreatitis
CAV1	Pancreatitis
CFTR	Pancreatitis
CTLA4	Pancreatitis
DBT	Pancreatitis
FOS	Pancreatitis
GNA11	Pancreatitis
HLA-B	Pancreatitis
HLA-C	Pancreatitis
HLA-DPE	Pancreatitis
IKZF1	Pancreatitis

LMNA	Pancreatitis
MT-CO1	Pancreatitis
MT-ND1	Pancreatitis
MT-ND6	Pancreatitis
NOD2	Pancreatitis
PCCB	Pancreatitis
PPARG	Pancreatitis
PRSS1	Pancreatitis
SLC25A1	Pancreatitis
TLR4	Pancreatitis
PRRT2	Paroxysmal dyskinesia
B3GAT3	Patent foramen ovale
PHGDH	Patent foramen ovale
SKI	Patent foramen ovale
STAMBP	Patent foramen ovale
TALDO1	Patent foramen ovale
TGFB3	Patent foramen ovale
ELN	Peripheral pulmonary artery stenosis
GTF2IRD	Peripheral pulmonary artery stenosis
JAG1	Peripheral pulmonary artery stenosis
RFC2	Peripheral pulmonary artery stenosis
TBL2	Peripheral pulmonary artery stenosis
WDR35	Peripheral pulmonary artery stenosis
AMER1	Pierre-Robin sequence
COMT	Pierre-Robin sequence
EIF4A3	Pierre-Robin sequence
HIRA	Pierre-Robin sequence
ARNT2	Pituitary hypothyroidism
LEPR	Pituitary hypothyroidism
CFTR	Pleural effusion
LBR	Pleural effusion
PRSS1	Pleural effusion
BTK	Pneumonia
CASP8	Pneumonia
CD81	Pneumonia
DCLRE1(	Pneumonia
DNAAF2	Pneumonia
DNAAF5	Pneumonia
DNMT3B	Pneumonia
DPP9	Pneumonia
DSP	Pneumonia
FAM13A	Pneumonia
HDAC8	Pneumonia
HELLS	Pneumonia

IL2RG	Pneumonia
IL7R	Pneumonia
LIG4	Pneumonia
LRBA	Pneumonia
NFKB1	Pneumonia
NFKB2	Pneumonia
OFD1	Pneumonia
PARN	Pneumonia
PNP	Pneumonia
PRKCD	Pneumonia
RAD21	Pneumonia
RANBP2	Pneumonia
SMC1A	Pneumonia
SMC3	Pneumonia
SPAG1	Pneumonia
ZBTB24	Pneumonia
POR	Pneumonia
POT1	Pneumonia
POU5F1	Pneumonia
PPARG	Pneumonia
PPFIBP1	Pneumonia
PPP2CA	Pneumonia
PPP2R1B	Pneumonia
PPP2R2A	Pneumonia
PRKDC	Pneumonia
PROC	Pneumonia
PSEN1	Pneumonia
PSEN2	Pneumonia
PTEN	Pneumonia
PTGFR	Pneumonia
PTGS2	Pneumonia
PTH1R	Pneumonia
PTMA	Pneumonia
PTPN1	Pneumonia
PUF60	Pneumonia
PYCARD	Pneumonia
ACTA2	Pneumothorax
FBN1	Pneumothorax
HRAS	Pneumothorax
SMAD3	Pneumothorax
PPARG	Preeclampsia
ACVRL1	Pulmonary arteriovenous malformation
SMAD4	Pulmonary arteriovenous malformation
ACTA2	Pulmonary artery aneurysm

EFEMP2	Pulmonary artery aneurysm
FBLN5	Pulmonary artery aneurysm
FBN1	Pulmonary artery aneurysm
SMAD3	Pulmonary artery aneurysm
CRELD1	Pulmonary artery atresia
FADD	Pulmonary artery atresia
SHH	Pulmonary artery atresia
EFEMP2	Pulmonary artery dilatation
FBLN5	Pulmonary artery dilatation
FBN1	Pulmonary artery dilatation
ACTC1	Pulmonary artery hypoplasia
BAG3	Pulmonary artery hypoplasia
DES	Pulmonary artery hypoplasia
DMD	Pulmonary artery hypoplasia
DSG2	Pulmonary artery hypoplasia
DTNA	Pulmonary artery hypoplasia
FHL2	Pulmonary artery hypoplasia
GATAD1	Pulmonary artery hypoplasia
LAMA4	Pulmonary artery hypoplasia
LDB3	Pulmonary artery hypoplasia
LMNA	Pulmonary artery hypoplasia
MIB1	Pulmonary artery hypoplasia
MYH6	Pulmonary artery hypoplasia
MYH7B	Pulmonary artery hypoplasia
PSEN1	Pulmonary artery hypoplasia
PSEN2	Pulmonary artery hypoplasia
RAF1	Pulmonary artery hypoplasia
SDHA	Pulmonary artery hypoplasia
TAZ	Pulmonary artery hypoplasia
TMPO	Pulmonary artery hypoplasia
TPM1	Pulmonary artery hypoplasia
TTN	Pulmonary artery hypoplasia
ZEB2	Pulmonary artery sling
DOCK6	Pulmonary artery stenosis
ELN	Pulmonary artery stenosis
NAA10	Pulmonary artery stenosis
NOTCH1	Pulmonary artery stenosis
RBPJ	Pulmonary artery stenosis
ZEB2	Pulmonary artery stenosis
VHL	Pulmonary capillary hemangiomatosis
SERPINC	Pulmonary edema
ACVRL1	Pulmonary embolism
AGGF1	Pulmonary embolism
AKAP9	Pulmonary embolism

AKT1	Pulmonary embolism
ANK2	Pulmonary embolism
CALM1	Pulmonary embolism
CALM2	Pulmonary embolism
CALM3	Pulmonary embolism
CTLA4	Pulmonary embolism
GNAQ	Pulmonary embolism
HLA-B	Pulmonary embolism
HLA-C	Pulmonary embolism
HLA-DPE	Pulmonary embolism
KCNQ1	Pulmonary embolism
MT-CO1	Pulmonary embolism
MT-ND1	Pulmonary embolism
MT-ND6	Pulmonary embolism
NOD2	Pulmonary embolism
NOS1AP	Pulmonary embolism
PROC	Pulmonary embolism
PROS1	Pulmonary embolism
PTEN	Pulmonary embolism
SERPINC	Pulmonary embolism
SMAD4	Pulmonary embolism
TLR4	Pulmonary embolism
SIRT1	Pulmonary embolism
SKI	Pulmonary embolism
SKIL	Pulmonary embolism
SLC11A2	Pulmonary embolism
TWIST1	Pulmonary embolism
TXN	Pulmonary embolism
SLC2A1	Pulmonary emphysema
SLC31A1	Pulmonary emphysema
AP3B1	Pulmonary fibrosis
CAV1	Pulmonary fibrosis
CCNL2	Pulmonary fibrosis
CFTR	Pulmonary fibrosis
CTLA4	Pulmonary fibrosis
DCTN4	Pulmonary fibrosis
DKC1	Pulmonary fibrosis
DPP9	Pulmonary fibrosis
DSP	Pulmonary fibrosis
FAM13A	Pulmonary fibrosis
HLA-DPE	Pulmonary fibrosis
HLA-DRE	Pulmonary fibrosis
HPS1	Pulmonary fibrosis
PARN	Pulmonary fibrosis

TINF2	Pulmonary fibrosis
SLC4A1	Pulmonary fibrosis
SMAD2	Pulmonary fibrosis
SMAD3	Pulmonary fibrosis
SMAD7	Pulmonary fibrosis
SMAD9	Pulmonary fibrosis
SMARCC	Pulmonary fibrosis
SOD2	Pulmonary fibrosis
SOX2	Pulmonary fibrosis
SPP1	Pulmonary fibrosis
SPRY2	Pulmonary fibrosis
STAT3	Pulmonary fibrosis
STAT5A	Pulmonary fibrosis
STAT6	Pulmonary fibrosis
STK11	Pulmonary fibrosis
ACTA2	Pulmonary hypertension
ACVRL1	Pulmonary hypertension
BANF1	Pulmonary hypertension
BSCL2	Pulmonary hypertension
CAV1	Pulmonary hypertension
CCNL2	Pulmonary hypertension
CLCN7	Pulmonary hypertension
COL1A2	Pulmonary hypertension
DOCK6	Pulmonary hypertension
DPP9	Pulmonary hypertension
DSP	Pulmonary hypertension
FAM13A	Pulmonary hypertension
FBN1	Pulmonary hypertension
FGFR3	Pulmonary hypertension
FKBP10	Pulmonary hypertension
FLNA	Pulmonary hypertension
FOS	Pulmonary hypertension
FOXF1	Pulmonary hypertension
GBA	Pulmonary hypertension
HBB	Pulmonary hypertension
HCCS	Pulmonary hypertension
HLA-B	Pulmonary hypertension
HLA-C	Pulmonary hypertension
HLA-DRI	Pulmonary hypertension
KRT18	Pulmonary hypertension
KRT8	Pulmonary hypertension
MLX	Pulmonary hypertension
MT-CO1	Pulmonary hypertension
MT-ND1	Pulmonary hypertension

MT-ND6	Pulmonary hypertension
NDUFB11	Pulmonary hypertension
NOD2	Pulmonary hypertension
NOTCH1	Pulmonary hypertension
PAM16	Pulmonary hypertension
PARN	Pulmonary hypertension
PDSS1	Pulmonary hypertension
PPARG	Pulmonary hypertension
PPIB	Pulmonary hypertension
RBPJ	Pulmonary hypertension
SCARB2	Pulmonary hypertension
SERPINH	Pulmonary hypertension
SMAD4	Pulmonary hypertension
SMAD9	Pulmonary hypertension
FN1	Pulmonary hypertension
FOXA2	Pulmonary hypertension
FOXM1	Pulmonary hypertension
GCLM	Pulmonary hypertension
GDF15	Pulmonary hypertension
GNAQ	Pulmonary hypertension
GNAS	Pulmonary hypertension
AGTR1	Pulmonary hypoplasia
ARL13B	Pulmonary hypoplasia
BCOR	Pulmonary hypoplasia
CEP290	Pulmonary hypoplasia
CSPP1	Pulmonary hypoplasia
DHCR7	Pulmonary hypoplasia
ETFA	Pulmonary hypoplasia
ETFB	Pulmonary hypoplasia
ETFDH	Pulmonary hypoplasia
GRIP1	Pulmonary hypoplasia
ITGA8	Pulmonary hypoplasia
LBR	Pulmonary hypoplasia
LMNA	Pulmonary hypoplasia
NAA10	Pulmonary hypoplasia
NSDHL	Pulmonary hypoplasia
PEX1	Pulmonary hypoplasia
PEX14	Pulmonary hypoplasia
PEX19	Pulmonary hypoplasia
PEX2	Pulmonary hypoplasia
PEX5	Pulmonary hypoplasia
PHGDH	Pulmonary hypoplasia
RPGRIP1	Pulmonary hypoplasia
SHH	Pulmonary hypoplasia

TMEM23	Pulmonary hypoplasia
WDR35	Pulmonary hypoplasia
APOE	Pulmonary infiltrates
BIRC3	Pulmonary infiltrates
CAV1	Pulmonary infiltrates
CCNL2	Pulmonary infiltrates
CTLA4	Pulmonary infiltrates
GBA	Pulmonary infiltrates
HLA-B	Pulmonary infiltrates
HLA-C	Pulmonary infiltrates
HLA-DPE	Pulmonary infiltrates
HLA-DRI	Pulmonary infiltrates
NOD2	Pulmonary infiltrates
PDGFRA	Pulmonary infiltrates
SCARB2	Pulmonary infiltrates
TLR4	Pulmonary infiltrates
TSC1	Pulmonary infiltrates
TSC2	Pulmonary infiltrates
COL1A2	Pulmonary insufficiency
EFEMP2	Pulmonary insufficiency
FBLN5	Pulmonary insufficiency
FOXF1	Pulmonary insufficiency
PPIB	Pulmonary insufficiency
TSC1	Pulmonary lymphangiomyomatosis
TSC2	Pulmonary lymphangiomyomatosis
BCOR	Pulmonic stenosis
CCDC22	Pulmonic stenosis
DAG1	Pulmonic stenosis
DOCK6	Pulmonic stenosis
ELN	Pulmonic stenosis
FBN1	Pulmonic stenosis
GATA4	Pulmonic stenosis
GPC3	Pulmonic stenosis
GPC4	Pulmonic stenosis
GTF2IRD	Pulmonic stenosis
HRAS	Pulmonic stenosis
KANSL1	Pulmonic stenosis
KDM6A	Pulmonic stenosis
KRAS	Pulmonic stenosis
MAP2K2	Pulmonic stenosis
NAA10	Pulmonic stenosis
NOTCH1	Pulmonic stenosis
NRAS	Pulmonic stenosis
POMGNT	Pulmonic stenosis

POMK	Pulmonic stenosis
RAF1	Pulmonic stenosis
RBPJ	Pulmonic stenosis
RFC2	Pulmonic stenosis
SHH	Pulmonic stenosis
SKIV2L	Pulmonic stenosis
SMAD3	Pulmonic stenosis
TBL2	Pulmonic stenosis
ZEB2	Pulmonic stenosis
NCF1	Recurrent Aspergillus infections
NCF2	Recurrent Aspergillus infections
GBA	Recurrent aspiration pneumonia
KDM6A	Recurrent aspiration pneumonia
YWHAE	Recurrent aspiration pneumonia
ADAM17	Recurrent bronchiolitis
EGFR	Recurrent bronchiolitis
CD81	Recurrent bronchitis
DNAAF2	Recurrent bronchitis
DNAAF5	Recurrent bronchitis
GNPTAB	Recurrent bronchitis
IGHG1	Recurrent bronchitis
IL2RG	Recurrent bronchitis
LRBA	Recurrent bronchitis
NFKB1	Recurrent bronchitis
NFKB2	Recurrent bronchitis
OFD1	Recurrent bronchitis
PRKCD	Recurrent bronchitis
SPAG1	Recurrent bronchitis
TAPBP	Recurrent bronchitis
TCF3	Recurrent bronchitis
CFTR	Recurrent bronchopulmonary infections
DCTN4	Recurrent bronchopulmonary infections
NCF1	Recurrent Burkholderia cepacia infections
NCF2	Recurrent Burkholderia cepacia infections
CIITA	Recurrent lower respiratory tract infections
COL6A1	Recurrent lower respiratory tract infections
PNP	Recurrent lower respiratory tract infections
PRKDC	Recurrent lower respiratory tract infections
RFX5	Recurrent lower respiratory tract infections
RFXANK	Recurrent lower respiratory tract infections
DOCK8	Recurrent mycobacterial infections
IFNGR1	Recurrent mycobacterial infections
ISG15	Recurrent mycobacterial infections
STAT1	Recurrent mycobacterial infections

TYK2	Recurrent mycobacterial infections
GATA2	Recurrent mycobacterium avium complex infections
ADAM17	Recurrent pneumonia
CD81	Recurrent pneumonia
CDC6	Recurrent pneumonia
CFTR	Recurrent pneumonia
DCTN4	Recurrent pneumonia
DNAAF2	Recurrent pneumonia
DNAAF5	Recurrent pneumonia
EGFR	Recurrent pneumonia
GNPTAB	Recurrent pneumonia
IGHG1	Recurrent pneumonia
LRBA	Recurrent pneumonia
NCF1	Recurrent pneumonia
NCF2	Recurrent pneumonia
NFKB1	Recurrent pneumonia
NFKB2	Recurrent pneumonia
OFD1	Recurrent pneumonia
ORC1	Recurrent pneumonia
PEPD	Recurrent pneumonia
PLOD1	Recurrent pneumonia
PRKCD	Recurrent pneumonia
SPAG1	Recurrent pneumonia
TCF3	Recurrent pneumonia
WDR35	Recurrent pneumonia
NCF1	Recurrent Serratia marcescens infections
NCF2	Recurrent Serratia marcescens infections
GFAP	Recurrent singultus
GLDC	Recurrent singultus
CASP8	Recurrent sinopulmonary infections
CD81	Recurrent sinopulmonary infections
DNAAF2	Recurrent sinopulmonary infections
DNAAF5	Recurrent sinopulmonary infections
DOCK8	Recurrent sinopulmonary infections
LRBA	Recurrent sinopulmonary infections
NFKB1	Recurrent sinopulmonary infections
NFKB2	Recurrent sinopulmonary infections
OFD1	Recurrent sinopulmonary infections
PRKCD	Recurrent sinopulmonary infections
SPAG1	Recurrent sinopulmonary infections
STAT3	Recurrent sinopulmonary infections
CIITA	Recurrent upper respiratory tract infections
CREBBP	Recurrent upper respiratory tract infections
CTLA4	Recurrent upper respiratory tract infections

DCLRE1C	Recurrent upper respiratory tract infections
GLB1	Recurrent upper respiratory tract infections
OFD1	Recurrent upper respiratory tract infections
PNP	Recurrent upper respiratory tract infections
PRPS1	Recurrent upper respiratory tract infections
RFX5	Recurrent upper respiratory tract infections
RFXANK	Recurrent upper respiratory tract infections
ACTA1	Reduced vital capacity
TPM3	Reduced vital capacity
TTN	Reduced vital capacity
DECR1	Respiratory acidosis
ITGA3	Respiratory acidosis
ASL	Respiratory alkalosis
ASS1	Respiratory alkalosis
CASK	Respiratory difficulties
CDKL5	Respiratory difficulties
COX6B1	Respiratory difficulties
CREBBP	Respiratory difficulties
EDA	Respiratory difficulties
EFTUD2	Respiratory difficulties
MT-CO1	Respiratory difficulties
NECAP1	Respiratory difficulties
PLCB1	Respiratory difficulties
RPL11	Respiratory difficulties
RPL15	Respiratory difficulties
RPL26	Respiratory difficulties
RPL5	Respiratory difficulties
RPS10	Respiratory difficulties
RPS19	Respiratory difficulties
RPS24	Respiratory difficulties
RPS29	Respiratory difficulties
RPS7	Respiratory difficulties
SCO2	Respiratory difficulties
SPTAN1	Respiratory difficulties
STT3B	Respiratory difficulties
STXBP1	Respiratory difficulties
WWOX	Respiratory difficulties
ACTA1	Respiratory distress
AGRN	Respiratory distress
ALDH7A	Respiratory distress
ARL6	Respiratory distress
CDC6	Respiratory distress
CEP290	Respiratory distress
DNAAF2	Respiratory distress

DNAAF5	Respiratory distress
ETFA	Respiratory distress
ETFB	Respiratory distress
ETFDH	Respiratory distress
GBA	Respiratory distress
IFT27	Respiratory distress
ITGA3	Respiratory distress
ITGA7	Respiratory distress
LRP4	Respiratory distress
LZTFL1	Respiratory distress
NAGS	Respiratory distress
OFD1	Respiratory distress
ORC1	Respiratory distress
SLC25A1	Respiratory distress
SPAG1	Respiratory distress
TPM3	Respiratory distress
TRIM32	Respiratory distress
TF	Respiratory distress
TFRC	Respiratory distress
BCS1L	Respiratory failure
CTSD	Respiratory failure
CYC1	Respiratory failure
DAG1	Respiratory failure
DMD	Respiratory failure
ECHS1	Respiratory failure
ERBB3	Respiratory failure
EXOSC3	Respiratory failure
EXOSC8	Respiratory failure
FLNA	Respiratory failure
FOXRED	Respiratory failure
HADHA	Respiratory failure
HADHB	Respiratory failure
HRAS	Respiratory failure
MCM4	Respiratory failure
MT-ND1	Respiratory failure
MT-ND6	Respiratory failure
NDUFA1	Respiratory failure
NDUFA1(	Respiratory failure
NDUFA1	Respiratory failure
NDUFA1:	Respiratory failure
NDUFAF	Respiratory failure
NDUFAF:	Respiratory failure
NDUFAF:	Respiratory failure
NDUFAF	Respiratory failure

NDUFB9	Respiratory failure
NDUFS1	Respiratory failure
NDUFS2	Respiratory failure
NDUFS3	Respiratory failure
NDUFS6	Respiratory failure
NDUFS7	Respiratory failure
NDUFS8	Respiratory failure
NDUFV2	Respiratory failure
NPC2	Respiratory failure
PDHA1	Respiratory failure
POMGNT	Respiratory failure
POMK	Respiratory failure
PTEN	Respiratory failure
RARS2	Respiratory failure
SCO2	Respiratory failure
SDHA	Respiratory failure
SMAD4	Respiratory failure
SMN1	Respiratory failure
SMN2	Respiratory failure
SURF1	Respiratory failure
TRPV4	Respiratory failure
TSEN54	Respiratory failure
TTC19	Respiratory failure
UQCC2	Respiratory failure
UQCRC2	Respiratory failure
UQCRQ	Respiratory failure
ACTA1	Respiratory insufficiency
AGGF1	Respiratory insufficiency
AGRN	Respiratory insufficiency
AGTR1	Respiratory insufficiency
ARL13B	Respiratory insufficiency
BAG3	Respiratory insufficiency
BCS1L	Respiratory insufficiency
BIN1	Respiratory insufficiency
CAV1	Respiratory insufficiency
CCNL2	Respiratory insufficiency
CDC6	Respiratory insufficiency
CDKL5	Respiratory insufficiency
CNTNAP	Respiratory insufficiency
COL1A2	Respiratory insufficiency
COL5A1	Respiratory insufficiency
COL6A1	Respiratory insufficiency
CRLF1	Respiratory insufficiency
CSPP1	Respiratory insufficiency

CTLA4	Respiratory insufficiency
CTSD	Respiratory insufficiency
DAG1	Respiratory insufficiency
DCTN1	Respiratory insufficiency
DNAAF2	Respiratory insufficiency
DNAAF5	Respiratory insufficiency
DNM2	Respiratory insufficiency
DST	Respiratory insufficiency
ECHS1	Respiratory insufficiency
EFEMP2	Respiratory insufficiency
EHMT1	Respiratory insufficiency
EIF4A3	Respiratory insufficiency
EMD	Respiratory insufficiency
EPOR	Respiratory insufficiency
EXOSC3	Respiratory insufficiency
EXOSC8	Respiratory insufficiency
FBLN5	Respiratory insufficiency
FBN1	Respiratory insufficiency
FGFR3	Respiratory insufficiency
FHL1	Respiratory insufficiency
FKBP10	Respiratory insufficiency
FLNA	Respiratory insufficiency
FLNB	Respiratory insufficiency
FOXF1	Respiratory insufficiency
FOXRED	Respiratory insufficiency
GATA2	Respiratory insufficiency
GBA	Respiratory insufficiency
GFAP	Respiratory insufficiency
GLA	Respiratory insufficiency
GLUL	Respiratory insufficiency
HBB	Respiratory insufficiency
HCCS	Respiratory insufficiency
HLA-B	Respiratory insufficiency
HLA-C	Respiratory insufficiency
HLA-DPB	Respiratory insufficiency
HLA-DRB	Respiratory insufficiency
IDH1	Respiratory insufficiency
IKZF1	Respiratory insufficiency
ITGA7	Respiratory insufficiency
KLHL41	Respiratory insufficiency
KRT14	Respiratory insufficiency
KRT17	Respiratory insufficiency
KRT5	Respiratory insufficiency
KRT6A	Respiratory insufficiency

LAMA3	Respiratory insufficiency
LAMB2	Respiratory insufficiency
LBR	Respiratory insufficiency
LIG4	Respiratory insufficiency
LMNA	Respiratory insufficiency
LRP4	Respiratory insufficiency
MCCC2	Respiratory insufficiency
MECP2	Respiratory insufficiency
MT-CO1	Respiratory insufficiency
MT-ND1	Respiratory insufficiency
MT-ND6	Respiratory insufficiency
MYBPC1	Respiratory insufficiency
NDUFA1(	Respiratory insufficiency
NDUFA1	Respiratory insufficiency
NDUFAF	Respiratory insufficiency
NDUFB1	Respiratory insufficiency
NDUFS1	Respiratory insufficiency
NDUFS2	Respiratory insufficiency
NDUFS3	Respiratory insufficiency
NDUFS7	Respiratory insufficiency
NDUFS8	Respiratory insufficiency
NDUFV2	Respiratory insufficiency
NOD2	Respiratory insufficiency
OCRL	Respiratory insufficiency
OFD1	Respiratory insufficiency
ORC1	Respiratory insufficiency
PDHA1	Respiratory insufficiency
PEX1	Respiratory insufficiency
PEX14	Respiratory insufficiency
PEX19	Respiratory insufficiency
PEX2	Respiratory insufficiency
PEX5	Respiratory insufficiency
PEX7	Respiratory insufficiency
PIP5K1C	Respiratory insufficiency
PLOD1	Respiratory insufficiency
PLOD2	Respiratory insufficiency
POMGNT	Respiratory insufficiency
POMK	Respiratory insufficiency
PPIB	Respiratory insufficiency
PRKCSH	Respiratory insufficiency
PRPS1	Respiratory insufficiency
PURA	Respiratory insufficiency
RARS2	Respiratory insufficiency
RFT1	Respiratory insufficiency

SCO2	Respiratory insufficiency
SDHA	Respiratory insufficiency
SHH	Respiratory insufficiency
SLC25A1	Respiratory insufficiency
SLC25A3	Respiratory insufficiency
SLC25A4	Respiratory insufficiency
SMAD4	Respiratory insufficiency
SPAG1	Respiratory insufficiency
SURF1	Respiratory insufficiency
SYNE1	Respiratory insufficiency
SYNE2	Respiratory insufficiency
TCOF1	Respiratory insufficiency
TMEM23	Respiratory insufficiency
TMEM43	Respiratory insufficiency
TMEM70	Respiratory insufficiency
TNNT1	Respiratory insufficiency
TPM3	Respiratory insufficiency
TSC1	Respiratory insufficiency
TSC2	Respiratory insufficiency
TSEN54	Respiratory insufficiency
TTN	Respiratory insufficiency
WDR35	Respiratory insufficiency
WFS1	Respiratory insufficiency
SYK	Respiratory insufficiency
TBKBP1	Respiratory insufficiency
TBXA2R	Respiratory insufficiency
TGFA	Respiratory insufficiency
DNAAF2	Respiratory insufficiency due to defective ciliary clearance
DNAAF5	Respiratory insufficiency due to defective ciliary clearance
OFD1	Respiratory insufficiency due to defective ciliary clearance
SPAG1	Respiratory insufficiency due to defective ciliary clearance
ACTA1	Respiratory insufficiency due to muscle weakness
AGRN	Respiratory insufficiency due to muscle weakness
AIFM1	Respiratory insufficiency due to muscle weakness
ATXN2	Respiratory insufficiency due to muscle weakness
BIN1	Respiratory insufficiency due to muscle weakness
CHMP2B	Respiratory insufficiency due to muscle weakness
COL6A1	Respiratory insufficiency due to muscle weakness
COX6B1	Respiratory insufficiency due to muscle weakness
DAG1	Respiratory insufficiency due to muscle weakness
DCTN1	Respiratory insufficiency due to muscle weakness
DES	Respiratory insufficiency due to muscle weakness
ERBB4	Respiratory insufficiency due to muscle weakness
FHL1	Respiratory insufficiency due to muscle weakness

FUS	Respiratory insufficiency due to muscle weakness
HNRNPA	Respiratory insufficiency due to muscle weakness
ITGA7	Respiratory insufficiency due to muscle weakness
LAMB2	Respiratory insufficiency due to muscle weakness
LMNA	Respiratory insufficiency due to muscle weakness
LRP4	Respiratory insufficiency due to muscle weakness
MATR3	Respiratory insufficiency due to muscle weakness
MT-CO1	Respiratory insufficiency due to muscle weakness
OPTN	Respiratory insufficiency due to muscle weakness
PFN1	Respiratory insufficiency due to muscle weakness
POMGNT	Respiratory insufficiency due to muscle weakness
POMK	Respiratory insufficiency due to muscle weakness
PON2	Respiratory insufficiency due to muscle weakness
SIGMAR1	Respiratory insufficiency due to muscle weakness
SLC25A1	Respiratory insufficiency due to muscle weakness
SQSTM1	Respiratory insufficiency due to muscle weakness
SUCLA2	Respiratory insufficiency due to muscle weakness
TARDBP	Respiratory insufficiency due to muscle weakness
TBK1	Respiratory insufficiency due to muscle weakness
TNPO3	Respiratory insufficiency due to muscle weakness
TPM3	Respiratory insufficiency due to muscle weakness
TTN	Respiratory insufficiency due to muscle weakness
UBA1	Respiratory insufficiency due to muscle weakness
UNC13A	Respiratory insufficiency due to muscle weakness
VAPB	Respiratory insufficiency due to muscle weakness
VCP	Respiratory insufficiency due to muscle weakness
IKBKB	Respiratory tract infection
PEX1	Respiratory tract infection
PEX14	Respiratory tract infection
PEX19	Respiratory tract infection
PEX2	Respiratory tract infection
PEX5	Respiratory tract infection
CD81	Restrictive lung disease
CTLA4	Restrictive lung disease
DKC1	Restrictive lung disease
FLNB	Restrictive lung disease
GBA	Restrictive lung disease
GLB1	Restrictive lung disease
HLA-B	Restrictive lung disease
HLA-C	Restrictive lung disease
HLA-DPB	Restrictive lung disease
HPS1	Restrictive lung disease
IKZF1	Restrictive lung disease
LRBA	Restrictive lung disease

NFKB1	Restrictive lung disease
NFKB2	Restrictive lung disease
NLRP3	Restrictive lung disease
PARN	Restrictive lung disease
PRKCD	Restrictive lung disease
SCARB2	Restrictive lung disease
TINF2	Restrictive lung disease
TSC1	Restrictive lung disease
TSC2	Restrictive lung disease
ACTA1	Restrictive respiratory insufficiency
FRG1	Restrictive respiratory insufficiency
POMGNT	Restrictive respiratory insufficiency
TTN	Restrictive respiratory insufficiency
TIMP3	Silicosis
TINF2	Silicosis
COL5A1	Spontaneous pneumothorax
PEPD	Systemic lupus erythematosus
PRKCD	Systemic lupus erythematosus
ACADVL	Tachypnea
ACTA2	Tachypnea
ARL13B	Tachypnea
CEP290	Tachypnea
CSPP1	Tachypnea
LRPPRC	Tachypnea
PAM16	Tachypnea
PCCB	Tachypnea
TMEM23	Tachypnea
CANT1	Thoracic hypoplasia
GBA	Thoracic hypoplasia
SNRPB	Thoracic hypoplasia
WDR35	Thoracic hypoplasia
DHCR24	Total anomalous pulmonary venous return
AFF4	Tracheal stenosis
DHCR7	Tracheal stenosis
EBP	Tracheal stenosis
FBN1	Tracheal stenosis
FLNA	Tracheal stenosis
FLNB	Tracheal stenosis
GRIP1	Tracheal stenosis
HYLS1	Tracheal stenosis
PCNT	Tracheal stenosis
EHMT1	Tracheobronchomalacia
COL7A1	Tracheoesophageal fistula
DKC1	Tracheoesophageal fistula

ELN	Tracheoesophageal fistula
FANCC	Tracheoesophageal fistula
FANCD2	Tracheoesophageal fistula
FANCI	Tracheoesophageal fistula
FANCM	Tracheoesophageal fistula
FBN2	Tracheoesophageal fistula
FOXF1	Tracheoesophageal fistula
GTF2IRD	Tracheoesophageal fistula
HESX1	Tracheoesophageal fistula
ITGA8	Tracheoesophageal fistula
LAMA3	Tracheoesophageal fistula
MYCN	Tracheoesophageal fistula
NCF1	Tracheoesophageal fistula
NCF2	Tracheoesophageal fistula
NOTCH3	Tracheoesophageal fistula
PALB2	Tracheoesophageal fistula
PARN	Tracheoesophageal fistula
PDGFRB	Tracheoesophageal fistula
RFC2	Tracheoesophageal fistula
SLX4	Tracheoesophageal fistula
SOX2	Tracheoesophageal fistula
TBL2	Tracheoesophageal fistula
TCOF1	Tracheoesophageal fistula
TINF2	Tracheoesophageal fistula
AMER1	Tracheomalacia
CDC6	Tracheomalacia
FLNB	Tracheomalacia
HDAC4	Tracheomalacia
HRAS	Tracheomalacia
KIF22	Tracheomalacia
ORC1	Tracheomalacia
RAB3GA	Tracheomalacia
SNRPB	Tracheomalacia
MBTPS2	Unilateral chest hypoplasia
COMT	Unilateral primary pulmonary dysgenesis
HIRA	Unilateral primary pulmonary dysgenesis
MAX	Vagal paraganglioma
SDHA	Vagal paraganglioma
SDHB	Vagal paraganglioma
ACTC1	Ventricular septal defect
AMER1	Ventricular septal defect
ANKRD1	Ventricular septal defect
ARID1A	Ventricular septal defect
ARID1B	Ventricular septal defect

BCOR	Ventricular septal defect
BCR	Ventricular septal defect
BUB1	Ventricular septal defect
BUB1B	Ventricular septal defect
BUB3	Ventricular septal defect
CANT1	Ventricular septal defect
CCDC22	Ventricular septal defect
CCND2	Ventricular septal defect
CD96	Ventricular septal defect
CEP290	Ventricular septal defect
CEP57	Ventricular septal defect
CHRM3	Ventricular septal defect
COMT	Ventricular septal defect
CREBBP	Ventricular septal defect
CRKL	Ventricular septal defect
CSPP1	Ventricular septal defect
DHCR7	Ventricular septal defect
DOCK6	Ventricular septal defect
DTNA	Ventricular septal defect
ECHS1	Ventricular septal defect
EFTUD2	Ventricular septal defect
EHMT1	Ventricular septal defect
ELN	Ventricular septal defect
ERBB3	Ventricular septal defect
ERCC3	Ventricular septal defect
ESCO2	Ventricular septal defect
FADD	Ventricular septal defect
FANCC	Ventricular septal defect
FANCD2	Ventricular septal defect
FANCI	Ventricular septal defect
FANCM	Ventricular septal defect
FBN1	Ventricular septal defect
FBN2	Ventricular septal defect
FLNB	Ventricular septal defect
FOXC2	Ventricular septal defect
FOXF1	Ventricular septal defect
FOXRED	Ventricular septal defect
GATA3	Ventricular septal defect
GATA4	Ventricular septal defect
GPC3	Ventricular septal defect
GPC4	Ventricular septal defect
GTF2H5	Ventricular septal defect
GTF2IRD	Ventricular septal defect
HCCS	Ventricular septal defect

HDAC8	Ventricular septal defect
HIRA	Ventricular septal defect
HRAS	Ventricular septal defect
HYLS1	Ventricular septal defect
IGBP1	Ventricular septal defect
JAG1	Ventricular septal defect
KANSL1	Ventricular septal defect
KDM6A	Ventricular septal defect
KRAS	Ventricular septal defect
LBR	Ventricular septal defect
LDB3	Ventricular septal defect
LETM1	Ventricular septal defect
LMNA	Ventricular septal defect
LONP1	Ventricular septal defect
MAPK1	Ventricular septal defect
MED12	Ventricular septal defect
MEIS2	Ventricular septal defect
MIB1	Ventricular septal defect
MYH7B	Ventricular septal defect
NAA10	Ventricular septal defect
NDUFA1[illegible]	Ventricular septal defect
NDUFA1[illegible]	Ventricular septal defect
NDUFAF[illegible]	Ventricular septal defect
NDUFB1[illegible]	Ventricular septal defect
NDUFS1	Ventricular septal defect
NDUFS2	Ventricular septal defect
NDUFS3	Ventricular septal defect
NDUFS7	Ventricular septal defect
NDUFS8	Ventricular septal defect
NDUFV2	Ventricular septal defect
NOTCH1	Ventricular septal defect
NOTCH3	Ventricular septal defect
NR2F2	Ventricular septal defect
NRAS	Ventricular septal defect
NSDHL	Ventricular septal defect
OFD1	Ventricular septal defect
PALB2	Ventricular septal defect
PDE6D	Ventricular septal defect
PDHA1	Ventricular septal defect
PEX1	Ventricular septal defect
PEX14	Ventricular septal defect
PEX19	Ventricular septal defect
PEX2	Ventricular septal defect
PEX5	Ventricular septal defect

PHGDH	Ventricular septal defect
PQBP1	Ventricular septal defect
RAD21	Ventricular septal defect
RAF1	Ventricular septal defect
RBPJ	Ventricular septal defect
RFC2	Ventricular septal defect
ROR2	Ventricular septal defect
RPGRIP1	Ventricular septal defect
RPL11	Ventricular septal defect
RPL15	Ventricular septal defect
RPL26	Ventricular septal defect
RPL5	Ventricular septal defect
RPS10	Ventricular septal defect
RPS19	Ventricular septal defect
RPS24	Ventricular septal defect
RPS29	Ventricular septal defect
RPS7	Ventricular septal defect
SDHA	Ventricular septal defect
SHANK3	Ventricular septal defect
SHH	Ventricular septal defect
SKI	Ventricular septal defect
SKIV2L	Ventricular septal defect
SLX4	Ventricular septal defect
SMARCA	Ventricular septal defect
SMARCB	Ventricular septal defect
SMARCE	Ventricular septal defect
SMC1A	Ventricular septal defect
SMC3	Ventricular septal defect
SMN1	Ventricular septal defect
SMN2	Ventricular septal defect
SNRPB	Ventricular septal defect
SOX2	Ventricular septal defect
STAMBP	Ventricular septal defect
SURF1	Ventricular septal defect
TALDO1	Ventricular septal defect
TAZ	Ventricular septal defect
TBL2	Ventricular septal defect
TBX3	Ventricular septal defect
TCTN3	Ventricular septal defect
TGFB3	Ventricular septal defect
TMEM23	Ventricular septal defect
TPM1	Ventricular septal defect
UBR1	Ventricular septal defect
UMPS	Ventricular septal defect

UPF3B	Ventricular septal defect
WDR35	Ventricular septal defect
WT1	Ventricular septal defect
XYLT2	Ventricular septal defect
ZEB2	Ventricular septal defect
ACTA1	Weak cry
AGRN	Weak cry
COQ9	Weak cry
HDAC8	Weak cry
ITGA7	Weak cry
LAMA3	Weak cry
LAMB2	Weak cry
LRP4	Weak cry
RAD21	Weak cry
SLC25A1	Weak cry
SMC1A	Weak cry
SMC3	Weak cry
TPM3	Weak cry
DCTN1	Weak voice
TRIM37	Weak voice
ACAD9	COVID19
AKAP9	COVID19
AP3B1	COVID19
BCS1L	COVID19
COL6A1	COVID19
COMT	COVID19
CYB5R3	COVID19
DNAJC19	COVID19
ETFA	COVID19
EXOSC3	COVID19
EXOSC8	COVID19
FBLN5	COVID19
FBN1	COVID19
FBN2	COVID19
FKBP10	COVID19
GDF15	COVID19
GIGYF2	COVID19
GLA	COVID19
GPX1	COVID19
IL17RA	COVID19
LOX	COVID19
MIB1	COVID19
MOGS	COVID19
NDUFAF	COVID19

NDUFAF[illegible]	COVID19
NDUFB9	COVID19
NEU1	COVID19
NIN	COVID19
NPC2	COVID19
PCNT	COVID19
PLOD2	COVID19
POR	COVID19
SIGMAR[illegible]	COVID19
TBK1	COVID19
TBKBP1	COVID19
WFS1	COVID19
