# Supplementary material for: Lung disease network reveals impact of comorbidity on SARS-CoV-2 infection and opportunities of drug repurposing
Source: BMC Med Genomics. 2021 Sep 17;14:226. doi: 10.1186/s12920-021-01079-7 (PMC8447809; doi:10.1186/s12920-021-01079-7)
Supplement: Supplementary file 5 — Additional file 5. Table S4. Network-based separation measure between disease modules and statistical significance. [file 12920_2021_1079_MOESM5_ESM.pdf]

Supplementary Table 4: network-based separation measure between disease modules and statistical significance

S.No.	Lung Disease	S_{ab}	z-score
1	Hemolytic-uremic syndrome	-0.2142037	-3.47595248
2	Abnormal respiratory motile cilium morphology	-0.21138124	-4.032989708
3	Absent respiratory ciliary axoneme radial spokes	-0.21138124	-4.249361639
4	Respiratory insufficiency due to defective ciliary clearance	-0.21138124	-4.2529104
5	Obstructive lung disease	-0.21022412	-4.301392465
6	Pleural effusion	-0.18215887	-3.264072743
7	Patent foramen ovale	-0.16189869	-3.490774621
8	Pulmonary insufficiency	-0.15693685	-3.090793756
9	Agenesis of pulmonary vessels	-0.15431593	-3.252383093
10	Thoracic hypoplasia	-0.15364412	-3.63447548
11	Tracheal stenosis	-0.14914623	-2.952889882
12	Chronic bronchitis	-0.146243	-3.675008088
13	Atelectasis	-0.14545002	-3.440012864
14	Bell-shaped thorax	-0.14320859	-4.175606731
15	Chronic obstructive pulmonary disease	-0.14190413	-5.065724397
16	Pulmonary artery atresia	-0.14121352	-2.740762168
17	Hyperventilation	-0.1411619	-3.729617014
18	Diaphragmatic eventration	-0.13980694	-2.528378179
19	Neonatal respiratory distress	-0.12557168	-3.192496119
20	Pancreatitis	-0.12427274	-3.503574993
21	Bilateral lung agenesis	-0.12339036	-2.797545467
22	Tachypnea	-0.12322996	-2.849665879
23	Recurrent bronchitis	-0.11553134	-3.836767725
24	Aplasia/Hypoplasia of the lungs	-0.11495024	-3.392884915
25	Recurrent sinopulmonary infections	-0.11339318	-3.057362014
26	Breathing dysregulation	-0.10376545	-2.341549601
27	Hyperthyroidism	-0.10341751	-2.397427447
28	Pulmonary hypoplasia	-0.10239771	-4.329333817
29	Restrictive respiratory insufficiency	-0.09686876	-2.237186536
30	Peripheral pulmonary artery stenosis	-0.09525192	-2.246397079
31	Pulmonary emphysema	-0.09394704	-2.768707254

32	Dyskinesia	-0.09074843	-2.220919865
33	Respiratory distress	-0.0901347	-4.017150119
34	Neonatal breathing dysregulation	-0.08549531	-2.239474653
35	Pulmonary hypertension	-0.08377575	-4.408515204
36	Exertional dyspnea	-0.0819685	-2.159036259
37	Recurrent pneumonia	-0.07180055	-2.608476254
38	Pulmonic stenosis	-0.06601213	-2.987941357
39	Bronchiectasis	-0.06300449	-2.636930844
40	Pulmonary fibrosis	-0.05052834	-2.153132505
41	Asthma	-0.05001913	-4.54809959
42	Apnea	-0.03769275	-3.561558146
43	Nephrotic syndrome	-0.03602254	-1.785250832
44	Hemoptysis	-0.0346631	-1.550614418
45	Pulmonary infiltrates	-0.03418652	-1.526519182
46	Hypothyroidism	-0.03107043	-2.144734335
47	Apneic episodes precipitated by illness, fatigue, stress	-0.03005663	-1.838939205
48	Mitral regurgitation	-0.0297424	-1.462975676
49	Pierre-Robin sequence	-0.02928158	-1.796373496
50	Respiratory insufficiency due to muscle weakness	-0.02722833	-1.71511319
51	Weak cry	-0.02558294	-1.235787089
52	Pneumonia	-0.02492737	-1.875260383
53	Restrictive lung disease	-0.0195702	-1.264305182
54	Systemic lupus erythematosus	-0.01500633	-1.188005296
55	Emphysema	-0.01098312	-0.907668889
56	Pulmonary embolism	-0.00815448	-0.926916994
57	Chronic lung disease	-0.00505384	-0.89112733
58	Congestive heart failure	-0.00082701	-0.778526176
59	Protein-losing enteropathy	-0.00068482	-2.452095923
