# Supplementary material for: Lung disease network reveals impact of comorbidity on SARS-CoV-2 infection and opportunities of drug repurposing
Source: BMC Med Genomics. 2021 Sep 17;14:226. doi: 10.1186/s12920-021-01079-7 (PMC8447809; doi:10.1186/s12920-021-01079-7)
Supplement: Supplementary file 6 — Additional file 6. Table S5 and S6. Comparison of different community detection algorithms applied to SARS-CoV2 target network (STN) and protein modules generated using Spinglass algorithem. [file 12920_2021_1079_MOESM6_ESM.pdf]

Supplementary Table 5: Comparison of different community detection algorithms applied to SARS-CoV2 target network (STN)

**Algorithm**	**Modularity score**	**Number of community**	**Maximum and minimum size of the community**
Fast-greedy	0.5354479	18	Max. Size: 792<br>Min. Size: 13
Walktrap	0.4762713	129	Max. Size: 1050<br>Min. Size: 1
Louvain	0.5326101	23	Max. Size: 530<br>Min. Size: 35
Leading eigenvector	0.4066907	19	Max. Size: 1287<br>Min. Size: 1
Spinglass (total of 10 iterations was done with the different seed value)			
iteration1	0.5609735	21	Max. Size: 543<br>Min. Size: 27
iteration2	0.5587781	23	Max.size: 556<br>Min.size: 4
iteration3	0.5597632	23	Max.size: 555<br>Min.size: 5
iteration4	0.5599708	23	Max.size: 570<br>Min.size: 13
iteration5	0.5604577	22	Max.size: 537<br>Min.size: 13
iteration6	0.5591416	21	Max.size: 567<br>Min.size: 16
iteration7	0.5593708	21	Max.size: 579<br>Min.size: 4
iteration8	0.5594855	21	Max.size: 536<br>Min.size: 13
iteration9*	0.5603824	21	Max.size: 553<br>Min.size: 42
iteration10	0.5598939	23	Max.size: 598<br>Min.size: 5

* communities generated in iteration 9 were selected for functional protein module detection as detected modules are not too small or too large compared to the original network

Supplementary Table 6 : Protein modules generated using Spinglass algorithem (iteration 9)

Module	Number of Node	Number of edge	Number of SARS-CoV-2 target	Median values of GO (Biological Process) semantic similarity score
1	492	1345	63	0.246
2	345	479	50	0.226
3	320	500	28	0.214
4	226	300	23	0.237
5	357	460	37	0.188
6	378	495	16	0.21
7	220	325	15	0.23
8	312	486	13	0.228
9	379	471	11	0.279
10	88	111	11	0.26
11	304	450	8	0.267

12	212	229	8	0.217
13	45	50	7	0.222
14	289	325	6	0.249
15	140	157	5	0.322
16	95	138	5	0.236
17	83	90	5	0.247
18	57	56	4	0.329
19	42	41	3	0.222
20	553	552	2	0.2
21	107	106	2	0.214
