# Supplementary material for: Lung disease network reveals impact of comorbidity on SARS-CoV-2 infection and opportunities of drug repurposing
Source: BMC Med Genomics. 2021 Sep 17;14:226. doi: 10.1186/s12920-021-01079-7 (PMC8447809; doi:10.1186/s12920-021-01079-7)
Supplement: Supplementary file 7 — Additional file 7. Table S7. Disease association with functional protein modules. [file 12920_2021_1079_MOESM7_ESM.pdf]

Supplementary Table 7: Disease association with functional protein modules

Module1

S. No.	Disease	Dgree(Number of genes connected)
1	Ventricular septal defect	12
2	Respiratory insufficiency	10
3	Pneumonia	6
4	Respiratory distress	6
5	COVID19	5
6	Hypothyroidism	5
7	Neonatal respiratory distress	5
8	Neoplasm of the lung	5
9	Apnea	4
10	Dyskinesia	4
11	Emphysema	4
12	Pulmonary fibrosis	4
13	Pulmonary hypertension	4
14	Pulmonary hypoplasia	4
15	Recurrent pneumonia	4
16	Aspiration	3
17	Asthma	3
18	Bronchiectasis	3
19	Congestive heart failure	3
20	Respiratory insufficiency due to muscle weakness	3
21	Tracheomalacia	3
22	Abnormal lung lobation	2
23	Alveolar cell carcinoma	2
24	Bell-shaped thorax	2
25	Bronchomalacia	2
26	Chronic obstructive pulmonary disease	2
27	Congenital diaphragmatic hernia	2
28	Exertional dyspnea	2
29	Hyperventilation	2
30	Hypoventilation	2
31	Lung adenocarcinoma	2
32	Mitral regurgitation	2
33	Nephrotic syndrome	2
34	Recurrent bronchitis	2
35	Respiratory difficulties	2
36	Respiratory failure	2
37	Restrictive lung disease	2
38	Tracheoesophageal fistula	2
39	Weak cry	2
40	Abnormality of the aryepiglottic fold	1
41	Anomalous tracheal cartilage	1
42	Chronic bronchitis	1
43	Chronic lung disease	1
44	Congenital hypothyroidism	1
45	Crohn's disease	1
46	Hyponatremia	1

47	Intermittent hyperventilation	1
48	Interstitial pneumonitis	1
49	Mesothelioma	1
50	Neonatal hypoglycemia	1
51	Obstructive lung disease	1
52	Obstructive sleep apnea	1
53	Patent foramen ovale	1
54	Pierre-Robin sequence	1
55	Pleural effusion	1
56	Pneumothorax	1
57	Pulmonary artery aneurysm	1
58	Pulmonary artery hypoplasia	1
59	Pulmonary embolism	1
60	Pulmonic stenosis	1
61	Recurrent mycobacterial infections	1
62	Recurrent sinopulmonary infections	1
63	Respiratory tract infection	1
64	Thoracic hypoplasia	1

Module2

S. No.	Disease	Dgree(Number of genes connected)
1	Respiratory insufficiency	18
2	Congestive heart failure	10
3	Ventricular septal defect	10
4	Respiratory insufficiency due to muscle weakness	9
5	Hypothyroidism	8
6	Pulmonary hypertension	8
7	Asthma	7
8	Pneumonia	7
9	Pulmonary hypoplasia	6
10	Respiratory failure	6
11	Aplasia/Hypoplasia of the lungs	5
12	COVID19	5
13	Mitral regurgitation	5
14	Nephrotic syndrome	5
15	Apnea	4
16	Chronic lung disease	4
17	Pancreatitis	4
18	Pulmonary embolism	4
19	Pulmonary fibrosis	4
20	Respiratory distress	4
21	Tracheoesophageal fistula	4
22	Weak cry	4
23	Chronic obstructive pulmonary disease	3
24	Congenital diaphragmatic hernia	3
25	Hypoplastic pulmonary veins	3
26	Neoplasm of the lung	3
27	Pulmonic stenosis	3
28	Recurrent pneumonia	3

29	Apneic episodes precipitated by illness, fatigue, stress	2
30	Aspiration	2
31	Dyskinesia	2
32	Emphysema	2
33	Hemoptysis	2
34	Hyperthyroidism	2
35	Hyperventilation	2
36	Interstitial pulmonary disease	2
37	Neonatal breathing dysregulation	2
38	Neonatal hypoglycemia	2
39	Nocturnal hypoventilation	2
40	Pulmonary artery hypoplasia	2
41	Pulmonary infiltrates	2
42	Pulmonary insufficiency	2
43	Recurrent bronchiolitis	2
44	Restrictive lung disease	2
45	Tachypnea	2
46	Tracheal stenosis	2
47	Abnormal lung lobation	1
48	Acute pancreatitis	1
49	Agenesis of pulmonary vessels	1
50	Alveolar cell carcinoma	1
51	Atelectasis	1
52	Bell-shaped thorax	1
53	Bilateral lung agenesis	1
54	Breathing dysregulation	1
55	Bronchiectasis	1
56	Bronchogenic cyst	1
57	Bronchomalacia	1
58	Cor pulmonale	1
59	Diaphragmatic eventration	1
60	Hemolytic-uremic syndrome	1
61	Low cholesterol esterification rates	1
62	Lung segmentation defects	1
63	Myelofibrosis	1
64	Obstructive sleep apnea	1
65	Peripheral pulmonary artery stenosis	1
66	Pierre-Robin sequence	1
67	Pulmonary artery atresia	1
68	Pulmonary edema	1
69	Recurrent bronchitis	1
70	Recurrent lower respiratory tract infections	1
71	Recurrent mycobacterial infections	1
72	Recurrent sinopulmonary infections	1
73	Reduced vital capacity	1
74	Respiratory acidosis	1
75	Respiratory difficulties	1
76	Restrictive respiratory insufficiency	1
77	Systemic lupus erythematosus	1
78	Unilateral chest hypoplasia	1

79	Unilateral primary pulmonary dysgenesis	1

Module3

S. No.	Disease	Dgree(Number of genes connected)
1	Respiratory insufficiency	12
2	Congestive heart failure	8
3	Pulmonary hypertension	6
4	Ventricular septal defect	6
5	Mitral regurgitation	5
6	Pulmonary infiltrates	5
7	Respiratory distress	4
8	Respiratory failure	4
9	Respiratory insufficiency due to muscle weakness	4
10	Restrictive lung disease	4
11	Apnea	3
12	Chronic obstructive pulmonary disease	3
13	Congenital diaphragmatic hernia	3
14	Dyskinesia	3
15	Nephrotic syndrome	3
16	Pancreatitis	3
17	Pneumonia	3
18	Pulmonary artery hypoplasia	3
19	Aspiration	2
20	Asthma	2
21	Atelectasis	2
22	Bronchiectasis	2
23	COVID19	2
24	Exertional dyspnea	2
25	Hemoptysis	2
26	Hydrops fetalis	2
27	Hypothyroidism	2
28	Interstitial pulmonary disease	2
29	Patent foramen ovale	2
30	Pulmonary embolism	2
31	Pulmonary fibrosis	2
32	Pulmonic stenosis	2
33	Respiratory difficulties	2
34	Abnormal respiratory motile cilium morphology	1
35	Absent respiratory ciliary axoneme radial spokes	1
36	Acute pancreatitis	1
37	Bronchiolitis	1
38	Chronic bronchitis	1
39	Chronic lung disease	1
40	Hyperthyroidism	1
41	Hyponatremia	1
42	Hypoplastic pulmonary veins	1
43	Increased pulmonary vascular resistance	1
44	Lung segmentation defects	1
45	Mesothelioma	1

46	Mitral stenosis	1
47	Myelofibrosis	1
48	Neonatal respiratory distress	1
49	Neoplasm of the lung	1
50	Obstructive lung disease	1
51	Peripheral pulmonary artery stenosis	1
52	Pulmonary emphysema	1
53	Recurrent aspiration pneumonia	1
54	Recurrent bronchitis	1
55	Recurrent pneumonia	1
56	Recurrent sinopulmonary infections	1
57	Respiratory alkalosis	1
58	Respiratory insufficiency due to defective ciliary clearanc	1
59	Thoracic hypoplasia	1
60	Tracheoesophageal fistula	1

Module4

S. No.	Disease	Dgree(Number of genes connected)
1	Congestive heart failure	10
2	Ventricular septal defect	7
3	Mitral regurgitation	5
4	Neoplasm of the lung	5
5	Pulmonary artery hypoplasia	5
6	Hypothyroidism	4
7	Respiratory insufficiency due to muscle weakness	4
8	Abnormal lung lobation	3
9	Apnea	3
10	Nephrotic syndrome	3
11	COVID19	2
12	Pneumonia	2
13	Pulmonary fibrosis	2
14	Pulmonary hypertension	2
15	Respiratory difficulties	2
16	Respiratory insufficiency	2
17	Alveolar cell carcinoma	1
18	Aspiration	1
19	Congenital diaphragmatic hernia	1
20	Exertional dyspnea	1
21	Hyperventilation	1
22	Hypoventilation	1
23	Mesothelioma	1
24	Neonatal respiratory distress	1
25	Pituitary hypothyroidism	1
26	Pulmonary embolism	1
27	Pulmonary infiltrates	1
28	Pulmonic stenosis	1
29	Recurrent aspiration pneumonia	1
30	Respiratory failure	1
31	Restrictive lung disease	1

32	Weak cry	1
