# Supplementary material for: Lung disease network reveals impact of comorbidity on SARS-CoV-2 infection and opportunities of drug repurposing
Source: BMC Med Genomics. 2021 Sep 17;14:226. doi: 10.1186/s12920-021-01079-7 (PMC8447809; doi:10.1186/s12920-021-01079-7)
Supplement: Supplementary file 8 — Additional file 8. Table S8. Targets in functional protein modules and drugs from DrugBank database. [file 12920_2021_1079_MOESM8_ESM.pdf]

Supplementary table 8 : Targets in functional protein modules and drugs from DrugBank database

Target Name	Gene Name(symbol)	Module	Drug IDs	drug_name
Multidrug resistance-associated protein 1	ABCC1	module4	DB01138	Sulfinpyrazone
Multidrug resistance-associated protein 1	ABCC1	module4	DB06176	Romidepsin
5'-AMP-activated protein kinase subunit beta-1	PRKAB1	module1	DB00331	Metformin
72 kDa type IV collagenase	MMP2	module2	DB00786	Marimastat
Amiloride-sensitive sodium channel subunit alpha	SCNN1A	module1	DB00384	Triamterene
Amiloride-sensitive sodium channel subunit alpha	SCNN1A	module1	DB00594	Amiloride
Amyloid beta A4 protein	APP	module3	DB09151	Flutemetamol (18F)
Amyloid beta A4 protein	APP	module3	DB09149	Florbetapir (18F)
Amyloid beta A4 protein	APP	module3	DB09148	Florbetaben (18F)
Antithrombin-III	SERPINC1	module2	DB06822	Tinzaparin
Antithrombin-III	SERPINC1	module2	DB08813	Nadroparin
Antithrombin-III	SERPINC1	module2	DB01109	Heparin
Antithrombin-III	SERPINC1	module2	DB00569	Fondaparinux
Antithrombin-III	SERPINC1	module2	DB01225	Enoxaparin
Antithrombin-III	SERPINC1	module2	DB06779	Dalteparin
ATP-binding cassette sub-family G member 2	ABCG2	module3	DB04851	Biricodar
ATP-binding cassette sub-family G member 2	ABCG2	module3	DB11995	Avatrombopag
Beta-2 adrenergic receptor	ADRB2	module2	DB09082	Vilanterol
Beta-2 adrenergic receptor	ADRB2	module2	DB00871	Terbutaline
Beta-2 adrenergic receptor	ADRB2	module2	DB00938	Salmeterol
Beta-2 adrenergic receptor	ADRB2	module2	DB01001	Salbutamol
Beta-2 adrenergic receptor	ADRB2	module2	DB00867	Ritodrine
Beta-2 adrenergic receptor	ADRB2	module2	DB11124	Racepinephrine
Beta-2 adrenergic receptor	ADRB2	module2	DB06814	Protokylol
Beta-2 adrenergic receptor	ADRB2	module2	DB01366	Procaterol
Beta-2 adrenergic receptor	ADRB2	module2	DB01291	Pirbuterol
Beta-2 adrenergic receptor	ADRB2	module2	DB00816	Orciprenaline
Beta-2 adrenergic receptor	ADRB2	module2	DB09080	Olodaterol
Beta-2 adrenergic receptor	ADRB2	module2	DB01214	Metipranolol
Beta-2 adrenergic receptor	ADRB2	module2	DB13139	Levosalbutamol
Beta-2 adrenergic receptor	ADRB2	module2	DB01210	Levobunolol
Beta-2 adrenergic receptor	ADRB2	module2	DB05039	Indacaterol

Beta-2 adrenergic receptor	ADRB2	module2	DB00983	Formoterol
Beta-2 adrenergic receptor	ADRB2	module2	DB01288	Fenoterol
Beta-2 adrenergic receptor	ADRB2	module2	DB11587	Etafedrine
Beta-2 adrenergic receptor	ADRB2	module2	DB01407	Clenbuterol
Beta-2 adrenergic receptor	ADRB2	module2	DB00521	Carteolol
Beta-2 adrenergic receptor	ADRB2	module2	DB01274	Arformoterol
Beta-2 adrenergic receptor	ADRB2	module2	DB01118	Amiodarone
Calcium-transporting ATPase type 2C member 1	ATP2C1	module3	DB00228	Enflurane
Catechol O-methyltransferase	COMT	module2	DB11632	Opicapone
Catechol O-methyltransferase	COMT	module2	DB00494	Entacapone
Coagulation factor X	F10	module2	DB06228	Rivaroxaban
Coagulation factor X	F10	module2	DB01109	Heparin
Coagulation factor X	F10	module2	DB00569	Fondaparinux
Coagulation factor X	F10	module2	DB01225	Enoxaparin
Coagulation factor X	F10	module2	DB09075	Edoxaban
Coagulation factor X	F10	module2	DB12364	Betrixaban
Coagulation factor X	F10	module2	DB09258	Bemiparin
Coagulation factor X	F10	module2	DB06605	Apixaban
Corticosteroid 11-beta-dehydrogenase isozyme 1	HSD11B1	module2	DB13751	Glycyrrhizic acid
Cytochrome P450 2D6	CYP2D6	module2	DB11994	Diacerein
Cytochrome P450 2E1	CYP2E1	module2	DB11994	Diacerein
DNA topoisomerase 1	TOP1	module1	DB01030	Topotecan
DNA topoisomerase 1	TOP1	module1	DB05630	Sodium stibogluconate
DNA topoisomerase 1	TOP1	module1	DB00762	Irinotecan
DNA topoisomerase I, mitochondrial	TOP1MT	module1	DB00762	Irinotecan
Dopamine beta-hydroxylase	DBH	module2	DB00988	Dopamine
Dopamine beta-hydroxylase	DBH	module2	DB00822	Disulfiram
Epidermal growth factor receptor	EGFR	module2	DB09330	Osimertinib
Epidermal growth factor receptor	EGFR	module2	DB11828	Neratinib
Epidermal growth factor receptor	EGFR	module2	DB01259	Lapatinib
Epidermal growth factor receptor	EGFR	module2	DB00317	Gefitinib
Epidermal growth factor receptor	EGFR	module2	DB00530	Erlotinib
Epidermal growth factor receptor	EGFR	module2	DB11963	Dacomitinib
Epidermal growth factor receptor	EGFR	module2	DB12267	Brigatinib

Epidermal growth factor receptor	EGFR	module2	DB08916	Afatinib
Estrogen receptor	ESR1	module1	DB09070	Tibolone
Estrogen receptor	ESR1	module1	DB00675	Tamoxifen
Estrogen receptor	ESR1	module1	DB09318	Synthetic Conjugated Estrogens, B
Estrogen receptor	ESR1	module1	DB09317	Synthetic Conjugated Estrogens, A
Estrogen receptor	ESR1	module1	DB00481	Raloxifene
Estrogen receptor	ESR1	module1	DB04575	Quinestrol
Estrogen receptor	ESR1	module1	DB09369	Polyestradiol phosphate
Estrogen receptor	ESR1	module1	DB04938	Ospemifene
Estrogen receptor	ESR1	module1	DB01357	Mestranol
Estrogen receptor	ESR1	module1	DB06202	Lasofoxifene
Estrogen receptor	ESR1	module1	DB00947	Fulvestrant
Estrogen receptor	ESR1	module1	DB00255	Diethylstilbestrol
Estrogen receptor	ESR1	module1	DB00890	Dienestrol
Estrogen receptor	ESR1	module1	DB01406	Danazol
Estrogen receptor	ESR1	module1	DB00286	Conjugated estrogens
Estrogen receptor	ESR1	module1	DB00882	Clomifene
Estrogen receptor	ESR1	module1	DB06401	Bazedoxifene
Estrogen receptor beta	ESR2	module1	DB00675	Tamoxifen
Estrogen receptor beta	ESR2	module1	DB00481	Raloxifene
Estrogen receptor beta	ESR2	module1	DB06202	Lasofoxifene
Estrogen receptor beta	ESR2	module1	DB00255	Diethylstilbestrol
Estrogen receptor beta	ESR2	module1	DB00286	Conjugated estrogens
Exportin-1	XPO1	module1	DB11942	Selinexor
Glutathione S-transferase Mu 1	GSTM1	module3	DB00608	Chloroquine
Hemoglobin subunit beta	HBB	module1	DB09112	Nitrous acid
Hemoglobin subunit beta	HBB	module1	DB13995	Ferric pyrophosphate citrate
High affinity nerve growth factor receptor	NTRK1	module1	DB08896	Regorafenib
High affinity nerve growth factor receptor	NTRK1	module1	DB14723	Larotrectinib
High affinity nerve growth factor receptor	NTRK1	module1	DB11986	Entrectinib
Histone deacetylase 11	HDAC11	module1	DB05015	Belinostat
Histone deacetylase 5	HDAC5	module1	DB06603	Panobinostat
Histone deacetylase 5	HDAC5	module1	DB05015	Belinostat
Histone deacetylase 7	HDAC7	module4	DB06603	Panobinostat

Histone deacetylase 7	HDAC7	module4	DB05015	Belinostat
Histone deacetylase 8	HDAC8	module1	DB06603	Panobinostat
Histone deacetylase 8	HDAC8	module1	DB05015	Belinostat
Histone deacetylase 9	HDAC9	module1	DB00313	Valproic acid
Histone deacetylase 9	HDAC9	module1	DB06603	Panobinostat
Histone deacetylase 9	HDAC9	module1	DB05015	Belinostat
Histone-lysine N-methyltransferase EZH2	EZH2	module1	DB12887	Tazemetostat
Histone-lysine N-methyltransferase EZH2	EZH2	module1	DB14581	CPI-1205
Inosine-5'-monophosphate dehydrogenase 1	IMPDH1	module1	DB00688	Mycophenolate mofetil
Inosine-5'-monophosphate dehydrogenase 2	IMPDH2	module1	DB00688	Mycophenolate mofetil
Intercellular adhesion molecule 1	ICAM1	module1	DB08818	Hyaluronic acid
Intermediate conductance calcium-activated potassium channel protein 4	KCNN4	module3	DB09089	Trimebutine
Intermediate conductance calcium-activated potassium channel protein 4	KCNN4	module3	DB00257	Clotrimazole
Microtubule-associated protein tau	MAPT	module4	DB01229	Paclitaxel
Microtubule-associated protein tau	MAPT	module4	DB01248	Docetaxel
Mitogen-activated protein kinase 3	MAPK3	module1	DB01169	Arsenic trioxide
Multidrug resistance-associated protein 1	ABCC1	module4	DB04851	Biricodar
NAD-dependent protein deacylase sirtuin-5, mitochondrial	SIRT5	module1	DB04786	Suramin
Neprilysin	MME	module1	DB09292	Sacubitril
Neprilysin	MME	module1	DB00886	Omapatrilat
Nuclear factor NF-kappa-B p100 subunit	NFKB2	module1	DB13751	Glycyrrhizic acid
Peptidyl-prolyl cis-trans isomerase A	PPIA	module3	DB00091	Cyclosporine
Peroxiredoxin-5, mitochondrial	PRDX5	module2	DB00995	Auranofin
Potassium voltage-gated channel subfamily D member 3	KCND3	module1	DB06217	Vernakalant
Potassium voltage-gated channel subfamily D member 3	KCND3	module1	DB04855	Dronedarone
Potassium voltage-gated channel subfamily D member 3	KCND3	module1	DB06637	Dalfampridine
Prostaglandin E2 receptor EP3 subtype	PTGER3	module4	DB00929	Misoprostol
Prostaglandin E2 receptor EP3 subtype	PTGER3	module4	DB00917	Dinoprostone
Prostaglandin E2 receptor EP3 subtype	PTGER3	module4	DB00905	Bimatoprost
Prostaglandin F2-alpha receptor	PTGFR	module2	DB00287	Travoprost
Prostaglandin F2-alpha receptor	PTGFR	module2	DB08819	Tafluprost
Prostaglandin F2-alpha receptor	PTGFR	module2	DB11660	Latanoprostene bunod
Prostaglandin F2-alpha receptor	PTGFR	module2	DB00654	Latanoprost
Prostaglandin F2-alpha receptor	PTGFR	module2	DB01160	Dinoprost tromethamine

Prostaglandin F2-alpha receptor	PTGFR	module2	DB00905	Bimatoprost
Protein kinase C delta type	PRKCD	module2	DB00675	Tamoxifen
Protein kinase C iota type	PRKCI	module4	DB00675	Tamoxifen
Proto-oncogene tyrosine-protein kinase Src	SRC	module2	DB01254	Dasatinib
Receptor tyrosine-protein kinase erbB-4	ERBB4	module4	DB08916	Afatinib
Sigma non-opioid intracellular receptor 1	SIGMAR1	module4	DB11186	Pentoxyverine
Sigma non-opioid intracellular receptor 1	SIGMAR1	module4	DB00652	Pentazocine
Sigma non-opioid intracellular receptor 1	SIGMAR1	module4	DB00514	Dextromethorphan
Sodium/potassium-transporting ATPase subunit alpha-1	ATP1A1	module2	DB01092	Ouabain
Sodium/potassium-transporting ATPase subunit alpha-1	ATP1A1	module2	DB00903	Etacrynic acid
Sodium/potassium-transporting ATPase subunit alpha-1	ATP1A1	module2	DB00390	Digoxin
Sodium/potassium-transporting ATPase subunit alpha-1	ATP1A1	module2	DB01396	Digitoxin
Sodium/potassium-transporting ATPase subunit alpha-1	ATP1A1	module2	DB01078	Deslanoside
Sodium/potassium-transporting ATPase subunit alpha-1	ATP1A1	module2	DB01188	Ciclopirox
Sodium/potassium-transporting ATPase subunit alpha-1	ATP1A1	module2	DB01158	Bretylium
Sodium/potassium-transporting ATPase subunit alpha-1	ATP1A1	module2	DB01430	Almitrine
Sodium/potassium-transporting ATPase subunit alpha-1	ATP1A1	module2	DB00511	Acetyldigitoxin
Sterol O-acyltransferase 1	SOAT1	module3	DB00973	Ezetimibe
Tissue-type plasminogen activator	PLAT	module2	DB00513	Aminocaproic acid
Transferrin receptor protein 1	TFRC	module3	DB14520	Tetraferric tricitrate decahydrate
Transferrin receptor protein 1	TFRC	module3	DB13949	Ferric cation
Vascular endothelial growth factor receptor 3	FLT4	module2	DB08896	Regorafenib
Vascular endothelial growth factor receptor 3	FLT4	module2	DB06589	Pazopanib
Vascular endothelial growth factor receptor 3	FLT4	module2	DB09079	Nintedanib
Vascular endothelial growth factor receptor 3	FLT4	module2	DB09078	Lenvatinib
Vascular endothelial growth factor receptor 3	FLT4	module2	DB06626	Axitinib
Voltage-dependent calcium channel subunit alpha-2/delta-1	CACNA2D1	module3	DB00230	Pregabalin
Voltage-dependent calcium channel subunit alpha-2/delta-1	CACNA2D1	module3	DB01054	Nitrendipine
Voltage-dependent calcium channel subunit alpha-2/delta-1	CACNA2D1	module3	DB00401	Nisoldipine
Voltage-dependent calcium channel subunit alpha-2/delta-1	CACNA2D1	module3	DB06712	Nilvadipine
Voltage-dependent calcium channel subunit alpha-2/delta-1	CACNA2D1	module3	DB00622	Nicardipine
Voltage-dependent calcium channel subunit alpha-2/delta-1	CACNA2D1	module3	DB00270	Isradipine
Voltage-dependent calcium channel subunit alpha-2/delta-1	CACNA2D1	module3	DB00996	Gabapentin
Voltage-dependent calcium channel subunit alpha-2/delta-1	CACNA2D1	module3	DB01023	Felodipine

V-type proton ATPase subunit B, brain isoform	ATP6V1B2	module3	DB05260	Gallium nitrate

* Drugs marked with red colour are in clinical trials for COVID19
