# Supplementary material for: Lung disease network reveals impact of comorbidity on SARS-CoV-2 infection and opportunities of drug repurposing
Source: BMC Med Genomics. 2021 Sep 17;14:226. doi: 10.1186/s12920-021-01079-7 (PMC8447809; doi:10.1186/s12920-021-01079-7)
Supplement: Supplementary file 9 — Additional file 9. Table S9. Proximity between targets and COVID-19 disease module. [file 12920_2021_1079_MOESM9_ESM.pdf]

Supplementary table 9: proximity between targets and COVID-19 disease module

S.No.	Gene symbol	z-score	module
1	HBB	-3.156973239	module 1
2	HDAC11	-2.862479031	module 1
3	HDAC5	-2.614718972	module 1
4	HDAC8	-3.16173851	module 1
5	HDAC9	-3.03803864	module 1
6	ICAM1	-2.855382902	module 1
7	IMPDH1	-3.315953677	module 1
8	IMPDH2	-4.645760869	module 1
9	KCND3	-3.182819349	module 1
10	MAPK3	-2.860776091	module 1
11	MME	-2.982209808	module 1
12	NFKB2	-2.92128457	module 1
13	NTRK1	-2.616547825	module 1
14	PRKAB1	-2.844386842	module 1
15	SCNN1A	-3.232815906	module 1
16	SIRT5	-5.00901849	module 1
17	TOP1	-2.927604524	module 1
18	TOP1MT	-3.345539451	module 1
19	XPO1	-2.607468519	module 1
20	ESR1	-2.497724	module 1
21	ESR2	-2.506792	module 1
22	EZH2	-2.520663	module 1
23	ADRB2	-2.769890586	module2
24	ATP1A1	-3.002450564	module2
25	COMT	-4.949845632	module2
26	CYP2D6	-3.331706753	module2
27	CYP2E1	-3.32848983	module2
28	DBH	-3.313628572	module2
29	EGFR	-2.612875217	module2
30	F10	-3.335939796	module2
31	FLT4	-3.273112429	module2
32	HSD11B1	-3.313885171	module2
33	MMP2	-3.289647589	module2
34	PLAT	-4.975815363	module2
35	PRDX5	-3.275684574	module2
36	PRKCD	-2.980639925	module2
37	PTGFR	-3.327477	module2
38	SERPINC1	-3.325091122	module2
39	SRC	-2.846152064	module2
40	ABCG2	-3.344169446	module3
41	APP	-3.341669828	module3
42	ATP2C1	-3.325716752	module3
43	ATP6V1B2	-3.230371118	module3
44	CACNA2D1	-3.266574043	module3
45	GSTM1	-3.340040417	module3
46	KCNN4	-3.32648446	module3
47	PPIA	-2.972216132	module3
48	SOAT1	-3.242287848	module3

49	TFRC	-3.031958546	module3
50	ABCC1	-5.055420052	module4
51	ERBB4	-3.0886327	module4
52	HDAC7	-3.027971187	module4
53	MAPT	-3.017172869	module4
54	PRKCI	-3.008434285	module4
55	PTGER3	-2.932693225	module4
56	SIGMAR1	-5.035001701	module4
