# Supplementary material for: Lung disease network reveals impact of comorbidity on SARS-CoV-2 infection and opportunities of drug repurposing
Source: BMC Med Genomics. 2021 Sep 17;14:226. doi: 10.1186/s12920-021-01079-7 (PMC8447809; doi:10.1186/s12920-021-01079-7)
Supplement: Supplementary file 10 — Additional file 10. Table S10. Proximity between drugs in clinical trial and COVID-19 disease module. [file 12920_2021_1079_MOESM10_ESM.pdf]

Supplementary table 10: proximity between drugs in clinical trial and COVID-19 disease module

Drug	z-score	module
Enoxaparin	-3.24981	module 2
Heparin	-3.51572	module 2
Formoterol	-3.19823	module2
Amiodarone	-3.00222	module2
Rivaroxaban	-3.06403	module2
Chloroquine	-3.05553	module3
Nintedanib	-3.12148	module2
Nitrendipine	-2.89249	module3
Nicardipine	-3.12367	module3
Felodipine	-3.08464	module3
